# Genetic Patterns in European Geometrid Moths Revealed by the Barcode Index Number (BIN) System

**DOI:** 10.1371/journal.pone.0084518

**Published:** 2013-12-17

**Authors:** Axel Hausmann, H. Charles J. Godfray, Peter Huemer, Marko Mutanen, Rodolphe Rougerie, Erik J. van Nieukerken, Sujeevan Ratnasingham, Paul D. N. Hebert

**Affiliations:** 1 Entomology Department, Bavarian State Collection of Zoology, Munich, Germany; 2 Bavarian Natural History Collections, Munich, Germany; 3 Department of Zoology, University of Oxford, Oxford, United Kingdom; 4 Naturwissenschaftliche Sammlungen, Tiroler Landesmuseen Betriebsgesellschaft, Innsbruck, Austria; 5 Zoological Museum of the Department of Biology, University of Oulu, Oulu, Finland; 6 Laboratoire d'Ecologie, Université de Rouen, Mont-Saint-Aignan, France; 7 Unité de Recherche en Zoologie Forestière, Orléans, France; 8 Naturalis Biodiversity Center, Leiden, The Netherlands; 9 Biodiversity Institute of Ontario, University of Guelph, Guelph, Canada; Chang Gung University, Taiwan

## Abstract

**Background:**

The geometrid moths of Europe are one of the best investigated insect groups in traditional taxonomy making them an ideal model group to test the accuracy of the Barcode Index Number (BIN) system of BOLD (Barcode of Life Datasystems), a method that supports automated, rapid species delineation and identification.

**Methodology/Principal Findings:**

This study provides a DNA barcode library for 219 of the 249 European geometrid moth species (88%) in five selected subfamilies. The data set includes COI sequences for 2130 specimens. Most species (93%) were found to possess diagnostic barcode sequences at the European level while only three species pairs (3%) were genetically indistinguishable in areas of sympatry. As a consequence, 97% of the European species we examined were unequivocally discriminated by barcodes within their natural areas of distribution. We found a 1:1 correspondence between BINs and traditionally recognized species for 67% of these species. Another 17% of the species (15 pairs, three triads) shared BINs, while specimens from the remaining species (18%) were divided among two or more BINs. Five of these species are mixtures, both sharing and splitting BINs. For 82% of the species with two or more BINs, the genetic splits involved allopatric populations, many of which have previously been hypothesized to represent distinct species or subspecies.

**Conclusions/Significance:**

This study confirms the effectiveness of DNA barcoding as a tool for species identification and illustrates the potential of the BIN system to characterize formal genetic units independently of an existing classification. This suggests the system can be used to efficiently assess the biodiversity of large, poorly known assemblages of organisms. For the moths examined in this study, cases of discordance between traditionally recognized species and BINs arose from several causes including overlooked species, synonymy, and cases where DNA barcodes revealed regional variation of uncertain taxonomic significance.

## Introduction

In the decade since DNA barcodes were proposed as a tool for species identification [1], many studies have shown that this approach yields excellent results for most groups of animals [2-4]. Furthermore, DNA barcodes based on the mitochondrial CO1 gene have gained acceptance as an important molecular component of integrated taxonomic analyses [5-8]. As the number of DNA barcode campaigns has increased, and large libraries of barcodes have been assembled, efforts have been directed towards the development of methods for automated species delineation [9-12]. Initial work in this area focused on the development of approaches enabling the estimation of species boundaries within the sequences gathered in a particular study. Ratnasingham & Hebert [13] recently implemented the Barcode Index Number (BIN) system as a registry for all records on the Barcode of Life Datasystems (BOLD, www.boldsystems.org) [14,15]. The BIN system employs a two-stage algorithm (Refined Single Linkage) that couples single linkage and Markov clustering to assign sequences to a sequence cluster that is subsequently assigned a unique identifier termed a Barcode Index Number. The Refined Single Linkage algorithm matches the taxonomic performance of competing approaches, but couples this with protocols that are simple enough to allow the automated assignment of all new barcode records to a BIN. BOLD currently hosts nearly 2.5 million DNA barcode sequences, deriving from more than 190,000 formally named species, and is used daily by hundreds of researchers. The development of the BIN system provides a new tool accessible to all users. Although the BIN system is potentially of great importance to the barcode research community, its performance has seen limited examination.

In this paper we report the assembly of a comprehensive DNA barcode library for a taxonomically very well-known fauna - five of the seven subfamilies of European geometrid moths. We use this data to test the correspondence between the BIN system currently implemented in BOLD and recognised species boundaries.

The specific aims of this study are (a) to present a public data release of DNA barcodes for five subfamilies of European geometrids, (b) to critically analyse intraspecific variation and interspecific distances in the barcode region and how they relate to traditionally recognized species, and (c) to test the correspondence between BINs and traditionally recognized species. The latter test is important because DNA barcode records are growing rapidly, and often involve poorly known faunas or include records that have received little or no taxonomic scrutiny. Automated species recognition such as that provided by the BIN system can be valuable in such circumstances to (1) refine current species determinations based on morphology; (2) assist in reliably and accurately assigning unknown samples to an existing species in BOLD; and (3) provide a first estimate of species diversity in groups where a taxonomic framework is missing or poor. If automated recognition performs well for groups whose taxonomy is accurately known, it will provide confidence that the BIN system can be used to estimate species richness in biodiversity surveys involving areas such as the tropics where basic taxonomic resources are unavailable or very limited.

This study benefited from a strong network of European projects, initiatives and campaigns: the Barcoding Fauna Bavarica project (BFB; [16]; www.faunabavarica.de), the German Barcode of Life project (GBOL; cf. www.bolgermany.de), the Finnish Barcode of Life project (FinBOL; cf. www.finbol.org), as well as Lepidoptera barcoding campaigns in the Alps [17], the Netherlands, Higher Normandy, France, Italy, Croatia and the United Kingdom. These efforts now fall under the aegis of the Barcoding European Lepidoptera campaign which was launched in 2011 at the XVIIth European Congress of Lepidopterology in Luxembourg. 

Summaries of the taxonomy and nomenclature of European geometrids are provided by the Fauna of Europe project [18], and the book series ‘Geometrid Moths of Europe’ [19,20].

## Materials and Methods

### Sampling

Specimens were sampled across Europe which was defined using the same boundaries as in Hausmann [19,20]: i.e. from Iceland to the Urals and northern foothills of the Caucasus, and from Malta to the Northern Cape, excluding Cyprus and Macaronesia.

This study covers five of the seven subfamilies of Geometridae known from Europe: Archiearinae, Desmobathrinae, Orthostixinae, Geometrinae and Sterrhinae, which jointly include 249 recognized species [19,20]. The two other subfamilies, the Larentiinae and Ennominae, include another 733 species which will be addressed in another paper. Specimens were sampled by the community of lepidopterists involved in assembling a comprehensive DNA barcode library for European Lepidoptera. In total, DNA was extracted from 2150 European specimens, representing 195 different species. Specimens collected outside Europe were included for 32 of the 54 missing species, adding an additional 520 specimens. The data set is somewhat geographically biased with Eastern Europe underrepresented. The largest samples derive from Italy (619 sequences; 107 species = 78% coverage of the national fauna), Germany (227 sequences; 55 species = 73%), Spain (215 sequences; 81 species = 48%), United Kingdom (146 sequences; 30 species = 55%), Finland (140 sequences; 43 species = 90%), France (118 sequences; 51 species = 36%) and Greece (88 sequences; 54 species = 50%).

### DNA analysis

PCR amplification and DNA sequencing was performed at the Canadian Centre for DNA Barcoding following standard high-throughput protocols [21,22] that can be accessed at http://www.dnabarcoding.ca/pa/ge/research/protocols. PCR amplification with a single pair of primers usually recovered a 658 bp region near the 5’ terminus of the mitochondrial cytochrome *c* oxidase I (COI) gene that includes the standard 648 bp barcode region for the animal kingdom [1]. All barcoded voucher specimens are listed in Appendix S1 and Appendix S2. A DNA extract from each specimen is cryopreserved at the Canadian Centre for DNA Barcoding and in the DNA-Bank facility of the Bavarian State Collection of Zoology (see http://www.zsm.mwn.de/dnabank/). All sequence records together with images, voucher deposition details, GenBank accession numbers, GPS coordinates, sequence and trace files are available on BOLD as a single citable dataset (http://dx.doi.org/10.5883/DS-GEOEU1). The sequences are also available on GenBank (Accession numbers see Appendix S2).

### Data analysis

The analyses of genetic distances were restricted to sequences > 500 bp unless stated otherwise (in single cases, shorter sequences are discussed in Appendix S1).

Sequence divergences for the barcode region were quantified using the Kimura 2 Parameter model, employing the analytical tools in BOLD (BOLD alignment, pairwise deletion). Genetic distances between species are reported as minimum pairwise distances, while intraspecific variation is reported as mean and maximum pairwise distances. 

Each specimen with a sequence longer than 500bp (listed in Appendix S1, similarities visualized in a neighbor joining tree, Appendix S5, records analysed in March 2013) automatically gained a BIN assignment on BOLD. It should be noted that BIN assignments are dynamically updated as new records are added to BOLD. BINs may be merged when genetically intermediate specimens are added or split when new records reveal clear sequence divergence structure. A nomenclature system, based on a set of simple rules [13], has been implemented in BOLD to make changes in assignments straightforward to trace and easy to understand. 

Whenever a discrepancy was found between DNA-based and standard taxonomy, the specimen was examined to confirm that its morphological identification was correct, and the alignment and trace files were carefully re-examined. 

The closest geographic distances between barcoded individuals of merged species or split clusters were measured in Google Earth, rounding to the nearest 10km-classes. 

## Results

### Traditional and BIN species delineations

In total 2,150 European individuals of the five geometrid subfamilies, including many older specimens (maximum 90y, mean 10.3y), were submitted for DNA barcoding. Sixteen (0.8%) generated sequences that proved to be contaminants, while 393 (18%) generated no sequence information.

**Figure 1:**
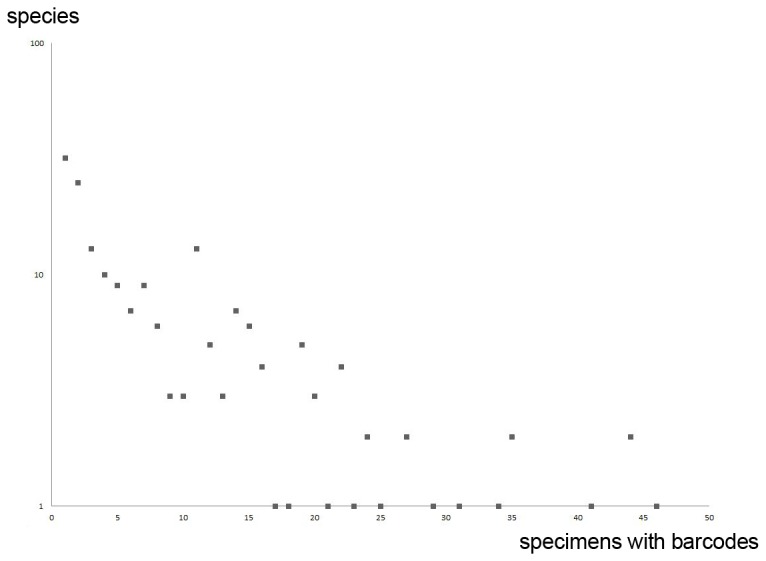
DNA barcoded specimens per species. Sampling statistics for the 1610 barcoded individuals (>500bp; from European countries) belonging to 183 species. On average there were 8.8 DNA barcodes from European specimens per species. However, 32 species were represented by a single DNA barcode, while the most heavily analyzed species had 46 records.

**Figure 2:**
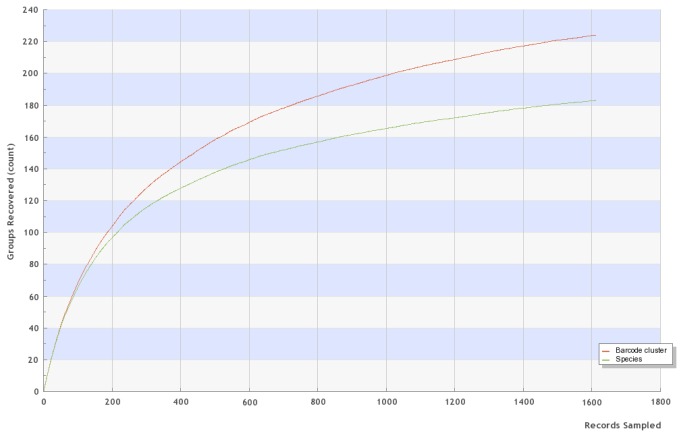
Accumulation curve for the 183 species and 224 BINs with DNA barcodes from European specimens. Accumulation curve (from BOLD database; randomized; 100 iterations) for the 1610 barcoded individuals (>500bp; from European countries).

Sequences were obtained for 1,741 European geometrid specimens representing 187 species. Most sequences (1,610 individuals belonging to 183 species and 224 BINs; cf. Figures 1-2) were longer than 500 bp, meeting the length requirement for DNA barcode status [14]. Additional data for 32 European species was based on specimens collected outside Europe (see Appendix S1). Most were from Turkey or the Middle East (20 species), but some derived from North Africa (9) and Russia (3). Thus, in total, COI barcode records were available for 215 species (2,130 DNA barcodes) in the five geometrid subfamilies included in this study (86% of the total fauna) with more limited sequences for a further four. 30 species are without DNA barcodes (see Appendix S3).

Analysis indicated that the 215 morphological species were assigned to 253 BINs. The morphological species could be separated into three categories: (i) those (67%) in which there was a perfect match between morphological species and BINs (145 species); (ii) those (17%) where different species shared a BIN assignment or where some specimens of a species shared a BIN with another morphological species (37 species placed in 18 BINs); (iii) those (18%) morphological species placed in more than one BIN (38 species placed in 98 BINs). Categories (ii) and (iii) include five species which are mixtures and are, hence, included in both categories: *Pseudoterpna pruinata, P. coronillaria, Idaea humiliata, I. seriata* and *Scopula confinaria*.

Our results indicate that DNA barcodes discriminate 93% of the European geometrid species examined in our study at a continental level. We define ‘diagnostic’ barcode clusters as those with a consistent difference from all other species recognized by past taxonomic efforts. We emphasize that DNA barcodes are considered diagnostic even in cases where specimens of a species were assigned to two or more distinct BINs, so long as those clusters contain only representatives of that species. DNA barcodes were also considered diagnostic in a few cases where species are genetically distinct, but with a very low divergence causing their assignment to a single BIN. In all these cases, the identification of an unknown specimen through matching its sequence to those in the reference library (a process that we subsequently refer to as “re-identification”) leads to a correct result. 

Re-identification accuracy increases when specimens are collected from smaller study areas; as is shown in Table 1 for Finland, United Kingdom, Germany, southern Italy, Sicily and Sardinia. In these examples, the merger of sequences from two morphological species into a single BIN was only observed in Finland (3 cases) and Germany (2). Most cases where the same species contained two or more BINs occurred on Sardinia (3 cases).

**Table 1:** Re-identification success for geometrid specimens collected in different regions of Europe based on barcode results.

**Country**	**Diagnostic barcodes**	**Species-BIN matches**	**Species with BINs**	**Number of specimens**
[Europe*]	93%	67%	215	2130
Finland	91%	81%	43	140
United Kingdom	100%	93%	30	146
Germany	93%	87%	55	227
Southern Italy**	100%	95%	76	254
Sicily	100%	100%	42	125
Sardinia	100%	91%	33	100

Re-identification success was measured as the percentage of diagnostic barcodes and exact species-BIN matches (see text for further explanation). * Including data from adjacent countries. **Specimens were collected from Calabria and Basilicata.

Considering all species in the five subfamilies examined in this study, European geometrids showed a mean interspecific genetic distance of 12.6% (SE < 0.01; n = 880,940 comparisons of barcodes > 500bp). By comparison, congeneric species averaged 8.8% divergence (SE < 0.01; n = 276,240), while the mean nearest neighbour divergence was 4.41% (n = 210). Mean and maximum intraspecific variation averaged 0.70% and 1.56% respectively based upon traditionally delimited species, even when those assigned to more than one BIN were treated as a single species (n = 149 species represented by more than one specimen). By comparison, the mean and maximum intra-BIN variation averaged 0.36% and 0.73% respectively (n= 189 BINs represented by more than one specimen).

Detailed barcode gap analysis of the three most species-rich genera (*Idaea, Scopula*, *Cyclophora*) revealed that *Cyclophora* showed the smallest interspecific genetic distances while the largest were in *Idaea* (Table 2). Reflecting this fact, a relatively large number of traditionally recognized species of *Idaea* were assigned to two or three BINs (15/88=17%).

**Table 2:** Barcode gap analysis for some species-rich taxa of European geometrids.

**Taxon**	**Number of species**	**Number of specimens**	**Mean intraspecific variation**	**Mean maximum variation**	**Mean distance to congenerics**	**Mean nearest neighbour divergence**
[5 subfamilies]	183	1610	0.70%	1.56%	8.8%	4.4%
*Idaea*	88	710	0.75%	1.64%	9.0%	4.5%
*Scopula*	33	309	0.57%	1.23%	9.4%	4.1%
*Cyclophora*	13	151	0.11%	0.50%	4.0%	1.7%

Total number of species and specimens from Europe with barcodes >500bp. Mean intraspecific variation and maximum variation (Kimura two-parameter genetic distances) are calculated for all representatives of the different taxa. The mean (pairwise) distance to congenerics was calculated using the distance summary function in BOLD. Mean nearest neighbour divergence was averaged from the minimum nearest neighbour divergences given for all species in Appendix S1.

### Multiple species assigned to the same BIN

**Figure 3:**
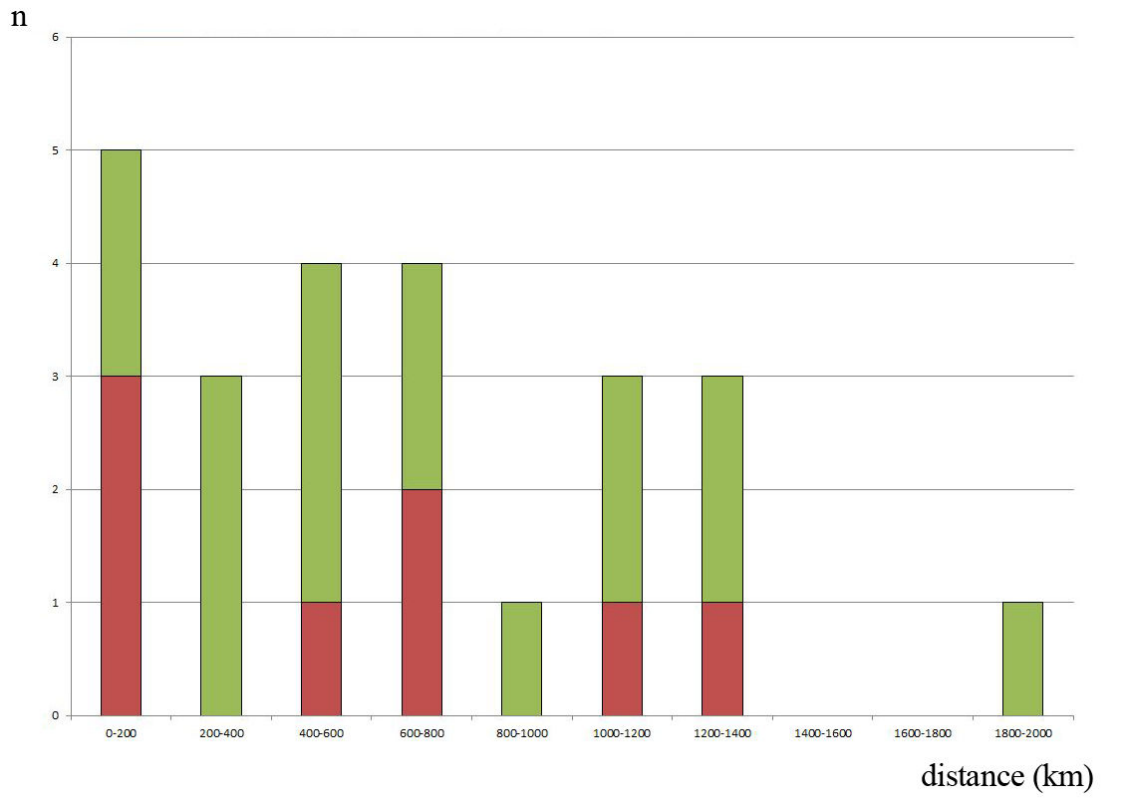
BIN-sharing species pairs versus shortest distance between barcoded vouchers of both taxa. Species pairs assigned to a single BIN, but that possess diagnostic sequence differences are shown in green, while exactly barcode-sharing pairs are in red; n = number of species pairs; each species in a triad shares a BIN with two other species, resulting in three entries for one triad. Distance was plotted in 200 km classes; for exact information cf. Appendix S1.

Members of 37 traditionally recognized European geometrid species (17%) shared a BIN with another species. Twenty eight of these cases involved a species pair, while the other nine cases involved a species triad. In one of these species pairs (*Pseudoterpna pruinata & P. coronillaria*) there is a double BIN-sharing in two lineages of each species. In four cases (*Pseudoterpna coronillaria, Idaea humiliata, I. seriata, Scopula confinaria*; see Appendix S1) a species was assigned to a unique BIN over part of its range, but shared a BIN with a second species in another region. Diagnostic sequence differences separated 21 species that were placed in pairs or triads with a single BIN. We found only eight species pairs whose DNA barcode sequences were indistinguishable. Of these, just three species pairs (3% of the fauna) were sympatric (see Figure 3) and so not amenable to molecular re-identification: *Boudinotiana notha & B. touranginii, Cyclophora punctaria & C. quercimontaria* and *Chlorissa viridata & C. cloraria*. The last species pair may represent a case of parapatry with a hybrid zone rather than true sympatry. In one other case (*Scopula frigidaria & S. ternata*), barcode sharing is rare and may reflect infrequent F1 hybrids or rare introgression: although both species are sympatric identical haplotypes were found in this species pair not closer than at a distance of 580 km.

### Species assigned to multiple BINs

**Figure 4:**
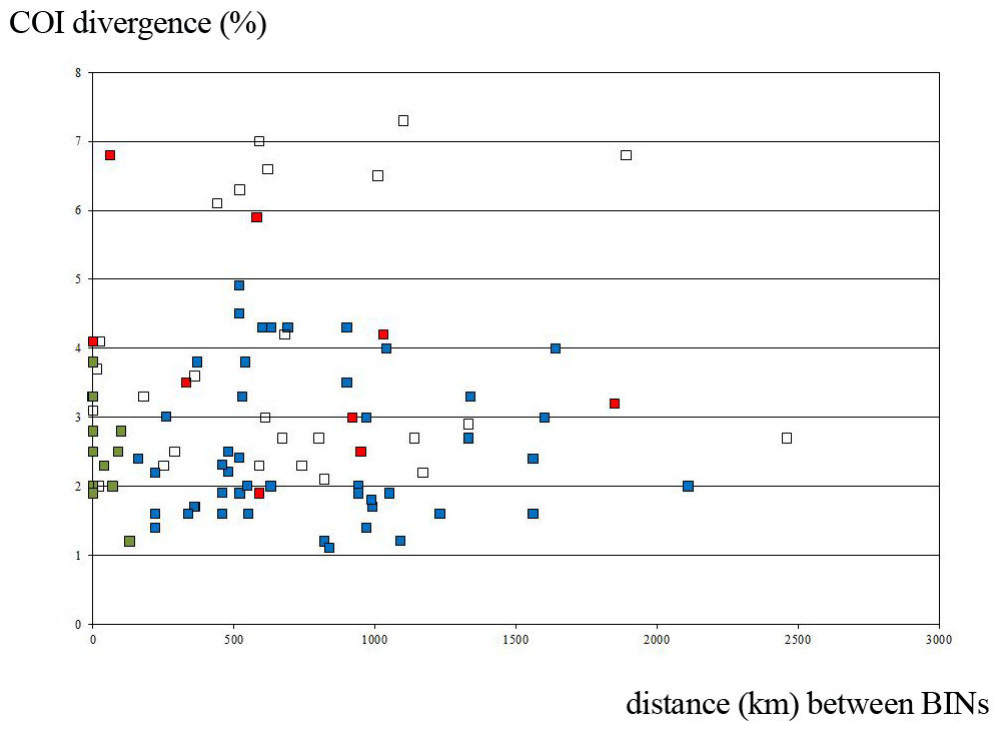
Intraspecific BIN splits: genetic distance versus shortest distance between records of both BINs. Genetic distance was measured by the minimum pairwise K2P distance (expressed as a percentage) and plotted against the shortest distance (in km) between the capture location of specimens from the two BINs. In species with multiple BIN-splits all possible combinations were included producing a total of 93 comparisons. Blue and green dots: BIN clusters with more than one specimen in Europe – green: sympatric – blue: allopatric – unfilled: one of the BINs based on one singleton; cf. exact tables in Appendix S1 and Appendix S4 – red dots: as in unfilled dots one of the BINs based on a singleton in Europe but with additional specimens outside Europe.

Most European geometrids show very limited intraspecific barcode variation, but 38 of our 215 traditional species (see Appendix S1) were placed in two or more BINs, typically with more than 1.5% sequence divergence. Of the 98 BINs these represent, about one seventh (15) involved a single European specimen distant from the cluster formed by its conspecifics (cf. Appendix S1; Appendix S4 and unfilled dots in Figure 4). Interestingly, nine of these 15 singletons involved a haplotype also detected in specimens collected outside Europe. In 33 of the 38 species with multiple BINs, two or more BIN were represented by multiple specimens, and nine of these cases involved taxa with more than 4% divergence.

Among the 38 traditional species assigned to more than one BIN, 17 of the 93 intraspecific BIN combinations (18%) represent cases where distinct BINs occur in sympatry (here defined as instances where the minimum geographic distance between members of the two BINs was less than 100 km). No morphological differences (wing colour, wing pattern, morphology of genitalia) are apparent between members of different BINs in any of these cases.

In the other 76 cases belonging to 31 species, the BINs represent geographically isolated lineages with the minimum geographic distance between members of the BINs exceeding 100 km. In nine of these species, the BIN splits correspond to subspecies recognized by traditional taxonomy: *Pseudoterpna* c. *coronillaria* & P. *c. flamignii, Hemistola* c. *chrysoprasaria* & *H*. *c. occidentalis, Idaea* c. *consanguinaria* & *I. c. consecraria, Idaea* o. *ochrata* & *I*. *o. albida, Idaea* f. *fractilineata* & *I. f. subrufaria, Idaea* o. *obsoletaria* & *I. o. dierli & I. o. lilaceola, Scopula* *s.* submutata & *S. s. nivellearia, Scopula asellaria dentatolineata &* S. *a. romanaria, Rhodostrophia pudorata sicanaria & R. p. perezaria*. In five other species there are clear differences in morphology and/or wing pattern between species assigned to different BINs: *Aplasta ononaria* from the Iberian Peninsula*, I. elongaria* from southern Italy*, I. longaria* from Macedonia*, I. seriata* from both Sardinia and Sicily, and *S. confinaria* from northern and central Italy all show consistent differences from the more widespread typical forms [19,20]. 

Many of the splits (17 species and 45 BINs) involve Mediterranean species with apparently genetically distinct populations on different southern European peninsulas or islands. Several other species with Sub-Mediterranean or European distribution show BIN divergence in (parts of) the Mediterranean region. In two other species (8 BINs) the divergent populations occur in southern France and the Iberian Peninsula. 

## Discussion

### Identification accuracy

This study examined patterns of DNA barcode variation in 215 species of European geometrids, establishing that 93% of these species could be unambiguously identified on a continental scale. Moreover, success in identification often rose to 100% when collections were restricted to a single country, confirming the effectiveness of DNA barcoding for the identification of Lepidoptera species. This performance corresponds closely with results obtained in other regions. For example, 99% of Lepidoptera from northeastern America were found to possess diagnostic barcodes [23], while re-identification success was 98% for Costa Rican Lepidoptera [24] and 90% for Romanian butterflies (based on a restrictive monophyly approach) [25]. Future studies with larger sample sizes may reveal cases of haplotype sharing overlooked in the present study, but it seems unlikely that identification success will decline by more than 1-2%. 

### Correspondence between BINs and species

BOLD assigned the 183 European species examined in this study to 224 BINs (215 species and 253 BINs when including adjacent countries). Although the BIN and species counts only showed an 18% (15%) discrepancy, only 67% of traditional species boundaries corresponded perfectly with BINs. Cases of discrepancy involved both the assignment of specimens from a single species into two or more BINs and the amalgamation of two or more species into a single BIN. These cases of discrepancy are discussed in more detail in subsequent sections, but we emphasize that some of these cases reflect instances where the current taxonomic system is flawed. Viewed from this perspective, the BIN system is a useful heuristic for revealing species deserving more intensive study. 

### Different species assigned to the same BIN

Some of the pairs or triads of species assigned to a single BIN involved species that have already been challenged on morphological grounds such as *Pseudoterpna coronillaria* & *P. pruinata* [26] and *Idaea incisaria* & *I. albarracina* [20,27]. Similarly, a subspecific status was suggested for three other species pairs [20]: *Idaea humiliata* & *I. davidi*, *Idaea seriata* & *I. minuscularia*, and *Scopula scalercii* & *S. beckeraria*. Other cases of BIN sharing involve species groups whose discrimination is challenging and sometimes uncertain. For example *Chlorissa viridata* & *C. cloraria* [19] and *Rhodostrophia calabra* & *R. discopunctata* [20] each involve a pair of species with uncertain taxonomy, patchy distribution and specimens with intermediate character patterns in putative hybrid zones. This leaves only five pairs and one triad of species sharing BINs (two pairs and the triad shared identical barcodes) with very clear differences in morphology involving species whose delineation has not been questioned: *Boudinotiana notha* & *B. touranginii, Idaea aversata* & *I. gelbrechti, Scopula decolor* & *S. imitaria, Scopula frigidaria* & *S. ternata, Cyclophora albiocellaria* & *C. ariadne*, and the triad *Cyclophora quercimontaria, C. suppunctaria* & *C. punctaria* [19,20].

We suggest that cases of BIN sharing among allopatric, slightly divergent genetic clusters represent recently separated lineages that have recently speciated or that are still undergoing genetic differentiation. The evolution of morphological traits such as genitalia is generally thought to be rapid [28-30], perhaps faster than COI diversification. This may be due to natural selection for isolating mechanisms in parapatric populations leading to reproductive character displacement [31], though it is more likely to arise from rapid evolution due to sexual selection involving cryptic female choice and sexual conflict [32].

We suspect that two of the three sympatric species pairs sharing identical barcodes reflect cases where hybridization led to replacement of the original mitochondrial genome. This can occur when a cytoplasmic driving element such as the bacteria *Wolbachia* is involved [33]. The marked morphological differences between the species pairs *Boudinotiana notha* & *B. touranginii* and *Cyclophora quercimontaria* & *C. punctaria* do not suggest recent speciation with incomplete lineage sorting or restricted COI diversification. The case of *Scopula frigidaria* & *S. ternata* in Finland may have a similar explanation, but specimens with the same barcode were always allopatric by more than 580km. The species *Chlorissa viridata* & *C. cloraria* may represent a very recent speciation event whose slight, inconsistent morphological divergence is accompanied by a lack of differentiation at COI [16,19]. Incomplete lineage sorting and introgression may also occur together since young species are more likely to hybridize than old species.

Incomplete lineage sorting [10,34,35] may explain the complex patterns of barcode variation in the genus *Pseudoterpna* and in the *Scopula confinaria/S. alba* complex. The close morphological similarity between taxa in these groups further supports their recent separation. In such cases, taxonomic decisions are often very subjective and depend on the choice of species delimitation models, application of species concepts and taxonomic principles, especially when allopatric populations are involved [36].

Cases of allopatric species sharing identical barcode may also reflect mitochondrial replacement when they occur in species with strong morphological differences (e.g. *Scopula decolor* & *S. imitaria*). In other cases, when morphological differentiation is confined to a few traits (e.g. *Scopula scalercii* & *S. beckeraria*), it may just reflect recent, incipient speciation.

### One species assigned to two or more BINs

Our work has indicated that successful identification and the incidence of monophyly is very high in the studies at a national scale. However, when considered at a European scale, the success of molecular identification is reduced, reflecting the lower number of monophyletic species. A similar pattern was detected in DNA barcode studies on a group of closely related beetles in Europe [37] and on a group of European leaf-mining moths [38]. Interestingly, geographical differentiation played a minor role over distances of up to 2800 km in north-eastern American Lepidoptera [23] and in Central Asian butterflies [39]. Although the small sample sizes in the latter study (an average of less than three specimens per species) may also be a factor, the variable relationship between the performance of DNA barcodes in differentiating species and geographic coverage in different settings may reflect differing regional histories of population isolation and differentiation. 

We detected nine cases of species whose members were assigned to two or three BINs with divergence greater than 4% and in which one BIN contained only a single European specimen. However, in four of these cases, additional specimens of the ‘rare’ BIN were detected outside Europe (Figure 2), suggesting these singletons may reflect rare immigration from outside Europe. However, these rare BINs may also be an artefact of our limited sample size because many BINs in our data are just based on single records. Some of these rare sequences may also reflect pseudogenes although this is considered unlikely because they lacked typical features such as indels, stop codons or unusual amino acid substitutions. 

The existence of cryptic species overlooked by current taxonomy may explain many of the cases where allopatric populations of the same morphological species were assigned to different BINs. In fact, 14 of these species involve lineages with differences in external appearance already noted in the literature or that were apparent from our investigation. These correlations may justify the upgrading of some taxa to a subspecies or species rank (see results), though such decisions are inevitably subjective without further morphological and genetic study [36]. Interestingly, most of our cases of deep genetic divergence were found in the Mediterranean region and involved the large number of species of drought-adapted Sterrhinae in this area. Their high speciation rates appear to have occurred in an area whose terrain and history has led to frequent episodes of geographical isolation. 

About one fifth of the cases where a species was split into two or more BINs occurred in sympatry making them interesting candidates for further studies using additional genetic data including nuclear markers. Because no morphological differences were apparent between the lineages in these cases, intraspecific DNA polymorphisms may be involved. The presence of two barcode lineages showing substantial divergence might, in some of these cases, reflect the merger of populations that were isolated in different glacial refugia during the Pleistocene. Alternatively, *Wolbachia* infections may have driven novel mitochondrial lineages through their host population (for a detailed review of different mechanisms see [33]). 

Some cases of very deep divergence in sympatric or almost sympatric populations (see Appendix S1) need further integrative taxonomic study, for example in *Idaea elongaria* and *I. longaria*. Several similar BIN splits occurred on Sardinia (three cases; cf. Table 1) where repeated colonization waves (cf. [40]) by the same mainland species may have caused recurrent speciation as noted in some insular bird and beetle lineages [41,42]. 

### DNA barcoding and evolutionary research

Complex patterns of barcode variation such as those observed in *Pseudoterpna, Hemistola, Idaea seriata* and the *Scopula confinaria* species-group have often been termed as ‘failures of DNA barcoding’. However, they can also be viewed as providing interesting insights into evolutionary patterns, by revealing how population mergers and splits in different parts of a species’ distribution produce departures from monophyly in gene trees [43]. DNA barcode results also provide the information on sequence variation needed to apply coalescence-based modelling of mitochondrial DNA genealogies. As such, they enable detailed analyses of historical events, such as dating of lineage divergences [44]. For example, genetic distances in the genus *Cyclophora* are much smaller than in most other geometrid genera (interspecific distances not exceeding 3.2%),so many species share a BIN. Interestingly, intraspecific genetic variation in this genus is also generally very low as evidenced by the fact that each of the three widely distributed species (*C. puppillaria, C. porata*, *C. linearia*) shows a maximum variation of just one base pair. These results suggest very recent speciation events in this genus, an hypothesis supported by the frequent observation of hybrids in nature [20], and the ease with which they can be induced under artificial conditions. Thus, both incomplete lineage sorting and introgression may have contributed to the observed high frequency of BIN-sharing between species of *Cyclophora*.

DNA barcoding campaigns also provide opportunities for spin-off research by generating DNA extracts which can enable the sequencing of nuclear markers and reveal museum specimens which merit detailed morphological analyses in an integrative taxonomic/phylogenetic approach. 

Based upon the results of this study, we anticipate that broad species coverage and increasing sample sizes obtained through the International Barcode of Life program will play an increasingly powerful role in taxonomy and evolutionary research. The BIN system as implemented in the BOLD database will also help to focus future research on those taxa most in need of detailed study by revealing species sharing BINs and those split amongst multiple BINs.

## Supporting Information

Appendix S1 **List of species, BINs (URIs) and barcode gap analysis.** List of species, URIs, barcoded material, and barcode gap analysis (intraspecific variation and distance of nearest neighbor) for 249 European species in the subfamilies Archiearinae, Desmobathrinae, Orthostixinae, Geometrinae and Sterrhinae. BC = number of European Barcodes >500bp, AD = additional short sequences from Europe, OE = additional sequences from outside Europe. URIs (BINs) exclusively from material outside Europe are in brackets. Barcode Gap Analysis: Kimura 2 parameter, BOLD Aligner, >500bp, only European data included. In species with intraspecific divergences >2% (marked with asterisk) the different BINs were pooled in the first line, separate analysis in subsequent lines. Cases of barcode sharing are marked with red, while species with slight, but consistent divergences are marked with orange. Notes see at the end of the list. Country codes in accordance with the ISO 3166-1-alpha-2 code (=Top-Level-Domain-Codes), Sic = Sicily, Sar = Sardinia, Cor = Corsica, Pel = Peloponnese, Cre = Crete, countries are listed from north to south and from west to east.
(PDF)

Appendix S2 **GenBank Accession numbers.** List of specimen-IDs (from BOLD database), GenBank Accession numbers, and species name, for the European geometrid vouchers with barcodes.
(PDF)

Appendix S3 **List of European geometrid species without BIN.** List of 34 European taxa without a BIN assignment (awaiting DNA barcoding); four species with short sequences are marked with an asterisk. 30 species are completely missing (12%), whilst 88% species of the five examined European geometrid subfamilies are represented by COI sequences.
(PDF)

Appendix S4 **Species with BIN-Splits.** Intraspecific genetic divergences (in % minimum pairwise distance, Kimura 2 parameter) and shortest geographic distance (in km) between representatives of each BIN; n = number of European BIN-representatives (barcoded >500 bp), ‘1+’ refers to BINs with a singleton in Europe, but with additional representatives outside Europe; in species with multiple BIN-splits all possible combinations were included producing a total of 93 comparisons.
(PDF)

Appendix S5 **Neighbor Joining Tree.** Neighbor Joining Tree (BOLD-Aligner, Kimura 2 parameter) for the 1610 European specimens (barcoded >500 bp), belonging to 183 species and 224 BINs.
(PDF)
