# Correction: Genetic Patterns in European Geometrid Moths Revealed by the Barcode Index Number (BIN) System

**DOI:** 10.1371/annotation/8c15cfdf-7bfe-4da1-a8c8-76d5d465f7b2

**Published:** 2013-12-31

**Authors:** Axel Hausmann, H. Charles J. Godfray, Peter Huemer, Marko Mutanen, Rodolphe Rougerie, Erik J. van Nieukerken, Sujeevan Ratnasingham, Paul D. N. Hebert

Affiliation number 7 for the fifth author, Rodolphe Rougerie, is incorrect. The correct affiliation is: INRA, UR633 Unité de Recherche en Zoologie Forestière, Orléans, France.

