# Supplementary material for: Genetic Patterns in European Geometrid Moths Revealed by the Barcode Index Number (BIN) System
Source: PLoS One. 2013 Dec 17;8(12):e84518. doi: 10.1371/journal.pone.0084518 (PMC3866169; doi:10.1371/journal.pone.0084518)
Supplement: Appendix S1 — List of species, BINs (URIs) and barcode gap analysis. List of species, URIs, barcoded material, and barcode gap analysis (intraspecific variation and distance of nearest neighbor) for 249 European species in the subfamilies Archiearinae, Desmobathrinae, Orthostixinae, Geometrinae and Sterrhinae. BC = number of European Barcodes >500bp, AD = additional short sequences from Europe, OE = additional sequences from outside Europe. URIs (BINs) exclusively from material outside Europe are in brackets. Barcode Gap Analysis: Kimura 2 parameter, BOLD Aligner, >500bp, only European data included. In species with intraspecific divergences >2% (marked with asterisk) the different BINs were pooled in the first line, separate analysis in subsequent lines. Cases of barcode sharing are marked with red, while species with slight, but consistent divergences are marked with orange. Notes see at the end of the list. Country codes in accordance with the ISO 3166-1-alpha-2 code (=Top-Level-Domain-Codes), Sic = Sicily, Sar = Sardinia, Cor = Corsica, Pel = Peloponnese, Cre = Crete, countries are listed from north to south and from west to east. (PDF) [file pone.0084518.s001.pdf]

Appendix S1: List of species, BINs (URIs) and barcode gap analysis

List of species, URIs, barcoded material, and barcode gap analysis (intraspecific variation and distance of nearest neighbor) for249 European species in the subfamilies Archiearinae, Desmobathrinae, Orthostixinae, Geometrinae and Sterrhinae. BC = number of European Barcodes >500bp, AD = additional short sequences from Europe, OE = additional sequences from outside Europe. URIs (BINs) exclusively from material outside Europe are in brackets. Barcode Gap Analysis: Kimura 2 parameter, BOLD Aligner, >500bp, only European data included. In species with intraspecific divergences >2% (marked with asterisk) the different BINs were pooled in the first line, separate analysis in subsequent lines. Cases of barcode sharing are marked with red, while species with slight, but consistentdivergences are marked with orange. Notes see at the end of the list. Country codes in accordance with the ISO 3166-1-alpha-2 code (=Top-Level-Domain-Codes), Sic = Sicily, Sar = Sardinia, Cor = Corsica, Pel = Peloponnese, Cre = Crete, countries are listed from north to south and from west to east.

	BIN (URI)	Countries in EU	BC	AD	OE	Var mean	Var max	Nearest Neighbor in EU	Distance to NN (%)	Notes
**Archiearinae**										
Archiearis parthenias (1-2)	pooled	FI SW FR DE	12	2	0	0.62	1.33	Boudinotiana notha	6.7	(1)
* A. parthenias (1)	BOLD:ACE9095	FI SW FR DE	7	2	0	0.05	0.16	* A. parthenias (2)	1.2	
* A. parthenias (2)	BOLD:AAC5368	FR	5	0	0	0.11	0.33	* A. parthenias (1)	1.2	
Archiearis parthenias	BOLD:AAC5368	FI SW FR DE	12	2	0	0.62	1.33	Boudinotiana notha	6.7	(2)
Boudinotiana notha	BOLD:AAD8337	FI RU FR DE AT IT	13	3	0	0.09	0.17	Boudinotiana touranginii	0.0	
Boudinotiana touranginii	BOLD:AAD8337	FR	8	0	0	0.09	0.17	Boudinotiana notha	0.0	(3)
Boudinotiana puella	BOLD:AAD8337	SK	1	1	0	-	-	Boudinotiana notha	1.5	
Leucobrephos middendorfii	-									
**Desmobathrinae**										
Myinodes interpunctaria	BOLD:AAX3251	ES IT	1	1	0	-	-	Myinodes shohami	6.2	
Myinodes constantina	BOLD:AAG5649	ES	2	0	0	0.16	0.16	Myinodes interpunctaria	8.2	
Myinodes shohami	[BOLD:AAG5612]	[IL]	0	0	3	-	-	Myinodes interpunctaria	6.2	
Gypsochroa renitidata	BOLD:AAY2476	FR	1	0	1	-	-	Ennomos quercinaria	7.8	(4)
**Orthostixinae**										
Orthostixis cribraria (1-2)	pooled	IT GR	3	0	6	2.01	3.01	Ectropis crepuscularia	8.2	
* O. cribraria (1)	BOLD:AAW9670	GR	1	0	3	-	-	* O. cribraria (2)	3.0	
* O. cribraria (2)	BOLD:ABZ6151	IT	2	0	3	0.00	0.00	* O. cribraria (1)	3.0	
**Geometrinae**										
Heliothea discoidaria	-	ES	0	1	0	-	-			
Aplasta ononaria (1-2)	pooled	FR ES IT	4	1	3	2.48	3.80	Phaiogramma etruscaria	11.0	

---

[1] closer neighbor outside Europe: *Archiearis infans* from North America (2.6%)
[2] closer neighbor outside Europe: *Archiearis infans* from North America (2.6%)
[3] data provided by the courtesy of Antoine Lévêque, a related publication is in preparation (Lévêque, pers. comm.).
[4] closer neighbor outside Europe: *Derambila strigicosta* from New Guinee (6.5%), confirming relationship between *Derambila* and *Gypsochroa* as suggested in Hausmann (2001)

	BIN (URI)	Countries in EU	BC	AD	OE	Var mean	Var max	Nearest Neighbor in EU	Distance to NN (%)	Notes
* A. ononaria (1)	BOLD:AAE7297	IT	2	0	3	0.31	0.31	* A. ononaria (2)	3.8	
* A. ononaria (2)	BOLD:AAZ3877	FR ES	2	1	0	0.00	0.00	* A. ononaria (1)	3.8	
Holoterpna pruinosata	[BOLD:AAF0567]	[IL]	0	0	3	-	-	Pingasa lahayei	7.2	([5])
Pingasa lahayei	[BOLD:AAC6714]	[TN, IR, AE, YE, OM etc.]	0	0	23	-	-	Pseudoterpna coronillaria	7.0	([6])
Pseudoterpna pruinata (1-2)	pooled	DE IT HR GR	11	0	1	1.64	4.63	Pseudoterpna coronillaria	0.0	
* P. pruinata (1)	BOLD:ACE9846	GB DE IT HR GR	10	0	1	1.06	1.87	* P. coronillaria (2)	0.5	([7])
* P. pruinata (2)	BOLD:AAB9888	GR	1	0	0	-	-	* P. coronillaria (3)	0.0	([7])
Pseudoterpna coronillaria (1-4)	pooled	ES FR IT	27	3	12	2.23	4.03	Pseudoterpna pruinata	0.0	([8])
* P. c. flamignii (1)	BOLD:AAB9887	IT (Sic)	7	0	0	0.42	1.46	* P. c. coronillaria (2)	2.4	
* P. c. coronillaria (2)	BOLD:AAB9886	ES FR	5	0	0	0.19	0.46	* P. c. flamignii (3)	1.4	
* P. c. flamignii (3)	BOLD:ACE9846	IT	11	2	3	0.56	1.17	* P. pruinata (1)	0.5	
* P. c. coronillaria (4)	BOLD:AAB9888	IT (Sar)	4	1	2	0.00	0.00	* P. pruinata (2)	0.0	([9])
Pseudoterpna corsicaria	BOLD:AAL9228	IT (Sar)	2	0	0	0.31	0.31	Pseudoterpna coronillaria (2)	4.8	
Geometra papilionaria	BOLD:AAB2012	FI GB NL FR DE IT HR	18	0	1	0.89	1.87	Eucrostes indigenata	10.0	
Comibaena bajularia	BOLD:AAC3677	FI GB NL FR DE HU IT	15	4	7	0.21	0.62	Proteuchloris neriaria	10.6	
Comibaena pseudoneriaria	-									
Proteuchloris neriaria	BOLD:AAD4805	GR	2	0	11	0.00	0.00	Thetidia smaragdaria	10.0	
Thetidia plusiaria	-	ES	0	1	0	-	-			
Thetidia correspondens	[BOLD:AAW6757]	[KG]	0	0	1	-	-	Thetidia smaragdaria	3.0	([10])
Thetidia smaragdaria	BOLD:AAB5663	FI DE HU ES IT HR GR	17	0	3	0.38	1.26	Thetidia sardinica	0.2	([11])
Thetidia sardinica	BOLD:AAB5663	IT (Sar)	1	0	0	-	-	Thetidia smaragdaria	0.2	
Hemistola chrysoprasaria (1-2)	pooled	GB FR DE AT ES IT HR	25	1	1	0.96	3.69	Hemistola siciliana	1.7	
* H. c. chrysoprasaria (1)	BOLD:AAC0672	GB FR DE AT IT HR	23	1	0	0.58	1.71	* Hemistola siciliana	1.7	([12])
* H. c. occidentalis (2)	BOLD:AAI6787	ES	2	0	1	0.00	0.00	* H. c. chrysoprasaria (1)	2.8	([13])
Hemistola siciliana	BOLD:ABY6460	IT (Sic)	1	0	0	-	-	Hemistola chrysoprasaria	1.7	
Xenochlorodes olympiaria (1-3)	pooled	ES IT HR	6	0	3	2.67	4.78	Hemistola chrysoprasaria (occ.)	7.9	
* X. olympiaria (1)	BOLD:AAU2210	ES IT (Sar)	2	0	0	0.15	0.15	* X. olympiaria (2)	3.3	
* X. olympiaria (2)	BOLD:AAE4516	IT (south)	2	0	3	0.00	0.00	* X. olympiaria (3)	1.7	
* X. olympiaria (3)	BOLD:ABY8630	HR	2	0	0	0.31	0.31	* X. olympiaria (2)	1.7	
Jodis lactearia	BOLD:AAD4811	FI GB FR DE PT IT	17	0	1	0.08	0.31	Jodis putata	3.2	
Jodis putata (1-2)	pooled	FI SW DE	8	1	0	1.12	2.57	Jodis lactearia	3.2	
* J. putata (1)	BOLD:ABZ4040	FI SW DE	6	1	0	0.16	0.48	* J. putata (2)	2.0	
* J. putata (2)	BOLD:AAF3148	DE	2	0	0	0.00	0.00	* J. putata (1)	2.0	

[5] closer neighbor outside Europe: Asian *Holoterpna diagrapharia* (3.8%; short sequence)
[6] an additional BIN in South Africa, outside Europe closest distance to *Holoterpna diagrapharia* (4.5%; short sequence), raising questions about generic concept
[7] divergence between the two intraspecific European BINs 4.1%, possibly due to incomplete lineage sorting
[8] two other BINs from outside Europe
[9] genetically identical with North African populations
[10] closer neighbour outside Europe: *Thetidia crucigerata* from Middle East (2.6%)
[11] another BIN from outside Europe
[12] in central and southern Italy with several regional genetic lineages, morphologically sometimes intermediate towards *H. siciliana*; these populations were previously drawn to *H. siciliana* but molecular data suggest conspecifity with *H. chrysoprasaria*
[13] divergence between the two intraspecific European BINs raising the question about a possible species status for the taxon *occidentalis*; BIN-sharing with populations from North Africa

	BIN (URI)	Countries in EU	BC	AD	OE	Var mean	Var max	Nearest Neighbor in EU	Distance to NN (%)	Notes
Eucrostes indigenata (1-2)	pooled	IT BG GR	9	1	3	1.04	4.45	Hemistola siciliana	6.8	([14])
* E. indigenata (1)	BOLD:AAC6469	IT BG GR	8	1	3	0.47	1.14	* E. indigenata (2)	3.7	
* E. indigenata (2)	BOLD:AAC6471	IT (Sar)	1	0	0	-	-	* E. indigenata (1)	3.7	
Thalera fimbrialis (1-2)	pooled	FI FR DE ES IT GR	16	0	4	1.31	3.64	Eucrostes indigenata	8.0	([15,16])
* T. fimbrialis (1)	BOLD:AAC6840	FI FR DE ES IT GR	15	0	4	1.02	1.87	* T. fimbrialis (2)	3.5	
* T. fimbrialis (2)	BOLD:AAC6841	FI	1	0	0	-	-	* T. fimbrialis (1)	3.5	
Dyschloropsis impararia	[BOLD:AAI9886]	[central RU]	0	0	1	-	-	Hemithea aestivaria	6.9	([17])
Bustilloxia saturata	-									
Kuchleria insignata	BOLD:AAJ3108	ES	4	1	0	0.53	0.85	Kuchleria menadiara	2.2	([18])
Kuchleria menadiara	[BOLD:ABZ0774]	[MA]	0	0	1	-	-	Kuchleria insignata	2.2	([18])
Hemithea aestivaria (1-2)	pooled	FI GB NL FR DE IT HR	28	0	1	0.47	2.98	Phaiogramma etruscaria	6.7	
* H. aestivaria (1)	BOLD:AAA4522	FI GB NL FR DE IT HR	25	0	1	0.30	1.24	* H. aestivaria (2)	2.5	
* H. aestivaria (2)	BOLD:AAA4524	GB NL	3	0	0	0.00	0.00	* H. aestivaria (1)	2.5	
Chlorissa viridata	BOLD:ABY5630	FI DE IT	5	0	4	0.34	0.61	Chlorissa cloraria	0.0	
Chlorissa cloraria	BOLD:ABY5630	DE AT IT HR GR	8	0	0	0.00	0.00	Chlorissa viridata	0.0	
Chlorissa pretiosaria	[BOLD:AAJ4437]	[IR]	0	0	1	-	-	Chlorissa viridata	6.9	([19])
Phaiogramma etruscaria	BOLD:AAB4914	ES IT HR BG	12	3	9	0.41	1.55	Phaiogramma faustinata	2.0	
Phaiogramma faustinata	BOLD:AAB4914	ES GR	2	0	34	0.61	0.61	Phaiogramma etruscaria	2.0	
Microloxia herbaria (1-2)	pooled	IT	11	0	4	0.68	2.03	Phaiogramma faustinata	7.2	([20])
* M. herbaria (1)	BOLD:AAB9356	IT (Sic, south)	9	0	4	0.17	0.46	* M. herbaria (2)	1.9	
* M. herbaria (2)	BOLD:ACE8779	IT (Sar)	2	0	0	0.00	0.00	* M. herbaria (1)	1.9	
**Sterrhinae**										
Anthometra plumularia	-									
Emmiltis pygmaearia	BOLD:AAM0343	IT	2	0	0	0.93	0.93	Idaea sericeata	8.1	
Cleta ramosaria	-									
Cleta perpusillaria	-									
Cleta filacearia	BOLD:AAA8395	IT	2	2	3	1.55	1.55	Idaea ochrata	6.4	([21])
Idaea serpentata	BOLD:AAA9647	FI DE AT HR MK	10	0	0	0.34	0.93	Idaea ochrata	6.9	
Idaea luteolaria	-									
Idaea aureolaria	BOLD:AAE6012	DE AT IT	4	0	3	0.71	1.12	Idaea leipnitzi	5.1	
Idaea flaveolaria	BOLD:AAF3049	IT	2	0	0	1.00	1.00	Idaea ochrata	4.9	
Idaea muricata	BOLD:AAD4604	FI GB FR DE IT ES	12	4	1	0.03	0.15	Idaea ochrata	5.1	
Idaea determinata	BOLD:AAC9955	IT BG GR	5	1	4	1.12	2.03	Idaea sardoniata	3.7	
Idaea litigiosaria	BOLD:AAK4264	ES	2	0	1	0.62	0.62	Idaea mutilata	3.5	

[14] closer neighbors outside Europe: *Eucrostes pygmaea* from Sokotra and *E. disparata* from Africa (3.9% and 4.3%)
[15] closer neighbor outside Europe: *Thalera chlorosaria* from Far East Asia (3.3%)
[16] another BIN from outside Europe
[17] closer neighbor outside Europe: *Thalera lacerataria* from Far East Asia (6.7%)
[18] *Kuchleria menadiara atlagenes* examined from Morocco, but conspecifity with nominotypical populations from Algeria and populations from Sicily unclear
[19] species identity awaiting confirmation by dissection and barcoding of types
[20] closer neighbor outside Europe: *Microloxia ruficornis* from North Africa and Cape Verde (3.6%)
[21] another BIN from outside Europe

	BIN (URI)	Countries in EU	BC	AD	OE	**Var** mean	**Var** max	Nearest Neighbor in EU	Distance to NN (%)	Notes
Idaea mutilata	BOLD:ABA0088	IT	1	0	0	-	-	Idaea litigiosaria	3.5	
Idaea lusohispanica	-									
Idaea sardoniata	BOLD:AAW9509	ES	1	1	0	-	-	Idaea determinata	3.7	
Idaea korbi	BOLD:AAY8514	ES	1	1	0	-	-	Idaea litigiosaria	4.8	
Idaea mediaria	BOLD:AAF3090	ES	3	0	0	0.51	0.77	Idaea leipnitzi	3.5	
Idaea leipnitzi	BOLD:AAE5601	IT HR	5	0	0	0.06	0.15	Idaea mediaria	3.5	
Idaea rufaria	BOLD:AAF3007	DE IT GR	3	0	5	1.09	1.81	Idaea admiranda	4.4	([22,23])
Idaea consanguiberica	-									
Idaea admiranda	[BOLD:AAW9618]	[TR]	0	0	1	-	-	Idaea rufaria	4.4	
Idaea consanguinaria (1-2)	pooled	IT BG GR	7	0	3	0.83	2.84	Idaea sardoniata	4.6	
* I. c. consanguinaria (1)	BOLD:AAC3148	IT GR	6	0	0	0.30	0.77	* I. c. consecraria (2)	1.9	
* I. c. consecraria (2)	BOLD:ABY5966	BG	1	0	3	-	-	* I. c. consanguinaria (1)	1.9	([24])
Idaea ossiculata	BOLD:AAD1124	GR	1	0	4	-	-	Idaea leipnitzi	5.2	([25])
Idaea sericeata	BOLD:ACE5960	ES IT	1	1	4	-	-	Idaea sardoniata	4.3	([26])
Idaea macilentaria	-	ES	0	1	0	-	-			
Idaea ochrata (1-2)	pooled	NL FR ES IT HR BG GR	16	0	9	0.85	2.33	Idaea leipnitzi	3.9	
* I. o. ochrata (1)	BOLD:AAC1604	NL FR IT HR BG GR	13	0	7	0.31	0.77	* I. o. albida (2)	1.6	
* I. o. albida (2)	BOLD:ACE7590	ES	3	0	2	0.00	0.00	* I. o. ochrata (1)	1.6	([27])
Idaea nevadata	-									
Idaea figuraria	[BOLD:AAL9520]	[MA]	0	0	2	-	-	Idaea intermedia	7.2	
Idaea alicantaria	BOLD:AAF2897	ES	4	0	0	1.02	1.84	Idaea ochrata	5.3	
Idaea nigrolineata	[BOLD:AAW9510]	[TN]	0	0	1	-	-	Idaea ochrata	6.1	
Idaea completa	[BOLD:AAW9710]	[MA, TN]	0	0	2	-	-	Idaea leipnitzi	5.7	([28])
Idaea intermedia	BOLD:AAE2807	GR	2	0	4	0.00	0.00	Idaea inquinata	5.3	([29,30])
Idaea rusticata	BOLD:AAB3980	GB FR IT HR	23	2	3	0.65	1.40	Idaea mustelata	4.2	
Idaea mustelata	BOLD:AAW9512	ES	2	0	0	0.16	0.16	Idaea rusticata	4.2	
Idaea filicata	BOLD:AAB5540	IT HR GR	15	0	7	0.88	2.03	Idaea troglodytaria	3.4	([31])
Idaea troglodytaria	BOLD:AAD8334	GR	1	0	6	-	-	Idaea filicata	3.4	([31])
Idaea laevigata	BOLD:AAW9598	ES IT	2	1	0	0.80	0.80	Idaea inquinata	4.4	
Idaea efflorata	BOLD:AAC6470	PT ES IT	2	3	0	1.32	1.32	Idaea politaria	3.9	
Idaea attenuaria	BOLD:AAI7625	ES IT	5	1	0	0.25	0.62	Idaea incalcarata	6.5	([32])
Idaea incalcarata	BOLD:AAK4271	ES	1	0	0	-	-	Idaea attenuaria	6.5	

[22] nearest neighbor inferred from data outside Europe; nearest neigbor with European data: *Idaea ochrata* (5.3%)
[23] another BIN from outside Europe
[24] BIN-sharing with subspecies *consecraria* from the Middle East
[25] closer neighbor outside Europe: *Idaea renataria* from Morocco (3.4%)
[26] another BIN from outside Europe
[27] BIN-sharing with populations from North Africa
[28] populations from Morocco and Tunisia BIN-sharing but diverging by 2.14%, indicating possible cryptic diversity at species or subspecies rank, for different habitus see Hausmann (2004)
[29] closer neighbor outside Europe: *Idaea proclivata* from Turkey (4.9%)
[30] another BIN from outside Europe
[31] distance between *Idaea filicata* and its sister species *I. troglodytaria* decreases to 2.6% when data from outside Europe are included
[32] closer neighbor outside Europe: *Idaea volloni* from Tunisia and Israel (6.0%)

	BIN (URI)	Countries in EU	BC	AD	OE	Var mean	Var max	Nearest Neighbor in EU	Distance to NN (%)	Notes
Idaea typicata	BOLD:AAF3045	FR IT	6	0	0	0.57	1.40	Idaea alyssumata	2.6	
Idaea alyssumata (1-6)	pooled	FR PT ES	19	2	0	2.84	4.93	Idaea urcitana	2.2	
* I. alyssumata (1)	BOLD:AAC5078	ES (south, Malaga)	5	0	0	0.52	0.92	* I. urcitana	2.2	
* I. alyssumata (2)	BOLD:AAL5780	PT	6	0	0	0.21	0.47	* I. alyssumata (5)	3.5	([33])
* I. alyssumata (3)	BOLD:ABZ1486	ES (north-east)	2	0	0	1.02	1.02	* I. alyssumata (4)	1.4	
* I. alyssumata (4)	BOLD:ACE8988	FR (Pyrenees)	2	0	0	0.33	0.33	* I. alyssumata (3)	1.4	
* I. alyssumata (5)	BOLD:ABZ1737	FR	3	2	0	0.00	0.00	* I. alyssumata (1)	1.9	
* I. alyssumata (6)	BOLD:ABZ1739	ES (south, Granada)	1	0	0	-	-	* I. alyssumata (5)	2.3	
Idaea urcitana	BOLD:ABY9944	ES	5	0	0	0.10	0.17	Idaea alyssumata (1,3)	2.2	
Idaea moniliata	BOLD:AAC7660	DE IT	7	1	6	0.44	0.77	Idaea leipnitzi	6.2	
Idaea spissilimbaria	-									
Idaea circuitaria	BOLD:AAF3086	GR	1	0	0	-	-	Idaea rainerii	4.1	([34])
Idaea rainerii	BOLD:AAP4080	IT (Sar)	1	1	0	-	-	Idaea circuitaria	4.1	
Idaea albarracina	BOLD:AAK4223	ES (east)	3	0	0	0.56	0.85	Idaea incisaria	0.2	([35])
Idaea incisaria	BOLD:AAK4223	PT (south)	1	0	0	-	-	Idaea albarracina	0.2	([35])
Idaea textaria	BOLD:AAD4546	GR (Samos)	3	0	5	0.00	0.00	Idaea obliquaria	4.1	([36])
Idaea calunetaria (1-2)	pooled	FR ES	3	2	0	2.81	4.20	Idaea albitorquata	4.5	
* I. calunetaria (1)	BOLD:AAO3782	FR ES	2	2	0	0.62	0.62	* I. calunetaria (2)	3.6	
* I. calunetaria (2)	BOLD:AAZ2849	ES (south)	1	0	0	-	-	* I. calunetaria (1)	3.6	
Idaea belemiata	BOLD:AAI7608	PT, ES	3	0	2	1.45	1.87	Idaea fractilineata	5.6	
Idaea elongaria (1-4)	pooled	ES IT HR GR	10	2	4	2.10	8.14	Idaea palaestinensis	5.3	
* I. elongaria (1)	BOLD:AAD0040	ES IT HR	7	0	0	0.93	2.00	* I. elongaria (2)	2.5	([37])
* I. elongaria (2)	BOLD:AAP2306	ES (south, Malaga)	1	0	0	-	-	* I. elongaria (1)	2.5	
* I. elongaria (3)	BOLD:AAY5007	GR	1	0	0	-	-	* I. elongaria (2)	2.7	
* I. elongaria (4)	BOLD:AAA8985	IT (Calabria)	1	2	5	-	-	* I. palaestinensis	5.3	([38,39])
Idaea palaestinensis	BOLD:AAC3201	GR	1	0	13	-	-	Idaea elongaria	5.3	([40])
Idaea obsoletaria (1-4)	pooled	ES IT GR	22	2	5	1.81	4.54	Idaea infirmaria	5.4	([41])
* I. obsoletaria (1)	BOLD:AAB4938	IT GR	9	2	2	0.22	0.62	* I. o. lilaceola (2)	1.2	
* I. o. lilaceola (2)	BOLD:ACE6095	ES	7	0	0	0.33	0.84	* I. obsoletaria (1)	1.2	
* I. o. lilaceola (3)	BOLD:ABY7684	ES	1	0	0	-	-	* I. obsoletaria (1)	2.1	
* I. o. dierli (4)	BOLD:AAB4939	IT (Sic)	5	0	0	0.00	0.00	* I. obsoletaria (1)	2.2	
Idaea obliquaria	BOLD:AAL9519	FR (Cor)	3	0	0	0.10	0.15	Idaea albarracina	2.5	
Idaea inquinata	BOLD:AAB5664	DE ES IT	14	0	5	1.15	2.18	Idaea laevigata	4.4	([42,43])

[33] data provided by the courtesy of Antoine Lévêque, a related publication is in press (Lévêque, pers. comm.), during the editorial process, a new species was described for this cluster: *Idaea barbuti* Tautel & Lévêque, 2013 (Alexanor, 25 (6), 2012 (2013) : 361-384)
[34] closer neighbor outside Europe: *Idaea mimosaria* from Turkey (1.9%), BIN-sharing but morphologically distinct
[35] close genetic distance supporting downgrading of *albarracina* to subspecies of *Idaea incisaria* (cf. Hausmann 2004)
[36] closer neighbor outside Europe: sister species *Idaea laszloi* from Turkmeniya and north-eastern Iran (0.8%), with differences in genitalia
[37] in Italy barcoded from Sicily, Sardinia and Tuscany; in Spain barcoded from Andalusia and the Balearics
[38] corresponding to the haplotype of the Middle East populations
[39] closest intraspecific distance to *I. elongaria* (4): 6.1%
[40] closer neighbor outside Europe: Iranian sister species *Idaea persidis* (2.9%)
[41] closer neighbor outside Europe: sister species *Idaea epaphrodita* from Israel (3.8%)

	BIN (URI)	Countries in EU	BC	AD	OE	Var mean	Var max	Nearest Neighbor in EU	Distance to NN (%)	Notes
Idaea blaesii	BOLD:AAF2873	ES	4	0	0	0.00	0.00	Idaea inquinata	6.3	
Idaea dilutaria	BOLD:AAD0840	DE IT GR	10	0	0	0.29	0.70	Idaea humiliata	3.5	
Idaea fuscovenosa (1-4)	pooled	GB FR DE IT	21	2	3	1.90	3.31	Idaea humiliata	3.7	([44])
* I. fuscovenosa (1)	BOLD:ABY4552	GB FR DE	13	1	0	0.40	1.24	* I. fuscovenosa (2)	2.7	
* I. fuscovenosa (2)	BOLD:AAB7362	GB DE	5	0	2	0.07	0.17	* I. fuscovenosa (3)	1.7	
* I. fuscovenosa (3)	BOLD:ACF2682	IT (south)	2	1	0	1.08	1.08	* I. fuscovenosa (2)	1.7	
* I. fuscovenosa (4)	BOLD:ABY5916	IT (Sic)	1	0	0	-	-	* I. fuscovenosa (3)	2.0	
Idaea robiginata	-									
Idaea lutulentaria	-									
Idaea humiliata (1-2)	pooled	FI NL FR DE CH IT GR	15	1	4	0.73	2.35	Idaea davidi	0.7	
* I. humiliata (1)	BOLD:ACF2945	FI NL FR DE CH IT	13	1	0	0.28	0.93	* I. davidi	0.7	
* I. humiliata (2)	BOLD:AAA8984	GR IT (south)	2	0	4	0.15	0.15	* I. davidi	1.7	([45])
Idaea davidi	BOLD:ACF2945	ES	1	0	0	-	-	Idaea humiliata	0.7	([46])
Idaea bigladiata	[BOLD:AAL9528]	ES [MA]	0	2	2	-	-	Idaea politaria	3.8	
Idaea politaria	BOLD:AAC4307	IT BG	7	0	5	0.29	0.62	Idaea humiliata	2.8	
Idaea longaria (1-4)	pooled	ES IT MK	11	1	11	2.39	7.88	Idaea calunetaria	7.2	([47,48])
* I. longaria (1)	BOLD:AAF3102	ES	4	0	0	0.05	0.32	* I. longaria (2)	1.6	
* I. longaria (2)	BOLD:ACE7056	IT (Sic)	4	0	0	0.00	0.00	* I. longaria (1)	1.6	
* I. longaria (3)	BOLD:AAY5147	IT (Sar)	1	1	0	-	-	* I. longaria (4)	6.5	
* I. longaria (4)	BOLD:ABY5104	MK	2	0	0	0.00	0.00	* I. longaria (1)	1.8	
Idaea nexata	-									
Idaea manicaria	[BOLD:AAW9525]	[TN]	0	0	1	-	-	Idaea distinctaria	7.0	
Idaea descitaria	-									
Idaea vesubiata	BOLD:AAM0983	FR	1	0	0	-	-	Idaea obliquaria	5.7	
Idaea libycata	BOLD:AAW9522	FR	1	0	0	-	-	Idaea politaria	6.2	([49])
Idaea consolidata	BOLD:AAW9715	IT	1	0	5	-	-	Idaea leipnitzi	6.1	([50,51])
Idaea joannisiata	BOLD:AAK4262	ES	3	0	0	0.31	0.46	Idaea consanguinaria	6.6	([51])
Idaea acutipennis	-									
Idaea seriata (1-4)	pooled	FI GB NL FR DE AT IT HR	41	4	2	1.84	4.13	Idaea minuscularia	0.2	
* I. seriata (1)	BOLD:ACF4900	FI GB NL FR AT	15	4	0	0.06	0.33	* I. seriata (2)	1.2	
* I. seriata (2)	BOLD:ABZ4137	IT (Sic)	6	0	0	0.10	0.31	* I. seriata (1)	1.2	
* I. seriata (3)	BOLD:ABY6334	IT (Sar)	4	0	0	0.16	0.33	* I. seriata (2)	2.0	([52])

[42] in Europe with three clusters at constant intraspecific distances of 1.3% and 1.4%, probably corresponding with West-, Central- and East-Mediterranean origins
[43] outside Europe BIN-sharing with *Idaea holliata* and *Idaea affinitata*, moreover partly exact barcode-sharing (introgression)
[44] another BIN from outside Europe
[45] *I. humiliata* (1) and *I. humiliata* (2) diverging by 1.9%
[46] close genetic distance supporting downgrading of *davidi* to subspecies of *Idaea humiliata*
[47] another BIN from outside Europe
[48] closer neighbor outside Europe: sister species *Idaea abnorma* from Canary islands (4.1%)
[49] closer neighbor outside Europe: *Idaea nigra* from Canary islands (5.9%)
[50] another BIN from outside Europe, at a distance of 5.9%, awaiting taxonomic revision
[51] genetic distance between the (morphologically) close allies *Idaea consolidata* and *I. joannisiata* 7.2%
[52] distance from the southern main cluster (4): 2.8%. Awaiting integrative taxonomic analysis.

	BIN (URI)	Countries in EU	BC	AD	OE	Var mean	Var max	Nearest Neighbor in EU	Distance to NN (%)	Notes
* I. seriata (4)	BOLD:AAA9645	FR (Cor) DE IT HR	16	0	2	0.08	0.48	* I. minuscularia	0.2	([53])
Idaea minuscularia	BOLD:AAA9645	ES	13	0	1	0.03	0.16	Idaea seriata	0.2	([54])
Idaea ibizaria	BOLD:AAW9671	ES	1	0	0	-	-	Idaea obliquaria	3.8	
Idaea albitorquata	BOLD:AAW9689	BG GR	3	0	1	0.38	0.61	Idaea obliquaria	3.4	
Idaea carvalhoi	BOLD:AAM0987	ES	1	0	0	-	-	Idaea infirmaria	5.5	
Idaea camparia	BOLD:AAB7027	IT (Sic) HR BG GR	10	0	14	0.52	2.35	Idaea textaria	6.9	([55])
Idaea subsericeata (1-2)	pooled	GB NL FR ES IT HR GR	34	2	5	0.57	2.08	Idaea obsoletaria	7.4	
* I. subsericeata (1)	BOLD:ACE4009	GB NL FR ES IT HR GR	28	2	5	0.08	0.46	* I. subsericeata (2)	1.7	
* I. subsericeata (2)	BOLD:ACE4010	IT (Sar)	6	0	0	0.19	0.46	* I. subsericeata (1)	1.7	
Idaea pallidata	BOLD:AAF3002	FI DE IT	6	2	0	0.27	0.52	Idaea ochrata	6.8	
Idaea mancipiata	[BOLD:ABU6270]	[TM]	0	0	1	-	-	Idaea politaria	6.4	
Idaea sylvestraria	BOLD:AAC3705	FI GB FR DE	14	1	0	0.22	0.49	Idaea muricata	5.2	
Idaea dimidiata	BOLD:AAA4213	FI GB FR NL DE IT	27	2	7	0.51	2.34	Idaea subsaturata	5.4	([56])
Idaea subsaturata	BOLD:AAZ7166	ES	1	0	0	-	-	Idaea dimidiata	5.4	
Idaea trigeminata (1-3)	pooled	GB IT GR	11	2	11	3.80	7.16	Idaea ochrata	9.1	([57,58])
* I. trigeminata (1)	BOLD:AAB6968	GB IT	9	2	0	0.40	0.89	* I. trigeminata (2)	5.9	
* I. trigeminata (2)	BOLD:ACE7419	GR (centre)	1	0	1	-	-	* I. trigeminata (3)	3.3	
* I. trigeminata (3)	BOLD:ABU8045	GR (Pel)	1	0	0	-	-	* I. trigeminata (2)	3.3	([59])
Idaea biselata	BOLD:AAB2239	FI GB NL FR DE AT IT	31	0	2	0.12	0.48	Idaea filicata	9.8	([60])
Idaea fractilineata (1-2)	pooled	PT ES IT	7	0	1	2.16	4.00	Idaea belemiata	5.6	([61])
* I. fractilineata (1)	BOLD:AAK4252	IT (Malta, Lampedusa)	3	0	1	0.51	0.77	* I. f. subrufaria (2)	3.3	
* I. fractilineata subrufaria (2)	BOLD:AAF3052	PT ES	4	0	0	0.00	0.00	* I. fractilineata (1)	3.3	
Idaea exilaria	BOLD:AAP4082	ES	1	0	0	-	-	Idaea distinctaria	7.1	
Idaea cervantaria	BOLD:AAC5077	ES	11	3	8	0.15	0.48	Idaea obliquaria	6.1	([62])
Idaea contiguaria	BOLD:AAP5642	DE PT IT	4	0	0	0.38	0.77	Idaea obliquaria	5.6	
Idaea rupicolaria	-									
Idaea deitanaria	BOLD:AAP4083	ES	1	0	0	-	-	Idaea humiliata	7.4	
Idaea saleri	-									
Idaea tineata	BOLD:AAE5589	GR	2	0	4	0.00	0.00	Idaea infirmaria	3.2	
Idaea infirmaria (1-3)	pooled	PT IT GR	15	0	1	1.99	3.48	Idaea tineata	3.2	
* I. infirmaria (1)	BOLD:AAC1723	IT (south + Sic)	7	0	0	0.41	0.92	* I. infirmaria (3)	2.2	
* I. infirmaria (2)	BOLD:AAC1724	GR IT (Sar)	5	0	0	0.06	0.15	* I. infirmaria (1)	2.5	([63])

---

[53] distance from the north-western main cluster (1): 3.0%. Awaiting integrative taxonomic analysis.

[54] *Idaea seriata* and *I. minuscularia* from Iberian peninsula separated by differences in male and female genitalia (Hausmann 2004). So far, no exact genetic match observed between both species, but some haplotypes very close to each other and differing by just one base pair of COI.

[55] large divergence of one Sicilian specimen (2.2%) supporting upgrading of the taxon *boeklini* from Sicily to subspecies rank (cf. Hausmann 2004).

[56] in Europe with two, partly sympatric clusters at a distance of 0.8%

[57] long distance from nearest neighbor supporting the hypothesis of an old evolutionary lineage for that taxon (cf. Hausmann 2004)

[58] five other BINs outside Europe

[59] distance from the European main cluster (1) 6.5%

[60] long distance from nearest neighbor supporting the hypothesis of an old evolutionary lineage for that taxon (cf. Hausmann 2004)

[61] closer neighbor outside Europe: sister species *Idaea inclinata* from the Levant (3.6%)

[62] in North Africa BIN-sharing, but with constant divergences and regional clusters; *Idaea okbaria* from Tunisia, BIN-sharing, too.

	BIN (URI)	Countries in EU	BC	AD	OE	Var mean	Var max	Nearest Neighbor in EU	Distance to NN (%)	Notes
* I. infirmaria (3)	BOLD:ABY5388	PT IT (Sar)	3	0	1	0.72	1.08	* I. infirmaria (1)	2.2	([63])
Idaea rhodogrammaria	BOLD:AAF3009	IT (Sar)	3	0	0	0.21	0.32	Idaea infirmaria	5.1	
Idaea lobaria	BOLD:ACD0706	ES	1	0	8	-	-	Idaea belemiata	5.8	([64])
Idaea ostrinaria (1-2)	pooled	ES IT GR	12	2	5	1.48	4.12	Idaea obliquaria	5.4	([65])
* I. ostrinaria (1)	BOLD:AAB2013	ES IT (south, centre, Sar)	10	2	0	0.46	1.87	* I. ostrinaria (2)	3.8	
* I. ostrinaria (2)	BOLD:AAE2645	IT (Sar) GR (Pel)	2	0	5	0.31	0.31	* I. ostrinaria (1)	3.8	([66])
Idaea metohiensis	BOLD:AAI7706	HR GR	2	0	1	0.92	0.92	Idaea ochrata	5.7	([67])
Idaea eugeniata	BOLD:AAE2790	PT ES	8	1	0	0.65	1.40	Idaea predotaria	5.1	
Idaea distinctaria	BOLD:AAB4028	IT HR	14	0	13	0.07	0.31	Idaea straminata	4.7	
Idaea predotaria	BOLD:AAK4150	ES	2	0	1	0.33	0.33	Idaea eugeniata	5.1	
Idaea nitidata	-	IT	0	1	0	-	-			
Idaea emarginata	BOLD:AAD0916	FI GB NL FR DE	9	2	0	0.17	0.33	Idaea simplicior	4.2	
Idaea dromikos	-									
Idaea simplicior	BOLD:AAL6582	ES	5	0	0	0.12	0.31	Idaea gelbrechti	4.1	
Idaea rubraria	BOLD:AAB4699	DE IT	11	1	0	0.06	0.32	Idaea deversaria	4.6	
Idaea aversata	BOLD:AAA8256	FI GB NL FR DE IT HR MK	46	1	4	0.21	0.98	Idaea gelbrechti	0.2	
Idaea gelbrechti	BOLD:AAA8256	ES	4	0	1	0.00	0.00	Idaea aversata	0.2	([68,69])
Idaea degeneraria	BOLD:AAB2099	FR PT ES IT HR	20	1	17	0.17	0.65	Idaea deversaria	4.7	
Idaea straminata	BOLD:AAA8258	FI GB DE AT ES IT	20	1	1	0.90	2.03	Idaea deversaria	4.3	
Idaea deversaria	BOLD:AAA9359	FI ES IT HR MK GR	15	0	11	0.63	2.83	Idaea gelbrechti	3.9	([70])
Idaea squalidaria	-									
Brachyglossina hispanaria	BOLD:AAP4045	ES	2	0	0	0.65	0.65	Idaea exilaria	5.7	([71])
Limeria macraria	-									
Oar reaumuraria	BOLD:AAI3423	ES	2	0	0	0.00	0.00	Scopula immistaria	8.9	([72])
Oar pratana	[BOLD:AAI3418]	[IL EG]	0	0	6	-	-	Scopula immistaria	8.6	([72,73])
Cinglis humifusaria	[BOLD:AAP2453]	[TR]	0	0	6	-	-	Cinglis andalusiaria	2.7	([74])
Cinglis andalusiaria	BOLD:AAY7856	ES	1	0	2	-	-	Cinglis humifusaria	2.7	([75])
Scopula immorata	BOLD:AAC9960	FI DE AT IT MK	14	0	0	0.37	1.17	Scopula tessellaria	2.3	
Scopula tessellaria	BOLD:ABZ2998	IT HR GR	6	1	0	0.57	1.08	Scopula immorata	2.3	
Scopula corrivalaria	BOLD:AAV8968	FI DE	2	0	0	0.77	0.77	Scopula caricaria	9.4	([76])

[63] the two clusters on Sardinia (2) and (3) at a distance of 3.5%
[64] actually under analysis, to be published in a separate paper (Ortiz pers. comm.); two further BINs from North Africa and from the Levant (locus typicus: south-eastern Algeria: Biskra)
[65] position in the NJ tree suggesting relationship with North African and Middle East *Brachyglossina* species, supported also by male genitalia (cf. Hausmann 2004)
[66] genetically very close to Turkish subsp. *demarginata*
[67] closely related populations in GE at a distance of 1.7%, BIN-sharing, probably belonging to *Idaea metohiensis*
[68] in genitalia clear differences between *Idaea aversata* and *Idaea gelbrechti*
[69] Spanish populations genetically diverging from those of Morocco by 0.46%
[70] distance between nominate subspecies and subsp. *fallax* from Iberian peninsula 0.95%; one specimen from southern Italy diverging by 2.1%, requiring further study
[71] position in the NJ tree and nearest neighbor suggesting relationship between *Idaea exilaria* and *Brachyglossina* species, supported also by morphologic traits (cf. Hausmann 2004); closer neighbor outside Europe: *Brachyglossina consociata* from Turkey (3.5%)
[72] distance to nearest neighbor inferred from a non-European barcode of the latter; distance between *Oar pratana* (specimen from Israel) and *O. reaumuraria*: 10.0%
[73] another BIN in YE
[74] three other BINs in JO, KZ KG and TM, awaiting integrative revision
[75] two other BINs in TN, awaiting integrative revision

	BIN (URI)	Countries in EU	BC	AD	OE	Var mean	Var max	Nearest Neighbor in EU	Distance to NN (%)	Notes
Scopula caricaria	BOLD:AAF0636	FI PL FR LI AT	7	0	0	0.22	0.31	Scopula emutaria	7.0	
Scopula nemoraria	BOLD:ABX2302	LV	1	0	0	-	-	Scopula incanata	8.2	
Scopula umbelaria	BOLD:AAE9751	DE AT	2	0	1	0.31	0.31	Scopula nigropunctata	5.2	
Scopula nigropunctata	BOLD:AAA9023	LV FR DE AT IT	13	0	4	0.20	0.66	Scopula umbelaria	5.2	
Scopula virgulata (1-2)	pooled	FI FR AT BA	5	1	8	1.25	2.98	Scopula alba	7.7	([77])
* S. virgulata (1)	BOLD:AAP7443	FI FR AT	4	1	8	0.15	0.38	* S. virgulata (2)	2.7	
* S. virgulata (2)	BOLD:AAD1137	BA	1	0	0	-	-	* S. virgulata (1)	2.7	
Scopula ornata (1-3)	pooled	FI DE AT ES IT HR MK GR	23	2	5	1.87	5.10	Scopula vigilata	7.5	
* S. ornata (1)	BOLD:AAB1550	FI DE AT IT HR MK GR	18	1	2	0.51	1.66	* S. ornata (3)	3.3	
* S. ornata (2)	BOLD:AAF0734	ES IT (Sar)	3	1	0	0.22	0.33	* S. ornata (3)	4.5	
* S. ornata (3)	BOLD:AAF0731	IT (south) MK	2	0	3	0.15	0.15	* S. ornata (1)	3.3	
Scopula concinnaria	-									
Scopula orientalis	[BOLD:AAE4029]	[TR GE]	0	0	6	-	-	Scopula tessellaria	7.9	
Scopula drenowskii	[BOLD:AAC1708]	[TR]	0	0	15	-	-	Scopula decorata	3.5	
Scopula decorata	BOLD:AAB1548	SE EE LV RU PT ES IT GR	11	1	24	0.21	0.62	Scopula drenowskii	3.5	([78])
Scopula honestata	-									
Scopula subtilata	[BOLD:ABA0178]	[TR]	0	0	1	-	-	Scopula submutata	6.4	
Scopula submutata (1-2)	pooled	ES IT HR MK GR	10	1	12			Scopula vigilata	5.2	([79,80])
* S. submutata (1)	BOLD:AAC4899	IT HR MK GR	9	0	11	0.77	1.55	* S. s. nivellearia (2)	4.2	([81])
* S. s. nivellearia (2)	BOLD:AAE1627	ES	1	1	0	-	-	* S. submutata (1)	4.2	
Scopula vigilata	BOLD:AAC2102	IT GR	14	0	0	0.06	0.31	Scopula submutata	5.2	
Scopula rubiginata	BOLD:AAC2115	FI NL FR DE CH ES IT SI *	13	6	3	0.56	1.14	Scopula ochraceata	5.1	(*[82])
Scopula ochraceata	BOLD:AAJ5890	GR	2	0	3	0.31	0.31	Scopula rubiginata	5.1	
Scopula turbidaria	[BOLD:AAD9162]	[MA]	0	0	1	-	-	Scopula turbulentaria	1.8	([83])
Scopula turbulentaria	BOLD:AAD9162	IT GR	2	1	5	0.32	0.32	Scopula turbidaria	1.8	([84,85])
Scopula decolor	BOLD:AAB6665	ES	1	0	9	-	-	Scopula imitaria	0.0	([86])
Scopula imitaria	BOLD:AAB6665	GB NL FR ES IT HR GR	25	2	8	0.17	1.13	Scopula decolor	0.0	([87])
Scopula rubellata	-									
Scopula scalercii	BOLD:AAB6574	IT	1	1	0	-	-	Scopula beckeraria	0.0	([88])

[76] long distance from nearest neighbor supporting the hypothesis of an old evolutionary lineage for that taxon (cf. Hausmann 2004)
[77] another BIN from outside Europe
[78] nearest neighbor inferred from non-European data; next neighboring species in Europe: *Scopula submutata* at a distance of 7.0%
[79] another BIN from outside Europe
[80] closer neighbor outside Europe: sister species *Scopula transcaspica* from eastern Turkey, Transcaucasus and Iran sharing the haplotype of *Scopula submutata taurilibanotica* (distance 0.0%), questioning its current taxonomic status as separate species despite constant differences in genitalia (cf. Hausmann 2004).
[81] BIN including several regional clusters
[82] further countries with barcoded specimens: HR BG GR
[83] BIN split probable as soon as more specimens of *S. tubidaria* will be barcoded
[84] another BIN from TM
[85] nearest neighbor inferred from non-European data; next neighboring species in Europe: *Scopula ochraceata* at a distance of 5.2%
[86] *Scopula imitaria* and *S. decolor* morphologically well separated; in Hausmann 2004 placed in the same group but not side by side, requiring correction. Exact barcode sharing not yet observed in the same country: In Spain *S. imitaria* (Andalusia) diverging from *S. decolor* (Catalonia) by 0.8%.
[87] another BIN from outside Europe supporting taxonomic status of subspecies *syriacaria* from southern Turkey and Cyprus to Israel diverging by 1.8%.
[88] nearest neighbor inferred from non-European data; genetic data supporting downgrading of *scalercii* to subspecies of *S. beckeraria*, differences in genitalia, however, constant

	BIN (URI)	Countries in EU	BC	AD	OE	Var mean	Var max	Nearest Neighbor in EU	Distance to NN (%)	Notes
Scopula beckeraria	[BOLD:AAB6574]	[TR AM AZ KZ TM etc.]	0	0	26	-	-	Scopula scalercii	0.0	
Scopula incanata (1-3)	pooled	FI SE RU DE AT IT MK	21	0	2	1.24	2.99	Scopula marginepunctata	3.5	([89])
* S. incanata (1)	BOLD:AAC9956	FI SE RU DE AT IT	15	0	0	0.42	1.24	* S. incanata (2)	1.6	
* S .incanata (2)	BOLD:ACF2292	DE MK	2	0	0	0.00	0.00	* S. incanata (1)	1.6	
* S. incanata (3)	BOLD:ACF0740	ES, IT (centre)	4	0	0	0.00	0.00	* S. incanata (1)	2.3	
Scopula marginepunctata (1-2)	pooled	RU NL FR DE AT ES IT *	35	3	29	2.17	3.96	Scopula incanata	3.5	(*[90,91])
* S. marginepunctata (1)	BOLD:AAA8392	FR IT HR BG GR	16	3	24	0.97	2.03	* S. marginepunctata (2)	2.8	
* S. marginepunctata (2)	BOLD:ACF3081	ES, IT, UK, AT, FR, NL, DE	19	0	2	1.07	2.21	* S. marginepunctata (1)	2.8	([92])
Scopula luridata	[BOLD:AAC4836]	[TR IL YE ET]	0	0	12	-	-	Scopula rufomixtaria	6.3	([93])
Scopula immutata	BOLD:AAC7473	FI GB NL FR DE IT	14	0	0	0.33	0.92	Scopula subpunctaria	5.1	
Scopula frigidaria	BOLD:AAA5384	FI	3	0	79	0.00	0.00	Scopula ternata	0.0	([94])
Scopula ternata	BOLD:AAA5384	NO SE FI DE AT IT	9	0	0	0.70	1.39	Scopula frigidaria	0.0	([95])
Scopula floslactata	BOLD:AAC5048	FI GB FR DE AT IT	15	2	1	0.18	0.62	Scopula emutaria	5.5	
Scopula subpunctaria	BOLD:AAD3949	FR DE IT	6	0	9	0.38	0.93	Scopula immutata	5.1	
Scopula flaccidaria	[BOLD:AAH9749]	[IL]	0	0	2	-	-	Scopula subpunctaria	6.2	
Scopula emutaria	BOLD:AAH9750	GB FR ES IT	7	0	0	0.97	1.86	Scopula floslactata	5.5	
Scopula minorata	BOLD:AAA9357	ES IT	16	4	47	0.78	1.82	Scopula nigropunctata	6.9	
Scopula cajanderi	-									
Scopula divisaria	-									
Scopula arenosaria	BOLD:AAP3090	KZ (westernmost)	2	0	1	0.00	0.00	Scopula albiceraria	3.1	([96])
Scopula albiceraria	[BOLD:AAM0074]	[RU MN]	0	0	4	-	-	Scopula arenosaria	3.1	
Scopula immistaria	[BOLD:AAE9783]	[TR GE IR]	0	0	10	-	-	Scopula albiceraria	5.0	([97])
Scopula confinaria (1-3)	pooled	IT HR GR	12	0	0	0.87	2.19	Scopula alba	0.5	([98,99])
* S. confinaria (1)	BOLD:AAD1171	GR IT (Sic)	5	0	0	1.15	1.55	* S. alba	0.5	
* S. confinaria (2)	BOLD:ACE8227	IT (north, centre) HR	5	0	0	0.11	0.20	* S. confinaria (3)	1.1	
* S. confinaria (3)	BOLD:ACF4768	GR (centre, south)	2	0	0	0.00	0.00	* S. confinaria (2)	1.1	
Scopula alba	BOLD:AAD1171	FR (Cor) IT	10	1	0	0.17	0.87	Scopula confinaria	0.5	([99])
Scopula mentzeri	BOLD:AAJ5728	GR (Cre)	1	0	0	-	-	Scopula alba	3.4	
Scopula rufomixtaria	BOLD:AAJ5941	ES	2	0	1	0.62	0.62	Scopula alba	3.9	([100])
Scopula asellaria (1-2)	pooled	ES IT (Sic)	5	0	3	1.37	2.70	Scopula rufomixtaria	5.4	

[89] another BIN from outside Europe
[90] further countries with barcoded specimens: HR BG GR
[91] two other BINs outside Europe
[92] BIN in Italy restricted to Sicily and Sardinia, in Austria so far to the west
[93] nearest neighbor outside Europe: Middle East *Scopula chalcographata* (6.1%).
[94] *S. ternata* in northern Europe with two haplotypes, one of them diverging from that of *S. frigidaria* by 1.4%, the other one – occasionally - barcode-sharing, probably due to introgression.
[95] central European and main northern European cluster of *S. ternata* diverging by 0.9%.
[96] nearest neighbor inferred from non-European data.
[97] nearest neighbor inferred from non-European data.
[98] this and the following 5 species were combined with the genus name *Glossotrophia* in Hausmann (2004), the latter downgraded to synonym of *Scopula* in Sihvonen (2005).
[99] so far, no exact barcode sharing observed between *S. confinaria* and *S. alba*. At the sites of (almost) sympatrical occurrence in central and southern Italy diverging by 1.1%. Nevertheless the complicated pattern of regional and intrapopulational clusters does not resolve both species clearly into two lineages. Requiring further study involving a large sample of dissected and barcoded specimens. Turkish sister species *S. diffinaria* with different BIN.
[100] closer neighbor outside Europe: Middle East *Scopula chalcographata* (3.6%).

	BIN (URI)	Countries in EU	BC	AD	OE	Var mean	Var max	Nearest Neighbor in EU	Distance to NN (%)	Notes
* S. a. dentatolineata (1)	BOLD:AAF0664	ES	4	0	0	0.59	0.83	* S. a. romanaria (2)	2.5	
* S. a. romanaria (2)	BOLD:ABZ1817	IT (Sic)	1	0	3	-	-	* S. a. dentatolineata (1)	2.5	([101])
Scopula sacraria	[BOLD:AAD8980]	[TR LB IL JO]	0	0	11	-	-	Scopula alba	4.4	([102])
Scopula rufinaria	-									([103])
Problepsis ocellata	[BOLD:ACF3550]	[CY TR LB IL OM]	0	0	16	-	-	Idaea obliquaria	7.3	([104])
Rhodostrophia jacularia	[BOLD:AAF7287]	[CN MN RU: Altai]	0	0	7	-	-	Rhodostrophia badiaria	7.0	([105])
Rhodostrophia vibicaria (1-2)	pooled	FI NL DE ES IT MK	20	2	23	1.04	4.45	Rhodostrophia calabra	7.3	([106])
* R. vibicaria (1)	BOLD:AAB6658	FI NL DE ES IT MK	19	1	22	0.72	1.82	* R. vibicaria (2)	4.1	
* R. vibicaria (2)	BOLD:AAB6657	FI	1	0	1	-	-	* R. vibicaria (1)	4.1	([107])
Rhodostrophia pudorata (1-2)	pooled	ES IT	3	1	1	2.42	3.64	Rhodostrophia calabra	3.9	
* R. p. sicanaria (1)	BOLD:AAZ7866	IT	2	0	0	0.46	0.46	* R. p. perezaria (2)	3.2	
* R. p. perezaria (2)	BOLD:AAI0390	ES	1	1	1	-	-	* R. p. sicanaria (1)	3.2	
Rhodostrophia cretacaria	BOLD:AAP4129	GR (Cre)	1	0	0	-	-	Rhodostrophia calabra	3.5	
Rhodostrophia calabra	BOLD:AAC3967	ES IT HR GR	7	2	1	0.50	1.03	Rhodostrophia discopunctata	0.0	([108])
Rhodostrophia tabidaria	BOLD:AAC3967	GR (Lesbos)	1	0	0	-	-	Rhodostrophia calabra	0.3	
Rhodostrophia discopunctata	[BOLD:AAC3967]	[TR LB IL JO]	0	0	13	-	-	Rhodostrophia calabra	0.0	([108])
Rhodostrophia badiaria	[BOLD:AAF1086]	[TR IR]	0	0	7	-	-	Rhodostrophia jacularia	7.0	([109])
Timandra comae	BOLD:AAB0828	FI LT DK GB NL DE CH*	44	1	3	0.41	1.66	Timandra griseata	0.2	(*[110,111])
Timandra griseata	BOLD:AAB0828	FI	11	0	0	0.69	1.60	Timandra comae	0.2	([111])
Timandra rectistrigaria	-									
Cyclophora pendularia	BOLD:AAE2841	FI FR DE	5	2	0	0.00	0.00	Cyclophora ruficiliaria	2.3	
Cyclophora albipunctata	BOLD:ACF3607	SE FI GB FR DE IT	12	1	0	0.27	0.64	Cyclophora ruficiliaria	2.2	([112])
Cyclophora lennigiaria	BOLD:ABZ2916	DE ES	4	1	0	0.08	0.16	Cyclophora albiocellaria	2.0	
Cyclophora albiocellaria	BOLD:ABZ2915	HR GR	2	0	2	0.00	0.00	Cyclophora ariadne	0.9	
Cyclophora ariadne	BOLD:ABZ2915	GR (Cre)	4	0	0	0.15	0.31	Cyclophora albiocellaria	0.9	
Cyclophora serveti	-									
Cyclophora annularia	BOLD:AAD6103	FI FR DE IT	8	2	3	0.18	0.46	Cyclophora ruficiliaria	2.5	
Cyclophora puppillaria	BOLD:AAB2523	PT ES IT HR	22	1	12	0.01	0.17	Cyclophora ruficiliaria	3.1	
Cyclophora quercimontaria	BOLD:AAB4698	FI LV DE	2	1	0	0.00	0.00	Cyclophora punctaria	0.0	([113])

---

[101] examined from Pantelleria island south of Sicily, subspecific assignment awaiting confirmation (cf. Hausmann 2004).
[102] another BIN in IR.
[103] closely related sister species *S. rufinularia* from Altai (KZ, RU) with several barcodes, GUID: BOLD:AAC1556. Combined with the genus name *Holarctias* in Hausmann (2004), the latter downgraded to synonym of *Scopula* in Sihvonen (2005).
[104] closer neighbor outside Europe: *Problepsis erythra* from Ethiopia (3.0%).
[105] another BIN in MN.
[106] another BIN in IR.
[107] same haplotype occurring in TR.
[108] *Rhodostrophia calabra* occasionally sharing the exact haplotype of Turkish specimens of *R. discopunctata*, but barcode sharing not yet observed in Europe. Current taxonomy (cf. Hausmann 2004) possibly requiring revision in the light of the COI data.
[109] sharing BIN but not the exact haplotype with *R. tumulosa* and *R. iranica* from IR.
[110] further countries with barcoded specimens: AT IT HR.
[111] populations from United Kingdom assigned to *T. comae* according to current taxonomy (cf. Hausmann 2004). 6 out of 11 barcoded specimens from the British Isles, however, clustering close to *T. griseata*. Requiring detailed review and integrated analysis. When quoting these 6 specimens as *T. griseata*, mean variation of *T. comae* drops to 0.08%, maximum variation to 0.31%. Distance between both species increases to 0.9%.
[112] closer neighbor outside Europe: North American sister species *Cyclophora pendulinaria* (1.7%, BIN-sharing), East Asian subspecies *C. a. griseolata* diverging by 1.1%.

	BIN (URI)	Countries in EU	BC	AD	OE	Var mean	Var max	Nearest Neighbor in EU	Distance to NN (%)	Notes
Cyclophora ruficiliaria	BOLD:ABX4957	GB DE IT GR	11	4	0	0.09	0.33	Cyclophora ariadne	2.2	
Cyclophora hyponoea	-									
Cyclophora porata	BOLD:AAC9576	GB FR ES IT GR	14	1	0	0.03	0.16	Cyclophora ruficiliaria	3.2	
Cyclophora suppunctaria	BOLD:AAB4698	IT	8	2	1	0.12	0.31	Cyclophora punctaria	0.2	([114])
Cyclophora punctaria	BOLD:AAB4698	FI GB NL FR DE PT IT	35	3	0	0.29	1.64	Cyclophora quercimontaria	0.0	([115])
Cyclophora linearia	BOLD:ABX5086	FI GB FR DE AT PT IT HR	24	3	6	0.02	0.16	Cyclophora ruficiliaria	2.5	
Rhodometra sacraria (1-2)	pooled	FR PT ES IT HR GR	19	4	48	1.33	6.75	Cyclophora annularia	8.3	([116])
* R. sacraria (1)	BOLD:AAA8983	FR PT ES IT HR GR	18	4	48	0.73	2.41	* R. sacraria (2)	6.1	
* R. sacraria (2)	BOLD:AAQ1498	ES (south)	1	0	0	-	-	* R. sacraria (1)	6.1	([117])
Casilda antophilaria	BOLD:AAW4090	GR	3	0	2	1.03	1.24	Casilda consecraria	7.0	([118,119])
Casilda consecraria	[BOLD:AAI6548]	[IL]	0	0	3	-	-	Casilda antophilaria	7.0	([120])
Ochodontia adustaria	[BOLD:ABA3565]	[GE IR MN]	0	0	3	-	-	Timandra comae	12.1	([121])
Lythria plumularia	BOLD:AAM0981	FR CH	2	0	0	0.33	0.33	Lythria purpuraria	7.1	
Lythria purpuraria	BOLD:AAC9098	ES IT GR	6	1	12	1.07	1.91	Lythria plumularia	7.1	([122])
Lythria cruentaria (1-2)	pooled	FI DE AT IT	8	0	0	1.09	2.18	Lythria sanguinaria	7.8	([123])
* L. cruentaria (1)	BOLD:AAE8007	FI DE AT	5	0	0	0.12	0.31	* L. cruentaria (2)	1.8	
* L. cruentaria (2)	BOLD:ACE5498	IT (south)	3	0	0	0.10	0.15	* L. cruentaria (1)	1.8	
Lythria sanguinaria	BOLD:AAX6175	ES	2	1	0	0.00	0.00	Lythria cruentaria	7.8	

[113] correct identification (all barcoded specimens were females where genitalia do not exhibit unambiguous differential characters) still awaiting confirmation. Since morphology of male genitalia is strikingly different between both species, introgression is supposed as the reason for the barcode-sharing rather than a very young divergence time.

[114] though occurring in two haplotypes, there is always a constant minimum distance of one base pair from *C. punctaria*, making the barcodes of *C. suppunctaria* unambiguous.

[115] large variation due to an anomalous specimen from the Netherlands

[116] closer neighbor outside Europe: *Rhodometra intervenata* from eastern Africa (5.9%). Distance from European *Casilda antophilaria* 8.9%.

[117] strongly diverging from 70 other COI sequences of this species, possibly referring to a pseudogene?

[118] nearest neighbor inferred from non-European data.

[119] another BIN from outside Europe.

[120] another BIN from southern KZ.

[121] long distance from nearest neighbor in the Timandrini-lineage of Sterrhinae supporting the hypothesis of an old evolutionary lineage for that taxon (cf. Hausmann 2004). There are several ‘closer neighbors’ in Geometridae (e.g. Eupitheciini) due to long branch attraction.

[122] another BIN from outside Europe.
