# Supplementary material for: Genetic Patterns in European Geometrid Moths Revealed by the Barcode Index Number (BIN) System
Source: PLoS One. 2013 Dec 17;8(12):e84518. doi: 10.1371/journal.pone.0084518 (PMC3866169; doi:10.1371/journal.pone.0084518)
Supplement: Appendix S2 — GenBank Accession numbers. List of specimen-IDs (from BOLD database), GenBank Accession numbers, and species name, for the European geometrid vouchers with barcodes. (PDF) [file pone.0084518.s002.pdf]

Appendix S2: GenBank Accession numbers

List of specimen-IDs (from BOLD database), GenBank Accession numbers, and species name, for the European geometrid vouchers with barcodes.

Process ID	Sample ID	GenBank Accession	Subfamily	Species
LENOA778-11	LN-BD0785	KF807090	Geometrinae	Aplasta ononaria
LENOA779-11	LN-BD0786	KF807431	Geometrinae	Aplasta ononaria
GWOTD327-12	BC ZSM Lep 63458	KF807601	Geometrinae	Aplasta ononaria
GWOTI872-12	BC ZSM SS Lep 0017	KF808081	Geometrinae	Aplasta ononaria
NAGEO020-09	09-JDWGEO-020	GU669369	Archiearinae	Archiearis parthenias
GWOAL233-09	BC AL Geo 00138	KF807717	Archiearinae	Archiearis parthenias
GWOAL232-09	BC AL Geo 00137	KF807838	Archiearinae	Archiearis parthenias
LEFIC985-10	MM05391	HM872800	Archiearinae	Archiearis parthenias
GWOAL230-09	BC AL Geo 00135	KF807204	Archiearinae	Archiearis parthenias
LEFIA042-10	MM00444	HM396391	Archiearinae	Archiearis parthenias
GWOAL231-09	BC AL Geo 00136	KF807497	Archiearinae	Archiearis parthenias
LEFIB077-10	MM00378	HM870986	Archiearinae	Archiearis parthenias
GWORE2181-09	BC ZSM Lep 24004	HM393529	Archiearinae	Boudinotiana notha
LEFIF019-10	MM10458	HM874733	Archiearinae	Boudinotiana notha
LEFIF017-10	MM10456	HM874731	Archiearinae	Boudinotiana notha
GWOAL228-09	BC AL Geo 00133	KF807202	Archiearinae	Boudinotiana notha
GWOSO376-11	BC ZSM Lep 44222	KF808062	Archiearinae	Boudinotiana notha
GWOTG096-12	BC ZSM Lep 58097	KF808050	Archiearinae	Boudinotiana notha
GWORM190-09	BC ZSM Lep 24239	GU687171	Archiearinae	Boudinotiana notha
LENOA772-11	LN-BD0779	KF807085	Archiearinae	Boudinotiana notha
LEFIF018-10	MM10457	HM874732	Archiearinae	Boudinotiana notha
LENOA771-11	LN-BD0778	KF807527	Archiearinae	Boudinotiana notha
GWOAL225-09	BC AL Geo 00130	KF808080	Archiearinae	Boudinotiana notha
GWOAL226-09	BC AL Geo 00131	KF808102	Archiearinae	Boudinotiana notha
GWOAL224-09	BC AL Geo 00129	KF807928	Archiearinae	Boudinotiana touranginii
GWOAL221-09	BC AL Geo 00126	KF807076	Archiearinae	Boudinotiana touranginii
GWOAL220-09	BC AL Geo 00125	KF807893	Archiearinae	Boudinotiana touranginii
GWOAL223-09	BC AL Geo 00128	KF807797	Archiearinae	Boudinotiana touranginii
GWOAL194-09	BC AL Geo 00099	KF807726	Archiearinae	Boudinotiana touranginii
GWOAL222-09	BC AL Geo 00127	KF807351	Archiearinae	Boudinotiana touranginii
GWOST158-11	BC ZSM Lep 45904	KF807330	Sterrhinae	Brachyglossina hispanaria
GWORU514-10	BC NP 0416	JF848879	Sterrhinae	Brachyglossina hispanaria
GWOSO545-11	BC ZSM Lep 45151	KF807461	Sterrhinae	Casilda antophilaria
GWOSO546-11	BC ZSM Lep 45152	KF807376	Sterrhinae	Casilda antophilaria
GWORP791-09	BC ZSM Lep 19577	HQ957598	Sterrhinae	Casilda antophilaria
GWOSO553-11	BC ZSM Lep 45159	KF808029	Geometrinae	Chlorissa cloraria
GWOSI068-10	RCIM 0068	KF808074	Geometrinae	Chlorissa cloraria
GWORM028-09	BC ZSM Lep 24077	GU687314	Geometrinae	Chlorissa cloraria
GWOSO482-11	BC ZSM Lep 45088	KF807657	Geometrinae	Chlorissa cloraria
PHLAF584-11	TLMF Lep 05754	KF807937	Geometrinae	Chlorissa cloraria
GWOSO509-11	BC ZSM Lep 45115	KF807374	Geometrinae	Chlorissa cloraria
GWOSO510-11	BC ZSM Lep 45116	KF807917	Geometrinae	Chlorissa cloraria
LEFIG160-10	MM14018	HM875840	Geometrinae	Chlorissa viridata
GWOSI067-10	RCIM 0067	KF807841	Geometrinae	Chlorissa viridata
LEFIC353-10	MM03871	HM872196	Geometrinae	Chlorissa viridata
LEFIE832-10	MM10048	HM874551	Geometrinae	Chlorissa viridata
FBLMT887-09	BC ZSM Lep 25447	HM391782	Geometrinae	Chlorissa viridata
PHLSA612-11	TLMF Lep 06067	KF808119	Geometrinae	Chlorissa viridata
GWOSO561-11	BC ZSM Lep 45167	KF807557	Sterrhinae	Cinglis andalusiaria
GWOTI839-12	BC ZSM SS Lep 0174	KF808096	Sterrhinae	Cleta filacearia

GWORC1158-08	BC ZSM Lep 12872	KF807942	Sterrhinae	Cleta filacearia
GWORG014-08	BC ZSM Lep 02834	GU655816	Geometrinae	Comibaena bajularia
LENOA784-11	LN-BD0791	KF807352	Geometrinae	Comibaena bajularia
LEFIG932-10	MM15796	HM876574	Geometrinae	Comibaena bajularia
LEFIK836-10	MM18411	JN271714	Geometrinae	Comibaena bajularia
PHLAA673-09	TLMF Lep 00713	HM426074	Geometrinae	Comibaena bajularia
CGUKA491-09	UKLB6B10	KF807756	Geometrinae	Comibaena bajularia
LEFIG931-10	MM15795	HM876573	Geometrinae	Comibaena bajularia
NLLEA204-12	RMNH.INS.538819	KF807656	Geometrinae	Comibaena bajularia
GWOSQ427-11	BC ZSM Lep 54320	KF807110	Geometrinae	Comibaena bajularia
GWOTD328-12	BC ZSM Lep 63459	KF807095	Geometrinae	Comibaena bajularia
GWORG016-08	BC ZSM Lep 02836	GU655818	Geometrinae	Comibaena bajularia
GWOTI879-12	BC ZSM SS Lep 0024	KF807780	Geometrinae	Comibaena bajularia
GWORG015-08	BC ZSM Lep 02835	GU655817	Geometrinae	Comibaena bajularia
NLLEA621-12	RMNH.INS.540816	KF808010	Geometrinae	Comibaena bajularia
CGUKB158-09	UKLB13C07	KF807268	Geometrinae	Comibaena bajularia
GWOSI084-10	RCIM 0084	KF807597	Sterrhinae	Cyclophora albiocellaria
GWOSO566-11	BC ZSM Lep 45172	KF807177	Sterrhinae	Cyclophora albiocellaria
LENOA797-11	LN-BD0804	KF808092	Sterrhinae	Cyclophora albipunctata
CGUKD437-09	UKLB37E06	KF807708	Sterrhinae	Cyclophora albipunctata
GWORO721-09	BC ZSM Lep 27657	GU688406	Sterrhinae	Cyclophora albipunctata
LEFIE149-10	MM08392	HM873897	Sterrhinae	Cyclophora albipunctata
LENOA796-11	LN-BD0803	KF807156	Sterrhinae	Cyclophora albipunctata
LEFIA274-10	MM01314	HM386618	Sterrhinae	Cyclophora albipunctata
GWOSO503-11	BC ZSM Lep 45109	KF807298	Sterrhinae	Cyclophora albipunctata
ODOPE206-11	BC ZSM Lep 53077	KF807666	Sterrhinae	Cyclophora albipunctata
GWOSZ176-11	BC ZSM Lep 41932	KF807482	Sterrhinae	Cyclophora albipunctata
GWORM031-09	BC ZSM Lep 24080	GU687316	Sterrhinae	Cyclophora albipunctata
LEFIA275-10	MM01315	HM386619	Sterrhinae	Cyclophora albipunctata
GWOSI790-10	BC ZSM Lep 49196	JN285257	Sterrhinae	Cyclophora albipunctata
LEFIL282-10	MM19282	JF854571	Sterrhinae	Cyclophora annularia
LEFIL281-10	MM19281	JF854570	Sterrhinae	Cyclophora annularia
GWOSV139-11	BC ZSM Lep 46170	KF808024	Sterrhinae	Cyclophora annularia
GWORD1086-08	BC ZSM Lep 01838	KF807462	Sterrhinae	Cyclophora annularia
LEFID055-10	MM05654	HM872869	Sterrhinae	Cyclophora annularia
PHLAV048-12	TLMF Lep 07867	KF807390	Sterrhinae	Cyclophora annularia
LENOA795-11	LN-BD0802	KF807418	Sterrhinae	Cyclophora annularia
GWORD1084-08	BC ZSM Lep 01836	KF807392	Sterrhinae	Cyclophora annularia
FBLMV436-09	BC ZSM Lep 28416	GU707364	Sterrhinae	Cyclophora annularia
GWOTG326-12	BC ZSM Lep 60702	KF807382	Sterrhinae	Cyclophora ariadne
GWOTG325-12	BC ZSM Lep 60701	KF807371	Sterrhinae	Cyclophora ariadne
GWOSA837-10	BC ZSM Lep 36703	KF807313	Sterrhinae	Cyclophora ariadne
GWORU542-10	BC NP 0444	KF807532	Sterrhinae	Cyclophora ariadne
GWORU544-10	BC NP 0446	JF848884	Sterrhinae	Cyclophora lennigiaria
GWOSO556-11	BC ZSM Lep 45162	KF807836	Sterrhinae	Cyclophora lennigiaria
GWOTG327-12	BC ZSM Lep 60703	KF807926	Sterrhinae	Cyclophora lennigiaria
GWOTG328-12	BC ZSM Lep 60704	KF808052	Sterrhinae	Cyclophora lennigiaria
MPSC022-11	RMNH.INS.27742	KF807918	Sterrhinae	Cyclophora linearia
CGUKD1049-09	UKLB42B12	HM405793	Sterrhinae	Cyclophora linearia
LEFIL545-10	MM18843	JF854651	Sterrhinae	Cyclophora linearia
GWOTH277-12	BC ZSM Lep 64928	KF807398	Sterrhinae	Cyclophora linearia
GWOSI086-10	RCIM 0086	KF807433	Sterrhinae	Cyclophora linearia
PHLAE316-11	TLMF Lep 04631	JN285757	Sterrhinae	Cyclophora linearia
LEFIJ318-10	MM15918	KF807317	Sterrhinae	Cyclophora linearia
PHLAC573-10	TLMF Lep 02608	JF860141	Sterrhinae	Cyclophora linearia
CGUKB496-09	UKLB16H03	KF807989	Sterrhinae	Cyclophora linearia

GWORD1095-08	BC ZSM Lep 01847	KF807900	Sterrhinae	Cyclophora linearia
LEFIL152-10	MM19152	KF807247	Sterrhinae	Cyclophora linearia
LEFIL151-10	MM19151	KF807244	Sterrhinae	Cyclophora linearia
GWORD1096-08	BC ZSM Lep 01848	KF807135	Sterrhinae	Cyclophora linearia
GWORD1098-08	BC ZSM Lep 01850	KF808078	Sterrhinae	Cyclophora linearia
LENOA800-11	LN-BD0807	KF807875	Sterrhinae	Cyclophora linearia
GWORD1097-08	BC ZSM Lep 01849	KF807117	Sterrhinae	Cyclophora linearia
CGUKD435-09	UKLB37E04	KF807248	Sterrhinae	Cyclophora linearia
LEFIL544-10	MM18842	JF854650	Sterrhinae	Cyclophora linearia
GWOTH276-12	BC ZSM Lep 64927	KF808020	Sterrhinae	Cyclophora linearia
GWORA988-08	BC ZSM Lep 02774	HQ601145	Sterrhinae	Cyclophora linearia
GWORB1607-08	BC ZSM Lep 12569	KF807852	Sterrhinae	Cyclophora linearia
GWORC1139-08	BC ZSM Lep 12853	KF807300	Sterrhinae	Cyclophora linearia
LEEUA515-11	MM20574	KF807409	Sterrhinae	Cyclophora linearia
GWORM032-09	BC ZSM Lep 24081	GU687310	Sterrhinae	Cyclophora linearia
GWORD1083-08	BC ZSM Lep 01835	KF807343	Sterrhinae	Cyclophora pendularia
GWORL683-09	BC ZSM Lep 23630	HM376801	Sterrhinae	Cyclophora pendularia
LEFIA354-10	MM01402	HM386697	Sterrhinae	Cyclophora pendularia
LEFIA394-10	MM01452	HM386736	Sterrhinae	Cyclophora pendularia
LEFIA393-10	MM01451	HM386735	Sterrhinae	Cyclophora pendularia
GWORP973-09	BC ZSM Lep 26580	HM394302	Sterrhinae	Cyclophora porata
GWOSO551-11	BC ZSM Lep 45157	KF807265	Sterrhinae	Cyclophora porata
GWORD425-07	BC ZSM Lep 09575	KF807866	Sterrhinae	Cyclophora porata
CGUKC453-09	UKLB27A07	KF807339	Sterrhinae	Cyclophora porata
GWOTH292-12	BC ZSM Lep 65608	KF807772	Sterrhinae	Cyclophora porata
GWOSO562-11	BC ZSM Lep 45168	KF807170	Sterrhinae	Cyclophora porata
GWORB1693-08	BC ZSM Lep 12655	KF808030	Sterrhinae	Cyclophora porata
CGUKC1012-09	UKLB25H11	HM405728	Sterrhinae	Cyclophora porata
GWORC710-07	BC ZSM Lep 09484	KF807604	Sterrhinae	Cyclophora porata
GWORB1694-08	BC ZSM Lep 12656	KF807528	Sterrhinae	Cyclophora porata
GWOSX958-11	BC ZSM Lep 60194	KF808120	Sterrhinae	Cyclophora porata
CGUKB766-09	UKLB19G03	KF808093	Sterrhinae	Cyclophora porata
LENOA799-11	LN-BD0806	KF807334	Sterrhinae	Cyclophora porata
CGUKC449-09	UKLB27A03	KF807696	Sterrhinae	Cyclophora porata
LENOA803-11	LN-BD0810	KF807134	Sterrhinae	Cyclophora punctaria
GWOSH465-10	BC ZSM Lep 39371	JF851444	Sterrhinae	Cyclophora punctaria
CGUKA576-09	UKLB7B01	KF807740	Sterrhinae	Cyclophora punctaria
LEFIC694-10	MM04677	HM872515	Sterrhinae	Cyclophora punctaria
LEFIC217-10	MM03609	HM872061	Sterrhinae	Cyclophora punctaria
GWORD2015-08	BC ZSM Lep 17970	KF807933	Sterrhinae	Cyclophora punctaria
GWOSN911-11	BC ZSM Lep 54542	KF807930	Sterrhinae	Cyclophora punctaria
NLLEA136-12	RMNH.INS.538751	KF807832	Sterrhinae	Cyclophora punctaria
GWOTI906-12	BC ZSM SS Lep 0051	KF807573	Sterrhinae	Cyclophora punctaria
GWORG034-08	BC ZSM Lep 02854	JF415297	Sterrhinae	Cyclophora punctaria
GWORB1503-08	BC ZSM LepLa 0013	HQ601147	Sterrhinae	Cyclophora punctaria
PHLSA608-11	TLMF Lep 06063	KF808077	Sterrhinae	Cyclophora punctaria
FBLMZ403-12	BC ZSM Lep 61159	KF807571	Sterrhinae	Cyclophora punctaria
CGUKA1015-09	UKLB5E03	HM405666	Sterrhinae	Cyclophora punctaria
GWOSN910-11	BC ZSM Lep 54541	KF807883	Sterrhinae	Cyclophora punctaria
CGUKD800-09	UKLB41D01	KF807849	Sterrhinae	Cyclophora punctaria
NLLEA135-12	RMNH.INS.538750	KF807940	Sterrhinae	Cyclophora punctaria
GWOSN912-11	BC ZSM Lep 54543	KF807541	Sterrhinae	Cyclophora punctaria
CGUKB493-09	UKLB16G12	KF807899	Sterrhinae	Cyclophora punctaria
CGUKD436-09	UKLB37E05	KF807534	Sterrhinae	Cyclophora punctaria
LEFIJ583-10	MM17208	KF808105	Sterrhinae	Cyclophora punctaria
GWOSI791-10	BC ZSM Lep 49197	JN285258	Sterrhinae	Cyclophora punctaria

GWOSP901-11	BC MI 0140	KF807934	Sterrhinae	Cyclophora punctaria
CGUKB616-09	UKLB18B06	KF808123	Sterrhinae	Cyclophora punctaria
GWORE1430-08	BC ZSM Lep 15023	HQ601148	Sterrhinae	Cyclophora punctaria
GWOSZ999-11	BC ZSM Lep 62800	KF807428	Sterrhinae	Cyclophora punctaria
GWORC1141-08	BC ZSM Lep 12855	KF807380	Sterrhinae	Cyclophora punctaria
GWORB1504-08	BC ZSM LepLa 0014	HQ601146	Sterrhinae	Cyclophora punctaria
GWOSM841-11	BC ZSM Lep 42312	JN285632	Sterrhinae	Cyclophora punctaria
LEFIA1079-10	MM00032	KF807401	Sterrhinae	Cyclophora punctaria
CGUKA186-09	UKLB2H10	KF807410	Sterrhinae	Cyclophora punctaria
MPSC088-11	RMNH.INS.27808	KF807357	Sterrhinae	Cyclophora punctaria
CGUKA191-09	UKLB3A03	KF807168	Sterrhinae	Cyclophora punctaria
PHLAC503-10	TLMF Lep 02538	JF860075	Sterrhinae	Cyclophora punctaria
NLLEA479-12	RMNH.INS.540671	KF807295	Sterrhinae	Cyclophora punctaria
MPSC089-11	RMNH.INS.27809	KF807344	Sterrhinae	Cyclophora puppillaria
GWORB1683-08	BC ZSM Lep 12645	KF807970	Sterrhinae	Cyclophora puppillaria
GWORB1684-08	BC ZSM Lep 12646	KF808006	Sterrhinae	Cyclophora puppillaria
GWOSP870-11	BC MI 0109	KF807545	Sterrhinae	Cyclophora puppillaria
GWORD632-08	BC ZSM Lep 14132	KF807816	Sterrhinae	Cyclophora puppillaria
GWOTI904-12	BC ZSM SS Lep 0049	KF808087	Sterrhinae	Cyclophora puppillaria
GWORD633-08	BC ZSM Lep 14133	KF807345	Sterrhinae	Cyclophora puppillaria
GWOSP880-11	BC MI 0119	KF807848	Sterrhinae	Cyclophora puppillaria
GWORB1610-08	BC ZSM Lep 12572	KF807278	Sterrhinae	Cyclophora puppillaria
GWOSP881-11	BC MI 0120	KF807714	Sterrhinae	Cyclophora puppillaria
PHLAB1164-10	TLMF Lep 01964	HQ968376	Sterrhinae	Cyclophora puppillaria
GWORB1606-08	BC ZSM Lep 12568	KF807447	Sterrhinae	Cyclophora puppillaria
GWORB1608-08	BC ZSM Lep 12570	KF808089	Sterrhinae	Cyclophora puppillaria
GWOTI903-12	BC ZSM SS Lep 0048	KF807969	Sterrhinae	Cyclophora puppillaria
GWORD634-08	BC ZSM Lep 14134	KF807451	Sterrhinae	Cyclophora puppillaria
GWOSI085-10	RCIM 0085	KF807684	Sterrhinae	Cyclophora puppillaria
GWORB3623-08	BC ZSM Lep add 0051	KF808012	Sterrhinae	Cyclophora puppillaria
GWORB1609-08	BC ZSM Lep 12571	KF807618	Sterrhinae	Cyclophora puppillaria
GWORB1685-08	BC ZSM Lep 12647	KF807512	Sterrhinae	Cyclophora puppillaria
GWORC705-07	BC ZSM Lep 09479	KF807319	Sterrhinae	Cyclophora puppillaria
GWOSI933-10	BC ZSM Lep 49339	JN285269	Sterrhinae	Cyclophora puppillaria
GWORA644-08	BC ZSM Lep 11512	KF807101	Sterrhinae	Cyclophora puppillaria
GWORD1090-08	BC ZSM Lep 01842	KF807815	Sterrhinae	Cyclophora quercimontaria
LEFIK287-10	MM17862	KF807301	Sterrhinae	Cyclophora quercimontaria
CGUKB649-09	UKLB18E04	KF807654	Sterrhinae	Cyclophora ruficiliaria
GWOSO504-11	BC ZSM Lep 45110	KF807286	Sterrhinae	Cyclophora ruficiliaria
GWORD1089-08	BC ZSM Lep 01841	KF807229	Sterrhinae	Cyclophora ruficiliaria
GWORD1088-08	BC ZSM Lep 01840	KF807710	Sterrhinae	Cyclophora ruficiliaria
CGUKB651-09	UKLB18E06	KF807441	Sterrhinae	Cyclophora ruficiliaria
GWORB1690-08	BC ZSM Lep 12652	KF808091	Sterrhinae	Cyclophora ruficiliaria
GWORB1692-08	BC ZSM Lep 12654	KF807419	Sterrhinae	Cyclophora ruficiliaria
GWORB1689-08	BC ZSM Lep 12651	KF807676	Sterrhinae	Cyclophora ruficiliaria
GWORB1614-08	BC ZSM Lep 12576	KF808053	Sterrhinae	Cyclophora ruficiliaria
GWORB1613-08	BC ZSM Lep 12575	KF807569	Sterrhinae	Cyclophora ruficiliaria
GWORB1691-08	BC ZSM Lep 12653	KF808036	Sterrhinae	Cyclophora ruficiliaria
GWORB1612-08	BC ZSM Lep 12574	KF807807	Sterrhinae	Cyclophora suppunctaria
GWORB1686-08	BC ZSM Lep 12648	KF807732	Sterrhinae	Cyclophora suppunctaria
GWOSP869-11	BC MI 0108	KF807578	Sterrhinae	Cyclophora suppunctaria
GWOSP871-11	BC MI 0110	KF807296	Sterrhinae	Cyclophora suppunctaria
GWOTH291-12	BC ZSM Lep 65607	KF807302	Sterrhinae	Cyclophora suppunctaria
GWORB1611-08	BC ZSM Lep 12573	KF807947	Sterrhinae	Cyclophora suppunctaria
GWORB1687-08	BC ZSM Lep 12649	KF807157	Sterrhinae	Cyclophora suppunctaria

GWORB1688-08	BC ZSM Lep 12650	KF807173	Sterrhinae	Cyclophora suppunctaria
GWORU476-10	BC NP 0378	HM910624	Sterrhinae	Emmiltis pygmaearia
GWOTI838-12	BC ZSM SS Lep 0173	KF807205	Sterrhinae	Emmiltis pygmaearia
GWOSO513-11	BC ZSM Lep 45119	KF807272	Geometrinae	Eucrostes indigenata
GWORD655-08	BC ZSM Lep 14155	KF807580	Geometrinae	Eucrostes indigenata
GWORD708-08	BC ZSM Lep 14208	KF807889	Geometrinae	Eucrostes indigenata
GWOSC945-10	BC ZSM Lep 41561	HQ958259	Geometrinae	Eucrostes indigenata
GWORB1677-08	BC ZSM Lep 12639	KF807504	Geometrinae	Eucrostes indigenata
GWOTH289-12	BC ZSM Lep 65605	KF807087	Geometrinae	Eucrostes indigenata
GWORD654-08	BC ZSM Lep 14154	KF807857	Geometrinae	Eucrostes indigenata
GWORC407-07	BC ZSM Lep 06329	KF807698	Geometrinae	Eucrostes indigenata
GWORE1441-08	BC ZSM Lep 15034	KF807341	Geometrinae	Eucrostes indigenata
LENOA782-11	LN-BD0789	KF807325	Geometrinae	Geometra papilionaria
GWORM026-09	BC ZSM Lep 24075	HQ601339	Geometrinae	Geometra papilionaria
LEFIA170-10	MM01147	HM396515	Geometrinae	Geometra papilionaria
CGUKB509-09	UKLB17A04	KF808100	Geometrinae	Geometra papilionaria
NLLEA593-12	RMNH.INS.540785	KF807476	Geometrinae	Geometra papilionaria
GWOSO479-11	BC ZSM Lep 45085	KF807222	Geometrinae	Geometra papilionaria
CGUKC323-09	UKLB25F07	KF807203	Geometrinae	Geometra papilionaria
LEFIE737-10	MM09837	HM874457	Geometrinae	Geometra papilionaria
GWOTI878-12	BC ZSM SS Lep 0023	KF807397	Geometrinae	Geometra papilionaria
GWOSI174-10	RCIM 0174	KF807975	Geometrinae	Geometra papilionaria
GWORG013-08	BC ZSM Lep 02833	GU655815	Geometrinae	Geometra papilionaria
NLLEA203-12	RMNH.INS.538818	KF808118	Geometrinae	Geometra papilionaria
GWORG012-08	BC ZSM Lep 02832	JF415298	Geometrinae	Geometra papilionaria
LEFIB841-10	MM02809	HM871718	Geometrinae	Geometra papilionaria
CGUKD431-09	UKLB37D12	KF807991	Geometrinae	Geometra papilionaria
LENOA783-11	LN-BD0790	KF807865	Geometrinae	Geometra papilionaria
CGUKB189-09	UKLB13F02	KF807827	Geometrinae	Geometra papilionaria
LEFIA1147-10	MM01146	KF807366	Geometrinae	Geometra papilionaria
GWORD397-07	BC ZSM Lep 09547	KF808142	Desmobathrinae	Gypsochroa renitidata
GWOR4110-09	BC ZSM Lep 21394	HQ601348	Geometrinae	Hemistola chrysoprasaria
GWORL446-09	BC ZSM Lep 22348	GU686855	Geometrinae	Hemistola chrysoprasaria
PHLAC491-10	TLMF Lep 02526	JF860065	Geometrinae	Hemistola chrysoprasaria
GWOTI891-12	BC ZSM SS Lep 0036	KF807109	Geometrinae	Hemistola chrysoprasaria
GWORM029-09	BC ZSM Lep 24078	GU687315	Geometrinae	Hemistola chrysoprasaria
GWOTH942-12	GF Lep 0087	KF808042	Geometrinae	Hemistola chrysoprasaria
GWORL459-09	BC ZSM Lep 22361	GU686844	Geometrinae	Hemistola chrysoprasaria
CGUKC102-09	UKLB23C10	KF807719	Geometrinae	Hemistola chrysoprasaria
GWORM212-09	BC ZSM Lep 24261	GU687163	Geometrinae	Hemistola chrysoprasaria
GWORA2138-09	BC ZSM Lep 26540	GU655417	Geometrinae	Hemistola chrysoprasaria
CGUKB503-09	UKLB16H10	KF807877	Geometrinae	Hemistola chrysoprasaria
GWOSI064-10	RCIM 0064	KF807506	Geometrinae	Hemistola chrysoprasaria
PHLAV304-12	TLMF Lep 08123	KF807263	Geometrinae	Hemistola chrysoprasaria
CGUKA737-09	UKLB8G09	KF808031	Geometrinae	Hemistola chrysoprasaria
PHLSA638-11	TLMF Lep 06093	KF807510	Geometrinae	Hemistola chrysoprasaria
GWOR3778-09	BC ZSM Lep 21062	HQ601349	Geometrinae	Hemistola chrysoprasaria
CGUKA526-09	UKLB6E10	KF807579	Geometrinae	Hemistola chrysoprasaria
GWOTH288-12	BC ZSM Lep 65604	KF807919	Geometrinae	Hemistola chrysoprasaria
CGUKC398-09	UKLB26D12	KF808110	Geometrinae	Hemistola chrysoprasaria
GWORE1434-08	BC ZSM Lep 15027	KF807077	Geometrinae	Hemistola chrysoprasaria
CGUKD409-09	UKLB37C02	KF807839	Geometrinae	Hemistola chrysoprasaria
GWORC396-07	BC ZSM Lep 06318	HQ601350	Geometrinae	Hemistola chrysoprasaria
GWORC681-07	BC ZSM Lep 09455	KF808141	Geometrinae	Hemistola chrysoprasaria
GWORC397-07	BC ZSM Lep 06319	KF808135	Geometrinae	Hemistola chrysoprasaria
LENOA790-11	LN-BD0797	KF807941	Geometrinae	Hemistola chrysoprasaria

PHLAA236-09	TLMF Lep 00276	HM425777	Geometrinae	Hemistola chrysoprasaria
GWOR4512-09	BC NP 0371	KF807736	Geometrinae	Hemistola siciliana
LEFIF208-10	MM10874	HM874902	Geometrinae	Hemithea aestivaria
CGUKC1005-09	UKLB25E01	HM405721	Geometrinae	Hemithea aestivaria
GWOTI885-12	BC ZSM SS Lep 0030	KF807526	Geometrinae	Hemithea aestivaria
GWOR4082-09	BC ZSM Lep 21366	HQ601351	Geometrinae	Hemithea aestivaria
GWORM027-09	BC ZSM Lep 24076	GU687319	Geometrinae	Hemithea aestivaria
GWORG021-08	BC ZSM Lep 02841	GU655820	Geometrinae	Hemithea aestivaria
GWORD581-08	BC ZSM Lep 14081	KF808149	Geometrinae	Hemithea aestivaria
LENOA781-11	LN-BD0788	KF807591	Geometrinae	Hemithea aestivaria
CGUKC1042-09	UKLB26E08	HM405748	Geometrinae	Hemithea aestivaria
CGUKB1003-09	UKLB12G12	HM405681	Geometrinae	Hemithea aestivaria
NLLEA777-12	RMNH.INS.540972	KF807303	Geometrinae	Hemithea aestivaria
GWOTI884-12	BC ZSM SS Lep 0029	KF807236	Geometrinae	Hemithea aestivaria
CGUKA490-09	UKLB6B09	KF807951	Geometrinae	Hemithea aestivaria
NLLEA824-12	RMNH.INS.541019	KF807909	Geometrinae	Hemithea aestivaria
CGUKA560-09	UKLB6H08	KF808095	Geometrinae	Hemithea aestivaria
CGUKB498-09	UKLB16H05	KF808101	Geometrinae	Hemithea aestivaria
LEFIF209-10	MM10875	HM874903	Geometrinae	Hemithea aestivaria
GWOSI066-10	RCIM 0066	KF807916	Geometrinae	Hemithea aestivaria
CGUKB499-09	UKLB16H06	KF807822	Geometrinae	Hemithea aestivaria
PHLAF601-11	TLMF Lep 05771	KF807116	Geometrinae	Hemithea aestivaria
GWORC386-07	BC ZSM Lep 06308	KF807665	Geometrinae	Hemithea aestivaria
PHLAC654-10	TLMF Lep 02689	JF860219	Geometrinae	Hemithea aestivaria
GWORG022-08	BC ZSM Lep 02842	JF415299	Geometrinae	Hemithea aestivaria
GWORC385-07	BC ZSM Lep 06307	KF807817	Geometrinae	Hemithea aestivaria
CGUKD392-09	UKLB37A09	KF807132	Geometrinae	Hemithea aestivaria
PHLAV373-12	TLMF Lep 08192	KF807214	Geometrinae	Hemithea aestivaria
CGUKC406-09	UKLB26E08.fail1	KF808058	Geometrinae	Hemithea aestivaria
LEFID237-10	MM06098	HM873037	Geometrinae	Hemithea aestivaria
LENOA780-11	LN-BD0787	KF807290	Geometrinae	Hemithea aestivaria
GWOSN366-11	BC ZSM Lep 46112	KF807079	Sterrhinae	Idaea albarracina
GWORA2078-09	BC ZSM Lep 26480	GU655411	Sterrhinae	Idaea albarracina
GWORI422-09	BC ZSM Lep 12324	KF807231	Sterrhinae	Idaea albarracina
GWOSO522-11	BC ZSM Lep 45128	KF807389	Sterrhinae	Idaea albitorquata
GWOSN340-11	BC ZSM Lep 46086	KF807118	Sterrhinae	Idaea albitorquata
GWOSN304-11	BC ZSM Lep 46050	KF807088	Sterrhinae	Idaea albitorquata
GWOST146-11	BC ZSM Lep 45892	KF807097	Sterrhinae	Idaea alicantaria
GWORE500-08	BC ZSM Lep 12120	KF807146	Sterrhinae	Idaea alicantaria
GWORE488-08	BC ZSM Lep 12108	KF807944	Sterrhinae	Idaea alicantaria
GWORE501-08	BC ZSM Lep 12121	KF807348	Sterrhinae	Idaea alicantaria
GWORI419-09	BC ZSM Lep 12321	KF807923	Sterrhinae	Idaea alyssumata
GWOTF715-12	BC ZSM Lep 62421	KF807581	Sterrhinae	Idaea alyssumata
GWOSI546-10	BC ZSM Lep 44392	JN285250	Sterrhinae	Idaea alyssumata
GWOTF714-12	BC ZSM Lep 62420	KF807383	Sterrhinae	Idaea alyssumata
GWORI420-09	BC ZSM Lep 12322	KF807662	Sterrhinae	Idaea alyssumata
GWOTF712-12	BC ZSM Lep 62418	KF807589	Sterrhinae	Idaea alyssumata
GWORB3624-08	BC ZSM Lep add 0052	KF807973	Sterrhinae	Idaea alyssumata
GWORQ463-10	BC ZSM Lep 27588	HM903803	Sterrhinae	Idaea alyssumata
GWOTI837-12	BC ZSM SS Lep 0172	KF807449	Sterrhinae	Idaea attenuaria
GWOSO499-11	BC ZSM Lep 45105	KF808065	Sterrhinae	Idaea attenuaria
GWOST180-11	BC ZSM Lep 45926	KF807637	Sterrhinae	Idaea attenuaria
GWORL915-09	BC MI 0062	GU686592	Sterrhinae	Idaea attenuaria
GWOSI934-10	BC ZSM Lep 49340	JN285270	Sterrhinae	Idaea attenuaria
GWOSO493-11	BC ZSM Lep 45099	KF808137	Sterrhinae	Idaea aureolaria

GWORA1690-08	BC ZSM Lep 16511	KF807968	Sterrhinae	Idaea aureolaria
GWORA1683-08	BC ZSM Lep 16504	KF808008	Sterrhinae	Idaea aureolaria
GWORM043-09	BC ZSM Lep 24092	GU687305	Sterrhinae	Idaea aureolaria
LENOA922-11	LN-BD0929	KF807139	Sterrhinae	Idaea aversata
CGUKB517-09	UKLB17B01	KF807556	Sterrhinae	Idaea aversata
GWORD1995-08	BC ZSM Lep 17950	KF807905	Sterrhinae	Idaea aversata
CGUKB190-09	UKLB13F03	KF808147	Sterrhinae	Idaea aversata
CGUKC238-09	UKLB24G04	KF807127	Sterrhinae	Idaea aversata
GWOSI074-10	RCIM 0074	KF807242	Sterrhinae	Idaea aversata
NLLEA226-12	RMNH.INS.538841	KF807513	Sterrhinae	Idaea aversata
GWORB1697-08	BC ZSM Lep 12659	KF807652	Sterrhinae	Idaea aversata
CGUKB520-09	UKLB17B04	KF807645	Sterrhinae	Idaea aversata
PHLAC408-10	TLMF Lep 02443	JF859984	Sterrhinae	Idaea aversata
CGUKA529-09	UKLB6F01	KF808075	Sterrhinae	Idaea aversata
CGUKD362-09	UKLB36G02	KF807648	Sterrhinae	Idaea aversata
GWOTI829-12	BC ZSM SS Lep 0164	KF807610	Sterrhinae	Idaea aversata
CGUKA450-09	UKLB5G04	KF807180	Sterrhinae	Idaea aversata
GWORG005-08	BC ZSM Lep 02825	GU655811	Sterrhinae	Idaea aversata
CGUKB068-09	UKLB12C11	KF807094	Sterrhinae	Idaea aversata
GWORB1632-08	BC ZSM Lep 12594	KF807595	Sterrhinae	Idaea aversata
CGUKB984-09	UKLB22A09	KF808009	Sterrhinae	Idaea aversata
CGUKC378-09	UKLB26C04	KF807535	Sterrhinae	Idaea aversata
GWOTI830-12	BC ZSM SS Lep 0165	KF807502	Sterrhinae	Idaea aversata
CGUKA806-09	UKLB9E08	KF807258	Sterrhinae	Idaea aversata
CGUKB518-09	UKLB17B02	KF807311	Sterrhinae	Idaea aversata
GWORB1696-08	BC ZSM Lep 12658	KF807306	Sterrhinae	Idaea aversata
GWORD1993-08	BC ZSM Lep 17948	KF807762	Sterrhinae	Idaea aversata
PHLAF238-11	TLMF Lep 05408	KF807623	Sterrhinae	Idaea aversata
GWORG004-08	BC ZSM Lep 02824	GU655810	Sterrhinae	Idaea aversata
NLLEA583-12	RMNH.INS.540775	KF807712	Sterrhinae	Idaea aversata
GWOR4060-09	BC ZSM Lep 21344	HQ601372	Sterrhinae	Idaea aversata
CGUKB516-09	UKLB17A11	KF807084	Sterrhinae	Idaea aversata
LENOA923-11	LN-BD0930	KF808115	Sterrhinae	Idaea aversata
GWORB1630-08	BC ZSM Lep 12592	KF808082	Sterrhinae	Idaea aversata
LEFIA431-10	MM01494	HM386772	Sterrhinae	Idaea aversata
LEFIA430-10	MM01493	HM386771	Sterrhinae	Idaea aversata
GWORB1634-08	BC ZSM Lep 12596	KF807251	Sterrhinae	Idaea aversata
PHLSA602-11	TLMF Lep 06057	KF807099	Sterrhinae	Idaea aversata
LEFIB851-10	MM02848	HM871728	Sterrhinae	Idaea aversata
CGUKA121-09	UKLB2C05	KF808041	Sterrhinae	Idaea aversata
GWORB769-07	BC ZSM Lep 01145	HQ601373	Sterrhinae	Idaea aversata
GWOTI828-12	BC ZSM SS Lep 0163	KF807333	Sterrhinae	Idaea aversata
GWORB1698-08	BC ZSM Lep 12660	KF807765	Sterrhinae	Idaea aversata
GWORB1633-08	BC ZSM Lep 12595	KF807863	Sterrhinae	Idaea aversata
GWORD1994-08	BC ZSM Lep 17949	KF807701	Sterrhinae	Idaea aversata
PHLAC360-10	TLMF Lep 02395	JF859940	Sterrhinae	Idaea aversata
GWORD1996-08	BC ZSM Lep 17951	KF807577	Sterrhinae	Idaea aversata
GWORB1716-08	BC ZSM Lep 12678	KF807364	Sterrhinae	Idaea aversata
CGUKB159-09	UKLB13C08	KF807511	Sterrhinae	Idaea aversata
GWORB3619-08	BC ZSM Lep add 0047	KF807437	Sterrhinae	Idaea belemiata
GWORQ468-10	BC ZSM Lep 27593	HM903807	Sterrhinae	Idaea belemiata
CGUKB640-09	UKLB18D07	KF807199	Sterrhinae	Idaea biselata
GWORG080-08	BC ZSM Lep 02900	JF415300	Sterrhinae	Idaea biselata
GWORD1105-08	BC ZSM Lep 01857	KF808026	Sterrhinae	Idaea biselata
CGUKB512-09	UKLB17A07	KF807414	Sterrhinae	Idaea biselata

GWOTI807-12	BC ZSM SS Lep 0142	KF807113	Sterrhinae	Idaea biselata
CGUKC162-09	UKLB23H10	KF807507	Sterrhinae	Idaea biselata
GWORC034-07	BC ZSM Lep 01256	HQ601376	Sterrhinae	Idaea biselata
GWORK521-09	BC ZSM Lep 21851	GU655896	Sterrhinae	Idaea biselata
CGUKC618-09	UKLB28G08	KF807407	Sterrhinae	Idaea biselata
GWORD1107-08	BC ZSM Lep 01859	KF807212	Sterrhinae	Idaea biselata
PHLAC355-10	TLMF Lep 02390	JF859935	Sterrhinae	Idaea biselata
CGUKB563-09	UKLB17E12	KF807733	Sterrhinae	Idaea biselata
GWOR4500-09	BC NP 0359	KF807896	Sterrhinae	Idaea biselata
LEFIB865-10	MM02918	HM871742	Sterrhinae	Idaea biselata
GWOTI808-12	BC ZSM SS Lep 0143	KF807551	Sterrhinae	Idaea biselata
NLLEA840-12	RMNH.INS.541035	KF807149	Sterrhinae	Idaea biselata
GWOR4120-09	BC ZSM Lep 21404	HQ601375	Sterrhinae	Idaea biselata
NLLEA841-12	RMNH.INS.541036	KF808057	Sterrhinae	Idaea biselata
GWOR4128-09	BC ZSM Lep 21412	HQ601374	Sterrhinae	Idaea biselata
CGUKB322-09	UKLB15A05	KF807641	Sterrhinae	Idaea biselata
LEFIF743-10	MM12904	HM875427	Sterrhinae	Idaea biselata
GWORC1176-08	BC ZSM Lep 12890	KF807864	Sterrhinae	Idaea biselata
PHLAV370-12	TLMF Lep 08189	KF808013	Sterrhinae	Idaea biselata
LEFIA444-10	MM01508	HM386785	Sterrhinae	Idaea biselata
GWOTI806-12	BC ZSM SS Lep 0141	KF807321	Sterrhinae	Idaea biselata
LENOA812-11	LN-BD0819	KF807872	Sterrhinae	Idaea biselata
NLLEA842-12	RMNH.INS.541037	KF807154	Sterrhinae	Idaea biselata
CGUKA821-09	UKLB9F11	KF807730	Sterrhinae	Idaea biselata
GWORD1106-08	BC ZSM Lep 01858	KF807081	Sterrhinae	Idaea biselata
LENOA811-11	LN-BD0818	KF807200	Sterrhinae	Idaea biselata
CGUKB522-09	UKLB17B06	KF807939	Sterrhinae	Idaea biselata
GWORB3621-08	BC ZSM Lep add 0049	KF807354	Sterrhinae	Idaea blaesii
GWOR4489-09	BC NP 0348	KF808134	Sterrhinae	Idaea blaesii
GWOSN342-11	BC ZSM Lep 46088	KF808122	Sterrhinae	Idaea blaesii
GWORA2152-09	BC ZSM Lep 26554	GU655414	Sterrhinae	Idaea blaesii
GWORP992-09	BC ZSM Lep 26599	HM903432	Sterrhinae	Idaea calunetaria
GWORP762-09	BC ZSM Lep 19548	HQ957593	Sterrhinae	Idaea calunetaria
GWOSO529-11	BC ZSM Lep 45135	KF807292	Sterrhinae	Idaea calunetaria
GWOSI163-10	RCIM 0163	KF808109	Sterrhinae	Idaea camparia
GWORC1177-08	BC ZSM Lep 12891	KF807778	Sterrhinae	Idaea camparia
GWORM200-09	BC ZSM Lep 24249	GU687170	Sterrhinae	Idaea camparia
GWOSO537-11	BC ZSM Lep 45143	KF808063	Sterrhinae	Idaea camparia
GWOSN325-11	BC ZSM Lep 46071	KF807728	Sterrhinae	Idaea camparia
GWORB1637-08	BC ZSM Lep 12599	KF807468	Sterrhinae	Idaea camparia
GWOSI072-10	RCIM 0072	KF807661	Sterrhinae	Idaea camparia
GWOSO523-11	BC ZSM Lep 45129	KF807958	Sterrhinae	Idaea camparia
GWOSN324-11	BC ZSM Lep 46070	KF808117	Sterrhinae	Idaea camparia
GWORQ475-10	BC ZSM Lep 27600	HM903813	Sterrhinae	Idaea camparia
GWORU510-10	BC NP 0412	HM910647	Sterrhinae	Idaea carvalhoi
GWORE492-08	BC ZSM Lep 12112	KF808011	Sterrhinae	Idaea cervantaria
GWOTG224-12	BC ZSM Lep 60030	KF807745	Sterrhinae	Idaea cervantaria
GWOTF716-12	BC ZSM Lep 62422	KF807309	Sterrhinae	Idaea cervantaria
GWORE506-08	BC ZSM Lep 12126	KF808022	Sterrhinae	Idaea cervantaria
GWORE494-08	BC ZSM Lep 12114	KF807869	Sterrhinae	Idaea cervantaria
GWOTF380-12	BC ZSM Lep 60946	KF807783	Sterrhinae	Idaea cervantaria
GWORE1438-08	BC ZSM Lep 15031	KF808099	Sterrhinae	Idaea cervantaria
GWORE493-08	BC ZSM Lep 12113	KF807246	Sterrhinae	Idaea cervantaria
GWORD2053-08	BC ZSM Lep 18008	KF807898	Sterrhinae	Idaea cervantaria
GWOSF936-10	BC ZSM Lep 46967	KF807773	Sterrhinae	Idaea cervantaria

GWORD426-07	BC ZSM Lep 09576	KF807147	Sterrhinae	Idaea cervantaria
GWOSO539-11	BC ZSM Lep 45145	KF807253	Sterrhinae	Idaea circuitaria
GWOSN329-11	BC ZSM Lep 46075	KF807124	Sterrhinae	Idaea consanguinaria
PHLSA614-11	TLMF Lep 06069	KF807360	Sterrhinae	Idaea consanguinaria
GWOSO498-11	BC ZSM Lep 45104	KF807137	Sterrhinae	Idaea consanguinaria
GWOTI835-12	BC ZSM SS Lep 0170	KF807207	Sterrhinae	Idaea consanguinaria
GWORC1155-08	BC ZSM Lep 12869	KF807946	Sterrhinae	Idaea consanguinaria
PHLSA467-11	TLMF Lep 05922	KF807096	Sterrhinae	Idaea consanguinaria
GWORK632-09	BC ZSM Lep 21962	KF807119	Sterrhinae	Idaea consanguinaria
GWORC1175-08	BC ZSM Lep 12889	KF807181	Sterrhinae	Idaea consolidata
MPSC032-11	RMNH.INS.27752	KF807985	Sterrhinae	Idaea contiguaria
GWOSN332-11	BC ZSM Lep 46078	KF807537	Sterrhinae	Idaea contiguaria
PHLAC410-10	TLMF Lep 02445	JF859986	Sterrhinae	Idaea contiguaria
GWOSN346-11	BC ZSM Lep 46092	KF808043	Sterrhinae	Idaea contiguaria
GWORD412-07	BC ZSM Lep 09562	KF807802	Sterrhinae	Idaea davidi
GWOTI821-12	BC ZSM SS Lep 0156	KF807721	Sterrhinae	Idaea degeneraria
PHLAD781-11	TLMF Lep 04146	JN285755	Sterrhinae	Idaea degeneraria
GWORB1720-08	BC ZSM Lep 12682	KF808114	Sterrhinae	Idaea degeneraria
PHLAD780-11	TLMF Lep 04145	JN285754	Sterrhinae	Idaea degeneraria
GWORD666-08	BC ZSM Lep 14166	KF807406	Sterrhinae	Idaea degeneraria
MPSC073-11	RMNH.INS.27793	KF807499	Sterrhinae	Idaea degeneraria
MPSC076-11	RMNH.INS.27796	KF807530	Sterrhinae	Idaea degeneraria
GWORD688-08	BC ZSM Lep 14188	KF808039	Sterrhinae	Idaea degeneraria
GWORD687-08	BC ZSM Lep 14187	KF808035	Sterrhinae	Idaea degeneraria
GWOTI820-12	BC ZSM SS Lep 0155	KF807972	Sterrhinae	Idaea degeneraria
PHLAC393-10	TLMF Lep 02428	JF859969	Sterrhinae	Idaea degeneraria
GWOSI075-10	RCIM 0075	KF808148	Sterrhinae	Idaea degeneraria
GWOTH941-12	GF Lep 0086	KF807367	Sterrhinae	Idaea degeneraria
GWORD686-08	BC ZSM Lep 14186	KF807536	Sterrhinae	Idaea degeneraria
GWORB1721-08	BC ZSM Lep 12683	KF807674	Sterrhinae	Idaea degeneraria
GWOTI822-12	BC ZSM SS Lep 0157	KF807178	Sterrhinae	Idaea degeneraria
MPSC074-11	RMNH.INS.27794	KF807489	Sterrhinae	Idaea degeneraria
GWOTI819-12	BC ZSM SS Lep 0154	KF807754	Sterrhinae	Idaea degeneraria
GWORP837-09	BC ZSM Lep 19623	HM394258	Sterrhinae	Idaea degeneraria
GWORB1719-08	BC ZSM Lep 12681	KF807347	Sterrhinae	Idaea degeneraria
GWORU512-10	BC NP 0414	JF848878	Sterrhinae	Idaea deitanaria
GWOTI825-12	BC ZSM SS Lep 0160	KF807464	Sterrhinae	Idaea determinata
GWORL934-09	BC MI 0081	GU686571	Sterrhinae	Idaea determinata
GWOSO495-11	BC ZSM Lep 45101	KF807823	Sterrhinae	Idaea determinata
GWOSN311-11	BC ZSM Lep 46057	KF807283	Sterrhinae	Idaea determinata
GWOSO557-11	BC ZSM Lep 45163	KF807962	Sterrhinae	Idaea determinata
GWOSP878-11	BC MI 0117	KF807438	Sterrhinae	Idaea deversaria
LEFIK841-10	MM18416	JN285639	Sterrhinae	Idaea deversaria
GWORB1699-08	BC ZSM Lep 12661	KF808021	Sterrhinae	Idaea deversaria
LEFID699-10	MM06752	HM873460	Sterrhinae	Idaea deversaria
GWOTI826-12	BC ZSM SS Lep 0161	KF808097	Sterrhinae	Idaea deversaria
GWORP982-09	BC ZSM Lep 26589	HM394310	Sterrhinae	Idaea deversaria
PHLAC359-10	TLMF Lep 02394	JF859939	Sterrhinae	Idaea deversaria
LEFIF724-10	MM12803	HM875408	Sterrhinae	Idaea deversaria
FBLMZ567-12	BC ZSM Lep 61323	KF807770	Sterrhinae	Idaea deversaria
GWOTI827-12	BC ZSM SS Lep 0162	KF807742	Sterrhinae	Idaea deversaria
GWORB1700-08	BC ZSM Lep 12662	KF807394	Sterrhinae	Idaea deversaria
GWOSI812-10	BC ZSM Lep 49218	JN285261	Sterrhinae	Idaea deversaria
GWOSI076-10	RCIM 0076	KF807484	Sterrhinae	Idaea deversaria
PHLAF295-11	TLMF Lep 05465	KF807148	Sterrhinae	Idaea deversaria
GWORA1680-08	BC ZSM Lep 16501	KF807288	Sterrhinae	Idaea deversaria

GWOSO534-11	BC ZSM Lep 45140	KF807335	Sterrhinae	Idaea dilutaria
GWORA1678-08	BC ZSM Lep 16499	KF807758	Sterrhinae	Idaea dilutaria
GWORE1446-08	BC ZSM Lep 15039	KF807908	Sterrhinae	Idaea dilutaria
GWOTI802-12	BC ZSM SS Lep 0137	KF807107	Sterrhinae	Idaea dilutaria
GWORD1114-08	BC ZSM Lep 01866	KF807243	Sterrhinae	Idaea dilutaria
FBLMV299-09	BC ZSM Lep 28279	GU707336	Sterrhinae	Idaea dilutaria
GWORA1679-08	BC ZSM Lep 16500	KF807218	Sterrhinae	Idaea dilutaria
GWORC1164-08	BC ZSM Lep 12878	KF807868	Sterrhinae	Idaea dilutaria
CGUKB758-09	UKLB19F07	KF807682	Sterrhinae	Idaea dimidiata
LEFIG480-10	MM14535	HM876156	Sterrhinae	Idaea dimidiata
GWORD1125-08	BC ZSM Lep 01877	KF807887	Sterrhinae	Idaea dimidiata
LEFIA351-10	MM01398	HM386694	Sterrhinae	Idaea dimidiata
GWORL902-09	BC MI 0049	GU686603	Sterrhinae	Idaea dimidiata
GWORD587-08	BC ZSM Lep 14087	KF807990	Sterrhinae	Idaea dimidiata
CGUKC327-09	UKLB25F11	KF807208	Sterrhinae	Idaea dimidiata
CGUKB513-09	UKLB17A08	KF807566	Sterrhinae	Idaea dimidiata
GWORD585-08	BC ZSM Lep 14085	KF807999	Sterrhinae	Idaea dimidiata
GWORL901-09	BC MI 0048	GU686602	Sterrhinae	Idaea dimidiata
NLLEA1183-12	RMNH.INS.544467	KF807796	Sterrhinae	Idaea dimidiata
CGUKD384-09	UKLB37A01	KF807495	Sterrhinae	Idaea dimidiata
GWOTI805-12	BC ZSM SS Lep 0140	KF807469	Sterrhinae	Idaea dimidiata
GWORD588-08	BC ZSM Lep 14088	KF807603	Sterrhinae	Idaea dimidiata
CGUKD983-09	UKLB43C06	KF807516	Sterrhinae	Idaea dimidiata
CGUKC272-09	UKLB25B03	KF807377	Sterrhinae	Idaea dimidiata
GWOSZ1001-11	BC ZSM Lep 62802	KF807704	Sterrhinae	Idaea dimidiata
GWOR4108-09	BC ZSM Lep 21392	HQ601378	Sterrhinae	Idaea dimidiata
GWORC019-07	BC ZSM Lep 01241	HQ601379	Sterrhinae	Idaea dimidiata
LEFIA350-10	MM01397	HM386693	Sterrhinae	Idaea dimidiata
CGUKB981-09	UKLB22A06	KF807114	Sterrhinae	Idaea dimidiata
GWORH003-09	BC ZSM Lep 03669	JF415301	Sterrhinae	Idaea dimidiata
GWOR4127-09	BC ZSM Lep 21411	HQ601377	Sterrhinae	Idaea dimidiata
GWOSP938-11	BC MI 0177	KF807270	Sterrhinae	Idaea dimidiata
GWORE1449-08	BC ZSM Lep 15042	KF807299	Sterrhinae	Idaea dimidiata
CGUKB079-09	UKLB12D10	KF807520	Sterrhinae	Idaea dimidiata
CGUKD571-09	UKLB38H09	KF807759	Sterrhinae	Idaea dimidiata
GWORB1542-08	BC ZSM Lep 12504	KF807416	Sterrhinae	Idaea distinctaria
GWOTI797-12	BC ZSM SS Lep 0132	KF808007	Sterrhinae	Idaea distinctaria
GWOTH290-12	BC ZSM Lep 65606	KF807187	Sterrhinae	Idaea distinctaria
GWORD625-08	BC ZSM Lep 14125	KF807337	Sterrhinae	Idaea distinctaria
GWORB1755-08	BC ZSM Lep 12717	KF807812	Sterrhinae	Idaea distinctaria
GWORL933-09	BC MI 0080	GU686570	Sterrhinae	Idaea distinctaria
GWORK634-09	BC ZSM Lep 21964	KF808014	Sterrhinae	Idaea distinctaria
GWORL905-09	BC MI 0052	GU686598	Sterrhinae	Idaea distinctaria
GWORB1757-08	BC ZSM Lep 12719	KF808127	Sterrhinae	Idaea distinctaria
GWORD624-08	BC ZSM Lep 14124	KF807314	Sterrhinae	Idaea distinctaria
GWORL903-09	BC MI 0050	GU686604	Sterrhinae	Idaea distinctaria
GWOSI167-10	RCIM 0167	KF807810	Sterrhinae	Idaea distinctaria
GWORB1756-08	BC ZSM Lep 12718	KF807867	Sterrhinae	Idaea distinctaria
GWORL904-09	BC MI 0051	GU686605	Sterrhinae	Idaea distinctaria
GWORU482-10	BC NP 0384	KF807561	Sterrhinae	Idaea efflorata
GWORC1171-08	BC ZSM Lep 12885	KF807624	Sterrhinae	Idaea efflorata
GWOSP889-11	BC MI 0128	KF807550	Sterrhinae	Idaea elongaria
GWORC1160-08	BC ZSM Lep 12874	KF807779	Sterrhinae	Idaea elongaria
GWOSO531-11	BC ZSM Lep 45137	KF807850	Sterrhinae	Idaea elongaria
GWOTI816-12	BC ZSM SS Lep 0151	KF807650	Sterrhinae	Idaea elongaria
GWOSI166-10	RCIM 0166	KF807467	Sterrhinae	Idaea elongaria

GWORP836-09	BC ZSM Lep 19622	HM394257	Sterrhinae	Idaea elongaria
GWOSF934-10	BC ZSM Lep 46965	KF807373	Sterrhinae	Idaea elongaria
GWORE622-08	BC ZSM Lep 15907	KF807408	Sterrhinae	Idaea elongaria
GWORP761-09	BC ZSM Lep 19547	HM394194	Sterrhinae	Idaea elongaria
GWORE482-08	BC ZSM Lep 12102	KF807943	Sterrhinae	Idaea elongaria
LEFIA355-10	MM01403	HM386698	Sterrhinae	Idaea emarginata
LEFIA409-10	MM01471	HM386751	Sterrhinae	Idaea emarginata
CGUKC178-09	UKLB24B03	KF807915	Sterrhinae	Idaea emarginata
GWORK522-09	BC ZSM Lep 21852	JF415302	Sterrhinae	Idaea emarginata
LEFIA410-10	MM01472	HM386752	Sterrhinae	Idaea emarginata
NLLEA868-12	RMNH.INS.541063	KF808076	Sterrhinae	Idaea emarginata
GWORL391-09	BC ZSM Lep 22293	GU686901	Sterrhinae	Idaea emarginata
GWORG003-08	BC ZSM Lep 02823	GU655809	Sterrhinae	Idaea emarginata
NLLEA1190-12	RMNH.INS.544474	KF807111	Sterrhinae	Idaea emarginata
GWOSI545-10	BC ZSM Lep 44391	KF807269	Sterrhinae	Idaea eugeniata
GWORB3620-08	BC ZSM Lep add 0048	KF807133	Sterrhinae	Idaea eugeniata
GWORE1437-08	BC ZSM Lep 15030	KF807353	Sterrhinae	Idaea eugeniata
GWOST128-11	BC ZSM Lep 45874	KF807277	Sterrhinae	Idaea eugeniata
MPSC021-11	RMNH.INS.27741	KF808019	Sterrhinae	Idaea eugeniata
GWORC704-07	BC ZSM Lep 09478	KF807692	Sterrhinae	Idaea eugeniata
GWORQ466-10	BC ZSM Lep 27591	HM903805	Sterrhinae	Idaea eugeniata
GWORQ467-10	BC ZSM Lep 27592	HM903806	Sterrhinae	Idaea eugeniata
GWORU509-10	BC NP 0411	JF848877	Sterrhinae	Idaea exilaria
GWOTI787-12	BC ZSM SS Lep 0122	KF807594	Sterrhinae	Idaea filicata
GWORP835-09	BC ZSM Lep 19621	HM394256	Sterrhinae	Idaea filicata
GWORP834-09	BC ZSM Lep 19620	HM394255	Sterrhinae	Idaea filicata
GWORB1774-08	BC ZSM Lep 12736	KF807689	Sterrhinae	Idaea filicata
GWORD698-08	BC ZSM Lep 14198	KF807378	Sterrhinae	Idaea filicata
GWORD697-08	BC ZSM Lep 14197	KF807687	Sterrhinae	Idaea filicata
GWORB1767-08	BC ZSM Lep 12729	KF807858	Sterrhinae	Idaea filicata
GWORD696-08	BC ZSM Lep 14196	KF807752	Sterrhinae	Idaea filicata
GWOSO533-11	BC ZSM Lep 45139	KF807326	Sterrhinae	Idaea filicata
GWOTI788-12	BC ZSM SS Lep 0123	KF807539	Sterrhinae	Idaea filicata
GWORB1765-08	BC ZSM Lep 12727	KF807707	Sterrhinae	Idaea filicata
GWOSI071-10	RCIM 0071	KF807751	Sterrhinae	Idaea filicata
GWORB1766-08	BC ZSM Lep 12728	KF807585	Sterrhinae	Idaea filicata
GWORB1544-08	BC ZSM Lep 12506	KF808033	Sterrhinae	Idaea filicata
GWORB1543-08	BC ZSM Lep 12505	KF807599	Sterrhinae	Idaea filicata
GWOTI791-12	BC ZSM SS Lep 0126	KF807888	Sterrhinae	Idaea flaveolaria
GWOTI792-12	BC ZSM SS Lep 0127	KF807781	Sterrhinae	Idaea flaveolaria
GWORM283-09	BC ZSM Lep 24332	HM376833	Sterrhinae	Idaea fractilineata
GWORE502-08	BC ZSM Lep 12122	KF807834	Sterrhinae	Idaea fractilineata
GWORA2615-09	BC ZSM Lep 31872	HM393269	Sterrhinae	Idaea fractilineata
GWORP784-09	BC ZSM Lep 19570	HQ957597	Sterrhinae	Idaea fractilineata
GWORE509-08	BC ZSM Lep 12129	KF807976	Sterrhinae	Idaea fractilineata
GWORP783-09	BC ZSM Lep 19569	HM394209	Sterrhinae	Idaea fractilineata
GWORE480-08	BC ZSM Lep 12100	KF807558	Sterrhinae	Idaea fractilineata
CGUKB437-09	UKLB16C04.fail1	KF808116	Sterrhinae	Idaea fuscovenosa
GWOSP941-11	BC MI 0180	KF807760	Sterrhinae	Idaea fuscovenosa
GWOSP917-11	BC MI 0156	KF807524	Sterrhinae	Idaea fuscovenosa
CGUKB554-09	UKLB17E03	KF807723	Sterrhinae	Idaea fuscovenosa
LENOA813-11	LN-BD0820	KF808066	Sterrhinae	Idaea fuscovenosa
GWOTI453-12	BC ZSM Lep 66719	KF807518	Sterrhinae	Idaea fuscovenosa
CGUKB536-09	UKLB17C09	KF807774	Sterrhinae	Idaea fuscovenosa
GWORB819-07	BC ZSM Lep 01195	HQ601381	Sterrhinae	Idaea fuscovenosa

CGUKC579-09	UKLB28D05	KF807305	Sterrhinae	Idaea fuscovenosa
CGUKB1033-09	UKLB16C04	HM405709	Sterrhinae	Idaea fuscovenosa
CGUKC051-09	UKLB22G05	KF807259	Sterrhinae	Idaea fuscovenosa
GWORC1162-08	BC ZSM Lep 12876	KF807198	Sterrhinae	Idaea fuscovenosa
GWOR4113-09	BC ZSM Lep 21397	HQ601380	Sterrhinae	Idaea fuscovenosa
CGUKB521-09	UKLB17B05	KF807737	Sterrhinae	Idaea fuscovenosa
GWOSP723-11	BC ZSM Lep add 0143	KF807799	Sterrhinae	Idaea fuscovenosa
CGUKB547-09	UKLB17D08	KF807907	Sterrhinae	Idaea fuscovenosa
LENOA817-11	LN-BD0824	KF807455	Sterrhinae	Idaea fuscovenosa
CGUKB436-09	UKLB16C03	KF807789	Sterrhinae	Idaea fuscovenosa
LENOA814-11	LN-BD0821	KF807992	Sterrhinae	Idaea fuscovenosa
CGUKC388-09	UKLB26D02	KF807675	Sterrhinae	Idaea fuscovenosa
GWORE2033-09	BC ZSM Lep 22431	HM393510	Sterrhinae	Idaea fuscovenosa
GWORZ704-10	BC ZSM Lep 34955	HM914372	Sterrhinae	Idaea gelbrechti
GWORZ702-10	BC ZSM Lep 34953	HM914370	Sterrhinae	Idaea gelbrechti
GWOST117-11	BC ZSM Lep 45863	KF807757	Sterrhinae	Idaea gelbrechti
GWORZ703-10	BC ZSM Lep 34954	HM914371	Sterrhinae	Idaea gelbrechti
FBLMV433-09	BC ZSM Lep 28413	GU707366	Sterrhinae	Idaea humiliata
GWOTI841-12	BC ZSM SS Lep 0176	KF807273	Sterrhinae	Idaea humiliata
GWORD1116-08	BC ZSM Lep 01868	KF807996	Sterrhinae	Idaea humiliata
NLLEA589-12	RMNH.INS.540781	KF807966	Sterrhinae	Idaea humiliata
GWORM083-09	BC ZSM Lep 24132	GU687264	Sterrhinae	Idaea humiliata
LEFIF738-10	MM12875	HM875422	Sterrhinae	Idaea humiliata
GWORM049-09	BC ZSM Lep 24098	GU687294	Sterrhinae	Idaea humiliata
NLLEA213-12	RMNH.INS.538828	KF807197	Sterrhinae	Idaea humiliata
FBLMZ408-12	BC ZSM Lep 61164	KF807472	Sterrhinae	Idaea humiliata
GWOTI840-12	BC ZSM SS Lep 0175	KF807473	Sterrhinae	Idaea humiliata
GWOTI801-12	BC ZSM SS Lep 0136	KF807440	Sterrhinae	Idaea humiliata
GWORD1115-08	BC ZSM Lep 01867	KF807880	Sterrhinae	Idaea humiliata
GWOSO535-11	BC ZSM Lep 45141	KF808111	Sterrhinae	Idaea humiliata
LEFID731-10	MM06789	HM873488	Sterrhinae	Idaea humiliata
LEFID789-10	MM06859	HM873546	Sterrhinae	Idaea humiliata
GWORR691-10	BC ZSM Lep 32187	KF807886	Sterrhinae	Idaea ibizaria
GWORP760-09	BC ZSM Lep 19546	HM394193	Sterrhinae	Idaea incalcarata
GWORM186-09	BC ZSM Lep 24235	GU687175	Sterrhinae	Idaea incisaria
GWORD694-08	BC ZSM Lep 14194	KF807994	Sterrhinae	Idaea infirmaria
GWORL867-09	BC MI 0014	GU686639	Sterrhinae	Idaea infirmaria
GWORL865-09	BC MI 0012	GU686637	Sterrhinae	Idaea infirmaria
GWOTI803-12	BC ZSM SS Lep 0138	KF807753	Sterrhinae	Idaea infirmaria
GWORD636-08	BC ZSM Lep 14136	KF807617	Sterrhinae	Idaea infirmaria
GWORB1770-08	BC ZSM Lep 12732	KF808086	Sterrhinae	Idaea infirmaria
GWORB1769-08	BC ZSM Lep 12731	KF808112	Sterrhinae	Idaea infirmaria
GWORM184-09	BC ZSM Lep 24233	GU687180	Sterrhinae	Idaea infirmaria
GWORC1159-08	BC ZSM Lep 12873	KF808048	Sterrhinae	Idaea infirmaria
GWOSO538-11	BC ZSM Lep 45144	KF807629	Sterrhinae	Idaea infirmaria
GWOSO525-11	BC ZSM Lep 45131	KF807417	Sterrhinae	Idaea infirmaria
GWORD637-08	BC ZSM Lep 14137	KF807412	Sterrhinae	Idaea infirmaria
GWORB1768-08	BC ZSM Lep 12730	KF807820	Sterrhinae	Idaea infirmaria
GWORD695-08	BC ZSM Lep 14195	KF807605	Sterrhinae	Idaea infirmaria
GWORD693-08	BC ZSM Lep 14193	KF807500	Sterrhinae	Idaea infirmaria
GWOSN730-11	BC ZSM Lep 52936	KF807102	Sterrhinae	Idaea inquinata
GWOSC411-10	BC ZSM Lep 36087	HQ566457	Sterrhinae	Idaea inquinata
GWORB831-07	BC ZSM Lep 01207	KF807846	Sterrhinae	Idaea inquinata
GWORM048-09	BC ZSM Lep 24097	GU687301	Sterrhinae	Idaea inquinata
GWORB820-07	BC ZSM Lep 01196	HQ601383	Sterrhinae	Idaea inquinata

GWORE1447-08	BC ZSM Lep 15040	KF807215	Sterrhinae	Idaea inquinata
GWORC680-07	BC ZSM Lep 09454	KF807891	Sterrhinae	Idaea inquinata
GWORC1166-08	BC ZSM Lep 12880	KF808103	Sterrhinae	Idaea inquinata
FBLMU101-09	BC ZSM Lep 25611	GU707202	Sterrhinae	Idaea inquinata
GWORA2146-09	BC ZSM Lep 26548	GU655415	Sterrhinae	Idaea inquinata
GWOTI809-12	BC ZSM SS Lep 0144	KF807474	Sterrhinae	Idaea inquinata
GWOSN706-11	BC ZSM Lep 52912	KF808143	Sterrhinae	Idaea inquinata
GWOSN924-11	BC ZSM Lep 54555	KF807165	Sterrhinae	Idaea inquinata
GWORB822-07	BC ZSM Lep 01198	HQ601382	Sterrhinae	Idaea inquinata
GWOSO502-11	BC ZSM Lep 45108	KF807808	Sterrhinae	Idaea intermedia
GWORM084-09	BC ZSM Lep 24133	HM376819	Sterrhinae	Idaea intermedia
GWOSF932-10	BC ZSM Lep 46963	KF807695	Sterrhinae	Idaea joannisiata
GWOSC932-10	BC ZSM Lep 41548	HQ958254	Sterrhinae	Idaea joannisiata
GWORA2153-09	BC ZSM Lep 26555	HM422533	Sterrhinae	Idaea joannisiata
GWOSO560-11	BC ZSM Lep 45166	KF807894	Sterrhinae	Idaea korbi
GWORC1167-08	BC ZSM Lep 12881	KF807912	Sterrhinae	Idaea laevigata
GWOTI776-12	BC ZSM SS Lep 0111	KF807971	Sterrhinae	Idaea laevigata
GWOSI177-10	RCIM 0177	KF807793	Sterrhinae	Idaea leipnitzi
GWOTI775-12	BC ZSM SS Lep 0110	KF807443	Sterrhinae	Idaea leipnitzi
GWORL917-09	BC MI 0064	GU686586	Sterrhinae	Idaea leipnitzi
GWORL918-09	BC MI 0065	GU686587	Sterrhinae	Idaea leipnitzi
GWORL919-09	BC MI 0066	GU686588	Sterrhinae	Idaea leipnitzi
GWORB2321-08	BC ZSM Lep 11121	KF807365	Sterrhinae	Idaea libycata
GWORO249-09	BC ZSM Lep 19414	HM422781	Sterrhinae	Idaea litigiosaria
GWORZ714-10	BC ZSM Lep 34965	HM914381	Sterrhinae	Idaea litigiosaria
GWOSN350-11	BC ZSM Lep 46096	KF807529	Sterrhinae	Idaea longaria
GWORE487-08	BC ZSM Lep 12107	KF807870	Sterrhinae	Idaea longaria
GWOSO536-11	BC ZSM Lep 45142	KF807924	Sterrhinae	Idaea longaria
GWORB3701-10	BC ZSM Lep 33953	HM903296	Sterrhinae	Idaea longaria
GWORB3702-10	BC ZSM Lep 33954	HM903297	Sterrhinae	Idaea longaria
GWOSP896-11	BC MI 0135	KF807583	Sterrhinae	Idaea longaria
GWORE505-08	BC ZSM Lep 12125	KF807609	Sterrhinae	Idaea longaria
GWOSP899-11	BC MI 0138	KF807844	Sterrhinae	Idaea longaria
GWORE499-08	BC ZSM Lep 12119	KF807266	Sterrhinae	Idaea longaria
GWOSP898-11	BC MI 0137	KF808113	Sterrhinae	Idaea longaria
GWOSP897-11	BC MI 0136	KF807167	Sterrhinae	Idaea longaria
GWORM279-09	BC ZSM Lep 24328	GU687119	Sterrhinae	Idaea mediaria
GWORM817-09	BC ZSM Lep 24392	GU687594	Sterrhinae	Idaea mediaria
GWORI455-09	BC ZSM Lep 12357	KF807091	Sterrhinae	Idaea mediaria
GWOSO520-11	BC ZSM Lep 45126	KF807690	Sterrhinae	Idaea metohiensis
GWORM086-09	BC ZSM Lep 24135	HM376820	Sterrhinae	Idaea metohiensis
GWOTI475-12	BC ZSM Lep 66741	KF807608	Sterrhinae	Idaea minuscularia
GWORE481-08	BC ZSM Lep 12101	KF807466	Sterrhinae	Idaea minuscularia
GWOSF935-10	BC ZSM Lep 46966	KF807632	Sterrhinae	Idaea minuscularia
GWORE498-08	BC ZSM Lep 12118	KF808038	Sterrhinae	Idaea minuscularia
GWORE503-08	BC ZSM Lep 12123	KF807223	Sterrhinae	Idaea minuscularia
GWOTI473-12	BC ZSM Lep 66739	KF807329	Sterrhinae	Idaea minuscularia
GWORE504-08	BC ZSM Lep 12124	KF807210	Sterrhinae	Idaea minuscularia
GWORC703-07	BC ZSM Lep 09477	KF808034	Sterrhinae	Idaea minuscularia
GWORM819-09	BC ZSM Lep 24394	GU687593	Sterrhinae	Idaea minuscularia
GWORE497-08	BC ZSM Lep 12117	KF807262	Sterrhinae	Idaea minuscularia
GWORM280-09	BC ZSM Lep 24329	GU687120	Sterrhinae	Idaea minuscularia
GWORE496-08	BC ZSM Lep 12116	KF807631	Sterrhinae	Idaea minuscularia
GWORE495-08	BC ZSM Lep 12115	KF807195	Sterrhinae	Idaea minuscularia
PHLAC567-10	TLMF Lep 02602	JF860135	Sterrhinae	Idaea moniliata
GWORD1123-08	BC ZSM Lep 01875	KF807570	Sterrhinae	Idaea moniliata

GWORD1124-08	BC ZSM Lep 01876	KF807906	Sterrhinae	Idaea moniliata
GWOSO500-11	BC ZSM Lep 45106	KF808018	Sterrhinae	Idaea moniliata
GWORC1161-08	BC ZSM Lep 12875	KF807565	Sterrhinae	Idaea moniliata
PHLSA461-11	TLMF Lep 05916	KF808047	Sterrhinae	Idaea moniliata
FBLMZ407-12	BC ZSM Lep 61163	KF807803	Sterrhinae	Idaea moniliata
GWOTI784-12	BC ZSM SS Lep 0119	KF807814	Sterrhinae	Idaea moniliata
CGUKB1008-09	UKLB13E07	HM405685	Sterrhinae	Idaea muricata
LEFIE573-10	MM09467	HM874296	Sterrhinae	Idaea muricata
LEFIA879-10	MM09756	HM387017	Sterrhinae	Idaea muricata
GWORD1121-08	BC ZSM Lep 01873	KF807647	Sterrhinae	Idaea muricata
GWOSN339-11	BC ZSM Lep 46085	KF807936	Sterrhinae	Idaea muricata
GWORL462-09	BC ZSM Lep 22364	GU686838	Sterrhinae	Idaea muricata
PHLAA692-09	TLMF Lep 00732	HM426092	Sterrhinae	Idaea muricata
CGUKB091-09	UKLB12E10	KF807190	Sterrhinae	Idaea muricata
ODOPE571-11	BC ZSM Lep 50972	KF807854	Sterrhinae	Idaea muricata
GWORK472-09	BC ZSM Lep 21802	JF415303	Sterrhinae	Idaea muricata
LEFID761-10	MM06826	HM873518	Sterrhinae	Idaea muricata
PHLAH719-12	TLMF Lep 08538	KF807271	Sterrhinae	Idaea muricata
GWOSP724-11	BC ZSM Lep add 0144	KF807979	Sterrhinae	Idaea muricata
GWOST097-11	BC ZSM Lep 45843	KF808107	Sterrhinae	Idaea mustelata
GWORE489-08	BC ZSM Lep 12109	KF807921	Sterrhinae	Idaea mustelata
GWOSP910-11	BC MI 0149	KF807425	Sterrhinae	Idaea mutilata
GWOSN353-11	BC ZSM Lep 46099	KF807358	Sterrhinae	Idaea obliquaria
GWORL931-09	BC MI 0078	GU686576	Sterrhinae	Idaea obsoletaria
GWORI454-09	BC ZSM Lep 12356	KF807856	Sterrhinae	Idaea obsoletaria
GWORI458-09	BC ZSM Lep 12360	KF808132	Sterrhinae	Idaea obsoletaria
GWORI457-09	BC ZSM Lep 12359	KF807340	Sterrhinae	Idaea obsoletaria
GWORD635-08	BC ZSM Lep 14135	KF807362	Sterrhinae	Idaea obsoletaria
GWORB1746-08	BC ZSM Lep 12708	KF807442	Sterrhinae	Idaea obsoletaria
PHLAC570-10	TLMF Lep 02605	JF860138	Sterrhinae	Idaea obsoletaria
GWOTI782-12	BC ZSM SS Lep 0117	KF807445	Sterrhinae	Idaea obsoletaria
GWORB1745-08	BC ZSM Lep 12707	KF807315	Sterrhinae	Idaea obsoletaria
PHLSA620-11	TLMF Lep 06075	KF807194	Sterrhinae	Idaea obsoletaria
GWORL866-09	BC MI 0013	GU686638	Sterrhinae	Idaea obsoletaria
GWORQ469-10	BC ZSM Lep 27594	HM903808	Sterrhinae	Idaea obsoletaria
GWORI459-09	BC ZSM Lep 12361	KF807727	Sterrhinae	Idaea obsoletaria
GWOSI547-10	BC ZSM Lep 44393	JN285251	Sterrhinae	Idaea obsoletaria
GWORD714-08	BC ZSM Lep 14214	KF807729	Sterrhinae	Idaea obsoletaria
GWORL868-09	BC MI 0015	GU686640	Sterrhinae	Idaea obsoletaria
GWORL869-09	BC MI 0016	GU686633	Sterrhinae	Idaea obsoletaria
GWOTI800-12	BC ZSM SS Lep 0135	KF807851	Sterrhinae	Idaea obsoletaria
GWORI453-09	BC ZSM Lep 12355	KF807670	Sterrhinae	Idaea obsoletaria
GWORI451-09	BC ZSM Lep 12353	KF807669	Sterrhinae	Idaea obsoletaria
GWOSO526-11	BC ZSM Lep 45132	KF807646	Sterrhinae	Idaea obsoletaria
GWORL932-09	BC MI 0079	GU686577	Sterrhinae	Idaea obsoletaria
GWOSI070-10	RCIM 0070	KF807963	Sterrhinae	Idaea ochrata
GWORE507-08	BC ZSM Lep 12127	KF807515	Sterrhinae	Idaea ochrata
GWORD615-08	BC ZSM Lep 14115	KF807615	Sterrhinae	Idaea ochrata
GWORO247-09	BC ZSM Lep 19412	GU688360	Sterrhinae	Idaea ochrata
GWORD617-08	BC ZSM Lep 14117	KF807576	Sterrhinae	Idaea ochrata
NLLEA903-12	RMNH.INS.544357	KF807179	Sterrhinae	Idaea ochrata
PHLSA622-11	TLMF Lep 06077	KF807141	Sterrhinae	Idaea ochrata
GWOTI834-12	BC ZSM SS Lep 0169	KF808056	Sterrhinae	Idaea ochrata
GWORL675-09	BC ZSM Lep 23622	GU686692	Sterrhinae	Idaea ochrata
GWOTI833-12	BC ZSM SS Lep 0168	KF807628	Sterrhinae	Idaea ochrata

LENOA806-11	LN-BD0813	KF807862	Sterrhinae	Idaea ochrata
GWORE479-08	BC ZSM Lep 12099	KF807837	Sterrhinae	Idaea ochrata
GWORC1157-08	BC ZSM Lep 12871	KF807613	Sterrhinae	Idaea ochrata
LEFID893-10	MM07212	HM873643	Sterrhinae	Idaea ochrata
GWOSO540-11	BC ZSM Lep 45146	KF807153	Sterrhinae	Idaea ochrata
GWORD616-08	BC ZSM Lep 14116	KF807385	Sterrhinae	Idaea ochrata
GWOSO496-11	BC ZSM Lep 45102	KF807514	Sterrhinae	Idaea ossiculata
GWORE478-08	BC ZSM Lep 12098	KF807193	Sterrhinae	Idaea ostrinaria
GWOSO528-11	BC ZSM Lep 45134	KF807895	Sterrhinae	Idaea ostrinaria
GWOTI774-12	BC ZSM SS Lep 0109	KF807307	Sterrhinae	Idaea ostrinaria
GWORD570-08	BC ZSM Lep 14070	KF807871	Sterrhinae	Idaea ostrinaria
GWORI449-09	BC ZSM Lep 12351	KF807821	Sterrhinae	Idaea ostrinaria
GWORI450-09	BC ZSM Lep 12352	KF807542	Sterrhinae	Idaea ostrinaria
GWOSO527-11	BC ZSM Lep 45133	KF807640	Sterrhinae	Idaea ostrinaria
GWORD569-08	BC ZSM Lep 14069	KF807399	Sterrhinae	Idaea ostrinaria
GWORD579-08	BC ZSM Lep 14079	KF807323	Sterrhinae	Idaea ostrinaria
GWORD568-08	BC ZSM Lep 14068	KF807239	Sterrhinae	Idaea ostrinaria
GWOSP888-11	BC MI 0127	KF807638	Sterrhinae	Idaea ostrinaria
GWORC1172-08	BC ZSM Lep 12886	KF807811	Sterrhinae	Idaea ostrinaria
GWORL676-09	BC ZSM Lep 23623	GU686693	Sterrhinae	Idaea palaestinensis
GWORD1100-08	BC ZSM Lep 01852	KF807748	Sterrhinae	Idaea pallidata
LEFIA280-10	MM01320	HM386624	Sterrhinae	Idaea pallidata
LEFIF735-10	MM12861	HM875419	Sterrhinae	Idaea pallidata
LEFIK840-10	MM18415	JN285638	Sterrhinae	Idaea pallidata
ODOPE648-11	BC ZSM Lep 51049	KF807956	Sterrhinae	Idaea pallidata
GWORC1169-08	BC ZSM Lep 12883	KF807768	Sterrhinae	Idaea pallidata
GWORL935-09	BC MI 0082	GU686572	Sterrhinae	Idaea politaria
GWOSP902-11	BC MI 0141	KF808129	Sterrhinae	Idaea politaria
GWORD674-08	BC ZSM Lep 14174	KF807384	Sterrhinae	Idaea politaria
GWORD675-08	BC ZSM Lep 14175	KF807249	Sterrhinae	Idaea politaria
GWORC1168-08	BC ZSM Lep 12882	KF807435	Sterrhinae	Idaea politaria
GWOSN330-11	BC ZSM Lep 46076	KF807105	Sterrhinae	Idaea politaria
GWORD673-08	BC ZSM Lep 14173	KF807211	Sterrhinae	Idaea politaria
GWORE1439-08	BC ZSM Lep 15032	KF807764	Sterrhinae	Idaea predotaria
GWOTF711-12	BC ZSM Lep 62417	KF807620	Sterrhinae	Idaea predotaria
GWORU566-10	BC NP 0468	JF848887	Sterrhinae	Idaea rainerii
GWORD626-08	BC ZSM Lep 14126	KF807100	Sterrhinae	Idaea rhodogrammaria
GWORD628-08	BC ZSM Lep 14128	KF807452	Sterrhinae	Idaea rhodogrammaria
GWORD627-08	BC ZSM Lep 14127	KF807074	Sterrhinae	Idaea rhodogrammaria
GWOTI824-12	BC ZSM SS Lep 0159	KF807755	Sterrhinae	Idaea rubraria
GWORB1717-08	BC ZSM Lep 12679	KF807233	Sterrhinae	Idaea rubraria
GWORB1629-08	BC ZSM Lep 12591	KF808049	Sterrhinae	Idaea rubraria
PHLAC562-10	TLMF Lep 02597	JF860131	Sterrhinae	Idaea rubraria
GWORB1627-08	BC ZSM Lep 12589	KF807324	Sterrhinae	Idaea rubraria
GWORB1626-08	BC ZSM Lep 12588	KF807183	Sterrhinae	Idaea rubraria
GWOSN717-11	BC ZSM Lep 52923	KF807120	Sterrhinae	Idaea rubraria
GWORB1631-08	BC ZSM Lep 12593	KF807523	Sterrhinae	Idaea rubraria
GWORB1718-08	BC ZSM Lep 12680	KF807191	Sterrhinae	Idaea rubraria
GWORB1628-08	BC ZSM Lep 12590	KF807276	Sterrhinae	Idaea rubraria
GWORL443-09	BC ZSM Lep 22345	GU686859	Sterrhinae	Idaea rubraria
GWORD1113-08	BC ZSM Lep 01865	KF807552	Sterrhinae	Idaea rufaria
GWORC1156-08	BC ZSM Lep 12870	KF807201	Sterrhinae	Idaea rufaria
GWOSO494-11	BC ZSM Lep 45100	KF807691	Sterrhinae	Idaea rufaria
FBLMZ331-12	BC ZSM Lep 64412	KF807349	Sterrhinae	Idaea rufaria
GWOTI793-12	BC ZSM SS Lep 0128	KF807959	Sterrhinae	Idaea rusticata
GWORB1773-08	BC ZSM Lep 12735	KF807164	Sterrhinae	Idaea rusticata

GWOTI796-12	BC ZSM SS Lep 0131	KF807616	Sterrhinae	Idaea rusticata
CGUKC223-09	UKLB24F01	KF807932	Sterrhinae	Idaea rusticata
CGUKC054-09	UKLB22G08	KF807809	Sterrhinae	Idaea rusticata
GWORL873-09	BC MI 0020	GU686629	Sterrhinae	Idaea rusticata
GWORB1772-08	BC ZSM Lep 12734	KF807122	Sterrhinae	Idaea rusticata
GWORD631-08	BC ZSM Lep 14131	KF807884	Sterrhinae	Idaea rusticata
PHLAC354-10	TLMF Lep 02389	KF807174	Sterrhinae	Idaea rusticata
CGUKC420-09	UKLB26F10	KF807634	Sterrhinae	Idaea rusticata
GWORL871-09	BC MI 0018	GU686635	Sterrhinae	Idaea rusticata
GWORM785-09	BC ZSM Lep 22745	GU687551	Sterrhinae	Idaea rusticata
GWORL872-09	BC MI 0019	GU686636	Sterrhinae	Idaea rusticata
GWORL874-09	BC MI 0021	GU686630	Sterrhinae	Idaea rusticata
CGUKB519-09	UKLB17B03	KF807673	Sterrhinae	Idaea rusticata
GWOTI795-12	BC ZSM SS Lep 0130	KF807293	Sterrhinae	Idaea rusticata
GWORD629-08	BC ZSM Lep 14129	KF807517	Sterrhinae	Idaea rusticata
GWORD630-08	BC ZSM Lep 14130	KF807131	Sterrhinae	Idaea rusticata
PHLAD746-11	TLMF Lep 04111	JN285753	Sterrhinae	Idaea rusticata
GWORB1771-08	BC ZSM Lep 12733	KF807241	Sterrhinae	Idaea rusticata
CGUKC592-09	UKLB28E06	KF807718	Sterrhinae	Idaea rusticata
GWOSI164-10	RCIM 0164	KF807564	Sterrhinae	Idaea rusticata
PHLSA472-11	TLMF Lep 05927	KF807611	Sterrhinae	Idaea rusticata
GWORL870-09	BC MI 0017	GU686634	Sterrhinae	Idaea rusticata
GWORI460-09	BC ZSM Lep 12362	KF807873	Sterrhinae	Idaea sardoniata
CGUKB957-09	UKLB21G06	KF807965	Sterrhinae	Idaea seriata
GWOTI813-12	BC ZSM SS Lep 0148	KF807543	Sterrhinae	Idaea seriata
GWORD586-08	BC ZSM Lep 14086	KF807479	Sterrhinae	Idaea seriata
GWOSO530-11	BC ZSM Lep 45136	KF807606	Sterrhinae	Idaea seriata
CGUKA716-09	UKLB8E12	KF807767	Sterrhinae	Idaea seriata
LEFID806-10	MM06892	HM873563	Sterrhinae	Idaea seriata
FBLMV434-09	BC ZSM Lep 28414	GU707367	Sterrhinae	Idaea seriata
NLLEA1089-12	RMNH.INS.539081	KF807260	Sterrhinae	Idaea seriata
GWORB1640-08	BC ZSM Lep 12602	KF807192	Sterrhinae	Idaea seriata
GWORB1732-08	BC ZSM Lep 12694	KF807480	Sterrhinae	Idaea seriata
LEFIK839-10	MM18414	JN285637	Sterrhinae	Idaea seriata
GWORB1643-08	BC ZSM Lep 12605	KF807978	Sterrhinae	Idaea seriata
CGUKA581-09	UKLB7B06	KF807904	Sterrhinae	Idaea seriata
GWORB1644-08	BC ZSM Lep 12606	KF807749	Sterrhinae	Idaea seriata
GWOTI812-12	BC ZSM SS Lep 0147	KF807833	Sterrhinae	Idaea seriata
CGUKA087-09	UKLB1H05	KF807743	Sterrhinae	Idaea seriata
GWORB1638-08	BC ZSM Lep 12600	KF807859	Sterrhinae	Idaea seriata
CGUKD361-09	UKLB36G01	KF807287	Sterrhinae	Idaea seriata
PHLAV372-12	TLMF Lep 08191	KF807175	Sterrhinae	Idaea seriata
GWORL945-09	BC MI 0092	GU686563	Sterrhinae	Idaea seriata
GWORB1731-08	BC ZSM Lep 12693	KF808067	Sterrhinae	Idaea seriata
GWORC058-07	BC ZSM Lep 01280	HQ601386	Sterrhinae	Idaea seriata
CGUKB078-09	UKLB12D09	KF807885	Sterrhinae	Idaea seriata
CGUKB755-09	UKLB19F04	KF807806	Sterrhinae	Idaea seriata
CGUKC524-09	UKLB27G08	KF807830	Sterrhinae	Idaea seriata
GWORB1729-08	BC ZSM Lep 12691	KF807486	Sterrhinae	Idaea seriata
GWORB3637-08	BC ZSM Lep add 0065	JF415304	Sterrhinae	Idaea seriata
LEFIE961-10	MM10367	HM874678	Sterrhinae	Idaea seriata
FBLMU102-09	BC ZSM Lep 25612	GU707199	Sterrhinae	Idaea seriata
GWORD712-08	BC ZSM Lep 14212	KF807498	Sterrhinae	Idaea seriata
GWOR4050-09	BC ZSM Lep 21334	HQ601385	Sterrhinae	Idaea seriata
GWORD713-08	BC ZSM Lep 14213	KF807575	Sterrhinae	Idaea seriata

GWORB1730-08	BC ZSM Lep 12692	KF807878	Sterrhinae	Idaea seriata
GWORD430-07	BC ZSM Lep 09580	KF807226	Sterrhinae	Idaea seriata
CGUKB433-09	UKLB16B11	KF807332	Sterrhinae	Idaea seriata
GWORM222-09	BC ZSM Lep 24271	GU687161	Sterrhinae	Idaea seriata
GWORB1639-08	BC ZSM Lep 12601	KF807593	Sterrhinae	Idaea seriata
GWOSI162-10	RCIM 0162	KF807709	Sterrhinae	Idaea seriata
GWORD1122-08	BC ZSM Lep 01874	KF808069	Sterrhinae	Idaea seriata
GWORB1733-08	BC ZSM Lep 12695	KF807080	Sterrhinae	Idaea seriata
GWORA951-08	BC ZSM Lep 02737	HQ601384	Sterrhinae	Idaea seriata
GWORA1682-08	BC ZSM Lep 16503	KF807359	Sterrhinae	Idaea sericeata
GWOTF699-12	BC ZSM Lep 62405	KF807356	Sterrhinae	Idaea serpentata
LEFID525-10	MM06505	HM873290	Sterrhinae	Idaea serpentata
PHLAH463-12	TLMF Lep 08282	KF807267	Sterrhinae	Idaea serpentata
GWORM044-09	BC ZSM Lep 24093	GU687298	Sterrhinae	Idaea serpentata
PHLAF434-11	TLMF Lep 05604	KF807725	Sterrhinae	Idaea serpentata
GWORD1117-08	BC ZSM Lep 01869	KF807977	Sterrhinae	Idaea serpentata
LEFIE955-10	MM10355	HM874672	Sterrhinae	Idaea serpentata
LEFIC003-10	MM03203	HM871873	Sterrhinae	Idaea serpentata
GWORD1118-08	BC ZSM Lep 01870	KF807182	Sterrhinae	Idaea serpentata
GWORD1119-08	BC ZSM Lep 01871	KF807184	Sterrhinae	Idaea serpentata
GWORM045-09	BC ZSM Lep 24094	GU687299	Sterrhinae	Idaea serpentata
GWORZ725-10	BC ZSM Lep 34976	HM914389	Sterrhinae	Idaea simplicior
GWORZ727-10	BC ZSM Lep 34978	HM914391	Sterrhinae	Idaea simplicior
GWORZ724-10	BC ZSM Lep 34975	HM914388	Sterrhinae	Idaea simplicior
GWOST096-11	BC ZSM Lep 45842	KF808152	Sterrhinae	Idaea simplicior
GWORZ726-10	BC ZSM Lep 34977	HM914390	Sterrhinae	Idaea simplicior
GWOSZ191-11	BC ZSM Lep 41947	KF807860	Sterrhinae	Idaea straminata
LEFIA326-10	MM01373	HM386669	Sterrhinae	Idaea straminata
GWORB1701-08	BC ZSM Lep 12663	KF807250	Sterrhinae	Idaea straminata
GWORC1178-08	BC ZSM Lep 12892	KF807664	Sterrhinae	Idaea straminata
GWORM051-09	BC ZSM Lep 24100	GU687295	Sterrhinae	Idaea straminata
GWORE1418-08	BC ZSM Lep 15011	KF807986	Sterrhinae	Idaea straminata
GWORM052-09	BC ZSM Lep 24101	GU687296	Sterrhinae	Idaea straminata
GWORG006-08	BC ZSM Lep 02826	GU655812	Sterrhinae	Idaea straminata
GWORE1440-08	BC ZSM Lep 15033	KF807747	Sterrhinae	Idaea straminata
LEFIF749-10	MM12944	HM875433	Sterrhinae	Idaea straminata
LEFIA325-10	MM01372	HM386668	Sterrhinae	Idaea straminata
CGUKB335-09	UKLB15B07	KF807275	Sterrhinae	Idaea straminata
PHLAE013-11	TLMF Lep 04233	JN285756	Sterrhinae	Idaea straminata
GWORL388-09	BC ZSM Lep 22290	GU686900	Sterrhinae	Idaea straminata
GWOTI832-12	BC ZSM SS Lep 0167	KF807460	Sterrhinae	Idaea straminata
GWORG007-08	BC ZSM Lep 02827	JF415305	Sterrhinae	Idaea straminata
LEFIE576-10	MM09472	HM874299	Sterrhinae	Idaea straminata
GWOSC935-10	BC ZSM Lep 41551	HQ958256	Sterrhinae	Idaea straminata
GWORD1997-08	BC ZSM Lep 17952	KF808025	Sterrhinae	Idaea straminata
PHLAA261-09	TLMF Lep 00301	HM425798	Sterrhinae	Idaea straminata
GWOSN333-11	BC ZSM Lep 46079	KF807432	Sterrhinae	Idaea subsaturata
CGUKB438-09	UKLB16C05	KF808061	Sterrhinae	Idaea subsericeata
NLLEA544-12	RMNH.INS.540736	KF807642	Sterrhinae	Idaea subsericeata
NLLEA1038-12	RMNH.INS.539029	KF808040	Sterrhinae	Idaea subsericeata
GWORC1165-08	BC ZSM Lep 12879	KF807163	Sterrhinae	Idaea subsericeata
GWOSI165-10	RCIM 0165	KF807945	Sterrhinae	Idaea subsericeata
GWORB1738-08	BC ZSM Lep 12700	KF807185	Sterrhinae	Idaea subsericeata
GWOTI780-12	BC ZSM SS Lep 0115	KF807400	Sterrhinae	Idaea subsericeata
GWORD593-08	BC ZSM Lep 14093	KF807544	Sterrhinae	Idaea subsericeata
GWORL877-09	BC MI 0024	GU686625	Sterrhinae	Idaea subsericeata

GWOTI777-12	BC ZSM SS Lep 0112	KF807508	Sterrhinae	Idaea subsericeata
GWOSO524-11	BC ZSM Lep 45130	KF807533	Sterrhinae	Idaea subsericeata
GWOTF310-12	BC ZSM Lep 60876	KF807980	Sterrhinae	Idaea subsericeata
GWOTI778-12	BC ZSM SS Lep 0113	KF808068	Sterrhinae	Idaea subsericeata
GWORB1739-08	BC ZSM Lep 12701	KF807658	Sterrhinae	Idaea subsericeata
GWORD595-08	BC ZSM Lep 14095	KF807600	Sterrhinae	Idaea subsericeata
GWORD621-08	BC ZSM Lep 14121	KF807829	Sterrhinae	Idaea subsericeata
GWORB1541-08	BC ZSM Lep 12503	KF807881	Sterrhinae	Idaea subsericeata
GWORL878-09	BC MI 0025	GU686626	Sterrhinae	Idaea subsericeata
GWORB1740-08	BC ZSM Lep 12702	KF807261	Sterrhinae	Idaea subsericeata
GWORD622-08	BC ZSM Lep 14122	KF807920	Sterrhinae	Idaea subsericeata
GWORL876-09	BC MI 0023	GU686632	Sterrhinae	Idaea subsericeata
GWOSN308-11	BC ZSM Lep 46054	KF807209	Sterrhinae	Idaea subsericeata
GWORD623-08	BC ZSM Lep 14123	KF807876	Sterrhinae	Idaea subsericeata
FBLMZ204-12	BC ZSM Lep 64285	KF807776	Sterrhinae	Idaea subsericeata
GWORD594-08	BC ZSM Lep 14094	KF807413	Sterrhinae	Idaea subsericeata
LENOA824-11	LN-BD0831	KF808138	Sterrhinae	Idaea subsericeata
PHLSA618-11	TLMF Lep 06073	KF807998	Sterrhinae	Idaea subsericeata
CGUKD351-09	UKLB36F03	KF808004	Sterrhinae	Idaea subsericeata
NLLEA1047-12	RMNH.INS.539038	KF807426	Sterrhinae	Idaea subsericeata
CGUKC240-09	UKLB24G06	KF807493	Sterrhinae	Idaea subsericeata
NLLEA481-12	RMNH.INS.540673	KF807983	Sterrhinae	Idaea subsericeata
CGUKB439-09	UKLB16C06	KF808073	Sterrhinae	Idaea subsericeata
CGUKB744-09	UKLB19E05	KF807927	Sterrhinae	Idaea subsericeata
NLLEA482-12	RMNH.INS.540674	KF807336	Sterrhinae	Idaea subsericeata
GWORL381-09	BC ZSM Lep 22283	HM393637	Sterrhinae	Idaea sylvestraria
CGUKC372-09	UKLB26B09	KF807952	Sterrhinae	Idaea sylvestraria
GWORL463-09	BC ZSM Lep 22365	HM393647	Sterrhinae	Idaea sylvestraria
GWORD1127-08	BC ZSM Lep 01879	KF807421	Sterrhinae	Idaea sylvestraria
GWORE2009-09	BC ZSM Lep 22407	HM393502	Sterrhinae	Idaea sylvestraria
GWORM047-09	BC ZSM Lep 24096	GU687300	Sterrhinae	Idaea sylvestraria
LEFIC719-10	MM04774	HM872540	Sterrhinae	Idaea sylvestraria
GWORD1128-08	BC ZSM Lep 01880	KF808145	Sterrhinae	Idaea sylvestraria
LEFIB054-10	MM00337	HM870963	Sterrhinae	Idaea sylvestraria
LENOA822-11	LN-BD0829	KF807967	Sterrhinae	Idaea sylvestraria
LEFID573-10	MM06568	HM873338	Sterrhinae	Idaea sylvestraria
LEFIF552-10	MM12251	HM875237	Sterrhinae	Idaea sylvestraria
GWORD1126-08	BC ZSM Lep 01878	KF807255	Sterrhinae	Idaea sylvestraria
GWORL390-09	BC ZSM Lep 22292	HM393640	Sterrhinae	Idaea sylvestraria
GWORE1493-08	BC ZSM Lep 15086	KF807668	Sterrhinae	Idaea textaria
GWORE1492-08	BC ZSM Lep 15085	KF808054	Sterrhinae	Idaea textaria
GWORE1494-08	BC ZSM Lep 15087	KF808027	Sterrhinae	Idaea textaria
GWOSR434-11	BC ZSM Lep 55490	KF807219	Sterrhinae	Idaea tineata
GWOSR435-11	BC ZSM Lep 55491	KF808108	Sterrhinae	Idaea tineata
GWORC1174-08	BC ZSM Lep 12888	KF807993	Sterrhinae	Idaea trigeminata
CGUKC1009-09	UKLB25G01	HM405725	Sterrhinae	Idaea trigeminata
CGUKB973-09	UKLB21H10	KF808060	Sterrhinae	Idaea trigeminata
GWOSP886-11	BC MI 0125	KF807651	Sterrhinae	Idaea trigeminata
GWORL881-09	BC MI 0028	GU686621	Sterrhinae	Idaea trigeminata
GWOSO532-11	BC ZSM Lep 45138	KF807831	Sterrhinae	Idaea trigeminata
CGUKA573-09	UKLB7A09	KF807128	Sterrhinae	Idaea trigeminata
CGUKC591-09	UKLB28E05	KF807492	Sterrhinae	Idaea trigeminata
CGUKB731-09	UKLB19D04	KF807713	Sterrhinae	Idaea trigeminata
GWOR4499-09	BC NP 0358	KF807491	Sterrhinae	Idaea trigeminata
GWOTI798-12	BC ZSM SS Lep 0133	KF807853	Sterrhinae	Idaea trigeminata
GWORE1489-08	BC ZSM Lep 15082	KF807835	Sterrhinae	Idaea troglodytaria

GWORE1453-08	BC ZSM Lep 15046	KF808094	Sterrhinae	Idaea typicata
GWOSQ543-11	BC ZSM Lep 57024	KF807741	Sterrhinae	Idaea typicata
PHLAC510-10	TLMF Lep 02545	JF860082	Sterrhinae	Idaea typicata
GWORC1173-08	BC ZSM Lep 12887	KF807274	Sterrhinae	Idaea typicata
GWOTI836-12	BC ZSM SS Lep 0171	KF807475	Sterrhinae	Idaea typicata
GWORI421-09	BC ZSM Lep 12323	KF807777	Sterrhinae	Idaea urcitana
GWOR3003-08	BC ZSM Lep+ 0015	KF807297	Sterrhinae	Idaea urcitana
GWORI409-09	BC ZSM Lep 12311	KF807825	Sterrhinae	Idaea urcitana
GWOR3004-08	BC ZSM Lep+ 0016	KF807448	Sterrhinae	Idaea urcitana
GWORU511-10	BC NP 0413	HM910648	Sterrhinae	Idaea vesubiata
PHLAF595-11	TLMF Lep 05765	KF807396	Geometrinae	Jodis lactearia
LENOA788-11	LN-BD0795	KF808051	Geometrinae	Jodis lactearia
LEFIA440-10	MM01504	HM386781	Geometrinae	Jodis lactearia
GWOR3795-09	BC ZSM Lep 21079	HQ601388	Geometrinae	Jodis lactearia
GWOTI892-12	BC ZSM SS Lep 0037	KF807115	Geometrinae	Jodis lactearia
GWORD1079-08	BC ZSM Lep 01831	KF807879	Geometrinae	Jodis lactearia
LENOA789-11	LN-BD0796	KF807913	Geometrinae	Jodis lactearia
GWOSO658-11	BC ZSM Lep 45549	KF807711	Geometrinae	Jodis lactearia
LEFIA441-10	MM01505	HM386782	Geometrinae	Jodis lactearia
LEFIG322-10	MM14292	HM876001	Geometrinae	Jodis lactearia
GWORD1077-08	BC ZSM Lep 01829	KF807540	Geometrinae	Jodis lactearia
GWOSR190-11	BC ZSM Lep 44986	KF807342	Geometrinae	Jodis lactearia
GWORL408-09	BC ZSM Lep 22310	GU686886	Geometrinae	Jodis lactearia
GWORA952-08	BC ZSM Lep 02738	HQ601387	Geometrinae	Jodis lactearia
CGUKA677-09	UKLB8B08	KF807216	Geometrinae	Jodis lactearia
GWORD1078-08	BC ZSM Lep 01830	KF807791	Geometrinae	Jodis lactearia
PHLAV157-12	TLMF Lep 07976	KF807766	Geometrinae	Jodis lactearia
MPSC024-11	RMNH.INS.27744	KF807411	Geometrinae	Jodis lactearia
LEFIA442-10	MM01506	HM386783	Geometrinae	Jodis putata
GWORD1082-08	BC ZSM Lep 01834	KF807075	Geometrinae	Jodis putata
LEFIA443-10	MM01507	HM386784	Geometrinae	Jodis putata
GWOSO567-11	BC ZSM Lep 45173	KF807818	Geometrinae	Jodis putata
FBLMV455-09	BC ZSM Lep 28435	HQ601389	Geometrinae	Jodis putata
GWORM030-09	BC ZSM Lep 24079	HM376818	Geometrinae	Jodis putata
GWORD1081-08	BC ZSM Lep 01833	KF807092	Geometrinae	Jodis putata
LEFIE054-10	MM08170	HQ570357	Geometrinae	Jodis putata
GWORB079-07	BC ZSM Lep 01113	KF807771	Geometrinae	Kuchleria insignata
GWORB078-07	BC ZSM Lep 01112	KF807477	Geometrinae	Kuchleria insignata
GWORE476-08	BC ZSM Lep 12096	KF807322	Geometrinae	Kuchleria insignata
GWORB075-07	BC ZSM Lep 01109	KF807402	Geometrinae	Kuchleria insignata
GWOSI823-10	BC ZSM Lep 49229	KF807911	Larentiinae	Lythria cruentaria
LEFIG383-10	MM14395	HM876060	Larentiinae	Lythria cruentaria
GWOTI948-12	BC ZSM SS Lep 0093	KF807746	Larentiinae	Lythria cruentaria
GWOTI949-12	BC ZSM SS Lep 0094	KF807667	Larentiinae	Lythria cruentaria
LEFIK842-10	MM18417	JN279402	Larentiinae	Lythria cruentaria
ODOPE650-11	BC ZSM Lep 51051	KF807738	Larentiinae	Lythria cruentaria
LEFIK843-10	MM18418	JN279403	Larentiinae	Lythria cruentaria
GWOTD299-12	BC ZSM Lep 63430	KF807671	Larentiinae	Lythria cruentaria
GWOSB286-10	BC ZSM Lep 34347	HQ957807	Larentiinae	Lythria plumularia
GWORU563-10	BC NP 0465	HM910683	Larentiinae	Lythria plumularia
GWOSB287-10	BC ZSM Lep 34348	HQ957808	Larentiinae	Lythria purpuraria
GWOTH295-12	BC ZSM Lep 65611	KF807312	Larentiinae	Lythria purpuraria
GWOTD298-12	BC ZSM Lep 63429	KF807750	Larentiinae	Lythria purpuraria
GWOSK926-11	BC ZSM Lep 49427	KF808098	Larentiinae	Lythria purpuraria
GWOSI821-10	BC ZSM Lep 49227	KF807285	Larentiinae	Lythria purpuraria
GWOTI761-12	BC ZSM SS Lep 0096	KF808023	Larentiinae	Lythria purpuraria

GWOTD1006-12	BMB Lep 00756	KF807166	Larentiinae	Lythria sanguinaria
GWOTD1007-12	BMB Lep 00757	KF807217	Larentiinae	Lythria sanguinaria
GWORB1695-08	BC ZSM Lep 12657	KF807496	Geometrinae	Microloxia herbaria
GWORD1989-08	BC ZSM Lep 17944	KF807644	Geometrinae	Microloxia herbaria
GWORD706-08	BC ZSM Lep 14206	KF807678	Geometrinae	Microloxia herbaria
GWORC408-07	BC ZSM Lep 06330	KF807955	Geometrinae	Microloxia herbaria
GWORL861-09	BC MI 0008	GU686641	Geometrinae	Microloxia herbaria
GWORL859-09	BC MI 0006	GU686647	Geometrinae	Microloxia herbaria
GWORL862-09	BC MI 0009	GU686642	Geometrinae	Microloxia herbaria
GWORL860-09	BC MI 0007	GU686648	Geometrinae	Microloxia herbaria
GWORD705-08	BC ZSM Lep 14205	KF807800	Geometrinae	Microloxia herbaria
GWORL858-09	BC MI 0005	GU686646	Geometrinae	Microloxia herbaria
GWORL857-09	BC MI 0004	GU686645	Geometrinae	Microloxia herbaria
GWORB262-07	BC ZSM Lep 02142	KF808002	Desmobathrinae	Myinodes constantina
GWORB263-07	BC ZSM Lep 02143	KF807805	Desmobathrinae	Myinodes constantina
GWORE1412-08	BC ZSM Lep 15005	KF808015	Desmobathrinae	Myinodes interpunctaria
GWORE485-08	BC ZSM Lep 12105	KF808124	Sterrhinae	Oar reaumuraria
GWORE486-08	BC ZSM Lep 12106	KF807213	Sterrhinae	Oar reaumuraria
GWORK654-09	BC ZSM Lep 21984	KF807186	Orthostixinae	Orthostixis cribraria
GWOSO377-11	BC ZSM Lep 44223	KF807902	Orthostixinae	Orthostixis cribraria
GWOTH287-12	BC ZSM Lep 65603	KF807456	Orthostixinae	Orthostixis cribraria
GWOSI069-10	RCIM 0069	KF807785	Geometrinae	Phaiogramma etruscaria
GWORD707-08	BC ZSM Lep 14207	KF807568	Geometrinae	Phaiogramma etruscaria
GWOTI888-12	BC ZSM SS Lep 0033	KF807559	Geometrinae	Phaiogramma etruscaria
GWORL863-09	BC MI 0010	GU686643	Geometrinae	Phaiogramma etruscaria
GWORB1680-08	BC ZSM Lep 12642	KF807984	Geometrinae	Phaiogramma etruscaria
GWORE475-08	BC ZSM Lep 12095	KF808085	Geometrinae	Phaiogramma etruscaria
GWOSP915-11	BC MI 0154	KF807824	Geometrinae	Phaiogramma etruscaria
GWOSN294-11	BC ZSM Lep 46040	KF807987	Geometrinae	Phaiogramma etruscaria
GWOSP916-11	BC MI 0155	KF807150	Geometrinae	Phaiogramma etruscaria
GWORL864-09	BC MI 0011	GU686644	Geometrinae	Phaiogramma etruscaria
GWORB1679-08	BC ZSM Lep 12641	KF808044	Geometrinae	Phaiogramma etruscaria
GWORB1678-08	BC ZSM Lep 12640	KF807587	Geometrinae	Phaiogramma etruscaria
GWOSN287-11	BC ZSM Lep 46033	KF808151	Geometrinae	Phaiogramma faustinata
GWOSR448-11	BC ZSM Lep 55504	KF807988	Geometrinae	Phaiogramma faustinata
GWOTI448-12	BC ZSM Lep 66714	KF807981	Geometrinae	Proteuchloris neriaria
GWOSR447-11	BC ZSM Lep 55503	KF808003	Geometrinae	Proteuchloris neriaria
GWORP972-09	BC ZSM Lep 26579	HM394301	Geometrinae	Pseudoterpna coronillaria
GWORC401-07	BC ZSM Lep 06323	KF807775	Geometrinae	Pseudoterpna coronillaria
GWORB149-07	BC ZSM Lep 07889	KF807503	Geometrinae	Pseudoterpna coronillaria
GWORC403-07	BC ZSM Lep 06325	KF807196	Geometrinae	Pseudoterpna coronillaria
GWOTI877-12	BC ZSM SS Lep 0022	KF807706	Geometrinae	Pseudoterpna coronillaria
GWOSF938-10	BC ZSM Lep 46969	KF807162	Geometrinae	Pseudoterpna coronillaria
GWORB1682-08	BC ZSM Lep 12644	KF807683	Geometrinae	Pseudoterpna coronillaria
GWOSO477-11	BC ZSM Lep 45083	KF807681	Geometrinae	Pseudoterpna coronillaria
GWORB1588-08	BC ZSM Lep 12550	KF807346	Geometrinae	Pseudoterpna coronillaria
GWORC399-07	BC ZSM Lep 06321	KF807501	Geometrinae	Pseudoterpna coronillaria
GWORC404-07	BC ZSM Lep 06326	KF807126	Geometrinae	Pseudoterpna coronillaria
GWORB1590-08	BC ZSM Lep 12552	KF807627	Geometrinae	Pseudoterpna coronillaria
GWORB1592-08	BC ZSM Lep 12554	KF808032	Geometrinae	Pseudoterpna coronillaria
GWORB1591-08	BC ZSM Lep 12553	KF807957	Geometrinae	Pseudoterpna coronillaria
GWOTH347-12	BC ZSM Lep 65663	KF807481	Geometrinae	Pseudoterpna coronillaria
GWOTI876-12	BC ZSM SS Lep 0021	KF807129	Geometrinae	Pseudoterpna coronillaria
GWORD618-08	BC ZSM Lep 14118	KF807393	Geometrinae	Pseudoterpna coronillaria
GWORC402-07	BC ZSM Lep 06324	KF807715	Geometrinae	Pseudoterpna coronillaria
PHLSA589-11	TLMF Lep 06044	KF807254	Geometrinae	Pseudoterpna coronillaria

GWORC398-07	BC ZSM Lep 06320	KF808125	Geometrinae	Pseudoterpna coronillaria
GWORD620-08	BC ZSM Lep 14120	KF807161	Geometrinae	Pseudoterpna coronillaria
GWORB3622-08	BC ZSM Lep add 0050	KF807874	Geometrinae	Pseudoterpna coronillaria
GWORB1681-08	BC ZSM Lep 12643	KF807910	Geometrinae	Pseudoterpna coronillaria
GWOTI875-12	BC ZSM SS Lep 0020	KF807798	Geometrinae	Pseudoterpna coronillaria
GWORB1589-08	BC ZSM Lep 12551	KF807938	Geometrinae	Pseudoterpna coronillaria
GWORB150-07	BC ZSM Lep 07890	KF807220	Geometrinae	Pseudoterpna coronillaria
GWORD619-08	BC ZSM Lep 14119	KF807308	Geometrinae	Pseudoterpna coronillaria
GWORU561-10	BC NP 0463	HM910681	Geometrinae	Pseudoterpna corsicaria
GWORU562-10	BC NP 0464	HM910682	Geometrinae	Pseudoterpna corsicaria
GWOSO378-11	BC ZSM Lep 44224	KF807548	Geometrinae	Pseudoterpna pruinata
LENOA777-11	LN-BD0784	KF807082	Geometrinae	Pseudoterpna pruinata
GWOTI873-12	BC ZSM SS Lep 0018	KF807439	Geometrinae	Pseudoterpna pruinata
GWORG011-08	BC ZSM Lep 02831	JF415306	Geometrinae	Pseudoterpna pruinata
GWOSO547-11	BC ZSM Lep 45153	KF807375	Geometrinae	Pseudoterpna pruinata
LENOA776-11	LN-BD0783	KF808121	Geometrinae	Pseudoterpna pruinata
PHLAC653-10	TLMF Lep 02688	JN820120	Geometrinae	Pseudoterpna pruinata
CGUKB186-09	UKLB13E11	KF807444	Geometrinae	Pseudoterpna pruinata
GWOSI062-10	RCIM 0062	KF807897	Geometrinae	Pseudoterpna pruinata
GWOSO380-11	BC ZSM Lep 44226	KF807949	Geometrinae	Pseudoterpna pruinata
GWOSO379-11	BC ZSM Lep 44225	KF808001	Geometrinae	Pseudoterpna pruinata
GWORB1531-08	BC ZSM Lep 12493	KF807240	Sterrhinae	Rhodometra sacraria
GWORB1782-08	BC ZSM Lep 12744	KF807172	Sterrhinae	Rhodometra sacraria
GWORL925-09	BC MI 0072	GU686578	Sterrhinae	Rhodometra sacraria
GWOTI947-12	BC ZSM SS Lep 0092	KF807563	Sterrhinae	Rhodometra sacraria
GWOSI087-10	RCIM 0087	KF807592	Sterrhinae	Rhodometra sacraria
GWOTH294-12	BC ZSM Lep 65610	KF807225	Sterrhinae	Rhodometra sacraria
GWORP841-09	BC ZSM Lep 19627	KF807602	Sterrhinae	Rhodometra sacraria
GWORB1784-08	BC ZSM Lep 12746	KF807403	Sterrhinae	Rhodometra sacraria
GWORD653-08	BC ZSM Lep 14153	KF807083	Sterrhinae	Rhodometra sacraria
LEFIA1338-10	MM00184	KF807828	Sterrhinae	Rhodometra sacraria
GWORD1998-08	BC ZSM Lep 17953	KF807291	Sterrhinae	Rhodometra sacraria
LEFIL156-10	MM19156	JF854500	Sterrhinae	Rhodometra sacraria
GWORB1530-08	BC ZSM Lep 12492	KF807546	Sterrhinae	Rhodometra sacraria
GWORD1999-08	BC ZSM Lep 17954	KF807997	Sterrhinae	Rhodometra sacraria
GWORM187-09	BC ZSM Lep 24236	GU687176	Sterrhinae	Rhodometra sacraria
LEFIL155-10	MM19155	KF808104	Sterrhinae	Rhodometra sacraria
GWORD652-08	BC ZSM Lep 14152	KF807882	Sterrhinae	Rhodometra sacraria
GWOTI945-12	BC ZSM SS Lep 0090	KF807521	Sterrhinae	Rhodometra sacraria
GWORD651-08	BC ZSM Lep 14151	KF807284	Sterrhinae	Rhodometra sacraria
GWOTF701-12	BC ZSM Lep 62407	KF714652	Sterrhinae	Rhodostrophia calabra
GWOSH381-10	BC ZSM Lep 39287	JF851392	Sterrhinae	Rhodostrophia calabra
GWORC1179-08	BC ZSM Lep 12893	KF714632	Sterrhinae	Rhodostrophia calabra
PHLAF577-11	TLMF Lep 05747	KF714617	Sterrhinae	Rhodostrophia calabra
GWOSI083-10	RCIM 0083	KF714607	Sterrhinae	Rhodostrophia calabra
GWOSH382-10	BC ZSM Lep 39288	JF851393	Sterrhinae	Rhodostrophia calabra
PHLAA655-09	TLMF Lep 00695	HM426056	Sterrhinae	Rhodostrophia calabra
GWORU515-10	BC NP 0417	JF848880	Sterrhinae	Rhodostrophia cretacaria
GWORM790-09	BC ZSM Lep 22750	HM422639	Sterrhinae	Rhodostrophia pudorata
GWOSP935-11	BC MI 0174	KF714568	Sterrhinae	Rhodostrophia pudorata
GWOSP875-11	BC MI 0114	KF714594	Sterrhinae	Rhodostrophia pudorata
GWORE1487-08	BC ZSM Lep 15080	KF714695	Sterrhinae	Rhodostrophia tabidaria
GWORD1604-08	BC ZSM Lep 16520	KF714688	Sterrhinae	Rhodostrophia vibicaria
GWORC1180-08	BC ZSM Lep 12894	KF714619	Sterrhinae	Rhodostrophia vibicaria
PHLSA604-11	TLMF Lep 06059	KF714570	Sterrhinae	Rhodostrophia vibicaria

NLLEA418-12	RMNH.INS.540610	KF714667	Sterrhinae	Rhodostrophia vibicaria
NLLEA411-12	RMNH.INS.540603	KF714641	Sterrhinae	Rhodostrophia vibicaria
LEFID748-10	MM06809	HM873505	Sterrhinae	Rhodostrophia vibicaria
PHLAF578-11	TLMF Lep 05748	KF714692	Sterrhinae	Rhodostrophia vibicaria
PHLAF241-11	TLMF Lep 05411	KF714708	Sterrhinae	Rhodostrophia vibicaria
GWORG008-08	BC ZSM Lep 02828	GU655813	Sterrhinae	Rhodostrophia vibicaria
GWORG009-08	BC ZSM Lep 02829	GU655814	Sterrhinae	Rhodostrophia vibicaria
LEFIJ135-10	MM09822	KF714648	Sterrhinae	Rhodostrophia vibicaria
LEFIJ132-10	MM09577	JF853450	Sterrhinae	Rhodostrophia vibicaria
LEFIJ168-10	MM10993	KF714620	Sterrhinae	Rhodostrophia vibicaria
GWORM053-09	BC ZSM Lep 24102	GU687297	Sterrhinae	Rhodostrophia vibicaria
GWOTI942-12	BC ZSM SS Lep 0087	KF714588	Sterrhinae	Rhodostrophia vibicaria
GWOTI941-12	BC ZSM SS Lep 0086	KF714602	Sterrhinae	Rhodostrophia vibicaria
GWORA1681-08	BC ZSM Lep 16502	KF714699	Sterrhinae	Rhodostrophia vibicaria
NLLEA419-12	RMNH.INS.540611	KF714647	Sterrhinae	Rhodostrophia vibicaria
LEFIF739-10	MM12877	HM875423	Sterrhinae	Rhodostrophia vibicaria
GWOSZ192-11	BC ZSM Lep 41948	KF714575	Sterrhinae	Rhodostrophia vibicaria
GWOTF309-12	BC ZSM Lep 60875	KF808005	Sterrhinae	Scopula alba
GWOR970-07	BC ZSM Lep 00970	KF807677	Sterrhinae	Scopula alba
GWORC1153-08	BC ZSM Lep 12867	KF807819	Sterrhinae	Scopula alba
GWOR990-07	BC ZSM Lep 00990	KF807982	Sterrhinae	Scopula alba
GWORB1789-08	BC ZSM Lep 12751	KF807922	Sterrhinae	Scopula alba
GWORD2017-08	BC ZSM Lep 17972	KF808079	Sterrhinae	Scopula alba
GWORB1788-08	BC ZSM Lep 12750	KF807098	Sterrhinae	Scopula alba
GWORB1787-08	BC ZSM Lep 12749	KF807463	Sterrhinae	Scopula alba
GWOR993-07	BC ZSM Lep 00993	KF808064	Sterrhinae	Scopula alba
GWORC1152-08	BC ZSM Lep 12866	KF807245	Sterrhinae	Scopula alba
GWORD2025-08	BC ZSM Lep 17980	KF807372	Sterrhinae	Scopula asellaria
GWORE1415-08	BC ZSM Lep 15008	KF808072	Sterrhinae	Scopula asellaria
GWORE1435-08	BC ZSM Lep 15028	KF807235	Sterrhinae	Scopula asellaria
GWORP759-09	BC ZSM Lep 19545	HM394192	Sterrhinae	Scopula asellaria
GWOST119-11	BC ZSM Lep 45865	KF807549	Sterrhinae	Scopula asellaria
GWOST115-11	BC ZSM Lep 45861	KF808140	Sterrhinae	Scopula caricaria
LEFIF023-10	MM10462	HM874737	Sterrhinae	Scopula caricaria
PHLAB336-10	TLMF Lep 01136	HQ968498	Sterrhinae	Scopula caricaria
LEFIK284-10	MM17859	KF808136	Sterrhinae	Scopula caricaria
LEFIK285-10	MM17860	KF807405	Sterrhinae	Scopula caricaria
GWORM219-09	BC ZSM Lep 24268	HM903332	Sterrhinae	Scopula caricaria
PHLAH707-12	TLMF Lep 08526	KF807801	Sterrhinae	Scopula caricaria
GWORA2074-09	BC ZSM Lep 26476	GU655383	Sterrhinae	Scopula confinaria
GWORC1154-08	BC ZSM Lep 12868	KF807964	Sterrhinae	Scopula confinaria
PHLSA615-11	TLMF Lep 06070	KF807395	Sterrhinae	Scopula confinaria
GWORK636-09	BC ZSM Lep 21966	KF807614	Sterrhinae	Scopula confinaria
GWORA2075-09	BC ZSM Lep 26477	GU655382	Sterrhinae	Scopula confinaria
GWOR969-07	BC ZSM Lep 00969	KF808146	Sterrhinae	Scopula confinaria
GWOSI082-10	RCIM 0082	KF808153	Sterrhinae	Scopula confinaria
GWOSO541-11	BC ZSM Lep 45147	KF807567	Sterrhinae	Scopula confinaria
GWORB077-07	BC ZSM Lep 01111	KF807572	Sterrhinae	Scopula confinaria
GWORM085-09	BC ZSM Lep 24134	GU687265	Sterrhinae	Scopula confinaria
GWORE1505-08	BC ZSM Lep 15098	KF807237	Sterrhinae	Scopula confinaria
GWOSO558-11	BC ZSM Lep 45164	KF807121	Sterrhinae	Scopula confinaria
GWOSV121-11	BC ZSM Lep 46152	KF807688	Sterrhinae	Scopula corrivalaria
LEFIJ803-10	MM17428	KF807457	Sterrhinae	Scopula corrivalaria
GWORI461-09	BC ZSM Lep 12363	KF807735	Sterrhinae	Scopula decolor
GWORC1148-08	BC ZSM Lep 12862	KF808139	Sterrhinae	Scopula decorata
GWOSO505-11	BC ZSM Lep 45111	KF807143	Sterrhinae	Scopula decorata

LEEUA509-11	MM20568	KF807238	Sterrhinae	Scopula decorata
GWOSI811-10	BC ZSM Lep 49217	JN285260	Sterrhinae	Scopula decorata
GWOTI917-12	BC ZSM SS Lep 0062	KF807840	Sterrhinae	Scopula decorata
LEFIL019-10	MM19019	JF854400	Sterrhinae	Scopula decorata
GWORB1556-08	BC ZSM Lep 12518	KF807522	Sterrhinae	Scopula decorata
GWORM199-09	BC ZSM Lep 24248	GU687174	Sterrhinae	Scopula decorata
LEFIL018-10	MM19018	JF854399	Sterrhinae	Scopula decorata
GWOSO506-11	BC ZSM Lep 45112	KF807123	Sterrhinae	Scopula decorata
LEFIL020-10	MM19020	KF807699	Sterrhinae	Scopula decorata
CGUKB161-09	UKLB13C10	KF807659	Sterrhinae	Scopula emutaria
LENOA843-11	LN-BD0850	KF807171	Sterrhinae	Scopula emutaria
LENOA841-11	LN-BD0848	KF807304	Sterrhinae	Scopula emutaria
GWOSO508-11	BC ZSM Lep 45114	KF807427	Sterrhinae	Scopula emutaria
GWOSV110-11	BC ZSM Lep 46141	KF807622	Sterrhinae	Scopula emutaria
GWOTI935-12	BC ZSM SS Lep 0080	KF807607	Sterrhinae	Scopula emutaria
LENOA842-11	LN-BD0849	KF807368	Sterrhinae	Scopula emutaria
GWORD1109-08	BC ZSM Lep 01861	KF807112	Sterrhinae	Scopula floslactata
LEFIA294-10	MM01338	HM386637	Sterrhinae	Scopula floslactata
PHLAC499-10	TLMF Lep 02534	JF860071	Sterrhinae	Scopula floslactata
GWORA1034-08	BC ZSM Lep 02820	HQ601531	Sterrhinae	Scopula floslactata
LEFIB855-10	MM02862	HM871732	Sterrhinae	Scopula floslactata
GWOR3814-09	BC ZSM Lep 21098	HQ601532	Sterrhinae	Scopula floslactata
PHLAV155-12	TLMF Lep 07974	KF807782	Sterrhinae	Scopula floslactata
FBLMT398-09	BC ZSM Lep 24958	HM391755	Sterrhinae	Scopula floslactata
CGUKA1018-09	UKLB8C10	HM405669	Sterrhinae	Scopula floslactata
LEFIA295-10	MM01339	HM386638	Sterrhinae	Scopula floslactata
GWORG102-08	BC ZSM Lep 03580	JF415307	Sterrhinae	Scopula floslactata
GWORG115-08	BC ZSM Lep 03593	JF415308	Sterrhinae	Scopula floslactata
FBLMV432-09	BC ZSM Lep 28412	GU707368	Sterrhinae	Scopula floslactata
CGUKA571-09	UKLB7A07	KF807160	Sterrhinae	Scopula floslactata
GWORD1111-08	BC ZSM Lep 01863	KF807227	Sterrhinae	Scopula floslactata
LEFIF021-10	MM10460	HM874735	Sterrhinae	Scopula frigidaria
LEFIF020-10	MM10459	HM874734	Sterrhinae	Scopula frigidaria
LEFIG933-10	MM15797	HM876575	Sterrhinae	Scopula frigidaria
GWORD672-08	BC ZSM Lep 14172	KF807446	Sterrhinae	Scopula imitaria
GWORB1790-08	BC ZSM Lep 12752	KF808128	Sterrhinae	Scopula imitaria
GWORB1792-08	BC ZSM Lep 12754	KF807487	Sterrhinae	Scopula imitaria
GWORB1597-08	BC ZSM Lep 12559	KF807861	Sterrhinae	Scopula imitaria
GWORD670-08	BC ZSM Lep 14170	KF807590	Sterrhinae	Scopula imitaria
GWORB1791-08	BC ZSM Lep 12753	KF807093	Sterrhinae	Scopula imitaria
CGUKD216-09	UKLB35B11	KF808106	Sterrhinae	Scopula imitaria
LENOA836-11	LN-BD0843	KF807221	Sterrhinae	Scopula imitaria
GWORD671-08	BC ZSM Lep 14171	KF808037	Sterrhinae	Scopula imitaria
CGUKA171-09	UKLB2G07	KF807845	Sterrhinae	Scopula imitaria
CGUKB523-09	UKLB17B07	KF807672	Sterrhinae	Scopula imitaria
GWORB1793-08	BC ZSM Lep 12755	KF807901	Sterrhinae	Scopula imitaria
CGUKC267-09	UKLB25A09	KF807626	Sterrhinae	Scopula imitaria
CGUKD427-09	UKLB37D08	KF807612	Sterrhinae	Scopula imitaria
NLLEA1037-12	RMNH.INS.539028	KF807144	Sterrhinae	Scopula imitaria
GWOSI081-10	RCIM 0081	KF807584	Sterrhinae	Scopula imitaria
CGUKB773-09	UKLB19G10	KF807355	Sterrhinae	Scopula imitaria
CGUKA601-09	UKLB7D03	KF807679	Sterrhinae	Scopula imitaria
GWORE477-08	BC ZSM Lep 12097	KF807724	Sterrhinae	Scopula imitaria
GWORB1598-08	BC ZSM Lep 12560	KF807925	Sterrhinae	Scopula imitaria
CGUKB524-09	UKLB17B08	KF807843	Sterrhinae	Scopula imitaria
LENOA837-11	LN-BD0844	KF807739	Sterrhinae	Scopula imitaria

GWOTI918-12	BC ZSM SS Lep 0063	KF808016	Sterrhinae	Scopula imitaria
CGUKA447-09	UKLB5G01	KF807547	Sterrhinae	Scopula imitaria
NLLEA560-12	RMNH.INS.540752	KF807700	Sterrhinae	Scopula imitaria
GWORM034-09	BC ZSM Lep 24083	GU687312	Sterrhinae	Scopula immorata
PHLAF353-11	TLMF Lep 05523	KF807138	Sterrhinae	Scopula immorata
GWORD1099-08	BC ZSM Lep 01851	KF807206	Sterrhinae	Scopula immorata
LEFIA150-10	MM01116	HM396496	Sterrhinae	Scopula immorata
LEFIA292-10	MM01336	HM386635	Sterrhinae	Scopula immorata
GWORM033-09	BC ZSM Lep 24082	GU687311	Sterrhinae	Scopula immorata
GWORC088-07	BC ZSM Lep 01310	HQ601533	Sterrhinae	Scopula immorata
LEFIA151-10	MM01117	HM396497	Sterrhinae	Scopula immorata
GWOTI921-12	BC ZSM SS Lep 0066	KF807387	Sterrhinae	Scopula immorata
LEFIA1356-10	MM00586	KF807310	Sterrhinae	Scopula immorata
GWORA1687-08	BC ZSM Lep 16508	KF807630	Sterrhinae	Scopula immorata
GWOSH470-10	BC ZSM Lep 39376	JF851449	Sterrhinae	Scopula immorata
GWORL436-09	BC ZSM Lep 22338	GU686860	Sterrhinae	Scopula immorata
GWORM050-09	BC ZSM Lep 24099	HQ564865	Sterrhinae	Scopula immutata
CGUKB481-09	UKLB16F12	KF807224	Sterrhinae	Scopula immutata
NLLEA821-12	RMNH.INS.541016	KF807450	Sterrhinae	Scopula immutata
CGUKB483-09	UKLB16G02	KF808150	Sterrhinae	Scopula immutata
LEFIA405-10	MM01464	HM386747	Sterrhinae	Scopula immutata
GWOSH467-10	BC ZSM Lep 39373	JF851446	Sterrhinae	Scopula immutata
CGUKC047-09	UKLB22G01	KF807458	Sterrhinae	Scopula immutata
GWORM040-09	BC ZSM Lep 24089	GU687302	Sterrhinae	Scopula immutata
GWORM218-09	BC ZSM Lep 24267	HM903331	Sterrhinae	Scopula immutata
CGUKD405-09	UKLB37B10	KF807106	Sterrhinae	Scopula immutata
LEFID524-10	MM06504	HM873289	Sterrhinae	Scopula immutata
NLLEA816-12	RMNH.INS.541011	KF807459	Sterrhinae	Scopula immutata
FBLMW370-10	BC ZSM Lep 37471	HQ563601	Sterrhinae	Scopula immutata
LEFIA404-10	MM01463	HM386746	Sterrhinae	Scopula immutata
PHLAH679-12	TLMF Lep 08498	KF807436	Sterrhinae	Scopula incanata
PHLAF313-11	TLMF Lep 05483	KF807961	Sterrhinae	Scopula incanata
GWOTG329-12	BC ZSM Lep 60705	KF807787	Sterrhinae	Scopula incanata
PHLSA455-11	TLMF Lep 05910	KF807582	Sterrhinae	Scopula incanata
GWOSI810-10	BC ZSM Lep 49216	JN285259	Sterrhinae	Scopula incanata
GWORA2608-09	BC ZSM Lep 31865	HQ957249	Sterrhinae	Scopula incanata
PHLAH809-12	TLMF Lep 08613	KF807586	Sterrhinae	Scopula incanata
PHLAB1163-10	TLMF Lep 01963	HQ968375	Sterrhinae	Scopula incanata
GWORA1677-08	BC ZSM Lep 16498	KF807256	Sterrhinae	Scopula incanata
LEFIF733-10	MM12852	HM875417	Sterrhinae	Scopula incanata
PHLAF251-11	TLMF Lep 05421	KF807155	Sterrhinae	Scopula incanata
GWOSI809-10	BC ZSM Lep 49215	KF807890	Sterrhinae	Scopula incanata
LEFIK838-10	MM18413	JN285636	Sterrhinae	Scopula incanata
PHLAA223-09	TLMF Lep 00263	HM381352	Sterrhinae	Scopula incanata
GWORM039-09	BC ZSM Lep 24088	GU687309	Sterrhinae	Scopula incanata
GWOTD390-12	BC ZSM Lep 63521	KF807478	Sterrhinae	Scopula incanata
GWORK544-09	BC ZSM Lep 21874	JF415309	Sterrhinae	Scopula incanata
PHLAC464-10	TLMF Lep 02499	JF860039	Sterrhinae	Scopula incanata
GWOTI914-12	BC ZSM SS Lep 0059	KF807948	Sterrhinae	Scopula incanata
GWORA1684-08	BC ZSM Lep 16505	KF807086	Sterrhinae	Scopula incanata
LEFIC624-10	MM04558	HM872445	Sterrhinae	Scopula incanata
GWORB1775-08	BC ZSM Lep 12737	KF807369	Sterrhinae	Scopula marginepunctata
LEFID223-10	MM06081	HM873024	Sterrhinae	Scopula marginepunctata
PHLAC511-10	TLMF Lep 02546	JF860083	Sterrhinae	Scopula marginepunctata
GWORA2104-09	BC ZSM Lep 26506	HM903259	Sterrhinae	Scopula marginepunctata
GWORD678-08	BC ZSM Lep 14178	KF807953	Sterrhinae	Scopula marginepunctata

GWORD1603-08	BC ZSM Lep 16519	KF807842	Sterrhinae	Scopula marginepunctata
LENOA834-11	LN-BD0841	KF807619	Sterrhinae	Scopula marginepunctata
GWOTH939-12	GF Lep 0084	KF807145	Sterrhinae	Scopula marginepunctata
LEFIK293-10	MM17868	KF807931	Sterrhinae	Scopula marginepunctata
PHLAV031-12	TLMF Lep 07850	KF807763	Sterrhinae	Scopula marginepunctata
GWORK580-09	BC ZSM Lep 21910	KF807769	Sterrhinae	Scopula marginepunctata
GWORI452-09	BC ZSM Lep 12354	KF807663	Sterrhinae	Scopula marginepunctata
FBLMV431-09	BC ZSM Lep 28411	HM902079	Sterrhinae	Scopula marginepunctata
LENOA833-11	LN-BD0840	KF807415	Sterrhinae	Scopula marginepunctata
GWORD676-08	BC ZSM Lep 14176	KF807553	Sterrhinae	Scopula marginepunctata
GWOTF311-12	BC ZSM Lep 60877	KF807794	Sterrhinae	Scopula marginepunctata
PHLSA474-11	TLMF Lep 05929	KF808144	Sterrhinae	Scopula marginepunctata
GWORB1776-08	BC ZSM Lep 12738	KF807761	Sterrhinae	Scopula marginepunctata
CGUKB738-09	UKLB19D11	KF807488	Sterrhinae	Scopula marginepunctata
NLLEA471-12	RMNH.INS.540663	KF807621	Sterrhinae	Scopula marginepunctata
NLLEA1189-12	RMNH.INS.544473	KF808070	Sterrhinae	Scopula marginepunctata
GWORL669-09	BC ZSM Lep 23616	GU686701	Sterrhinae	Scopula marginepunctata
GWOSI941-10	BC ZSM Lep 49347	KF807430	Sterrhinae	Scopula marginepunctata
CGUKD935-09	UKLB42G05	KF807826	Sterrhinae	Scopula marginepunctata
CGUKA182-09	UKLB2H06	KF807176	Sterrhinae	Scopula marginepunctata
CGUKA515-09	UKLB6D11	KF807702	Sterrhinae	Scopula marginepunctata
GWORD677-08	BC ZSM Lep 14177	KF807422	Sterrhinae	Scopula marginepunctata
GWORM038-09	BC ZSM Lep 24087	GU687308	Sterrhinae	Scopula marginepunctata
GWORB1635-08	BC ZSM Lep 12597	KF807525	Sterrhinae	Scopula marginepunctata
GWOSI080-10	RCIM 0080	KF807485	Sterrhinae	Scopula marginepunctata
GWOTH940-12	GF Lep 0085	KF807228	Sterrhinae	Scopula marginepunctata
GWORL913-09	BC MI 0060	GU686590	Sterrhinae	Scopula marginepunctata
GWORB1778-08	BC ZSM Lep 12740	KF807465	Sterrhinae	Scopula marginepunctata
GWORB1636-08	BC ZSM Lep 12598	KF807847	Sterrhinae	Scopula marginepunctata
GWORB1777-08	BC ZSM Lep 12739	KF807786	Sterrhinae	Scopula marginepunctata
GWOR971-07	BC ZSM Lep 00971	KF807929	Sterrhinae	Scopula mentzeri
GWORD667-08	BC ZSM Lep 14167	KF807471	Sterrhinae	Scopula minorata
GWORB1753-08	BC ZSM Lep 12715	KF807103	Sterrhinae	Scopula minorata
GWORD661-08	BC ZSM Lep 14161	KF807140	Sterrhinae	Scopula minorata
GWORL930-09	BC MI 0077	GU686575	Sterrhinae	Scopula minorata
GWORB1744-08	BC ZSM Lep 12706	KF807423	Sterrhinae	Scopula minorata
GWORB1752-08	BC ZSM Lep 12714	KF807370	Sterrhinae	Scopula minorata
GWOSP923-11	BC MI 0162	KF807560	Sterrhinae	Scopula minorata
GWORP993-09	BC ZSM Lep 26600	HM394319	Sterrhinae	Scopula minorata
GWORD668-08	BC ZSM Lep 14168	KF807424	Sterrhinae	Scopula minorata
GWORD660-08	BC ZSM Lep 14160	KF807705	Sterrhinae	Scopula minorata
GWOSP903-11	BC MI 0142	KF807125	Sterrhinae	Scopula minorata
GWORD669-08	BC ZSM Lep 14169	KF807104	Sterrhinae	Scopula minorata
GWORD659-08	BC ZSM Lep 14159	KF808028	Sterrhinae	Scopula minorata
GWORB1754-08	BC ZSM Lep 12716	KF807388	Sterrhinae	Scopula minorata
LEEUA516-11	MM20575	KF807538	Sterrhinae	Scopula nemoraria
GWORC093-07	BC ZSM Lep 01315	HQ601535	Sterrhinae	Scopula nigropunctata
LEEUA496-11	MM20555	KF807142	Sterrhinae	Scopula nigropunctata
LENOA827-11	LN-BD0834	KF807434	Sterrhinae	Scopula nigropunctata
GWORM036-09	BC ZSM Lep 24085	GU687306	Sterrhinae	Scopula nigropunctata
FBLMU105-09	BC ZSM Lep 25615	GU707200	Sterrhinae	Scopula nigropunctata
GWOR4063-09	BC ZSM Lep 21347	HQ601534	Sterrhinae	Scopula nigropunctata
GWORD1101-08	BC ZSM Lep 01853	KF808154	Sterrhinae	Scopula nigropunctata
GWOTI926-12	BC ZSM SS Lep 0071	KF807386	Sterrhinae	Scopula nigropunctata
LEEUA517-11	MM20576	KF807519	Sterrhinae	Scopula nigropunctata
FBLMZ139-12	BC ZSM Lep 51395	KF808046	Sterrhinae	Scopula nigropunctata

FBLMZ148-12	BC ZSM Lep 51404	KF807257	Sterrhinae	Scopula nigropunctata
GWOTI925-12	BC ZSM SS Lep 0070	KF807950	Sterrhinae	Scopula nigropunctata
GWORC1145-08	BC ZSM Lep 12859	KF807316	Sterrhinae	Scopula nigropunctata
FBLMZ121-12	BC ZSM Lep 51377	KF807381	Sterrhinae	Scopula nigropunctata
LENOA826-11	LN-BD0833	KF807685	Sterrhinae	Scopula nigropunctata
LEEUA492-11	MM20551	KF807483	Sterrhinae	Scopula ochraceata
GWOSO488-11	BC ZSM Lep 45094	KF807649	Sterrhinae	Scopula ochraceata
GWOR4140-09	BC ZSM Lep 21424	HQ601536	Sterrhinae	Scopula ornata
LEFIF022-10	MM10461	HM874736	Sterrhinae	Scopula ornata
GWORE2003-09	BC ZSM Lep 22401	HM393499	Sterrhinae	Scopula ornata
GWORB1554-08	BC ZSM Lep 12516	KF807633	Sterrhinae	Scopula ornata
GWORL372-09	BC ZSM Lep 22084	GU686911	Sterrhinae	Scopula ornata
PHLAF246-11	TLMF Lep 05416	KF807903	Sterrhinae	Scopula ornata
GWORB1553-08	BC ZSM Lep 12515	KF808090	Sterrhinae	Scopula ornata
GWORB1780-08	BC ZSM Lep 12742	KF807722	Sterrhinae	Scopula ornata
PHLAV329-12	TLMF Lep 08148	KF807279	Sterrhinae	Scopula ornata
GWORB1779-08	BC ZSM Lep 12741	KF807234	Sterrhinae	Scopula ornata
GWORD1103-08	BC ZSM Lep 01855	KF807289	Sterrhinae	Scopula ornata
GWORD601-08	BC ZSM Lep 14101	KF807792	Sterrhinae	Scopula ornata
LEFID886-10	MM07059	KF807391	Sterrhinae	Scopula ornata
GWORB1781-08	BC ZSM Lep 12743	KF807505	Sterrhinae	Scopula ornata
GWORD600-08	BC ZSM Lep 14100	KF807974	Sterrhinae	Scopula ornata
GWOR3796-09	BC ZSM Lep 21080	HQ601537	Sterrhinae	Scopula ornata
GWOSO507-11	BC ZSM Lep 45113	KF807734	Sterrhinae	Scopula ornata
GWORM037-09	BC ZSM Lep 24086	GU687307	Sterrhinae	Scopula ornata
GWORB1555-08	BC ZSM Lep 12517	KF807795	Sterrhinae	Scopula ornata
GWORD599-08	BC ZSM Lep 14099	KF807151	Sterrhinae	Scopula ornata
GWOSI078-10	RCIM 0078	KF808126	Sterrhinae	Scopula ornata
GWORL875-09	BC MI 0022	GU686631	Sterrhinae	Scopula ornata
FBLMW583-10	BC ZSM Lep 37969	HQ955631	Sterrhinae	Scopula rubiginata
GWOSO565-11	BC ZSM Lep 45171	KF807350	Sterrhinae	Scopula rubiginata
LEFIC343-10	MM03855	HQ570305	Sterrhinae	Scopula rubiginata
LEFIC342-10	MM03854	HM872186	Sterrhinae	Scopula rubiginata
GWOR4135-09	BC ZSM Lep 21419	HQ601539	Sterrhinae	Scopula rubiginata
LENOA832-11	LN-BD0839	KF807731	Sterrhinae	Scopula rubiginata
GWORC1146-08	BC ZSM Lep 12860	KF807089	Sterrhinae	Scopula rubiginata
GWORE2006-09	BC ZSM Lep 22404	HM393500	Sterrhinae	Scopula rubiginata
GWOR4138-09	BC ZSM Lep 21422	HQ601538	Sterrhinae	Scopula rubiginata
LEFIK837-10	MM18412	JN285635	Sterrhinae	Scopula rubiginata
NLLEA425-12	RMNH.INS.540617	KF807653	Sterrhinae	Scopula rubiginata
GWORM087-09	BC ZSM Lep 24136	GU687260	Sterrhinae	Scopula rubiginata
GWOSO564-11	BC ZSM Lep 45170	KF808017	Sterrhinae	Scopula rufomixtaria
GWOST163-11	BC ZSM Lep 45909	KF807686	Sterrhinae	Scopula rufomixtaria
GWORC1149-08	BC ZSM Lep 12863	KF807282	Sterrhinae	Scopula scalercii
GWOTI934-12	BC ZSM SS Lep 0079	KF807429	Sterrhinae	Scopula submutata
PHLAF231-11	TLMF Lep 05401	KF807596	Sterrhinae	Scopula submutata
PHLAF232-11	TLMF Lep 05402	KF807680	Sterrhinae	Scopula submutata
GWOSI079-10	RCIM 0079	KF807635	Sterrhinae	Scopula submutata
GWORB1785-08	BC ZSM Lep 12747	KF807892	Sterrhinae	Scopula submutata
PHLAC508-10	TLMF Lep 02543	JF860080	Sterrhinae	Scopula submutata
GWOSO487-11	BC ZSM Lep 45093	KF807790	Sterrhinae	Scopula submutata
LEEUA494-11	MM20553	KF807855	Sterrhinae	Scopula submutata
GWORI456-09	BC ZSM Lep 12358	KF807327	Sterrhinae	Scopula submutata
GWORB1786-08	BC ZSM Lep 12748	KF807914	Sterrhinae	Scopula submutata
LENOA840-11	LN-BD0847	KF808083	Sterrhinae	Scopula subpunctaria
FBLMZ541-12	BC ZSM Lep 61297	KF807130	Sterrhinae	Scopula subpunctaria

GWOTI920-12	BC ZSM SS Lep 0065	KF807697	Sterrhinae	Scopula subpunctaria
GWORM042-09	BC ZSM Lep 24091	GU687304	Sterrhinae	Scopula subpunctaria
GWOSV111-11	BC ZSM Lep 46142	KF807788	Sterrhinae	Scopula subpunctaria
LENOA839-11	LN-BD0846	KF807562	Sterrhinae	Scopula subpunctaria
GWORD1112-08	BC ZSM Lep 01864	KF807328	Sterrhinae	Scopula subpunctaria
LEFIA388-10	MM01445	HM386730	Sterrhinae	Scopula ternata
PHLAC452-10	TLMF Lep 02487	JF860027	Sterrhinae	Scopula ternata
GWOSO483-11	BC ZSM Lep 45089	KF807784	Sterrhinae	Scopula ternata
LEFIE179-10	MM08463	HM873925	Sterrhinae	Scopula ternata
GWOSI920-10	BC ZSM Lep 49326	JN285268	Sterrhinae	Scopula ternata
LEFIA387-10	MM01444	HM386729	Sterrhinae	Scopula ternata
PHLAA274-09	TLMF Lep 00314	HM425810	Sterrhinae	Scopula ternata
GWORM041-09	BC ZSM Lep 24090	GU687303	Sterrhinae	Scopula ternata
GWORM214-09	BC ZSM Lep 24263	GU687164	Sterrhinae	Scopula tessellaria
GWORC1143-08	BC ZSM Lep 12857	KF807404	Sterrhinae	Scopula tessellaria
GWOSI077-10	RCIM 0077	KF807813	Sterrhinae	Scopula tessellaria
GWOTI912-12	BC ZSM SS Lep 0057	KF808000	Sterrhinae	Scopula tessellaria
GWOSO548-11	BC ZSM Lep 45154	KF807363	Sterrhinae	Scopula tessellaria
GWORK635-09	BC ZSM Lep 21965	KF807232	Sterrhinae	Scopula tessellaria
GWOSO559-11	BC ZSM Lep 45165	KF807189	Sterrhinae	Scopula turbulentaria
GWORC1147-08	BC ZSM Lep 12861	KF807935	Sterrhinae	Scopula turbulentaria
GWORM035-09	BC ZSM Lep 24084	GU687313	Sterrhinae	Scopula umbelaria
PHLAV026-12	TLMF Lep 07845	KF807158	Sterrhinae	Scopula umbelaria
GWORB1587-08	BC ZSM Lep 12549	KF807453	Sterrhinae	Scopula vigilata
GWORL882-09	BC MI 0029	GU686622	Sterrhinae	Scopula vigilata
GWORB1763-08	BC ZSM Lep 12725	KF808045	Sterrhinae	Scopula vigilata
GWOTI937-12	BC ZSM SS Lep 0082	KF808055	Sterrhinae	Scopula vigilata
GWOSO486-11	BC ZSM Lep 45092	KF807694	Sterrhinae	Scopula vigilata
GWORB1764-08	BC ZSM Lep 12726	KF807625	Sterrhinae	Scopula vigilata
GWOR5190-09	BC ZSM Lep 22480	GU655171	Sterrhinae	Scopula vigilata
GWORB1586-08	BC ZSM Lep 12548	KF807598	Sterrhinae	Scopula vigilata
GWORL883-09	BC MI 0030	GU686623	Sterrhinae	Scopula vigilata
GWOTI936-12	BC ZSM SS Lep 0081	KF807804	Sterrhinae	Scopula vigilata
GWORB1585-08	BC ZSM Lep 12547	KF807954	Sterrhinae	Scopula vigilata
GWORL682-09	BC ZSM Lep 23629	GU686691	Sterrhinae	Scopula vigilata
GWORL946-09	BC MI 0093	GU686564	Sterrhinae	Scopula vigilata
GWOSO552-11	BC ZSM Lep 45158	KF807720	Sterrhinae	Scopula vigilata
GWORB1762-08	BC ZSM Lep 12724	KF807531	Sterrhinae	Scopula vigilata
GWORA2084-09	BC ZSM Lep 26486	HM903252	Sterrhinae	Scopula virgulata
LEFIJ804-10	MM17429	JF853833	Sterrhinae	Scopula virgulata
PHLAV309-12	TLMF Lep 08128	KF807655	Sterrhinae	Scopula virgulata
LEFIL397-10	MM18695	JF854582	Sterrhinae	Scopula virgulata
LEFIL398-10	MM18696	JF854583	Sterrhinae	Scopula virgulata
GWOTD391-12	BC ZSM Lep 63522	KF807152	Geometrinae	Thalera fimbrialis
LEFIE440-10	MM09202	HM874164	Geometrinae	Thalera fimbrialis
GWOSO512-11	BC ZSM Lep 45118	KF807252	Geometrinae	Thalera fimbrialis
GWORZ712-10	BC ZSM Lep 34963	HM914379	Geometrinae	Thalera fimbrialis
LENOA787-11	LN-BD0794	KF807960	Geometrinae	Thalera fimbrialis
GWORZ711-10	BC ZSM Lep 34962	HM914378	Geometrinae	Thalera fimbrialis
GWOTI889-12	BC ZSM SS Lep 0034	KF807108	Geometrinae	Thalera fimbrialis
PHLSA639-11	TLMF Lep 06094	KF807136	Geometrinae	Thalera fimbrialis
GWOR4141-09	BC ZSM Lep 21425	HQ601550	Geometrinae	Thalera fimbrialis
LEFIF367-10	MM11573	HM875052	Geometrinae	Thalera fimbrialis
LENOA786-11	LN-BD0793	KF807639	Geometrinae	Thalera fimbrialis
LEFIF368-10	MM11575	HM875053	Geometrinae	Thalera fimbrialis
GWORZ713-10	BC ZSM Lep 34964	HM914380	Geometrinae	Thalera fimbrialis

GWOTI890-12	BC ZSM SS Lep 0035	KF807280	Geometrinae	Thalera fimbrialis
GWORG024-08	BC ZSM Lep 02844	GU655822	Geometrinae	Thalera fimbrialis
GWORG023-08	BC ZSM Lep 02843	GU655821	Geometrinae	Thalera fimbrialis
GWORU517-10	BC NP 0419	JF848881	Geometrinae	Thetidia sardinica
PHLAF303-11	TLMF Lep 05473	KF807588	Geometrinae	Thetidia smaragdaria
GWORP010-09	BC PG Lep 0010	HM393745	Geometrinae	Thetidia smaragdaria
GWORG019-08	BC ZSM Lep 02839	JF415310	Geometrinae	Thetidia smaragdaria
GWORL936-09	BC MI 0083	GU686573	Geometrinae	Thetidia smaragdaria
GWORG018-08	BC ZSM Lep 02838	GU655819	Geometrinae	Thetidia smaragdaria
GWOTI882-12	BC ZSM SS Lep 0027	KF807995	Geometrinae	Thetidia smaragdaria
GWORC406-07	BC ZSM Lep 06328	KF807281	Geometrinae	Thetidia smaragdaria
GWORC405-07	BC ZSM Lep 06327	KF807554	Geometrinae	Thetidia smaragdaria
GWOTI883-12	BC ZSM SS Lep 0028	KF807078	Geometrinae	Thetidia smaragdaria
FBLMZ143-12	BC ZSM Lep 51399	KF807716	Geometrinae	Thetidia smaragdaria
GWOSI063-10	RCIM 0063	KF807159	Geometrinae	Thetidia smaragdaria
GWOSO515-11	BC ZSM Lep 45121	KF807338	Geometrinae	Thetidia smaragdaria
LEFIA254-10	MM01291	HM386598	Geometrinae	Thetidia smaragdaria
LEFIA253-10	MM01290	HM386597	Geometrinae	Thetidia smaragdaria
LEFIA332-10	MM01379	HM386675	Geometrinae	Thetidia smaragdaria
GWORG020-08	BC ZSM Lep 02840	JF415311	Geometrinae	Thetidia smaragdaria
GWORL937-09	BC MI 0084	GU686567	Geometrinae	Thetidia smaragdaria
GWOSO514-11	BC ZSM Lep 45120	KF808088	Geometrinae	Thetidia smaragdaria
LEFIG352-10	MM14349	HM876029	Geometrinae	Thetidia smaragdaria
FBLMT362-09	BC ZSM Lep 24922	HM391754	Sterrhinae	Timandra comae
CGUKD208-09	UKLB35B03	KF808131	Sterrhinae	Timandra comae
GWOTH382-12	BC ZSM Lep 66363	KF807660	Sterrhinae	Timandra comae
PHLAC727-10	TLMF Lep 02762	JF860279	Sterrhinae	Timandra comae
GWORC057-07	BC ZSM Lep 01279	HQ601576	Sterrhinae	Timandra comae
GWORC1142-08	BC ZSM Lep 12856	KF807454	Sterrhinae	Timandra comae
NLLEA163-12	RMNH.INS.538778	KF808130	Sterrhinae	Timandra comae
CGUKA057-09	UKLB1E11	KF807361	Sterrhinae	Timandra comae
GWOTI911-12	BC ZSM SS Lep 0056	KF807490	Sterrhinae	Timandra comae
FBLMX142-11	BC ZSM Lep 37623	JN285192	Sterrhinae	Timandra comae
NLLEA434-12	RMNH.INS.540626	KF807643	Sterrhinae	Timandra comae
GWORD690-08	BC ZSM Lep 14190	KF807574	Sterrhinae	Timandra comae
CGUKB492-09	UKLB16G11	KF808133	Sterrhinae	Timandra comae
CGUKD267-09	UKLB35G02	KF807318	Sterrhinae	Timandra comae
GWORD691-08	BC ZSM Lep 14191	KF807509	Sterrhinae	Timandra comae
GWOR4057-09	BC ZSM Lep 21341	HQ601575	Sterrhinae	Timandra comae
NLLEA121-12	RMNH.INS.538736	KF807494	Sterrhinae	Timandra comae
GWORD689-08	BC ZSM Lep 14189	KF807331	Sterrhinae	Timandra comae
CGUKB980-09	UKLB22A05	KF807379	Sterrhinae	Timandra comae
CGUKA127-09	UKLB2C11	KF807294	Sterrhinae	Timandra comae
GWORC050-07	BC ZSM Lep 01272	HQ601577	Sterrhinae	Timandra comae
FBLMW358-10	BC ZSM Lep 37459	HQ563589	Sterrhinae	Timandra comae
GWOR4126-09	BC ZSM Lep 21410	HQ601574	Sterrhinae	Timandra comae
CGUKC343-09	UKLB25H03	KF807420	Sterrhinae	Timandra comae
CGUKB098-09	UKLB12F05	KF807693	Sterrhinae	Timandra comae
CGUKA398-09	UKLB5B11	KF807636	Sterrhinae	Timandra comae
LEFIC730-10	MM04806	HM872551	Sterrhinae	Timandra comae
LEFIA433-10	MM01496	HM386774	Sterrhinae	Timandra comae
LEFIC677-10	MM04645	HM872498	Sterrhinae	Timandra comae
LEFIC737-10	MM04838	HM872558	Sterrhinae	Timandra comae
CGUKC119-09	UKLB23E03	KF808084	Sterrhinae	Timandra comae
LEFIF721-10	MM12784	HM875405	Sterrhinae	Timandra comae
LEFIA315-10	MM01361	HM386658	Sterrhinae	Timandra comae

LEFIF722-10	MM12785	HM875406	Sterrhinae	Timandra comae
PHLAH726-12	TLMF Lep 08545	KF807555	Sterrhinae	Timandra comae
CGUKC442-09	UKLB26H08	KF807188	Sterrhinae	Timandra comae
LEFIJ1134-11	MM21288	KF807703	Sterrhinae	Timandra comae
GWORB799-07	BC ZSM Lep 01175	HQ601578	Sterrhinae	Timandra comae
GWOSI181-10	RCIM 0181	KF807320	Sterrhinae	Timandra comae
LEFIJ1135-11	MM21289	KF807230	Sterrhinae	Timandra comae
GWOTH293-12	BC ZSM Lep 65609	KF807744	Sterrhinae	Timandra comae
LEFIF741-10	MM12899	HM875425	Sterrhinae	Timandra comae
LEFIA293-10	MM01337	HM386636	Sterrhinae	Timandra griseata
LEFIA432-10	MM01495	HM386773	Sterrhinae	Timandra griseata
LEFIF386-10	MM11653	HM875071	Sterrhinae	Timandra griseata
LEFIB848-10	MM02839	HM871725	Sterrhinae	Timandra griseata
LEFIC701-10	MM04701	HM872522	Sterrhinae	Timandra griseata
LEFIA316-10	MM01362	HM386659	Sterrhinae	Timandra griseata
LEFIC696-10	MM04684	HM872517	Sterrhinae	Timandra griseata
LEFIA399-10	MM01458	HM386741	Sterrhinae	Timandra griseata
LEFIF723-10	MM12786	HM875407	Sterrhinae	Timandra griseata
LEFIG477-10	MM14528	HM876153	Sterrhinae	Timandra griseata
LEFID958-10	MM07916	HM873708	Sterrhinae	Timandra griseata
GWORB1675-08	BC ZSM Lep 12637	KF808059	Geometrinae	Xenochlorodes olympiaria
GWORB1676-08	BC ZSM Lep 12638	KF807470	Geometrinae	Xenochlorodes olympiaria
GWOSI065-10	RCIM 0065	KF807169	Geometrinae	Xenochlorodes olympiaria
GWOSI925-10	BC ZSM Lep 49331	JN271376	Geometrinae	Xenochlorodes olympiaria
GWOSO517-11	BC ZSM Lep 45123	KF807264	Geometrinae	Xenochlorodes olympiaria
GWOTI894-12	BC ZSM SS Lep 0039	KF808071	Geometrinae	Xenochlorodes olympiaria
