# Supplementary material for: Genetic Patterns in European Geometrid Moths Revealed by the Barcode Index Number (BIN) System
Source: PLoS One. 2013 Dec 17;8(12):e84518. doi: 10.1371/journal.pone.0084518 (PMC3866169; doi:10.1371/journal.pone.0084518)
Supplement: Appendix S3 — List of European geometrid species without BIN. List of 34 European taxa without a BIN assignment (awaiting DNA barcoding); four species with short sequences are marked with an asterisk. 30 species are completely missing (12%), whilst 88% species of the five examined European geometrid subfamilies are represented by COI sequences. (PDF) [file pone.0084518.s003.pdf]

Appendix S3: List of European geometrid species without BIN

List of 34 European taxa without a BIN assignment (awaiting DNA barcoding); four species with short sequences are marked with an asterisk. 30 species are completely missing (12%), whilst 88% species of the five examined European geometrid subfamilies are represented by COI sequences.

Archiearinae

Leucobrephos middendorfii

Geometrinae

Heliothea discoidaria *
Comibaena pseudoneriaria
*Thetidia plusiaria**
Bustilloxia saturata

Sterrhinae

Anthometra plumularia
Cleta ramosaria
Cleta perpusillaria
Idaea luteolaria
Idaea lusohispanica
Idaea consanguiberica
*Idaea macilentaria**
Idaea nevadata
Idaea spissilimbaria
Idaea robiginata
Idaea lutulentaria
Idaea nexata
Idaea descitaria
Idaea acutipennis
Idaea rupicolaria
Idaea saleri
*Idaea nitidata**
Idaea dromikos
Idaea squalidaria
Limeria macraria
Scopula concinnaria
Scopula honestata
Scopula rubellata
Scopula cajanderi
Scopula divisaria
Scopula rufinaria
Timandra rectistrigaria
Cyclophora serveti
Cyclophora hyponoea
