# Supplementary material for: Genetic Patterns in European Geometrid Moths Revealed by the Barcode Index Number (BIN) System
Source: PLoS One. 2013 Dec 17;8(12):e84518. doi: 10.1371/journal.pone.0084518 (PMC3866169; doi:10.1371/journal.pone.0084518)
Supplement: Appendix S4 — Species with BIN-Splits. Intraspecific genetic divergences (in % minimum pairwise distance, Kimura 2 parameter) and shortest geographic distance (in km) between representatives of each BIN; n = number of European BIN-representatives (barcoded >500 bp), ‘1+’ refers to BINs with a singleton in Europe, but with additional representatives outside Europe; in species with multiple BIN-splits all possible combinations were included producing a total of 93 comparisons. (PDF) [file pone.0084518.s004.pdf]

Appendix S4: Species with BIN-Splits

Intraspecific genetic divergences (in % minimum pairwise distance, Kimura 2 parameter) and shortest geographic distance (in km) between representatives of each BIN; n = number of European BIN-representatives (barcoded >500 bp), '1+' refers to BINS with a singleton in Europe, but with additional representatives outside Europe; in species with multiple BIN-splits all possible combinations were included producing a total of 93 comparisons.

species	shortest distance	genetic divergence	n cluster 1	n cluster 2
A. parthenias	130 km	1.2 %	7	5
O. cribraria	920 km	3.0 %	2	1+
A. ononaria	1640 km	4.0 %	2	2
P. pruinata	20 km	4.1 %	10	1
P. coronillaria 1-2	1560 km	2.4 %	7	5
P. coronillaria 1-3	160 km	2.4 %	7	11
P. coronillaria 1-4	540 km	3.8 %	7	4
P. coronillaria 2-3	970 km	1.4 %	5	11
P. coronillaria 2-4	1040 km	4.0 %	5	4
P. coronillaria 3-4	370 km	3.8 %	11	4
H. chrysoprasaria	1600 km	3.0 %	12	2
X. olympiaria 1-2	530 km	3.3 %	2	2
X. olympiaria 1-3	690 km	4.3 %	2	2
X. olympiaria 2-3	360 km	1.7 %	2	2
J. putata	70 km	2.0 %	6	2
E. indigenata	10 km	3.7 %	8	1
T. fimbrialis	330 km	3.5 %	15	1+
H. aestivaria	0 km	2.5 %	18	3
M. herbaria	520 km	1.9 %	9	2
I. consanguinaria	590 km	1.9 %	6	1+
I. ochrata	1230 km	1.6 %	13	3
I. alyssumata 1-2	630 km	4.3 %	5	6
I. alyssumata 1-3	550 km	1.6 %	5	2
I. alyssumata 1-4	940 km	2.0 %	5	2
I. alyssumata 1-5	940 km	1.9 %	5	3
I. alyssumata 1-6	250 km	2.3 %	5	1
I. alyssumata 2-3	600 km	4.3 %	6	2
I. alyssumata 2-4	900 km	4.3 %	6	2
I. alyssumata 2-5	900 km	3.5 %	6	3
I. alyssumata 2-6	680 km	4.2 %	6	1
I. alyssumata 3-4	220 km	1.4 %	2	2
I. alyssumata 3-5	220 km	1.6 %	2	3
I. alyssumata 3-6	590 km	2.3 %	2	1
I. alyssumata 4-5	0 km	2.0 %	2	3
I. alyssumata 4-6	800 km	2.7 %	2	1
I. alyssumata 5-6	740 km	2.3 %	3	1
I. calunetaria	360 km	3.6 %	2	1
I. elongaria 1-2	290 km	2.5 %	7	1
I. elongaria 1-3	610 km	3.0 %	7	1
I. elongaria 1-4	60 km	6.8 %	7	1+
I. elongaria 2-3	2460 km	2.7 %	1	1
I. elongaria 2-4	1890 km	6.8 %	1	1+
I. elongaria 3-4	520 km	6.3 %	1	1+
I. obsoletaria 1-2	820 km	1.2 %	9	7
I. obsoletaria 1-3	820 km	2.1 %	9	1

I. obsoletaria 1-4	220 km	2.2 %	9	5
I. obsoletaria 2-3	0 km	3.1 %	7	1
I. obsoletaria 2-4	1330 km	2.7 %	7	5
I. obsoletaria 3-4	1330 km	2.9 %	1	5
I. fuscovenosa 1-2	40 km	2.3 %	13	5
I. fuscovenosa 1-3	970 km	3.0 %	13	2
I. fuscovenosa 1-4	1140 km	2.7 %	13	1
I. fuscovenosa 2-3	990 km	1.7 %	5	2
I. fuscovenosa 2-4	1170 km	2.2 %	5	1
I. fuscovenosa 3-4	20 km	2.0 %	2	1
I. humiliata	1050 km	1.9 %	13	2
I. longaria 1-2	1560 km	1.6 %	4	4
I. longaria 1-3	1100 km	7.3 %	4	1
I. longaria 1-4	2110 km	2.0 %	4	2
I. longaria 2-3	590 km	7.0 %	4	1
I. longaria 2-4	630 km	2.0 %	4	2
I. longaria 3-4	1010 km	6.5 %	1	2
I. seriata 1-2	1090 km	1.2 %	15	6
I. seriata 1-3	520 km	2.4 %	15	4
I. seriata 1-4	260 km	3.0 %	15	16
I. seriata 2-3	550 km	2.0 %	6	4
I. seriata 2-4	90 km	2.5 %	6	16
I. seriata 3-4	100 km	2.8 %	4	16
I. subsericeata	360 km	1.7 %	28	6
I. trigeminata 1-2	580 km	5.9 %	9	1+
I. trigeminata 1-3	620 km	6.6 %	9	1
I. trigeminata 2-3	180 km	3.3 %	1+	1
I. fractilineata	1340 km	3.3 %	3	4
I. infirmaria 1-2	480 km	2.5 %	7	5
I. infirmaria 1-3	480 km	2.2 %	7	3
I. infirmaria 2-3	0 km	3.3 %	5	3
I. ostrinaria	0 km	3.8 %	10	2
S. virgulata	670 km	2.7 %	4	1
S. ornata 1-2	520 km	4.9 %	18	3
S. ornata 1-3	0 km	3.3 %	18	2
S. ornata 2-3	520 km	4.5 %	3	2
S. submutata	1030 km	4.2 %	9	1+
S. incanata 1-2	0 km	1.9 %	15	2
S. incanata 1-3	460 km	2.3 %	15	4
S. incanata 2-3	460 km	1.9 %	2	4
S. marginepunctata	0 km	2.8 %	16	19
S. confinaria 1-2	460 km	1.6 %	5	5
S. confinaria 1-3	340 km	1.6 %	5	2
S. confinaria 2-3	840 km	1.1 %	5	2
S. asellaria	950 km	2.5 %	4	1+
R. vibicaria	0 km	4.1 %	19	1+
R. pudorata	1850 km	3.2 %	2	1+
R. sacraria	440 km	6.1 %	18	1
L. cruentaria	990 km	1.8 %	5	3
