# Supplementary material for: Genetic Patterns in European Geometrid Moths Revealed by the Barcode Index Number (BIN) System
Source: PLoS One. 2013 Dec 17;8(12):e84518. doi: 10.1371/journal.pone.0084518 (PMC3866169; doi:10.1371/journal.pone.0084518)
Supplement: Appendix S5 — Neighbor Joining Tree. Neighbor Joining Tree (BOLD-Aligner, Kimura 2 parameter) for the 1610 European specimens (barcoded >500 bp), belonging to 183 species and 224 BINs. (PDF) [file pone.0084518.s005.pdf]

BOLD TaxonID Tree

Title	:	SEARCH: Process ids(1611 ids) [SEARCH1]
Date	:	17-July-2013
Data Type	:	Nucleotide
Distance Model	:	Kimura 2 Parameter
Marker	:	COI-5P
Codon Positions	:	1st, 2nd, 3rd
Labels	:	Country & Province, BIN guid
Filters	:	Length > 200
Sequence Count	:	1610
Species count	:	183
Genus count	:	34
Family count	:	1
Unidentified	:	0
BIN Count	:	224

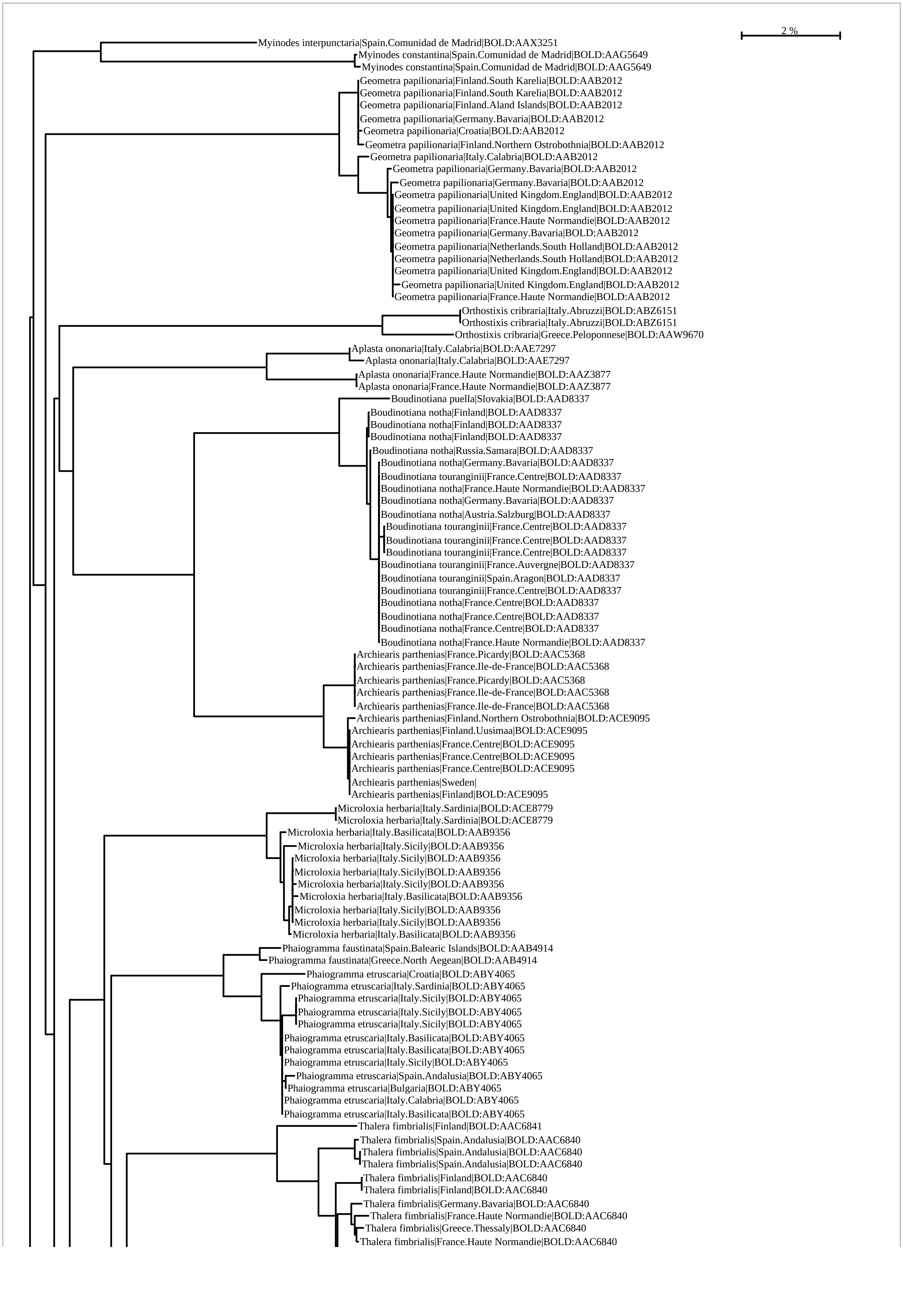
2 %
Myinodes interpunctaria|Spain.Comunidad de Madrid|BOLD:AAX3251
Myinodes constantina|Spain.Comunidad de Madrid|BOLD:AAG5649
Myinodes constantina|Spain.Comunidad de Madrid|BOLD:AAG5649
Geometra papilionaria|Finland.South Karelia|BOLD:AAB2012
Geometra papilionaria|Finland.South Karelia|BOLD:AAB2012
Geometra papilionaria|Finland.Aland Islands|BOLD:AAB2012
Geometra papilionaria|Germany.Bavaria|BOLD:AAB2012
Geometra papilionaria|Croatia|BOLD:AAB2012
Geometra papilionaria|Finland.Northern Ostrobothnia|BOLD:AAB2012
Geometra papilionaria|Italy.Calabria|BOLD:AAB2012
Geometra papilionaria|Germany.Bavaria|BOLD:AAB2012
Geometra papilionaria|Germany.Bavaria|BOLD:AAB2012
Geometra papilionaria|United Kingdom.England|BOLD:AAB2012
Geometra papilionaria|United Kingdom.England|BOLD:AAB2012
Geometra papilionaria|France.Haute Normandie|BOLD:AAB2012
Geometra papilionaria|Germany.Bavaria|BOLD:AAB2012
Geometra papilionaria|Netherlands.South Holland|BOLD:AAB2012
Geometra papilionaria|Netherlands.South Holland|BOLD:AAB2012
Geometra papilionaria|United Kingdom.England|BOLD:AAB2012
Geometra papilionaria|United Kingdom.England|BOLD:AAB2012
Geometra papilionaria|France.Haute Normandie|BOLD:AAB2012
Orthostixis cribraria|Italy.Abruzzi|BOLD:ABZ6151
Orthostixis cribraria|Italy.Abruzzi|BOLD:ABZ6151
Orthostixis cribraria|Greece.Peloponnese|BOLD:AAW9670
Aplasta ononaria|Italy.Calabria|BOLD:AAE7297
Aplasta ononaria|Italy.Calabria|BOLD:AAE7297
Aplasta ononaria|France.Haute Normandie|BOLD:AAZ3877
Aplasta ononaria|France.Haute Normandie|BOLD:AAZ3877
Boudinotiana puella|Slovakia|BOLD:AAD8337
Boudinotiana notha|Finland|BOLD:AAD8337
Boudinotiana notha|Finland|BOLD:AAD8337
Boudinotiana notha|Finland|BOLD:AAD8337
Boudinotiana notha|Russia.Samara|BOLD:AAD8337
Boudinotiana notha|Germany.Bavaria|BOLD:AAD8337
Boudinotiana touranginii|France.Centre|BOLD:AAD8337
Boudinotiana notha|France.Haute Normandie|BOLD:AAD8337
Boudinotiana notha|Germany.Bavaria|BOLD:AAD8337
Boudinotiana notha|Austria.Salzburg|BOLD:AAD8337
Boudinotiana touranginii|France.Centre|BOLD:AAD8337
Boudinotiana touranginii|France.Centre|BOLD:AAD8337
Boudinotiana touranginii|France.Centre|BOLD:AAD8337
Boudinotiana touranginii|France.Auvergne|BOLD:AAD8337
Boudinotiana touranginii|Spain.Aragon|BOLD:AAD8337
Boudinotiana touranginii|France.Centre|BOLD:AAD8337
Boudinotiana notha|France.Centre|BOLD:AAD8337
Boudinotiana notha|France.Centre|BOLD:AAD8337
Boudinotiana notha|France.Centre|BOLD:AAD8337
Boudinotiana notha|France.Haute Normandie|BOLD:AAD8337
Archiearis parthenias|France.Picardy|BOLD:AAC5368
Archiearis parthenias|France.Ile-de-France|BOLD:AAC5368
Archiearis parthenias|France.Picardy|BOLD:AAC5368
Archiearis parthenias|France.Ile-de-France|BOLD:AAC5368
Archiearis parthenias|France.Ile-de-France|BOLD:AAC5368
Archiearis parthenias|Finland.Northern Ostrobothnia|BOLD:ACE9095
Archiearis parthenias|Finland.Uusimaa|BOLD:ACE9095
Archiearis parthenias|France.Centre|BOLD:ACE9095
Archiearis parthenias|France.Centre|BOLD:ACE9095
Archiearis parthenias|France.Centre|BOLD:ACE9095
Archiearis parthenias|Sweden|
Archiearis parthenias|Finland|BOLD:ACE9095
Microloxia herbaria|Italy.Sardinia|BOLD:ACE8779
Microloxia herbaria|Italy.Sardinia|BOLD:ACE8779
Microloxia herbaria|Italy.Basilicata|BOLD:AAB9356
Microloxia herbaria|Italy.Sicily|BOLD:AAB9356
Microloxia herbaria|Italy.Sicily|BOLD:AAB9356
Microloxia herbaria|Italy.Sicily|BOLD:AAB9356
Microloxia herbaria|Italy.Sicily|BOLD:AAB9356
Microloxia herbaria|Italy.Basilicata|BOLD:AAB9356
Microloxia herbaria|Italy.Sicily|BOLD:AAB9356
Microloxia herbaria|Italy.Sicily|BOLD:AAB9356
Microloxia herbaria|Italy.Basilicata|BOLD:AAB9356
Phaiogramma faustinata|Spain.Balearic Islands|BOLD:AAB4914
Phaiogramma faustinata|Greece.North Aegean|BOLD:AAB4914
Phaiogramma etruscaria|Croatia|BOLD:ABY4065
Phaiogramma etruscaria|Italy.Sardinia|BOLD:ABY4065
Phaiogramma etruscaria|Italy.Sicily|BOLD:ABY4065
Phaiogramma etruscaria|Italy.Sicily|BOLD:ABY4065
Phaiogramma etruscaria|Italy.Sicily|BOLD:ABY4065
Phaiogramma etruscaria|Italy.Basilicata|BOLD:ABY4065
Phaiogramma etruscaria|Italy.Basilicata|BOLD:ABY4065
Phaiogramma etruscaria|Italy.Sicily|BOLD:ABY4065
Phaiogramma etruscaria|Spain.Andalusia|BOLD:ABY4065
Phaiogramma etruscaria|Bulgaria|BOLD:ABY4065
Phaiogramma etruscaria|Italy.Calabria|BOLD:ABY4065
Phaiogramma etruscaria|Italy.Basilicata|BOLD:ABY4065
Thalera fimbrialis|Finland|BOLD:AAC6841
Thalera fimbrialis|Spain.Andalusia|BOLD:AAC6840
Thalera fimbrialis|Spain.Andalusia|BOLD:AAC6840
Thalera fimbrialis|Spain.Andalusia|BOLD:AAC6840
Thalera fimbrialis|Finland|BOLD:AAC6840
Thalera fimbrialis|Finland|BOLD:AAC6840
Thalera fimbrialis|Germany.Bavaria|BOLD:AAC6840
Thalera fimbrialis|France.Haute Normandie|BOLD:AAC6840
Thalera fimbrialis|Greece.Thessaly|BOLD:AAC6840
Thalera fimbrialis|France.Haute Normandie|BOLD:AAC6840

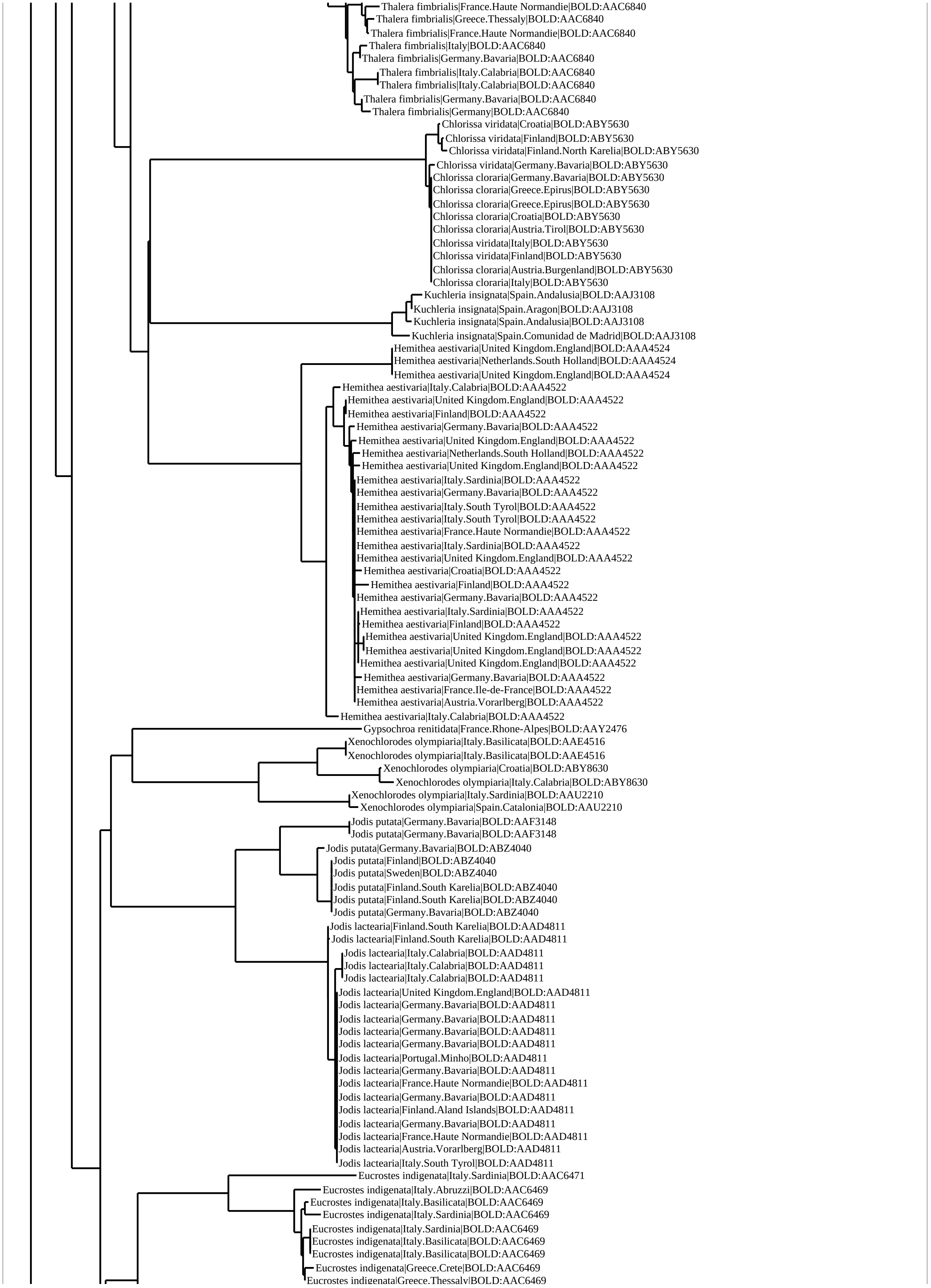
Thalera fimbrialis|France.Haute Normandie|BOLD:AAC6840
Thalera fimbrialis|Greece.Thessaly|BOLD:AAC6840
Thalera fimbrialis|France.Haute Normandie|BOLD:AAC6840
Thalera fimbrialis|Italy|BOLD:AAC6840
Thalera fimbrialis|Germany.Bavaria|BOLD:AAC6840
Thalera fimbrialis|Italy.Calabria|BOLD:AAC6840
Thalera fimbrialis|Italy.Calabria|BOLD:AAC6840
Thalera fimbrialis|Germany.Bavaria|BOLD:AAC6840
Thalera fimbrialis|Germany|BOLD:AAC6840
Chlorissa viridata|Croatia|BOLD:ABY5630
Chlorissa viridata|Finland|BOLD:ABY5630
Chlorissa viridata|Finland.North Karelia|BOLD:ABY5630
Chlorissa viridata|Germany.Bavaria|BOLD:ABY5630
Chlorissa cloraria|Germany.Bavaria|BOLD:ABY5630
Chlorissa cloraria|Greece.Epirus|BOLD:ABY5630
Chlorissa cloraria|Greece.Epirus|BOLD:ABY5630
Chlorissa cloraria|Croatia|BOLD:ABY5630
Chlorissa cloraria|Austria.Tirol|BOLD:ABY5630
Chlorissa viridata|Italy|BOLD:ABY5630
Chlorissa viridata|Finland|BOLD:ABY5630
Chlorissa cloraria|Austria.Burgenland|BOLD:ABY5630
Chlorissa cloraria|Italy|BOLD:ABY5630
Kuchleria insignata|Spain.Andalusia|BOLD:AAJ3108
Kuchleria insignata|Spain.Aragon|BOLD:AAJ3108
Kuchleria insignata|Spain.Andalusia|BOLD:AAJ3108
Kuchleria insignata|Spain.Comunidad de Madrid|BOLD:AAJ3108
Hemithea aestivaria|United Kingdom.England|BOLD:AAA4524
Hemithea aestivaria|Netherlands.South Holland|BOLD:AAA4524
Hemithea aestivaria|United Kingdom.England|BOLD:AAA4524
Hemithea aestivaria|Italy.Calabria|BOLD:AAA4522
Hemithea aestivaria|United Kingdom.England|BOLD:AAA4522
Hemithea aestivaria|Finland|BOLD:AAA4522
Hemithea aestivaria|Germany.Bavaria|BOLD:AAA4522
Hemithea aestivaria|United Kingdom.England|BOLD:AAA4522
Hemithea aestivaria|Netherlands.South Holland|BOLD:AAA4522
Hemithea aestivaria|United Kingdom.England|BOLD:AAA4522
Hemithea aestivaria|Italy.Sardinia|BOLD:AAA4522
Hemithea aestivaria|Germany.Bavaria|BOLD:AAA4522
Hemithea aestivaria|Italy.South Tyrol|BOLD:AAA4522
Hemithea aestivaria|Italy.South Tyrol|BOLD:AAA4522
Hemithea aestivaria|France.Haute Normandie|BOLD:AAA4522
Hemithea aestivaria|Italy.Sardinia|BOLD:AAA4522
Hemithea aestivaria|United Kingdom.England|BOLD:AAA4522
Hemithea aestivaria|Croatia|BOLD:AAA4522
Hemithea aestivaria|Finland|BOLD:AAA4522
Hemithea aestivaria|Germany.Bavaria|BOLD:AAA4522
Hemithea aestivaria|Italy.Sardinia|BOLD:AAA4522
Hemithea aestivaria|Finland|BOLD:AAA4522
Hemithea aestivaria|United Kingdom.England|BOLD:AAA4522
Hemithea aestivaria|United Kingdom.England|BOLD:AAA4522
Hemithea aestivaria|United Kingdom.England|BOLD:AAA4522
Hemithea aestivaria|Germany.Bavaria|BOLD:AAA4522
Hemithea aestivaria|France.Ile-de-France|BOLD:AAA4522
Hemithea aestivaria|Austria.Vorarlberg|BOLD:AAA4522
Hemithea aestivaria|Italy.Calabria|BOLD:AAA4522
Gypsochroa renitidata|France.Rhone-Alpes|BOLD:AAY2476
Xenochlorodes olympiaria|Italy.Basilicata|BOLD:AAE4516
Xenochlorodes olympiaria|Italy.Basilicata|BOLD:AAE4516
Xenochlorodes olympiaria|Croatia|BOLD:ABY8630
Xenochlorodes olympiaria|Italy.Calabria|BOLD:ABY8630
Xenochlorodes olympiaria|Italy.Sardinia|BOLD:AAU2210
Xenochlorodes olympiaria|Spain.Catalonia|BOLD:AAU2210
Jodis putata|Germany.Bavaria|BOLD:AAF3148
Jodis putata|Germany.Bavaria|BOLD:AAF3148
Jodis putata|Germany.Bavaria|BOLD:ABZ4040
Jodis putata|Finland|BOLD:ABZ4040
Jodis putata|Sweden|BOLD:ABZ4040
Jodis putata|Finland.South Karelia|BOLD:ABZ4040
Jodis putata|Finland.South Karelia|BOLD:ABZ4040
Jodis putata|Germany.Bavaria|BOLD:ABZ4040
Jodis lactearia|Finland.South Karelia|BOLD:AAD4811
Jodis lactearia|Finland.South Karelia|BOLD:AAD4811
Jodis lactearia|Italy.Calabria|BOLD:AAD4811
Jodis lactearia|Italy.Calabria|BOLD:AAD4811
Jodis lactearia|Italy.Calabria|BOLD:AAD4811
Jodis lactearia|United Kingdom.England|BOLD:AAD4811
Jodis lactearia|Germany.Bavaria|BOLD:AAD4811
Jodis lactearia|Germany.Bavaria|BOLD:AAD4811
Jodis lactearia|Germany.Bavaria|BOLD:AAD4811
Jodis lactearia|Germany.Bavaria|BOLD:AAD4811
Jodis lactearia|Portugal.Minho|BOLD:AAD4811
Jodis lactearia|Germany.Bavaria|BOLD:AAD4811
Jodis lactearia|France.Haute Normandie|BOLD:AAD4811
Jodis lactearia|Germany.Bavaria|BOLD:AAD4811
Jodis lactearia|Finland.Aland Islands|BOLD:AAD4811
Jodis lactearia|Germany.Bavaria|BOLD:AAD4811
Jodis lactearia|France.Haute Normandie|BOLD:AAD4811
Jodis lactearia|Austria.Vorarlberg|BOLD:AAD4811
Jodis lactearia|Italy.South Tyrol|BOLD:AAD4811
Eucrostes indigenata|Italy.Sardinia|BOLD:AAC6471
Eucrostes indigenata|Italy.Abruzzi|BOLD:AAC6469
Eucrostes indigenata|Italy.Basilicata|BOLD:AAC6469
Eucrostes indigenata|Italy.Sardinia|BOLD:AAC6469
Eucrostes indigenata|Italy.Sardinia|BOLD:AAC6469
Eucrostes indigenata|Italy.Basilicata|BOLD:AAC6469
Eucrostes indigenata|Italy.Basilicata|BOLD:AAC6469
Eucrostes indigenata|Greece.Crete|BOLD:AAC6469
Eucrostes indigenata|Greece.Thessaly|BOLD:AAC6469

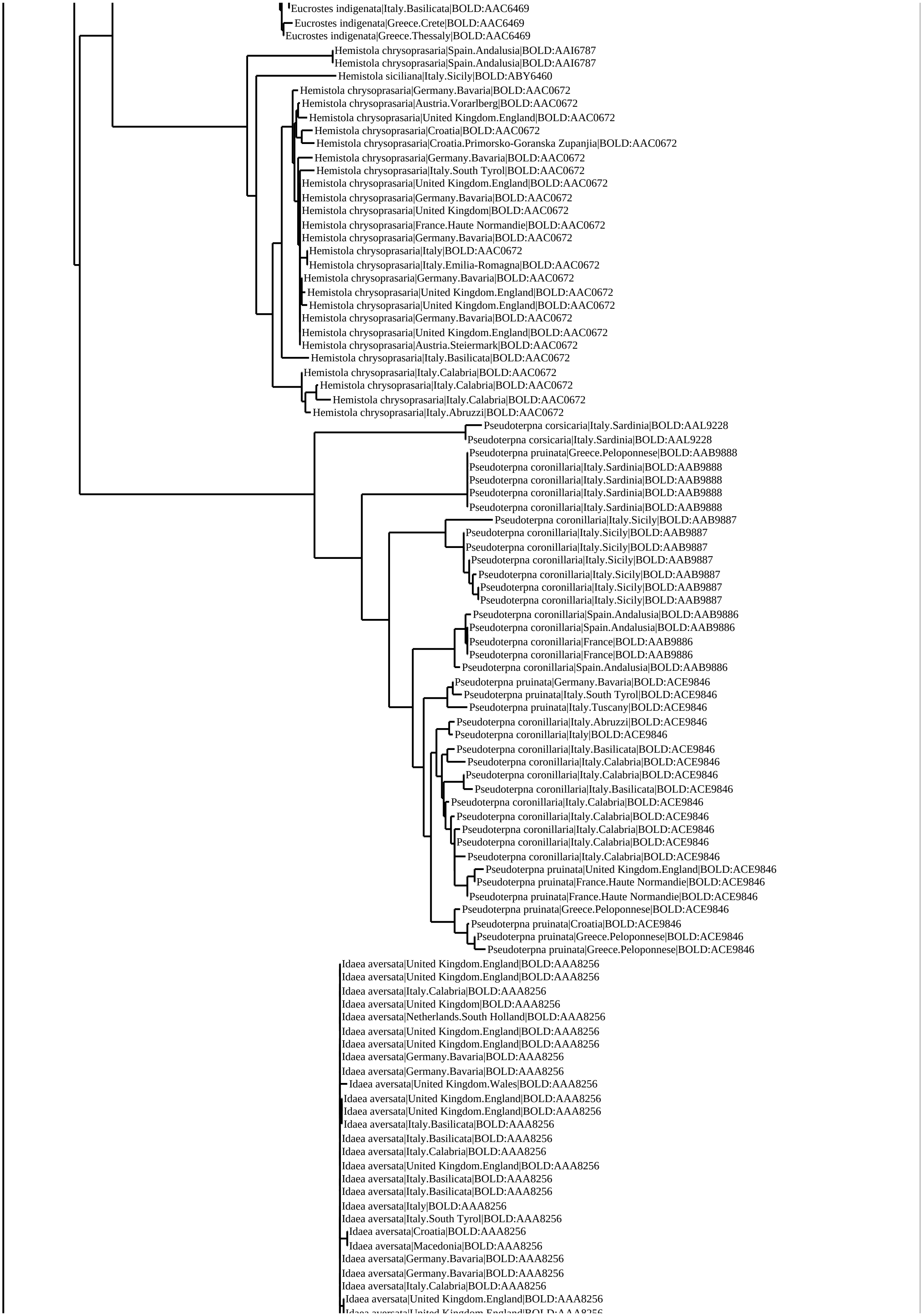
Eucrostes indigenata|Italy.Basilicata|BOLD:AAC6469
Eucrostes indigenata|Greece.Crete|BOLD:AAC6469
Eucrostes indigenata|Greece.Thessaly|BOLD:AAC6469
Hemistola chrysoprasaria|Spain.Andalusia|BOLD:AAI6787
Hemistola chrysoprasaria|Spain.Andalusia|BOLD:AAI6787
Hemistola siciliana|Italy.Sicily|BOLD:ABY6460
Hemistola chrysoprasaria|Germany.Bavaria|BOLD:AAC0672
Hemistola chrysoprasaria|Austria.Vorarlberg|BOLD:AAC0672
Hemistola chrysoprasaria|United Kingdom.England|BOLD:AAC0672
Hemistola chrysoprasaria|Croatia|BOLD:AAC0672
Hemistola chrysoprasaria|Croatia.Primorsko-Goranska Zupanjia|BOLD:AAC0672
Hemistola chrysoprasaria|Germany.Bavaria|BOLD:AAC0672
Hemistola chrysoprasaria|Italy.South Tyrol|BOLD:AAC0672
Hemistola chrysoprasaria|United Kingdom.England|BOLD:AAC0672
Hemistola chrysoprasaria|Germany.Bavaria|BOLD:AAC0672
Hemistola chrysoprasaria|United Kingdom|BOLD:AAC0672
Hemistola chrysoprasaria|France.Haute Normandie|BOLD:AAC0672
Hemistola chrysoprasaria|Germany.Bavaria|BOLD:AAC0672
Hemistola chrysoprasaria|Italy|BOLD:AAC0672
Hemistola chrysoprasaria|Italy.Emilia-Romagna|BOLD:AAC0672
Hemistola chrysoprasaria|Germany.Bavaria|BOLD:AAC0672
Hemistola chrysoprasaria|United Kingdom.England|BOLD:AAC0672
Hemistola chrysoprasaria|United Kingdom.England|BOLD:AAC0672
Hemistola chrysoprasaria|Germany.Bavaria|BOLD:AAC0672
Hemistola chrysoprasaria|United Kingdom.England|BOLD:AAC0672
Hemistola chrysoprasaria|Austria.Steiermark|BOLD:AAC0672
Hemistola chrysoprasaria|Italy.Basilicata|BOLD:AAC0672
Hemistola chrysoprasaria|Italy.Calabria|BOLD:AAC0672
Hemistola chrysoprasaria|Italy.Calabria|BOLD:AAC0672
Hemistola chrysoprasaria|Italy.Calabria|BOLD:AAC0672
Hemistola chrysoprasaria|Italy.Abruzzi|BOLD:AAC0672
Pseudoterpna corsicaria|Italy.Sardinia|BOLD:AAL9228
Pseudoterpna corsicaria|Italy.Sardinia|BOLD:AAL9228
Pseudoterpna pruinata|Greece.Peloponnese|BOLD:AAB9888
Pseudoterpna coronillaria|Italy.Sardinia|BOLD:AAB9888
Pseudoterpna coronillaria|Italy.Sardinia|BOLD:AAB9888
Pseudoterpna coronillaria|Italy.Sardinia|BOLD:AAB9888
Pseudoterpna coronillaria|Italy.Sardinia|BOLD:AAB9888
Pseudoterpna coronillaria|Italy.Sicily|BOLD:AAB9887
Pseudoterpna coronillaria|Italy.Sicily|BOLD:AAB9887
Pseudoterpna coronillaria|Italy.Sicily|BOLD:AAB9887
Pseudoterpna coronillaria|Italy.Sicily|BOLD:AAB9887
Pseudoterpna coronillaria|Italy.Sicily|BOLD:AAB9887
Pseudoterpna coronillaria|Italy.Sicily|BOLD:AAB9887
Pseudoterpna coronillaria|Italy.Sicily|BOLD:AAB9887
Pseudoterpna coronillaria|Spain.Andalusia|BOLD:AAB9886
Pseudoterpna coronillaria|Spain.Andalusia|BOLD:AAB9886
Pseudoterpna coronillaria|France|BOLD:AAB9886
Pseudoterpna coronillaria|France|BOLD:AAB9886
Pseudoterpna coronillaria|Spain.Andalusia|BOLD:AAB9886
Pseudoterpna pruinata|Germany.Bavaria|BOLD:ACE9846
Pseudoterpna pruinata|Italy.South Tyrol|BOLD:ACE9846
Pseudoterpna pruinata|Italy.Tuscany|BOLD:ACE9846
Pseudoterpna coronillaria|Italy.Abruzzi|BOLD:ACE9846
Pseudoterpna coronillaria|Italy|BOLD:ACE9846
Pseudoterpna coronillaria|Italy.Basilicata|BOLD:ACE9846
Pseudoterpna coronillaria|Italy.Calabria|BOLD:ACE9846
Pseudoterpna coronillaria|Italy.Calabria|BOLD:ACE9846
Pseudoterpna coronillaria|Italy.Basilicata|BOLD:ACE9846
Pseudoterpna coronillaria|Italy.Calabria|BOLD:ACE9846
Pseudoterpna coronillaria|Italy.Calabria|BOLD:ACE9846
Pseudoterpna coronillaria|Italy.Calabria|BOLD:ACE9846
Pseudoterpna coronillaria|Italy.Calabria|BOLD:ACE9846
Pseudoterpna coronillaria|Italy.Calabria|BOLD:ACE9846
Pseudoterpna pruinata|United Kingdom.England|BOLD:ACE9846
Pseudoterpna pruinata|France.Haute Normandie|BOLD:ACE9846
Pseudoterpna pruinata|France.Haute Normandie|BOLD:ACE9846
Pseudoterpna pruinata|Greece.Peloponnese|BOLD:ACE9846
Pseudoterpna pruinata|Croatia|BOLD:ACE9846
Pseudoterpna pruinata|Greece.Peloponnese|BOLD:ACE9846
Pseudoterpna pruinata|Greece.Peloponnese|BOLD:ACE9846
Idaea aversata|United Kingdom.England|BOLD:AAA8256
Idaea aversata|United Kingdom.England|BOLD:AAA8256
Idaea aversata|Italy.Calabria|BOLD:AAA8256
Idaea aversata|United Kingdom|BOLD:AAA8256
Idaea aversata|Netherlands.South Holland|BOLD:AAA8256
Idaea aversata|United Kingdom.England|BOLD:AAA8256
Idaea aversata|United Kingdom.England|BOLD:AAA8256
Idaea aversata|Germany.Bavaria|BOLD:AAA8256
Idaea aversata|Germany.Bavaria|BOLD:AAA8256
Idaea aversata|United Kingdom.Wales|BOLD:AAA8256
Idaea aversata|United Kingdom.England|BOLD:AAA8256
Idaea aversata|United Kingdom.England|BOLD:AAA8256
Idaea aversata|Italy.Basilicata|BOLD:AAA8256
Idaea aversata|Italy.Basilicata|BOLD:AAA8256
Idaea aversata|Italy.Calabria|BOLD:AAA8256
Idaea aversata|United Kingdom.England|BOLD:AAA8256
Idaea aversata|Italy.Basilicata|BOLD:AAA8256
Idaea aversata|Italy.Basilicata|BOLD:AAA8256
Idaea aversata|Italy|BOLD:AAA8256
Idaea aversata|Italy.South Tyrol|BOLD:AAA8256
Idaea aversata|Croatia|BOLD:AAA8256
Idaea aversata|Macedonia|BOLD:AAA8256
Idaea aversata|Germany.Bavaria|BOLD:AAA8256
Idaea aversata|Germany.Bavaria|BOLD:AAA8256
Idaea aversata|Italy.Calabria|BOLD:AAA8256
Idaea aversata|United Kingdom.England|BOLD:AAA8256

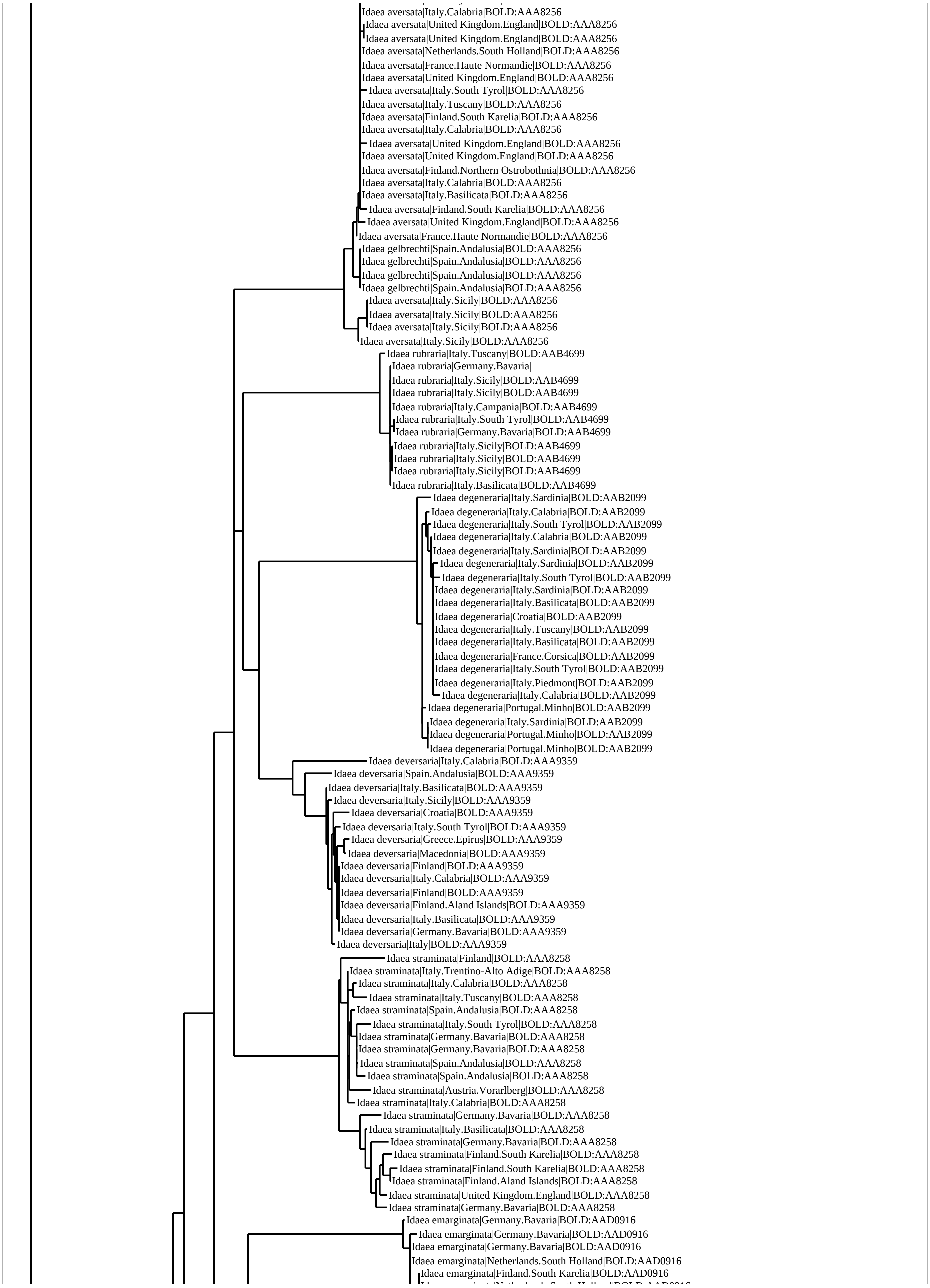
Idaea aversata|Italy.Calabria|BOLD:AAA8256
Idaea aversata|United Kingdom.England|BOLD:AAA8256
Idaea aversata|United Kingdom.England|BOLD:AAA8256
Idaea aversata|Netherlands.South Holland|BOLD:AAA8256
Idaea aversata|France.Haute Normandie|BOLD:AAA8256
Idaea aversata|United Kingdom.England|BOLD:AAA8256
Idaea aversata|Italy.South Tyrol|BOLD:AAA8256
Idaea aversata|Italy.Tuscany|BOLD:AAA8256
Idaea aversata|Finland.South Karelia|BOLD:AAA8256
Idaea aversata|Italy.Calabria|BOLD:AAA8256
Idaea aversata|United Kingdom.England|BOLD:AAA8256
Idaea aversata|United Kingdom.England|BOLD:AAA8256
Idaea aversata|Finland.Northern Ostrobothnia|BOLD:AAA8256
Idaea aversata|Italy.Calabria|BOLD:AAA8256
Idaea aversata|Italy.Basilicata|BOLD:AAA8256
Idaea aversata|Finland.South Karelia|BOLD:AAA8256
Idaea aversata|United Kingdom.England|BOLD:AAA8256
Idaea aversata|France.Haute Normandie|BOLD:AAA8256
Idaea gelbrechti|Spain.Andalusia|BOLD:AAA8256
Idaea gelbrechti|Spain.Andalusia|BOLD:AAA8256
Idaea gelbrechti|Spain.Andalusia|BOLD:AAA8256
Idaea gelbrechti|Spain.Andalusia|BOLD:AAA8256
Idaea aversata|Italy.Sicily|BOLD:AAA8256
Idaea aversata|Italy.Sicily|BOLD:AAA8256
Idaea aversata|Italy.Sicily|BOLD:AAA8256
Idaea aversata|Italy.Sicily|BOLD:AAA8256
Idaea rubraria|Italy.Tuscany|BOLD:AAB4699
Idaea rubraria|Germany.Bavaria|
Idaea rubraria|Italy.Sicily|BOLD:AAB4699
Idaea rubraria|Italy.Sicily|BOLD:AAB4699
Idaea rubraria|Italy.Campania|BOLD:AAB4699
Idaea rubraria|Italy.South Tyrol|BOLD:AAB4699
Idaea rubraria|Germany.Bavaria|BOLD:AAB4699
Idaea rubraria|Italy.Sicily|BOLD:AAB4699
Idaea rubraria|Italy.Sicily|BOLD:AAB4699
Idaea rubraria|Italy.Sicily|BOLD:AAB4699
Idaea rubraria|Italy.Basilicata|BOLD:AAB4699
Idaea degeneraria|Italy.Sardinia|BOLD:AAB2099
Idaea degeneraria|Italy.Calabria|BOLD:AAB2099
Idaea degeneraria|Italy.South Tyrol|BOLD:AAB2099
Idaea degeneraria|Italy.Calabria|BOLD:AAB2099
Idaea degeneraria|Italy.Sardinia|BOLD:AAB2099
Idaea degeneraria|Italy.Sardinia|BOLD:AAB2099
Idaea degeneraria|Italy.South Tyrol|BOLD:AAB2099
Idaea degeneraria|Italy.Sardinia|BOLD:AAB2099
Idaea degeneraria|Italy.Basilicata|BOLD:AAB2099
Idaea degeneraria|Croatia|BOLD:AAB2099
Idaea degeneraria|Italy.Tuscany|BOLD:AAB2099
Idaea degeneraria|Italy.Basilicata|BOLD:AAB2099
Idaea degeneraria|France.Corsica|BOLD:AAB2099
Idaea degeneraria|Italy.South Tyrol|BOLD:AAB2099
Idaea degeneraria|Italy.Piedmont|BOLD:AAB2099
Idaea degeneraria|Italy.Calabria|BOLD:AAB2099
Idaea degeneraria|Portugal.Minho|BOLD:AAB2099
Idaea degeneraria|Italy.Sardinia|BOLD:AAB2099
Idaea degeneraria|Portugal.Minho|BOLD:AAB2099
Idaea degeneraria|Portugal.Minho|BOLD:AAB2099
Idaea deversaria|Italy.Calabria|BOLD:AAA9359
Idaea deversaria|Spain.Andalusia|BOLD:AAA9359
Idaea deversaria|Italy.Basilicata|BOLD:AAA9359
Idaea deversaria|Italy.Sicily|BOLD:AAA9359
Idaea deversaria|Croatia|BOLD:AAA9359
Idaea deversaria|Italy.South Tyrol|BOLD:AAA9359
Idaea deversaria|Greece.Epirus|BOLD:AAA9359
Idaea deversaria|Macedonia|BOLD:AAA9359
Idaea deversaria|Finland|BOLD:AAA9359
Idaea deversaria|Italy.Calabria|BOLD:AAA9359
Idaea deversaria|Finland|BOLD:AAA9359
Idaea deversaria|Finland.Aland Islands|BOLD:AAA9359
Idaea deversaria|Italy.Basilicata|BOLD:AAA9359
Idaea deversaria|Germany.Bavaria|BOLD:AAA9359
Idaea deversaria|Italy|BOLD:AAA9359
Idaea straminata|Finland|BOLD:AAA8258
Idaea straminata|Italy.Trentino-Alto Adige|BOLD:AAA8258
Idaea straminata|Italy.Calabria|BOLD:AAA8258
Idaea straminata|Italy.Tuscany|BOLD:AAA8258
Idaea straminata|Spain.Andalusia|BOLD:AAA8258
Idaea straminata|Italy.South Tyrol|BOLD:AAA8258
Idaea straminata|Germany.Bavaria|BOLD:AAA8258
Idaea straminata|Germany.Bavaria|BOLD:AAA8258
Idaea straminata|Spain.Andalusia|BOLD:AAA8258
Idaea straminata|Spain.Andalusia|BOLD:AAA8258
Idaea straminata|Austria.Vorarlberg|BOLD:AAA8258
Idaea straminata|Italy.Calabria|BOLD:AAA8258
Idaea straminata|Germany.Bavaria|BOLD:AAA8258
Idaea straminata|Italy.Basilicata|BOLD:AAA8258
Idaea straminata|Germany.Bavaria|BOLD:AAA8258
Idaea straminata|Finland.South Karelia|BOLD:AAA8258
Idaea straminata|Finland.South Karelia|BOLD:AAA8258
Idaea straminata|Finland.Aland Islands|BOLD:AAA8258
Idaea straminata|United Kingdom.England|BOLD:AAA8258
Idaea straminata|Germany.Bavaria|BOLD:AAA8258
Idaea emarginata|Germany.Bavaria|BOLD:AAD0916
Idaea emarginata|Germany.Bavaria|BOLD:AAD0916
Idaea emarginata|Germany.Bavaria|BOLD:AAD0916
Idaea emarginata|Netherlands.South Holland|BOLD:AAD0916
Idaea emarginata|Finland.South Karelia|BOLD:AAD0916

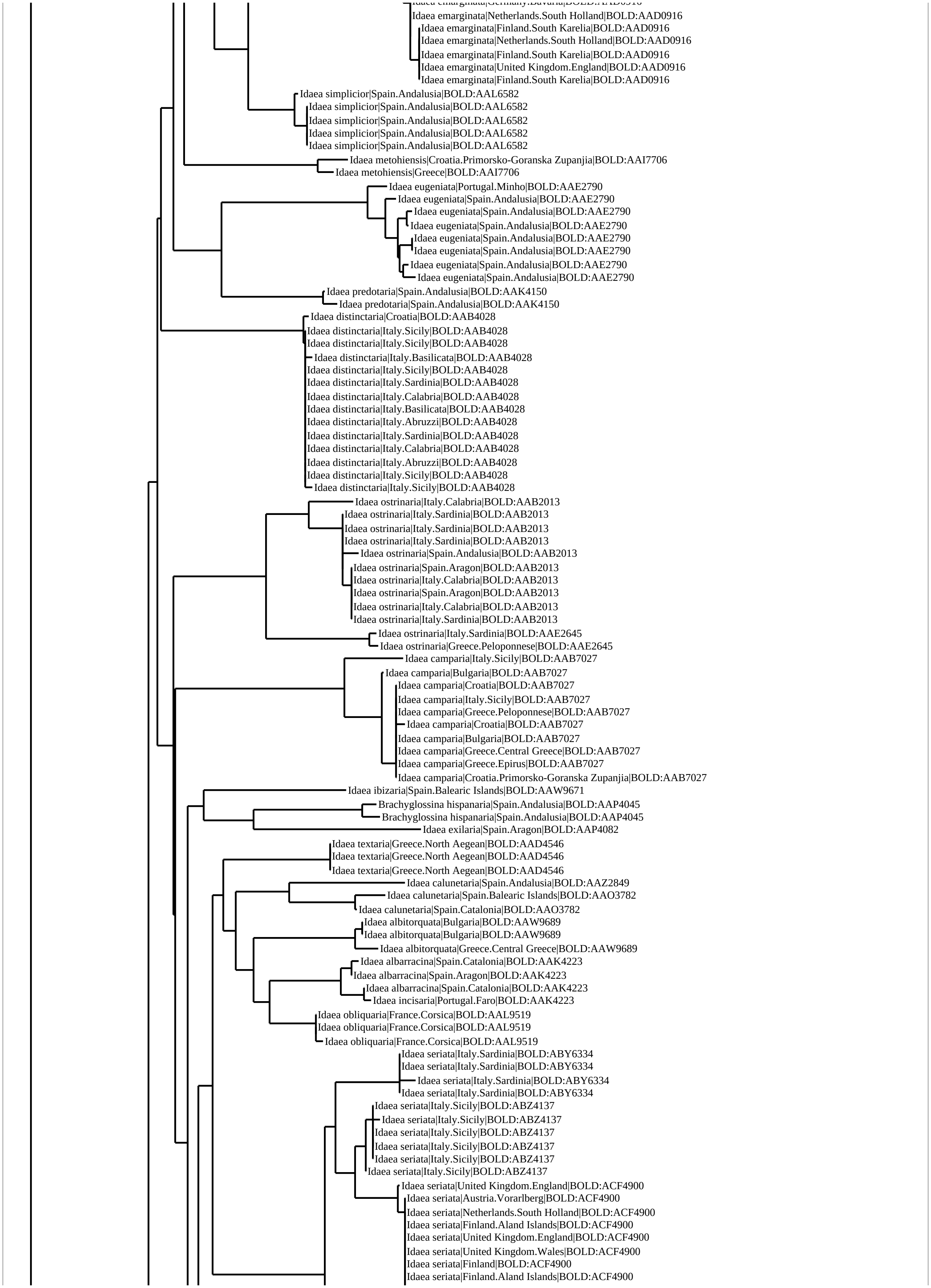
Idaea emarginata|Netherlands.South Holland|BOLD:AAD0916
Idaea emarginata|Finland.South Karelia|BOLD:AAD0916
Idaea emarginata|Netherlands.South Holland|BOLD:AAD0916
Idaea emarginata|Finland.South Karelia|BOLD:AAD0916
Idaea emarginata|United Kingdom.England|BOLD:AAD0916
Idaea emarginata|Finland.South Karelia|BOLD:AAD0916
Idaea simplicior|Spain.Andalusia|BOLD:AAL6582
Idaea simplicior|Spain.Andalusia|BOLD:AAL6582
Idaea simplicior|Spain.Andalusia|BOLD:AAL6582
Idaea simplicior|Spain.Andalusia|BOLD:AAL6582
Idaea simplicior|Spain.Andalusia|BOLD:AAL6582
Idaea metohiensis|Croatia.Primorsko-Goranska Zupanjia|BOLD:AAI7706
Idaea metohiensis|Greece|BOLD:AAI7706
Idaea eugeniata|Portugal.Minho|BOLD:AAE2790
Idaea eugeniata|Spain.Andalusia|BOLD:AAE2790
Idaea eugeniata|Spain.Andalusia|BOLD:AAE2790
Idaea eugeniata|Spain.Andalusia|BOLD:AAE2790
Idaea eugeniata|Spain.Andalusia|BOLD:AAE2790
Idaea eugeniata|Spain.Andalusia|BOLD:AAE2790
Idaea eugeniata|Spain.Andalusia|BOLD:AAE2790
Idaea eugeniata|Spain.Andalusia|BOLD:AAE2790
Idaea predotaria|Spain.Andalusia|BOLD:AAK4150
Idaea predotaria|Spain.Andalusia|BOLD:AAK4150
Idaea distinctaria|Croatia|BOLD:AAB4028
Idaea distinctaria|Italy.Sicily|BOLD:AAB4028
Idaea distinctaria|Italy.Sicily|BOLD:AAB4028
Idaea distinctaria|Italy.Basilicata|BOLD:AAB4028
Idaea distinctaria|Italy.Sicily|BOLD:AAB4028
Idaea distinctaria|Italy.Sardinia|BOLD:AAB4028
Idaea distinctaria|Italy.Calabria|BOLD:AAB4028
Idaea distinctaria|Italy.Basilicata|BOLD:AAB4028
Idaea distinctaria|Italy.Abruzzi|BOLD:AAB4028
Idaea distinctaria|Italy.Sardinia|BOLD:AAB4028
Idaea distinctaria|Italy.Calabria|BOLD:AAB4028
Idaea distinctaria|Italy.Abruzzi|BOLD:AAB4028
Idaea distinctaria|Italy.Sicily|BOLD:AAB4028
Idaea distinctaria|Italy.Sicily|BOLD:AAB4028
Idaea ostrinaria|Italy.Calabria|BOLD:AAB2013
Idaea ostrinaria|Italy.Sardinia|BOLD:AAB2013
Idaea ostrinaria|Italy.Sardinia|BOLD:AAB2013
Idaea ostrinaria|Italy.Sardinia|BOLD:AAB2013
Idaea ostrinaria|Spain.Andalusia|BOLD:AAB2013
Idaea ostrinaria|Spain.Aragon|BOLD:AAB2013
Idaea ostrinaria|Italy.Calabria|BOLD:AAB2013
Idaea ostrinaria|Spain.Aragon|BOLD:AAB2013
Idaea ostrinaria|Italy.Calabria|BOLD:AAB2013
Idaea ostrinaria|Italy.Sardinia|BOLD:AAB2013
Idaea ostrinaria|Italy.Sardinia|BOLD:AAE2645
Idaea ostrinaria|Greece.Peloponnese|BOLD:AAE2645
Idaea camparia|Italy.Sicily|BOLD:AAB7027
Idaea camparia|Bulgaria|BOLD:AAB7027
Idaea camparia|Croatia|BOLD:AAB7027
Idaea camparia|Italy.Sicily|BOLD:AAB7027
Idaea camparia|Greece.Peloponnese|BOLD:AAB7027
Idaea camparia|Croatia|BOLD:AAB7027
Idaea camparia|Bulgaria|BOLD:AAB7027
Idaea camparia|Greece.Central Greece|BOLD:AAB7027
Idaea camparia|Greece.Epirus|BOLD:AAB7027
Idaea camparia|Croatia.Primorsko-Goranska Zupanjia|BOLD:AAB7027
Idaea ibizaria|Spain.Balearic Islands|BOLD:AAW9671
Brachyglossina hispanaria|Spain.Andalusia|BOLD:AAP4045
Brachyglossina hispanaria|Spain.Andalusia|BOLD:AAP4045
Idaea exilaria|Spain.Aragon|BOLD:AAP4082
Idaea textaria|Greece.North Aegean|BOLD:AAD4546
Idaea textaria|Greece.North Aegean|BOLD:AAD4546
Idaea textaria|Greece.North Aegean|BOLD:AAD4546
Idaea calunetaria|Spain.Andalusia|BOLD:AAZ2849
Idaea calunetaria|Spain.Balearic Islands|BOLD:AAO3782
Idaea calunetaria|Spain.Catalonia|BOLD:AAO3782
Idaea albitorquata|Bulgaria|BOLD:AAW9689
Idaea albitorquata|Bulgaria|BOLD:AAW9689
Idaea albitorquata|Greece.Central Greece|BOLD:AAW9689
Idaea albarracina|Spain.Catalonia|BOLD:AAK4223
Idaea albarracina|Spain.Aragon|BOLD:AAK4223
Idaea albarracina|Spain.Catalonia|BOLD:AAK4223
Idaea incisaria|Portugal.Faro|BOLD:AAK4223
Idaea obliquaria|France.Corsica|BOLD:AAL9519
Idaea obliquaria|France.Corsica|BOLD:AAL9519
Idaea obliquaria|France.Corsica|BOLD:AAL9519
Idaea seriata|Italy.Sardinia|BOLD:ABY6334
Idaea seriata|Italy.Sardinia|BOLD:ABY6334
Idaea seriata|Italy.Sardinia|BOLD:ABY6334
Idaea seriata|Italy.Sardinia|BOLD:ABY6334
Idaea seriata|Italy.Sicily|BOLD:ABZ4137
Idaea seriata|Italy.Sicily|BOLD:ABZ4137
Idaea seriata|Italy.Sicily|BOLD:ABZ4137
Idaea seriata|Italy.Sicily|BOLD:ABZ4137
Idaea seriata|Italy.Sicily|BOLD:ABZ4137
Idaea seriata|Italy.Sicily|BOLD:ABZ4137
Idaea seriata|United Kingdom.England|BOLD:ACF4900
Idaea seriata|Austria.Vorarlberg|BOLD:ACF4900
Idaea seriata|Netherlands.South Holland|BOLD:ACF4900
Idaea seriata|Finland.Aland Islands|BOLD:ACF4900
Idaea seriata|United Kingdom.England|BOLD:ACF4900
Idaea seriata|United Kingdom.Wales|BOLD:ACF4900
Idaea seriata|Finland|BOLD:ACF4900
Idaea seriata|Finland.Aland Islands|BOLD:ACF4900

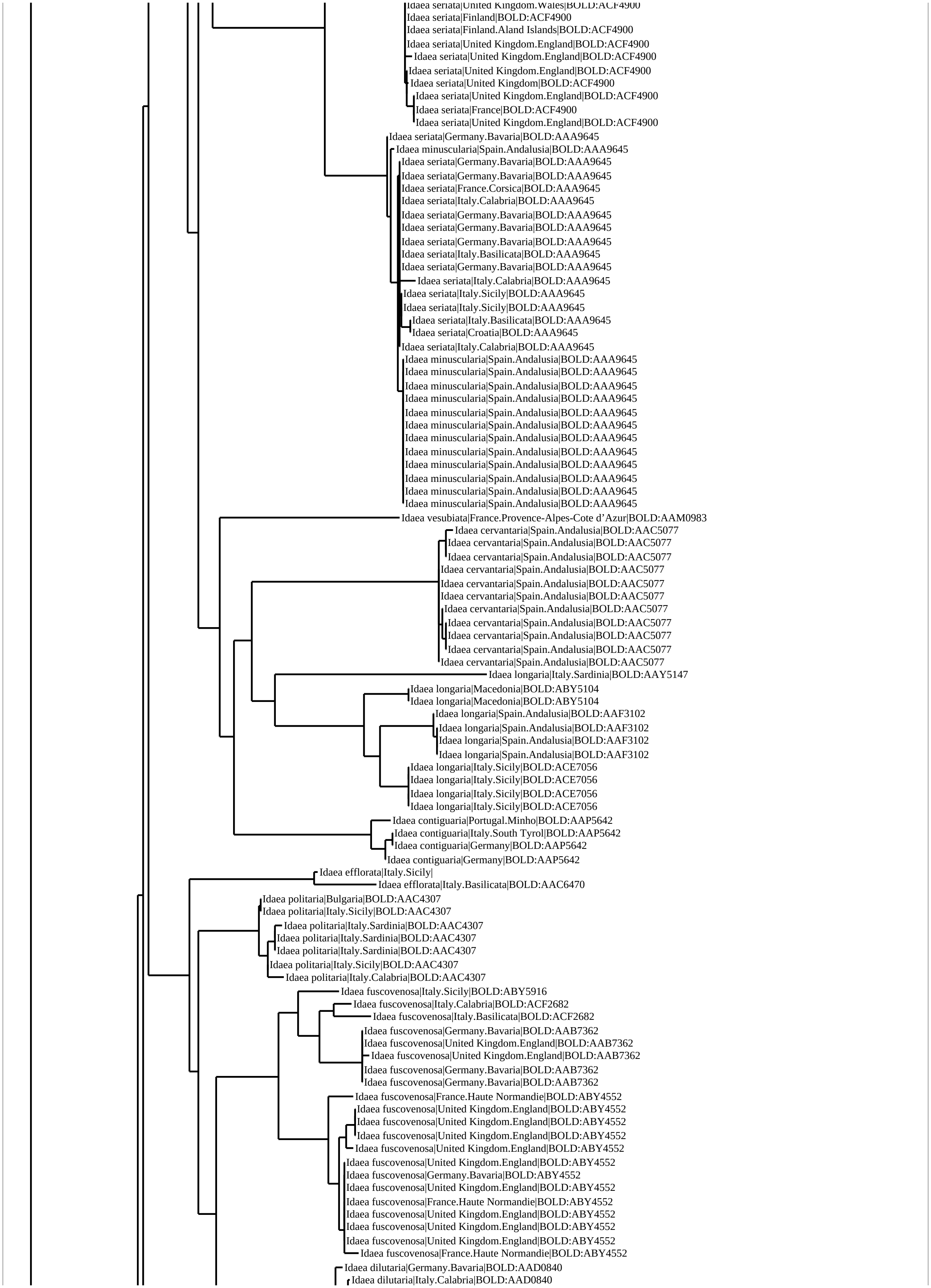
Idaea seriata|United Kingdom.Wales|BOLD:ACF4900
Idaea seriata|Finland|BOLD:ACF4900
Idaea seriata|Finland.Aland Islands|BOLD:ACF4900
Idaea seriata|United Kingdom.England|BOLD:ACF4900
Idaea seriata|United Kingdom.England|BOLD:ACF4900
Idaea seriata|United Kingdom.England|BOLD:ACF4900
Idaea seriata|United Kingdom|BOLD:ACF4900
Idaea seriata|United Kingdom.England|BOLD:ACF4900
Idaea seriata|France|BOLD:ACF4900
Idaea seriata|United Kingdom.England|BOLD:ACF4900
Idaea seriata|Germany.Bavaria|BOLD:AAA9645
Idaea minuscularia|Spain.Andalusia|BOLD:AAA9645
Idaea seriata|Germany.Bavaria|BOLD:AAA9645
Idaea seriata|Germany.Bavaria|BOLD:AAA9645
Idaea seriata|France.Corsica|BOLD:AAA9645
Idaea seriata|Italy.Calabria|BOLD:AAA9645
Idaea seriata|Germany.Bavaria|BOLD:AAA9645
Idaea seriata|Germany.Bavaria|BOLD:AAA9645
Idaea seriata|Germany.Bavaria|BOLD:AAA9645
Idaea seriata|Italy.Basilicata|BOLD:AAA9645
Idaea seriata|Germany.Bavaria|BOLD:AAA9645
Idaea seriata|Italy.Calabria|BOLD:AAA9645
Idaea seriata|Italy.Sicily|BOLD:AAA9645
Idaea seriata|Italy.Sicily|BOLD:AAA9645
Idaea seriata|Italy.Basilicata|BOLD:AAA9645
Idaea seriata|Croatia|BOLD:AAA9645
Idaea seriata|Italy.Calabria|BOLD:AAA9645
Idaea minuscularia|Spain.Andalusia|BOLD:AAA9645
Idaea minuscularia|Spain.Andalusia|BOLD:AAA9645
Idaea minuscularia|Spain.Andalusia|BOLD:AAA9645
Idaea minuscularia|Spain.Andalusia|BOLD:AAA9645
Idaea minuscularia|Spain.Andalusia|BOLD:AAA9645
Idaea minuscularia|Spain.Andalusia|BOLD:AAA9645
Idaea minuscularia|Spain.Andalusia|BOLD:AAA9645
Idaea minuscularia|Spain.Andalusia|BOLD:AAA9645
Idaea minuscularia|Spain.Andalusia|BOLD:AAA9645
Idaea minuscularia|Spain.Andalusia|BOLD:AAA9645
Idaea minuscularia|Spain.Andalusia|BOLD:AAA9645
Idaea minuscularia|Spain.Andalusia|BOLD:AAA9645
Idaea vesubiata|France.Provence-Alpes-Cote d'Azur|BOLD:AAM0983
Idaea cervantaria|Spain.Andalusia|BOLD:AAC5077
Idaea cervantaria|Spain.Andalusia|BOLD:AAC5077
Idaea cervantaria|Spain.Andalusia|BOLD:AAC5077
Idaea cervantaria|Spain.Andalusia|BOLD:AAC5077
Idaea cervantaria|Spain.Andalusia|BOLD:AAC5077
Idaea cervantaria|Spain.Andalusia|BOLD:AAC5077
Idaea cervantaria|Spain.Andalusia|BOLD:AAC5077
Idaea cervantaria|Spain.Andalusia|BOLD:AAC5077
Idaea cervantaria|Spain.Andalusia|BOLD:AAC5077
Idaea cervantaria|Spain.Andalusia|BOLD:AAC5077
Idaea cervantaria|Spain.Andalusia|BOLD:AAC5077
Idaea longaria|Italy.Sardinia|BOLD:AAY5147
Idaea longaria|Macedonia|BOLD:ABY5104
Idaea longaria|Macedonia|BOLD:ABY5104
Idaea longaria|Spain.Andalusia|BOLD:AAF3102
Idaea longaria|Spain.Andalusia|BOLD:AAF3102
Idaea longaria|Spain.Andalusia|BOLD:AAF3102
Idaea longaria|Spain.Andalusia|BOLD:AAF3102
Idaea longaria|Italy.Sicily|BOLD:ACE7056
Idaea longaria|Italy.Sicily|BOLD:ACE7056
Idaea longaria|Italy.Sicily|BOLD:ACE7056
Idaea longaria|Italy.Sicily|BOLD:ACE7056
Idaea contiguaria|Portugal.Minho|BOLD:AAP5642
Idaea contiguaria|Italy.South Tyrol|BOLD:AAP5642
Idaea contiguaria|Germany|BOLD:AAP5642
Idaea contiguaria|Germany|BOLD:AAP5642
Idaea efflorata|Italy.Sicily|
Idaea efflorata|Italy.Basilicata|BOLD:AAC6470
Idaea politaria|Bulgaria|BOLD:AAC4307
Idaea politaria|Italy.Sicily|BOLD:AAC4307
Idaea politaria|Italy.Sardinia|BOLD:AAC4307
Idaea politaria|Italy.Sardinia|BOLD:AAC4307
Idaea politaria|Italy.Sardinia|BOLD:AAC4307
Idaea politaria|Italy.Sicily|BOLD:AAC4307
Idaea politaria|Italy.Calabria|BOLD:AAC4307
Idaea fuscovenosa|Italy.Sicily|BOLD:ABY5916
Idaea fuscovenosa|Italy.Calabria|BOLD:ACF2682
Idaea fuscovenosa|Italy.Basilicata|BOLD:ACF2682
Idaea fuscovenosa|Germany.Bavaria|BOLD:AAB7362
Idaea fuscovenosa|United Kingdom.England|BOLD:AAB7362
Idaea fuscovenosa|United Kingdom.England|BOLD:AAB7362
Idaea fuscovenosa|Germany.Bavaria|BOLD:AAB7362
Idaea fuscovenosa|Germany.Bavaria|BOLD:AAB7362
Idaea fuscovenosa|France.Haute Normandie|BOLD:ABY4552
Idaea fuscovenosa|United Kingdom.England|BOLD:ABY4552
Idaea fuscovenosa|United Kingdom.England|BOLD:ABY4552
Idaea fuscovenosa|United Kingdom.England|BOLD:ABY4552
Idaea fuscovenosa|United Kingdom.England|BOLD:ABY4552
Idaea fuscovenosa|United Kingdom.England|BOLD:ABY4552
Idaea fuscovenosa|Germany.Bavaria|BOLD:ABY4552
Idaea fuscovenosa|United Kingdom.England|BOLD:ABY4552
Idaea fuscovenosa|France.Haute Normandie|BOLD:ABY4552
Idaea fuscovenosa|United Kingdom.England|BOLD:ABY4552
Idaea fuscovenosa|United Kingdom.England|BOLD:ABY4552
Idaea fuscovenosa|United Kingdom.England|BOLD:ABY4552
Idaea fuscovenosa|France.Haute Normandie|BOLD:ABY4552
Idaea dilutaria|Germany.Bavaria|BOLD:AAD0840
Idaea dilutaria|Italy.Calabria|BOLD:AAD0840

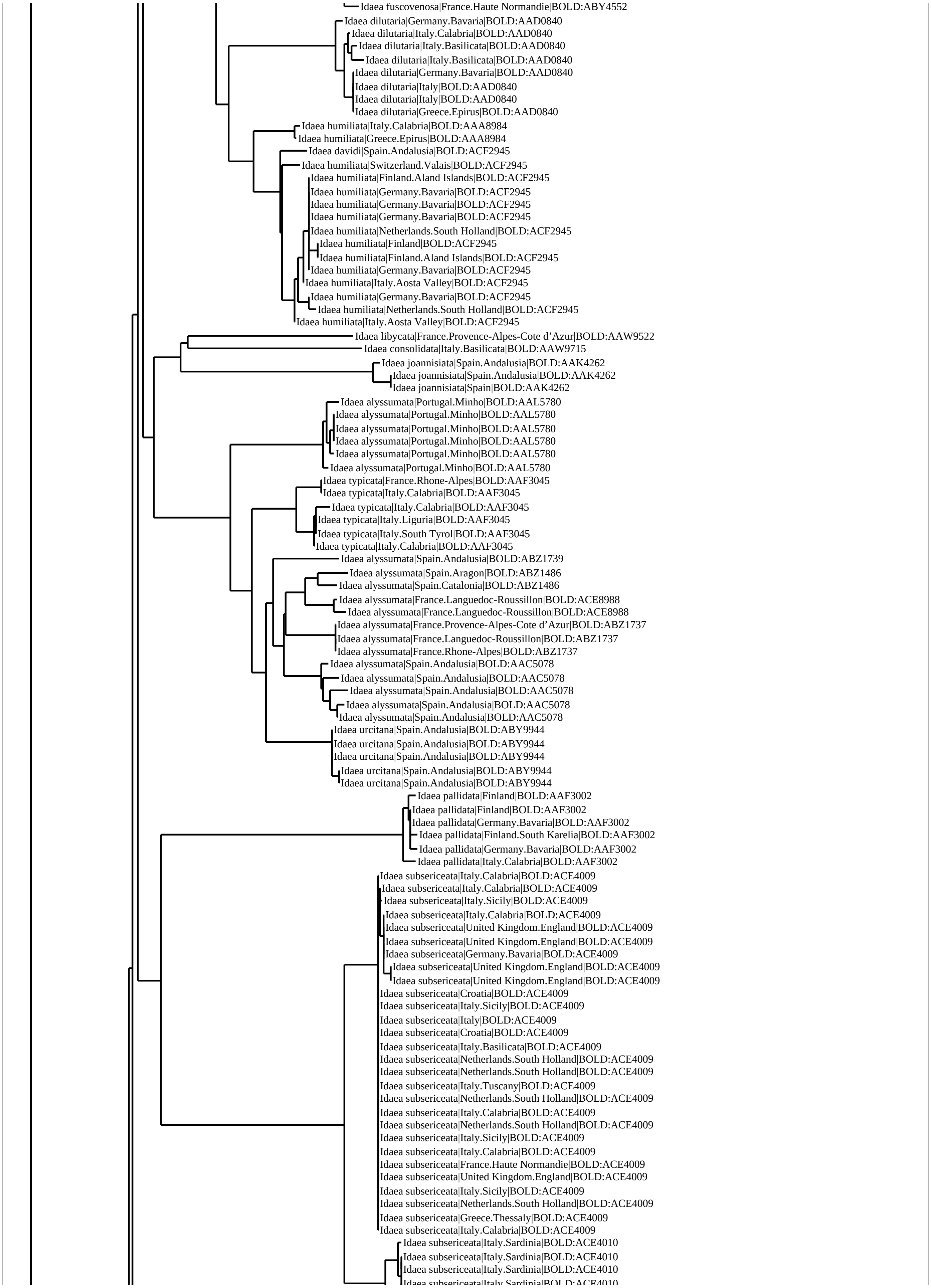
Idaea fuscovenosa|France.Haute Normandie|BOLD:ABY4552
Idaea dilutaria|Germany.Bavaria|BOLD:AAD0840
Idaea dilutaria|Italy.Calabria|BOLD:AAD0840
Idaea dilutaria|Italy.Basilicata|BOLD:AAD0840
Idaea dilutaria|Italy.Basilicata|BOLD:AAD0840
Idaea dilutaria|Germany.Bavaria|BOLD:AAD0840
Idaea dilutaria|Italy|BOLD:AAD0840
Idaea dilutaria|Italy|BOLD:AAD0840
Idaea dilutaria|Greece.Epirus|BOLD:AAD0840
Idaea humiliata|Italy.Calabria|BOLD:AAA8984
Idaea humiliata|Greece.Epirus|BOLD:AAA8984
Idaea davidi|Spain.Andalusia|BOLD:ACF2945
Idaea humiliata|Switzerland.Valais|BOLD:ACF2945
Idaea humiliata|Finland.Aland Islands|BOLD:ACF2945
Idaea humiliata|Germany.Bavaria|BOLD:ACF2945
Idaea humiliata|Germany.Bavaria|BOLD:ACF2945
Idaea humiliata|Germany.Bavaria|BOLD:ACF2945
Idaea humiliata|Netherlands.South Holland|BOLD:ACF2945
Idaea humiliata|Finland|BOLD:ACF2945
Idaea humiliata|Finland.Aland Islands|BOLD:ACF2945
Idaea humiliata|Germany.Bavaria|BOLD:ACF2945
Idaea humiliata|Italy.Aosta Valley|BOLD:ACF2945
Idaea humiliata|Germany.Bavaria|BOLD:ACF2945
Idaea humiliata|Netherlands.South Holland|BOLD:ACF2945
Idaea humiliata|Italy.Aosta Valley|BOLD:ACF2945
Idaea libycata|France.Provence-Alpes-Cote d'Azur|BOLD:AAW9522
Idaea consolidata|Italy.Basilicata|BOLD:AAW9715
Idaea joannisiata|Spain.Andalusia|BOLD:AAK4262
Idaea joannisiata|Spain.Andalusia|BOLD:AAK4262
Idaea joannisiata|Spain|BOLD:AAK4262
Idaea alyssumata|Portugal.Minho|BOLD:AAL5780
Idaea alyssumata|Portugal.Minho|BOLD:AAL5780
Idaea alyssumata|Portugal.Minho|BOLD:AAL5780
Idaea alyssumata|Portugal.Minho|BOLD:AAL5780
Idaea alyssumata|Portugal.Minho|BOLD:AAL5780
Idaea alyssumata|Portugal.Minho|BOLD:AAL5780
Idaea typicata|France.Rhone-Alpes|BOLD:AAF3045
Idaea typicata|Italy.Calabria|BOLD:AAF3045
Idaea typicata|Italy.Calabria|BOLD:AAF3045
Idaea typicata|Italy.Liguria|BOLD:AAF3045
Idaea typicata|Italy.South Tyrol|BOLD:AAF3045
Idaea typicata|Italy.Calabria|BOLD:AAF3045
Idaea alyssumata|Spain.Andalusia|BOLD:ABZ1739
Idaea alyssumata|Spain.Aragon|BOLD:ABZ1486
Idaea alyssumata|Spain.Catalonia|BOLD:ABZ1486
Idaea alyssumata|France.Languedoc-Roussillon|BOLD:ACE8988
Idaea alyssumata|France.Languedoc-Roussillon|BOLD:ACE8988
Idaea alyssumata|France.Provence-Alpes-Cote d'Azur|BOLD:ABZ1737
Idaea alyssumata|France.Languedoc-Roussillon|BOLD:ABZ1737
Idaea alyssumata|France.Rhone-Alpes|BOLD:ABZ1737
Idaea alyssumata|Spain.Andalusia|BOLD:AAC5078
Idaea alyssumata|Spain.Andalusia|BOLD:AAC5078
Idaea alyssumata|Spain.Andalusia|BOLD:AAC5078
Idaea alyssumata|Spain.Andalusia|BOLD:AAC5078
Idaea alyssumata|Spain.Andalusia|BOLD:AAC5078
Idaea urcitana|Spain.Andalusia|BOLD:ABY9944
Idaea urcitana|Spain.Andalusia|BOLD:ABY9944
Idaea urcitana|Spain.Andalusia|BOLD:ABY9944
Idaea urcitana|Spain.Andalusia|BOLD:ABY9944
Idaea urcitana|Spain.Andalusia|BOLD:ABY9944
Idaea pallidata|Finland|BOLD:AAF3002
Idaea pallidata|Finland|BOLD:AAF3002
Idaea pallidata|Germany.Bavaria|BOLD:AAF3002
Idaea pallidata|Finland.South Karelia|BOLD:AAF3002
Idaea pallidata|Germany.Bavaria|BOLD:AAF3002
Idaea pallidata|Italy.Calabria|BOLD:AAF3002
Idaea subsericeata|Italy.Calabria|BOLD:ACE4009
Idaea subsericeata|Italy.Calabria|BOLD:ACE4009
Idaea subsericeata|Italy.Sicily|BOLD:ACE4009
Idaea subsericeata|Italy.Calabria|BOLD:ACE4009
Idaea subsericeata|United Kingdom.England|BOLD:ACE4009
Idaea subsericeata|United Kingdom.England|BOLD:ACE4009
Idaea subsericeata|Germany.Bavaria|BOLD:ACE4009
Idaea subsericeata|United Kingdom.England|BOLD:ACE4009
Idaea subsericeata|United Kingdom.England|BOLD:ACE4009
Idaea subsericeata|Croatia|BOLD:ACE4009
Idaea subsericeata|Italy.Sicily|BOLD:ACE4009
Idaea subsericeata|Italy|BOLD:ACE4009
Idaea subsericeata|Croatia|BOLD:ACE4009
Idaea subsericeata|Italy.Basilicata|BOLD:ACE4009
Idaea subsericeata|Netherlands.South Holland|BOLD:ACE4009
Idaea subsericeata|Netherlands.South Holland|BOLD:ACE4009
Idaea subsericeata|Italy.Tuscany|BOLD:ACE4009
Idaea subsericeata|Netherlands.South Holland|BOLD:ACE4009
Idaea subsericeata|Italy.Calabria|BOLD:ACE4009
Idaea subsericeata|Netherlands.South Holland|BOLD:ACE4009
Idaea subsericeata|Italy.Sicily|BOLD:ACE4009
Idaea subsericeata|Italy.Calabria|BOLD:ACE4009
Idaea subsericeata|France.Haute Normandie|BOLD:ACE4009
Idaea subsericeata|United Kingdom.England|BOLD:ACE4009
Idaea subsericeata|Italy.Sicily|BOLD:ACE4009
Idaea subsericeata|Netherlands.South Holland|BOLD:ACE4009
Idaea subsericeata|Greece.Thessaly|BOLD:ACE4009
Idaea subsericeata|Italy.Calabria|BOLD:ACE4009
Idaea subsericeata|Italy.Sardinia|BOLD:ACE4010
Idaea subsericeata|Italy.Sardinia|BOLD:ACE4010
Idaea subsericeata|Italy.Sardinia|BOLD:ACE4010
Idaea subsericeata|Italy.Sardinia|BOLD:ACE4010

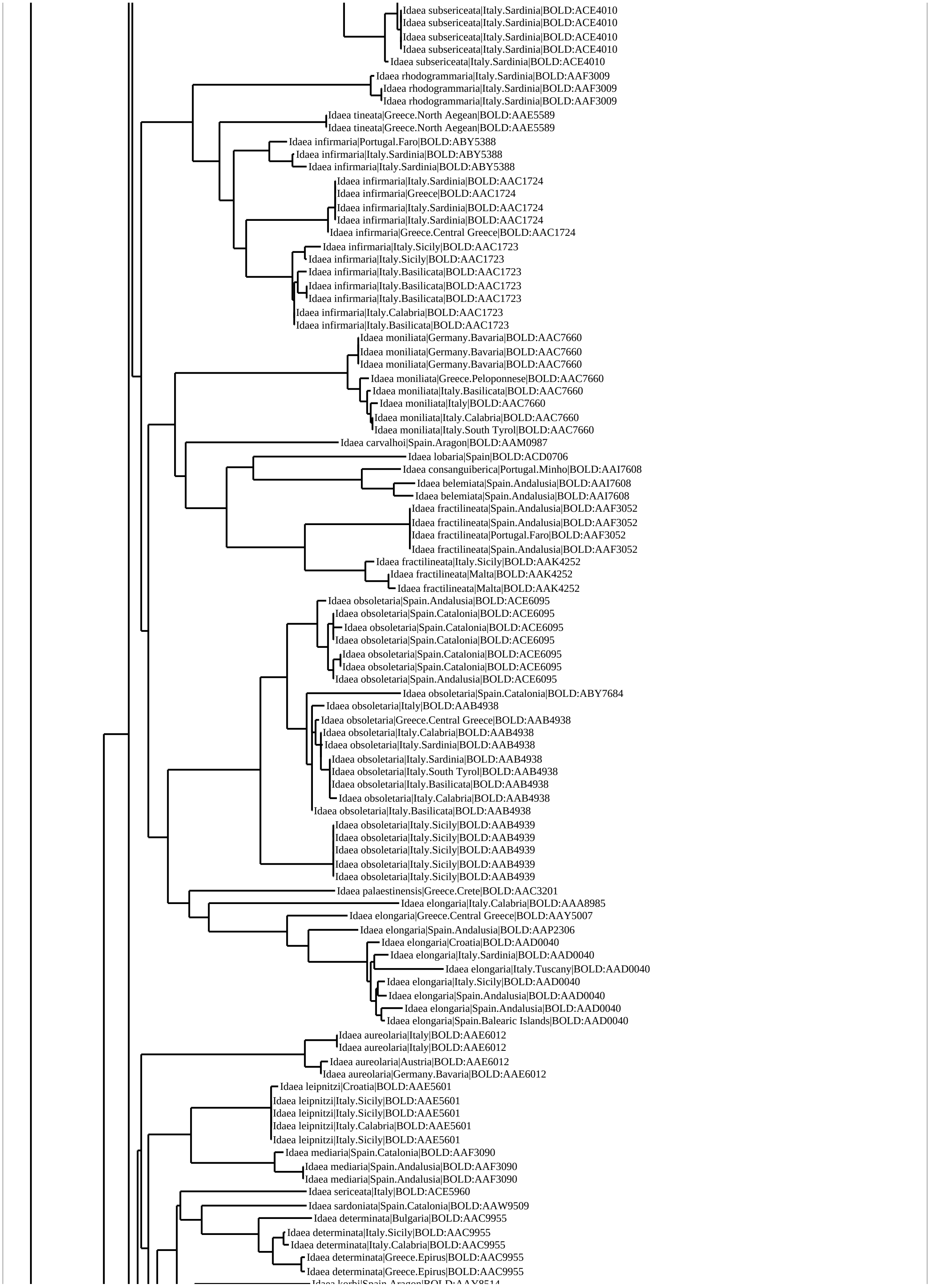

Idaea subsericeata|Italy.Sardinia|BOLD:ACE4010
Idaea subsericeata|Italy.Sardinia|BOLD:ACE4010
Idaea subsericeata|Italy.Sardinia|BOLD:ACE4010
Idaea subsericeata|Italy.Sardinia|BOLD:ACE4010
Idaea subsericeata|Italy.Sardinia|BOLD:ACE4010
Idaea rhodogrammaria|Italy.Sardinia|BOLD:AAF3009
Idaea rhodogrammaria|Italy.Sardinia|BOLD:AAF3009
Idaea rhodogrammaria|Italy.Sardinia|BOLD:AAF3009
Idaea tineata|Greece.North Aegean|BOLD:AAE5589
Idaea tineata|Greece.North Aegean|BOLD:AAE5589
Idaea infirmaria|Portugal.Faro|BOLD:ABY5388
Idaea infirmaria|Italy.Sardinia|BOLD:ABY5388
Idaea infirmaria|Italy.Sardinia|BOLD:ABY5388
Idaea infirmaria|Italy.Sardinia|BOLD:AAC1724
Idaea infirmaria|Greece|BOLD:AAC1724
Idaea infirmaria|Italy.Sardinia|BOLD:AAC1724
Idaea infirmaria|Italy.Sardinia|BOLD:AAC1724
Idaea infirmaria|Greece.Central Greece|BOLD:AAC1724
Idaea infirmaria|Italy.Sicily|BOLD:AAC1723
Idaea infirmaria|Italy.Sicily|BOLD:AAC1723
Idaea infirmaria|Italy.Basilicata|BOLD:AAC1723
Idaea infirmaria|Italy.Basilicata|BOLD:AAC1723
Idaea infirmaria|Italy.Basilicata|BOLD:AAC1723
Idaea infirmaria|Italy.Calabria|BOLD:AAC1723
Idaea infirmaria|Italy.Basilicata|BOLD:AAC1723
Idaea moniliata|Germany.Bavaria|BOLD:AAC7660
Idaea moniliata|Germany.Bavaria|BOLD:AAC7660
Idaea moniliata|Germany.Bavaria|BOLD:AAC7660
Idaea moniliata|Greece.Peloponnese|BOLD:AAC7660
Idaea moniliata|Italy.Basilicata|BOLD:AAC7660
Idaea moniliata|Italy|BOLD:AAC7660
Idaea moniliata|Italy.Calabria|BOLD:AAC7660
Idaea moniliata|Italy.South Tyrol|BOLD:AAC7660
Idaea carvalhoi|Spain.Aragon|BOLD:AAM0987
Idaea lobaria|Spain|BOLD:ACD0706
Idaea consanguiberica|Portugal.Minho|BOLD:AAI7608
Idaea belemiata|Spain.Andalusia|BOLD:AAI7608
Idaea belemiata|Spain.Andalusia|BOLD:AAI7608
Idaea fractilineata|Spain.Andalusia|BOLD:AAF3052
Idaea fractilineata|Spain.Andalusia|BOLD:AAF3052
Idaea fractilineata|Portugal.Faro|BOLD:AAF3052
Idaea fractilineata|Spain.Andalusia|BOLD:AAF3052
Idaea fractilineata|Italy.Sicily|BOLD:AAK4252
Idaea fractilineata|Malta|BOLD:AAK4252
Idaea fractilineata|Malta|BOLD:AAK4252
Idaea obsoletaria|Spain.Andalusia|BOLD:ACE6095
Idaea obsoletaria|Spain.Catalonia|BOLD:ACE6095
Idaea obsoletaria|Spain.Catalonia|BOLD:ACE6095
Idaea obsoletaria|Spain.Catalonia|BOLD:ACE6095
Idaea obsoletaria|Spain.Catalonia|BOLD:ACE6095
Idaea obsoletaria|Spain.Catalonia|BOLD:ACE6095
Idaea obsoletaria|Spain.Andalusia|BOLD:ACE6095
Idaea obsoletaria|Spain.Catalonia|BOLD:ABY7684
Idaea obsoletaria|Italy|BOLD:AAB4938
Idaea obsoletaria|Greece.Central Greece|BOLD:AAB4938
Idaea obsoletaria|Italy.Calabria|BOLD:AAB4938
Idaea obsoletaria|Italy.Sardinia|BOLD:AAB4938
Idaea obsoletaria|Italy.Sardinia|BOLD:AAB4938
Idaea obsoletaria|Italy.South Tyrol|BOLD:AAB4938
Idaea obsoletaria|Italy.Basilicata|BOLD:AAB4938
Idaea obsoletaria|Italy.Calabria|BOLD:AAB4938
Idaea obsoletaria|Italy.Basilicata|BOLD:AAB4938
Idaea obsoletaria|Italy.Sicily|BOLD:AAB4939
Idaea obsoletaria|Italy.Sicily|BOLD:AAB4939
Idaea obsoletaria|Italy.Sicily|BOLD:AAB4939
Idaea obsoletaria|Italy.Sicily|BOLD:AAB4939
Idaea obsoletaria|Italy.Sicily|BOLD:AAB4939
Idaea palaestinensis|Greece.Crete|BOLD:AAC3201
Idaea elongaria|Italy.Calabria|BOLD:AAA8985
Idaea elongaria|Greece.Central Greece|BOLD:AAY5007
Idaea elongaria|Spain.Andalusia|BOLD:AAP2306
Idaea elongaria|Croatia|BOLD:AAD0040
Idaea elongaria|Italy.Sardinia|BOLD:AAD0040
Idaea elongaria|Italy.Tuscany|BOLD:AAD0040
Idaea elongaria|Italy.Sicily|BOLD:AAD0040
Idaea elongaria|Spain.Andalusia|BOLD:AAD0040
Idaea elongaria|Spain.Andalusia|BOLD:AAD0040
Idaea elongaria|Spain.Balearic Islands|BOLD:AAD0040
Idaea aureolaria|Italy|BOLD:AAE6012
Idaea aureolaria|Italy|BOLD:AAE6012
Idaea aureolaria|Austria|BOLD:AAE6012
Idaea aureolaria|Germany.Bavaria|BOLD:AAE6012
Idaea leipnitzi|Croatia|BOLD:AAE5601
Idaea leipnitzi|Italy.Sicily|BOLD:AAE5601
Idaea leipnitzi|Italy.Sicily|BOLD:AAE5601
Idaea leipnitzi|Italy.Calabria|BOLD:AAE5601
Idaea leipnitzi|Italy.Sicily|BOLD:AAE5601
Idaea mediaria|Spain.Catalonia|BOLD:AAF3090
Idaea mediaria|Spain.Andalusia|BOLD:AAF3090
Idaea mediaria|Spain.Andalusia|BOLD:AAF3090
Idaea sericeata|Italy|BOLD:ACE5960
Idaea sardoniata|Spain.Catalonia|BOLD:AAW9509
Idaea determinata|Bulgaria|BOLD:AAC9955
Idaea determinata|Italy.Sicily|BOLD:AAC9955
Idaea determinata|Italy.Calabria|BOLD:AAC9955
Idaea determinata|Greece.Epirus|BOLD:AAC9955
Idaea determinata|Greece.Epirus|BOLD:AAC9955
Idaea korbi|Spain.Aragon|BOLD:AAY8514

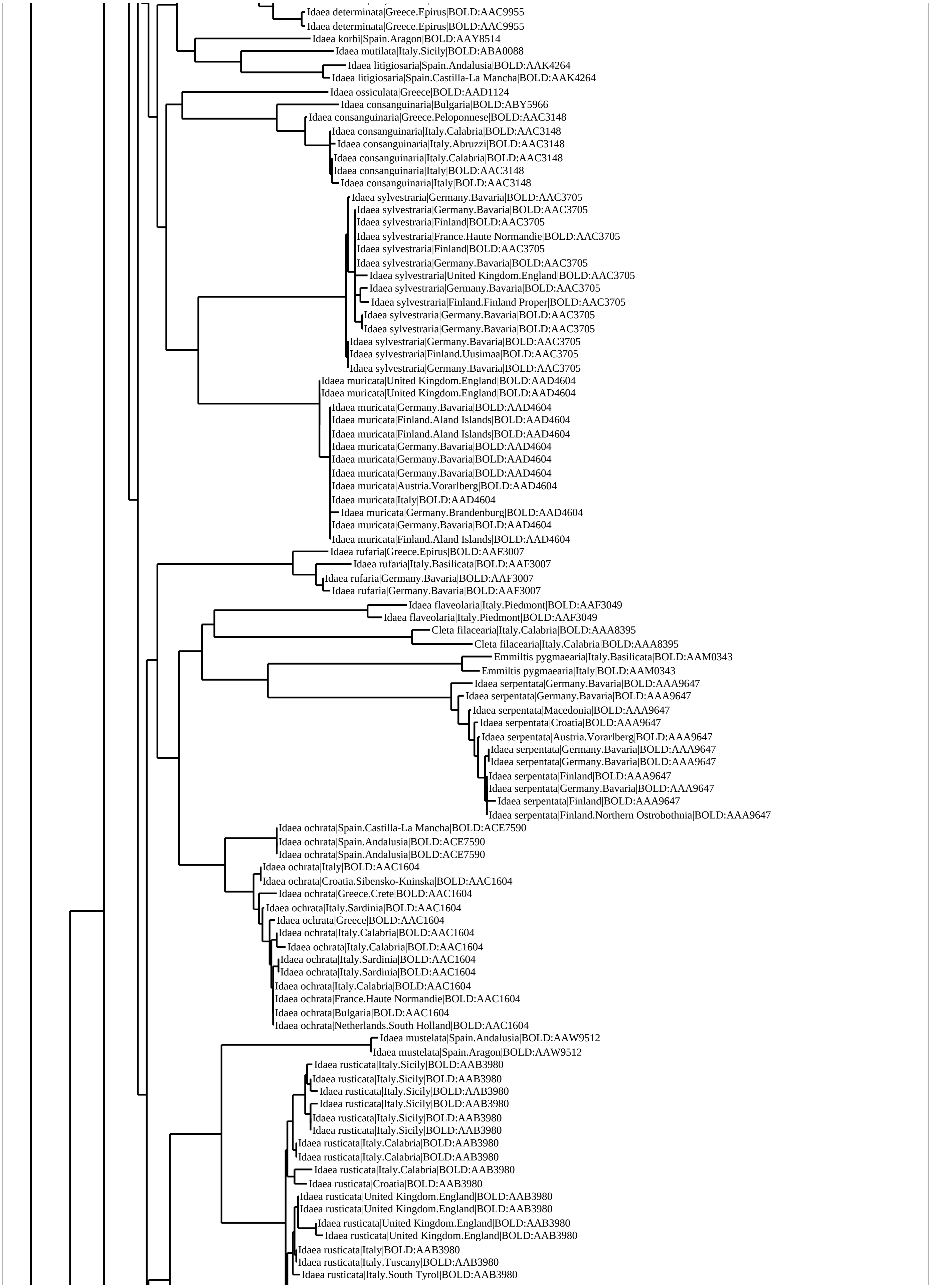
Idaea determinata|Greece.Epirus|BOLD:AAC9955
Idaea determinata|Greece.Epirus|BOLD:AAC9955
Idaea korbi|Spain.Aragon|BOLD:AAY8514
Idaea mutilata|Italy.Sicily|BOLD:ABA0088
Idaea litigiosaria|Spain.Andalusia|BOLD:AAK4264
Idaea litigiosaria|Spain.Castilla-La Mancha|BOLD:AAK4264
Idaea ossiculata|Greece|BOLD:AAD1124
Idaea consanguinaria|Bulgaria|BOLD:ABY5966
Idaea consanguinaria|Greece.Peloponnese|BOLD:AAC3148
Idaea consanguinaria|Italy.Calabria|BOLD:AAC3148
Idaea consanguinaria|Italy.Abruzzi|BOLD:AAC3148
Idaea consanguinaria|Italy.Calabria|BOLD:AAC3148
Idaea consanguinaria|Italy|BOLD:AAC3148
Idaea consanguinaria|Italy|BOLD:AAC3148
Idaea sylvestraria|Germany.Bavaria|BOLD:AAC3705
Idaea sylvestraria|Germany.Bavaria|BOLD:AAC3705
Idaea sylvestraria|Finland|BOLD:AAC3705
Idaea sylvestraria|France.Haute Normandie|BOLD:AAC3705
Idaea sylvestraria|Finland|BOLD:AAC3705
Idaea sylvestraria|Germany.Bavaria|BOLD:AAC3705
Idaea sylvestraria|United Kingdom.England|BOLD:AAC3705
Idaea sylvestraria|Germany.Bavaria|BOLD:AAC3705
Idaea sylvestraria|Finland.Finland Proper|BOLD:AAC3705
Idaea sylvestraria|Germany.Bavaria|BOLD:AAC3705
Idaea sylvestraria|Germany.Bavaria|BOLD:AAC3705
Idaea sylvestraria|Germany.Bavaria|BOLD:AAC3705
Idaea sylvestraria|Finland.Uusimaa|BOLD:AAC3705
Idaea sylvestraria|Germany.Bavaria|BOLD:AAC3705
Idaea muricata|United Kingdom.England|BOLD:AAD4604
Idaea muricata|United Kingdom.England|BOLD:AAD4604
Idaea muricata|Germany.Bavaria|BOLD:AAD4604
Idaea muricata|Finland.Aland Islands|BOLD:AAD4604
Idaea muricata|Finland.Aland Islands|BOLD:AAD4604
Idaea muricata|Germany.Bavaria|BOLD:AAD4604
Idaea muricata|Germany.Bavaria|BOLD:AAD4604
Idaea muricata|Germany.Bavaria|BOLD:AAD4604
Idaea muricata|Austria.Vorarlberg|BOLD:AAD4604
Idaea muricata|Italy|BOLD:AAD4604
Idaea muricata|Germany.Brandenburg|BOLD:AAD4604
Idaea muricata|Germany.Bavaria|BOLD:AAD4604
Idaea muricata|Finland.Aland Islands|BOLD:AAD4604
Idaea rufaria|Greece.Epirus|BOLD:AAF3007
Idaea rufaria|Italy.Basilicata|BOLD:AAF3007
Idaea rufaria|Germany.Bavaria|BOLD:AAF3007
Idaea rufaria|Germany.Bavaria|BOLD:AAF3007
Idaea flaveolaria|Italy.Piedmont|BOLD:AAF3049
Idaea flaveolaria|Italy.Piedmont|BOLD:AAF3049
Cleta filacearia|Italy.Calabria|BOLD:AAA8395
Cleta filacearia|Italy.Calabria|BOLD:AAA8395
Emmiltis pygmaearia|Italy.Basilicata|BOLD:AAM0343
Emmiltis pygmaearia|Italy|BOLD:AAM0343
Idaea serpentata|Germany.Bavaria|BOLD:AAA9647
Idaea serpentata|Germany.Bavaria|BOLD:AAA9647
Idaea serpentata|Macedonia|BOLD:AAA9647
Idaea serpentata|Croatia|BOLD:AAA9647
Idaea serpentata|Austria.Vorarlberg|BOLD:AAA9647
Idaea serpentata|Germany.Bavaria|BOLD:AAA9647
Idaea serpentata|Germany.Bavaria|BOLD:AAA9647
Idaea serpentata|Finland|BOLD:AAA9647
Idaea serpentata|Germany.Bavaria|BOLD:AAA9647
Idaea serpentata|Finland|BOLD:AAA9647
Idaea serpentata|Finland.Northern Ostrobothnia|BOLD:AAA9647
Idaea ochrata|Spain.Castilla-La Mancha|BOLD:ACE7590
Idaea ochrata|Spain.Andalusia|BOLD:ACE7590
Idaea ochrata|Spain.Andalusia|BOLD:ACE7590
Idaea ochrata|Italy|BOLD:AAC1604
Idaea ochrata|Croatia.Sibensko-Kninska|BOLD:AAC1604
Idaea ochrata|Greece.Crete|BOLD:AAC1604
Idaea ochrata|Italy.Sardinia|BOLD:AAC1604
Idaea ochrata|Greece|BOLD:AAC1604
Idaea ochrata|Italy.Calabria|BOLD:AAC1604
Idaea ochrata|Italy.Calabria|BOLD:AAC1604
Idaea ochrata|Italy.Sardinia|BOLD:AAC1604
Idaea ochrata|Italy.Sardinia|BOLD:AAC1604
Idaea ochrata|Italy.Calabria|BOLD:AAC1604
Idaea ochrata|France.Haute Normandie|BOLD:AAC1604
Idaea ochrata|Bulgaria|BOLD:AAC1604
Idaea ochrata|Netherlands.South Holland|BOLD:AAC1604
Idaea mustelata|Spain.Andalusia|BOLD:AAW9512
Idaea mustelata|Spain.Aragon|BOLD:AAW9512
Idaea rusticata|Italy.Sicily|BOLD:AAB3980
Idaea rusticata|Italy.Sicily|BOLD:AAB3980
Idaea rusticata|Italy.Sicily|BOLD:AAB3980
Idaea rusticata|Italy.Sicily|BOLD:AAB3980
Idaea rusticata|Italy.Sicily|BOLD:AAB3980
Idaea rusticata|Italy.Sicily|BOLD:AAB3980
Idaea rusticata|Italy.Calabria|BOLD:AAB3980
Idaea rusticata|Italy.Calabria|BOLD:AAB3980
Idaea rusticata|Italy.Calabria|BOLD:AAB3980
Idaea rusticata|Croatia|BOLD:AAB3980
Idaea rusticata|United Kingdom.England|BOLD:AAB3980
Idaea rusticata|United Kingdom.England|BOLD:AAB3980
Idaea rusticata|United Kingdom.England|BOLD:AAB3980
Idaea rusticata|United Kingdom.England|BOLD:AAB3980
Idaea rusticata|Italy|BOLD:AAB3980
Idaea rusticata|Italy.Tuscany|BOLD:AAB3980
Idaea rusticata|Italy.South Tyrol|BOLD:AAB3980

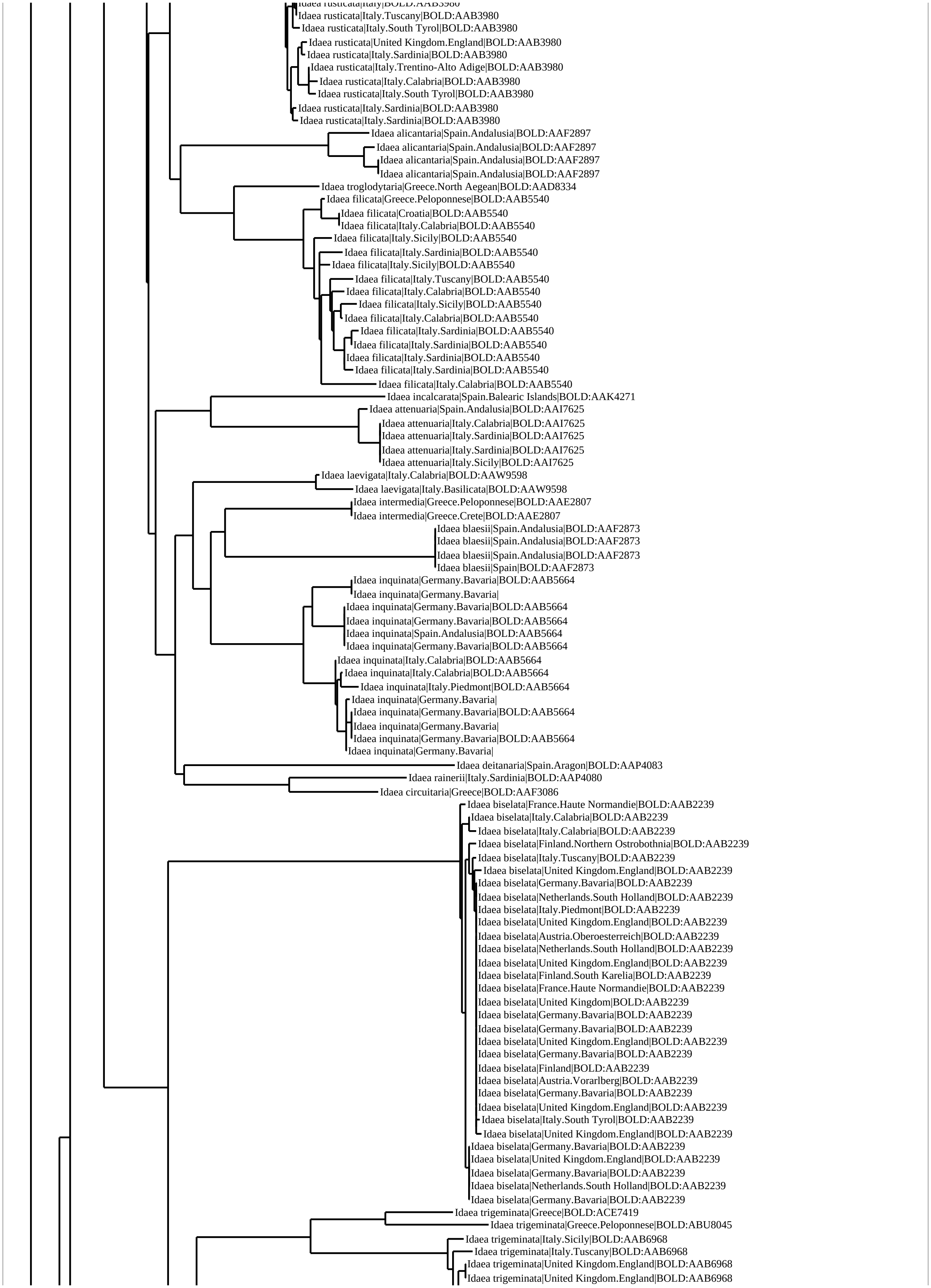
Idaea rusticata|Italy.Tuscany|BOLD:AAB3980
Idaea rusticata|Italy.South Tyrol|BOLD:AAB3980
Idaea rusticata|United Kingdom.England|BOLD:AAB3980
Idaea rusticata|Italy.Sardinia|BOLD:AAB3980
Idaea rusticata|Italy.Trentino-Alto Adige|BOLD:AAB3980
Idaea rusticata|Italy.Calabria|BOLD:AAB3980
Idaea rusticata|Italy.South Tyrol|BOLD:AAB3980
Idaea rusticata|Italy.Sardinia|BOLD:AAB3980
Idaea rusticata|Italy.Sardinia|BOLD:AAB3980
Idaea alicantaria|Spain.Andalusia|BOLD:AAF2897
Idaea alicantaria|Spain.Andalusia|BOLD:AAF2897
Idaea alicantaria|Spain.Andalusia|BOLD:AAF2897
Idaea alicantaria|Spain.Andalusia|BOLD:AAF2897
Idaea troglodytaria|Greece.North Aegean|BOLD:AAD8334
Idaea filicata|Greece.Peloponnese|BOLD:AAB5540
Idaea filicata|Croatia|BOLD:AAB5540
Idaea filicata|Italy.Calabria|BOLD:AAB5540
Idaea filicata|Italy.Sicily|BOLD:AAB5540
Idaea filicata|Italy.Sardinia|BOLD:AAB5540
Idaea filicata|Italy.Sicily|BOLD:AAB5540
Idaea filicata|Italy.Tuscany|BOLD:AAB5540
Idaea filicata|Italy.Calabria|BOLD:AAB5540
Idaea filicata|Italy.Sicily|BOLD:AAB5540
Idaea filicata|Italy.Calabria|BOLD:AAB5540
Idaea filicata|Italy.Sardinia|BOLD:AAB5540
Idaea filicata|Italy.Sardinia|BOLD:AAB5540
Idaea filicata|Italy.Sardinia|BOLD:AAB5540
Idaea filicata|Italy.Sardinia|BOLD:AAB5540
Idaea filicata|Italy.Calabria|BOLD:AAB5540
Idaea incalcarata|Spain.Balearic Islands|BOLD:AAK4271
Idaea attenuaria|Spain.Andalusia|BOLD:AAI7625
Idaea attenuaria|Italy.Calabria|BOLD:AAI7625
Idaea attenuaria|Italy.Sardinia|BOLD:AAI7625
Idaea attenuaria|Italy.Sardinia|BOLD:AAI7625
Idaea attenuaria|Italy.Sicily|BOLD:AAI7625
Idaea laevigata|Italy.Calabria|BOLD:AAW9598
Idaea laevigata|Italy.Basilicata|BOLD:AAW9598
Idaea intermedia|Greece.Peloponnese|BOLD:AAE2807
Idaea intermedia|Greece.Crete|BOLD:AAE2807
Idaea blaesii|Spain.Andalusia|BOLD:AAF2873
Idaea blaesii|Spain.Andalusia|BOLD:AAF2873
Idaea blaesii|Spain.Andalusia|BOLD:AAF2873
Idaea blaesii|Spain|BOLD:AAF2873
Idaea inquinata|Germany.Bavaria|BOLD:AAB5664
Idaea inquinata|Germany.Bavaria|
Idaea inquinata|Germany.Bavaria|BOLD:AAB5664
Idaea inquinata|Germany.Bavaria|BOLD:AAB5664
Idaea inquinata|Spain.Andalusia|BOLD:AAB5664
Idaea inquinata|Germany.Bavaria|BOLD:AAB5664
Idaea inquinata|Italy.Calabria|BOLD:AAB5664
Idaea inquinata|Italy.Calabria|BOLD:AAB5664
Idaea inquinata|Italy.Piedmont|BOLD:AAB5664
Idaea inquinata|Germany.Bavaria|
Idaea inquinata|Germany.Bavaria|BOLD:AAB5664
Idaea inquinata|Germany.Bavaria|
Idaea inquinata|Germany.Bavaria|BOLD:AAB5664
Idaea inquinata|Germany.Bavaria|
Idaea deitanaria|Spain.Aragon|BOLD:AAP4083
Idaea rainerii|Italy.Sardinia|BOLD:AAP4080
Idaea circuitaria|Greece|BOLD:AAF3086
Idaea biselata|France.Haute Normandie|BOLD:AAB2239
Idaea biselata|Italy.Calabria|BOLD:AAB2239
Idaea biselata|Italy.Calabria|BOLD:AAB2239
Idaea biselata|Finland.Northern Ostrobothnia|BOLD:AAB2239
Idaea biselata|Italy.Tuscany|BOLD:AAB2239
Idaea biselata|United Kingdom.England|BOLD:AAB2239
Idaea biselata|Germany.Bavaria|BOLD:AAB2239
Idaea biselata|Netherlands.South Holland|BOLD:AAB2239
Idaea biselata|Italy.Piedmont|BOLD:AAB2239
Idaea biselata|United Kingdom.England|BOLD:AAB2239
Idaea biselata|Austria.Oberoesterreich|BOLD:AAB2239
Idaea biselata|Netherlands.South Holland|BOLD:AAB2239
Idaea biselata|United Kingdom.England|BOLD:AAB2239
Idaea biselata|Finland.South Karelia|BOLD:AAB2239
Idaea biselata|France.Haute Normandie|BOLD:AAB2239
Idaea biselata|United Kingdom|BOLD:AAB2239
Idaea biselata|Germany.Bavaria|BOLD:AAB2239
Idaea biselata|Germany.Bavaria|BOLD:AAB2239
Idaea biselata|United Kingdom.England|BOLD:AAB2239
Idaea biselata|Germany.Bavaria|BOLD:AAB2239
Idaea biselata|Finland|BOLD:AAB2239
Idaea biselata|Austria.Vorarlberg|BOLD:AAB2239
Idaea biselata|Germany.Bavaria|BOLD:AAB2239
Idaea biselata|United Kingdom.England|BOLD:AAB2239
Idaea biselata|Italy.South Tyrol|BOLD:AAB2239
Idaea biselata|United Kingdom.England|BOLD:AAB2239
Idaea biselata|Germany.Bavaria|BOLD:AAB2239
Idaea biselata|United Kingdom.England|BOLD:AAB2239
Idaea biselata|Germany.Bavaria|BOLD:AAB2239
Idaea biselata|Netherlands.South Holland|BOLD:AAB2239
Idaea biselata|Germany.Bavaria|BOLD:AAB2239
Idaea trigeminata|Greece|BOLD:ACE7419
Idaea trigeminata|Greece.Peloponnese|BOLD:ABU8045
Idaea trigeminata|Italy.Sicily|BOLD:AAB6968
Idaea trigeminata|Italy.Tuscany|BOLD:AAB6968
Idaea trigeminata|United Kingdom.England|BOLD:AAB6968
Idaea trigeminata|United Kingdom.England|BOLD:AAB6968

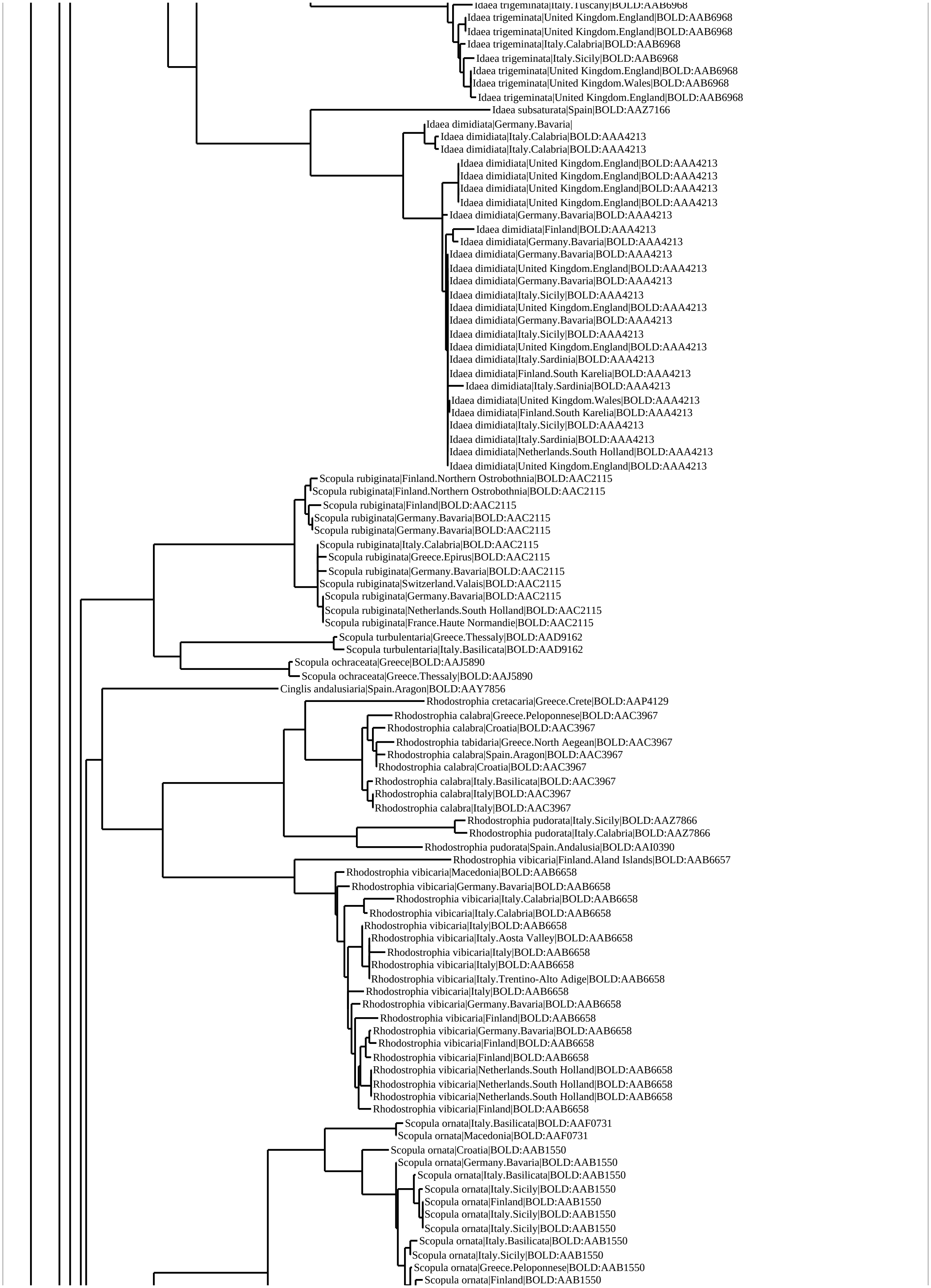
Idaea trigeminata|Italy.Tuscany|BOLD:AAB6968
Idaea trigeminata|United Kingdom.England|BOLD:AAB6968
Idaea trigeminata|United Kingdom.England|BOLD:AAB6968
Idaea trigeminata|Italy.Calabria|BOLD:AAB6968
Idaea trigeminata|Italy.Sicily|BOLD:AAB6968
Idaea trigeminata|United Kingdom.England|BOLD:AAB6968
Idaea trigeminata|United Kingdom.Wales|BOLD:AAB6968
Idaea trigeminata|United Kingdom.England|BOLD:AAB6968
Idaea subsaturata|Spain|BOLD:AAZ7166
Idaea dimidiata|Germany.Bavaria|
Idaea dimidiata|Italy.Calabria|BOLD:AAA4213
Idaea dimidiata|Italy.Calabria|BOLD:AAA4213
Idaea dimidiata|United Kingdom.England|BOLD:AAA4213
Idaea dimidiata|United Kingdom.England|BOLD:AAA4213
Idaea dimidiata|United Kingdom.England|BOLD:AAA4213
Idaea dimidiata|United Kingdom.England|BOLD:AAA4213
Idaea dimidiata|Germany.Bavaria|BOLD:AAA4213
Idaea dimidiata|Finland|BOLD:AAA4213
Idaea dimidiata|Germany.Bavaria|BOLD:AAA4213
Idaea dimidiata|Germany.Bavaria|BOLD:AAA4213
Idaea dimidiata|United Kingdom.England|BOLD:AAA4213
Idaea dimidiata|Germany.Bavaria|BOLD:AAA4213
Idaea dimidiata|Italy.Sicily|BOLD:AAA4213
Idaea dimidiata|United Kingdom.England|BOLD:AAA4213
Idaea dimidiata|Germany.Bavaria|BOLD:AAA4213
Idaea dimidiata|Italy.Sicily|BOLD:AAA4213
Idaea dimidiata|United Kingdom.England|BOLD:AAA4213
Idaea dimidiata|Italy.Sardinia|BOLD:AAA4213
Idaea dimidiata|Finland.South Karelia|BOLD:AAA4213
Idaea dimidiata|Italy.Sardinia|BOLD:AAA4213
Idaea dimidiata|United Kingdom.Wales|BOLD:AAA4213
Idaea dimidiata|Finland.South Karelia|BOLD:AAA4213
Idaea dimidiata|Italy.Sicily|BOLD:AAA4213
Idaea dimidiata|Italy.Sardinia|BOLD:AAA4213
Idaea dimidiata|Netherlands.South Holland|BOLD:AAA4213
Idaea dimidiata|United Kingdom.England|BOLD:AAA4213
Scopula rubiginata|Finland.Northern Ostrobothnia|BOLD:AAC2115
Scopula rubiginata|Finland.Northern Ostrobothnia|BOLD:AAC2115
Scopula rubiginata|Finland|BOLD:AAC2115
Scopula rubiginata|Germany.Bavaria|BOLD:AAC2115
Scopula rubiginata|Germany.Bavaria|BOLD:AAC2115
Scopula rubiginata|Italy.Calabria|BOLD:AAC2115
Scopula rubiginata|Greece.Epirus|BOLD:AAC2115
Scopula rubiginata|Germany.Bavaria|BOLD:AAC2115
Scopula rubiginata|Switzerland.Valais|BOLD:AAC2115
Scopula rubiginata|Germany.Bavaria|BOLD:AAC2115
Scopula rubiginata|Netherlands.South Holland|BOLD:AAC2115
Scopula rubiginata|France.Haute Normandie|BOLD:AAC2115
Scopula turbulentaria|Greece.Thessaly|BOLD:AAD9162
Scopula turbulentaria|Italy.Basilicata|BOLD:AAD9162
Scopula ochraceata|Greece|BOLD:AAJ5890
Scopula ochraceata|Greece.Thessaly|BOLD:AAJ5890
Cinglis andalusiaria|Spain.Aragon|BOLD:AAY7856
Rhodostrophia cretacaria|Greece.Crete|BOLD:AAP4129
Rhodostrophia calabra|Greece.Peloponnese|BOLD:AAC3967
Rhodostrophia calabra|Croatia|BOLD:AAC3967
Rhodostrophia tabidaria|Greece.North Aegean|BOLD:AAC3967
Rhodostrophia calabra|Spain.Aragon|BOLD:AAC3967
Rhodostrophia calabra|Croatia|BOLD:AAC3967
Rhodostrophia calabra|Italy.Basilicata|BOLD:AAC3967
Rhodostrophia calabra|Italy|BOLD:AAC3967
Rhodostrophia calabra|Italy|BOLD:AAC3967
Rhodostrophia pudorata|Italy.Sicily|BOLD:AAZ7866
Rhodostrophia pudorata|Italy.Calabria|BOLD:AAZ7866
Rhodostrophia pudorata|Spain.Andalusia|BOLD:AAI0390
Rhodostrophia vibicaria|Finland.Aland Islands|BOLD:AAB6657
Rhodostrophia vibicaria|Macedonia|BOLD:AAB6658
Rhodostrophia vibicaria|Germany.Bavaria|BOLD:AAB6658
Rhodostrophia vibicaria|Italy.Calabria|BOLD:AAB6658
Rhodostrophia vibicaria|Italy.Calabria|BOLD:AAB6658
Rhodostrophia vibicaria|Italy|BOLD:AAB6658
Rhodostrophia vibicaria|Italy.Aosta Valley|BOLD:AAB6658
Rhodostrophia vibicaria|Italy|BOLD:AAB6658
Rhodostrophia vibicaria|Italy|BOLD:AAB6658
Rhodostrophia vibicaria|Italy.Trentino-Alto Adige|BOLD:AAB6658
Rhodostrophia vibicaria|Italy|BOLD:AAB6658
Rhodostrophia vibicaria|Germany.Bavaria|BOLD:AAB6658
Rhodostrophia vibicaria|Finland|BOLD:AAB6658
Rhodostrophia vibicaria|Germany.Bavaria|BOLD:AAB6658
Rhodostrophia vibicaria|Finland|BOLD:AAB6658
Rhodostrophia vibicaria|Finland|BOLD:AAB6658
Rhodostrophia vibicaria|Netherlands.South Holland|BOLD:AAB6658
Rhodostrophia vibicaria|Netherlands.South Holland|BOLD:AAB6658
Rhodostrophia vibicaria|Netherlands.South Holland|BOLD:AAB6658
Rhodostrophia vibicaria|Finland|BOLD:AAB6658
Scopula ornata|Italy.Basilicata|BOLD:AAF0731
Scopula ornata|Macedonia|BOLD:AAF0731
Scopula ornata|Croatia|BOLD:AAB1550
Scopula ornata|Germany.Bavaria|BOLD:AAB1550
Scopula ornata|Italy.Basilicata|BOLD:AAB1550
Scopula ornata|Italy.Sicily|BOLD:AAB1550
Scopula ornata|Finland|BOLD:AAB1550
Scopula ornata|Italy.Sicily|BOLD:AAB1550
Scopula ornata|Italy.Sicily|BOLD:AAB1550
Scopula ornata|Italy.Basilicata|BOLD:AAB1550
Scopula ornata|Italy.Sicily|BOLD:AAB1550
Scopula ornata|Greece.Peloponnese|BOLD:AAB1550
Scopula ornata|Finland|BOLD:AAB1550

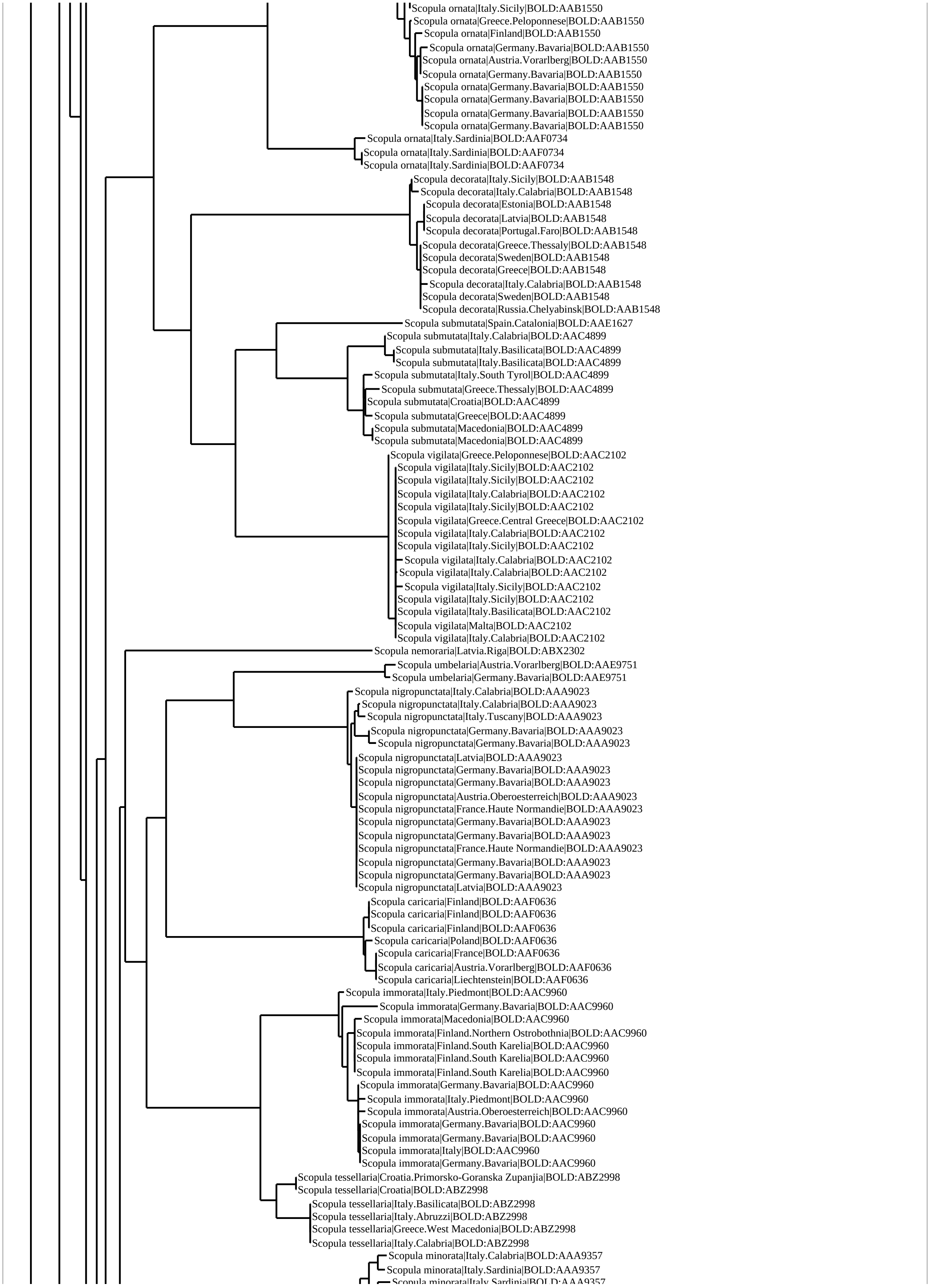
Scopula ornata|Italy.Sicily|BOLD:AAB1550
Scopula ornata|Greece.Peloponnese|BOLD:AAB1550
Scopula ornata|Finland|BOLD:AAB1550
Scopula ornata|Germany.Bavaria|BOLD:AAB1550
Scopula ornata|Austria.Vorarlberg|BOLD:AAB1550
Scopula ornata|Germany.Bavaria|BOLD:AAB1550
Scopula ornata|Germany.Bavaria|BOLD:AAB1550
Scopula ornata|Germany.Bavaria|BOLD:AAB1550
Scopula ornata|Germany.Bavaria|BOLD:AAB1550
Scopula ornata|Germany.Bavaria|BOLD:AAB1550
Scopula ornata|Italy.Sardinia|BOLD:AAF0734
Scopula ornata|Italy.Sardinia|BOLD:AAF0734
Scopula ornata|Italy.Sardinia|BOLD:AAF0734
Scopula decorata|Italy.Sicily|BOLD:AAB1548
Scopula decorata|Italy.Calabria|BOLD:AAB1548
Scopula decorata|Estonia|BOLD:AAB1548
Scopula decorata|Latvia|BOLD:AAB1548
Scopula decorata|Portugal.Faro|BOLD:AAB1548
Scopula decorata|Greece.Thessaly|BOLD:AAB1548
Scopula decorata|Sweden|BOLD:AAB1548
Scopula decorata|Greece|BOLD:AAB1548
Scopula decorata|Italy.Calabria|BOLD:AAB1548
Scopula decorata|Sweden|BOLD:AAB1548
Scopula decorata|Russia.Chelyabinsk|BOLD:AAB1548
Scopula submutata|Spain.Catalonia|BOLD:AAE1627
Scopula submutata|Italy.Calabria|BOLD:AAC4899
Scopula submutata|Italy.Basilicata|BOLD:AAC4899
Scopula submutata|Italy.Basilicata|BOLD:AAC4899
Scopula submutata|Italy.South Tyrol|BOLD:AAC4899
Scopula submutata|Greece.Thessaly|BOLD:AAC4899
Scopula submutata|Croatia|BOLD:AAC4899
Scopula submutata|Greece|BOLD:AAC4899
Scopula submutata|Macedonia|BOLD:AAC4899
Scopula submutata|Macedonia|BOLD:AAC4899
Scopula vigilata|Greece.Peloponnese|BOLD:AAC2102
Scopula vigilata|Italy.Sicily|BOLD:AAC2102
Scopula vigilata|Italy.Sicily|BOLD:AAC2102
Scopula vigilata|Italy.Calabria|BOLD:AAC2102
Scopula vigilata|Italy.Sicily|BOLD:AAC2102
Scopula vigilata|Greece.Central Greece|BOLD:AAC2102
Scopula vigilata|Italy.Calabria|BOLD:AAC2102
Scopula vigilata|Italy.Sicily|BOLD:AAC2102
Scopula vigilata|Italy.Calabria|BOLD:AAC2102
Scopula vigilata|Italy.Calabria|BOLD:AAC2102
Scopula vigilata|Italy.Sicily|BOLD:AAC2102
Scopula vigilata|Italy.Sicily|BOLD:AAC2102
Scopula vigilata|Italy.Basilicata|BOLD:AAC2102
Scopula vigilata|Malta|BOLD:AAC2102
Scopula vigilata|Italy.Calabria|BOLD:AAC2102
Scopula nemoraria|Latvia.Riga|BOLD:ABX2302
Scopula umbelaria|Austria.Vorarlberg|BOLD:AAE9751
Scopula umbelaria|Germany.Bavaria|BOLD:AAE9751
Scopula nigropunctata|Italy.Calabria|BOLD:AAA9023
Scopula nigropunctata|Italy.Calabria|BOLD:AAA9023
Scopula nigropunctata|Italy.Tuscany|BOLD:AAA9023
Scopula nigropunctata|Germany.Bavaria|BOLD:AAA9023
Scopula nigropunctata|Germany.Bavaria|BOLD:AAA9023
Scopula nigropunctata|Latvia|BOLD:AAA9023
Scopula nigropunctata|Germany.Bavaria|BOLD:AAA9023
Scopula nigropunctata|Germany.Bavaria|BOLD:AAA9023
Scopula nigropunctata|Austria.Oberoesterreich|BOLD:AAA9023
Scopula nigropunctata|France.Haute Normandie|BOLD:AAA9023
Scopula nigropunctata|Germany.Bavaria|BOLD:AAA9023
Scopula nigropunctata|Germany.Bavaria|BOLD:AAA9023
Scopula nigropunctata|France.Haute Normandie|BOLD:AAA9023
Scopula nigropunctata|Germany.Bavaria|BOLD:AAA9023
Scopula nigropunctata|Germany.Bavaria|BOLD:AAA9023
Scopula nigropunctata|Latvia|BOLD:AAA9023
Scopula caricaria|Finland|BOLD:AAF0636
Scopula caricaria|Finland|BOLD:AAF0636
Scopula caricaria|Finland|BOLD:AAF0636
Scopula caricaria|Poland|BOLD:AAF0636
Scopula caricaria|France|BOLD:AAF0636
Scopula caricaria|Austria.Vorarlberg|BOLD:AAF0636
Scopula caricaria|Liechtenstein|BOLD:AAF0636
Scopula immorata|Italy.Piedmont|BOLD:AAC9960
Scopula immorata|Germany.Bavaria|BOLD:AAC9960
Scopula immorata|Macedonia|BOLD:AAC9960
Scopula immorata|Finland.Northern Ostrobothnia|BOLD:AAC9960
Scopula immorata|Finland.South Karelia|BOLD:AAC9960
Scopula immorata|Finland.South Karelia|BOLD:AAC9960
Scopula immorata|Finland.South Karelia|BOLD:AAC9960
Scopula immorata|Germany.Bavaria|BOLD:AAC9960
Scopula immorata|Italy.Piedmont|BOLD:AAC9960
Scopula immorata|Austria.Oberoesterreich|BOLD:AAC9960
Scopula immorata|Germany.Bavaria|BOLD:AAC9960
Scopula immorata|Germany.Bavaria|BOLD:AAC9960
Scopula immorata|Italy|BOLD:AAC9960
Scopula immorata|Germany.Bavaria|BOLD:AAC9960
Scopula tessellaria|Croatia.Primorsko-Goranska Zupanjia|BOLD:ABZ2998
Scopula tessellaria|Croatia|BOLD:ABZ2998
Scopula tessellaria|Italy.Basilicata|BOLD:ABZ2998
Scopula tessellaria|Italy.Abruzzi|BOLD:ABZ2998
Scopula tessellaria|Greece.West Macedonia|BOLD:ABZ2998
Scopula tessellaria|Italy.Calabria|BOLD:ABZ2998
Scopula minorata|Italy.Calabria|BOLD:AAA9357
Scopula minorata|Italy.Sardinia|BOLD:AAA9357
Scopula minorata|Italy.Sardinia|BOLD:AAA9357

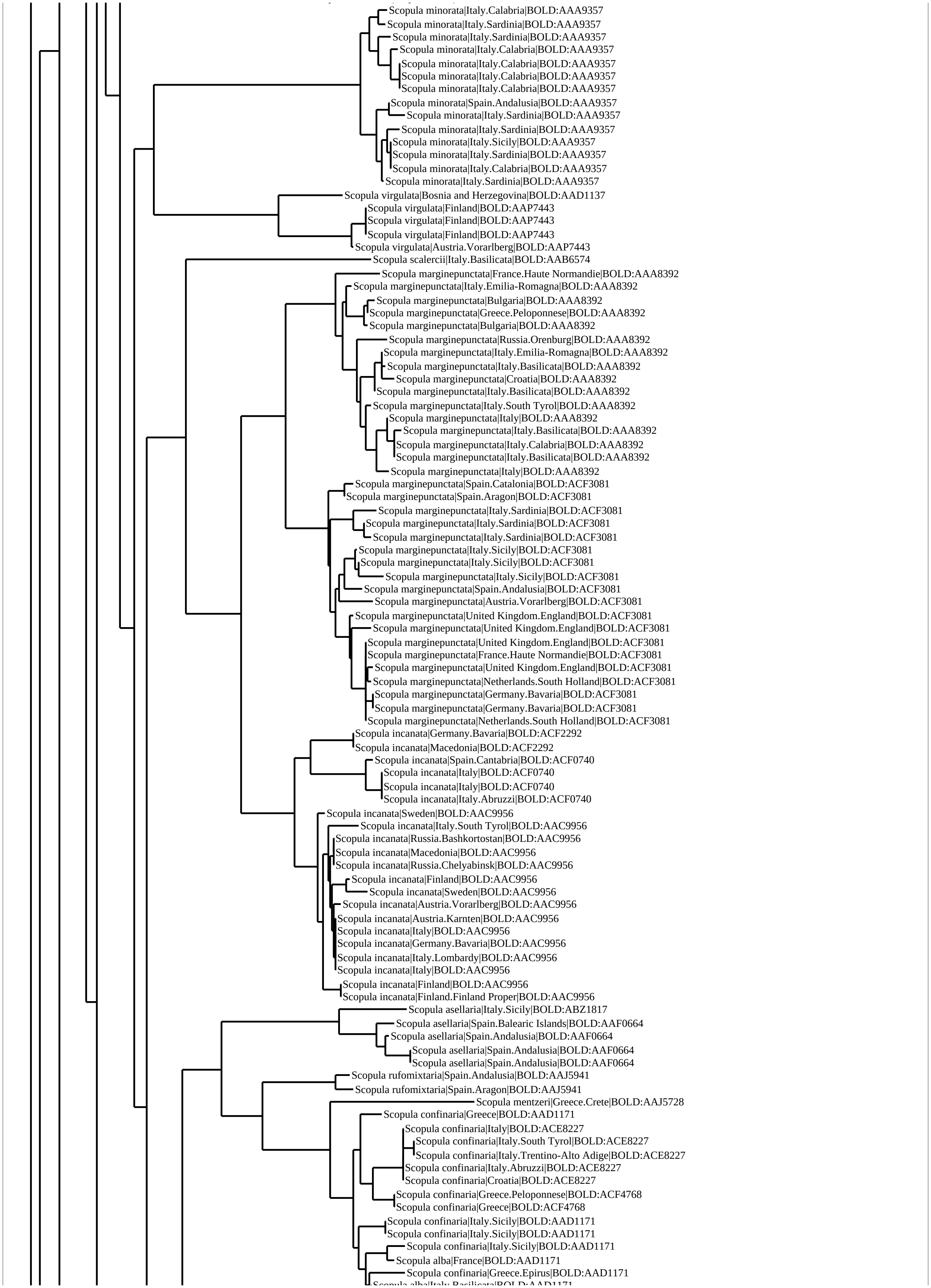
Scopula minorata|Italy.Calabria|BOLD:AAA9357
Scopula minorata|Italy.Sardinia|BOLD:AAA9357
Scopula minorata|Italy.Sardinia|BOLD:AAA9357
Scopula minorata|Italy.Calabria|BOLD:AAA9357
Scopula minorata|Italy.Calabria|BOLD:AAA9357
Scopula minorata|Italy.Calabria|BOLD:AAA9357
Scopula minorata|Italy.Calabria|BOLD:AAA9357
Scopula minorata|Spain.Andalusia|BOLD:AAA9357
Scopula minorata|Italy.Sardinia|BOLD:AAA9357
Scopula minorata|Italy.Sardinia|BOLD:AAA9357
Scopula minorata|Italy.Sicily|BOLD:AAA9357
Scopula minorata|Italy.Sardinia|BOLD:AAA9357
Scopula minorata|Italy.Calabria|BOLD:AAA9357
Scopula minorata|Italy.Sardinia|BOLD:AAA9357
Scopula virgulata|Bosnia and Herzegovina|BOLD:AAD1137
Scopula virgulata|Finland|BOLD:AAP7443
Scopula virgulata|Finland|BOLD:AAP7443
Scopula virgulata|Finland|BOLD:AAP7443
Scopula virgulata|Austria.Vorarlberg|BOLD:AAP7443
Scopula scalercii|Italy.Basilicata|BOLD:AAB6574
Scopula marginepunctata|France.Haute Normandie|BOLD:AAA8392
Scopula marginepunctata|Italy.Emilia-Romagna|BOLD:AAA8392
Scopula marginepunctata|Bulgaria|BOLD:AAA8392
Scopula marginepunctata|Greece.Peloponnese|BOLD:AAA8392
Scopula marginepunctata|Bulgaria|BOLD:AAA8392
Scopula marginepunctata|Russia.Orenburg|BOLD:AAA8392
Scopula marginepunctata|Italy.Emilia-Romagna|BOLD:AAA8392
Scopula marginepunctata|Italy.Basilicata|BOLD:AAA8392
Scopula marginepunctata|Croatia|BOLD:AAA8392
Scopula marginepunctata|Italy.Basilicata|BOLD:AAA8392
Scopula marginepunctata|Italy.South Tyrol|BOLD:AAA8392
Scopula marginepunctata|Italy|BOLD:AAA8392
Scopula marginepunctata|Italy.Basilicata|BOLD:AAA8392
Scopula marginepunctata|Italy.Calabria|BOLD:AAA8392
Scopula marginepunctata|Italy.Basilicata|BOLD:AAA8392
Scopula marginepunctata|Italy|BOLD:AAA8392
Scopula marginepunctata|Spain.Catalonia|BOLD:ACF3081
Scopula marginepunctata|Spain.Aragon|BOLD:ACF3081
Scopula marginepunctata|Italy.Sardinia|BOLD:ACF3081
Scopula marginepunctata|Italy.Sardinia|BOLD:ACF3081
Scopula marginepunctata|Italy.Sardinia|BOLD:ACF3081
Scopula marginepunctata|Italy.Sicily|BOLD:ACF3081
Scopula marginepunctata|Italy.Sicily|BOLD:ACF3081
Scopula marginepunctata|Italy.Sicily|BOLD:ACF3081
Scopula marginepunctata|Spain.Andalusia|BOLD:ACF3081
Scopula marginepunctata|Austria.Vorarlberg|BOLD:ACF3081
Scopula marginepunctata|United Kingdom.England|BOLD:ACF3081
Scopula marginepunctata|United Kingdom.England|BOLD:ACF3081
Scopula marginepunctata|United Kingdom.England|BOLD:ACF3081
Scopula marginepunctata|France.Haute Normandie|BOLD:ACF3081
Scopula marginepunctata|United Kingdom.England|BOLD:ACF3081
Scopula marginepunctata|Netherlands.South Holland|BOLD:ACF3081
Scopula marginepunctata|Germany.Bavaria|BOLD:ACF3081
Scopula marginepunctata|Germany.Bavaria|BOLD:ACF3081
Scopula marginepunctata|Netherlands.South Holland|BOLD:ACF3081
Scopula incanata|Germany.Bavaria|BOLD:ACF2292
Scopula incanata|Macedonia|BOLD:ACF2292
Scopula incanata|Spain.Cantabria|BOLD:ACF0740
Scopula incanata|Italy|BOLD:ACF0740
Scopula incanata|Italy|BOLD:ACF0740
Scopula incanata|Italy.Abruzzi|BOLD:ACF0740
Scopula incanata|Sweden|BOLD:AAC9956
Scopula incanata|Italy.South Tyrol|BOLD:AAC9956
Scopula incanata|Russia.Bashkortostan|BOLD:AAC9956
Scopula incanata|Macedonia|BOLD:AAC9956
Scopula incanata|Russia.Chelyabinsk|BOLD:AAC9956
Scopula incanata|Finland|BOLD:AAC9956
Scopula incanata|Sweden|BOLD:AAC9956
Scopula incanata|Austria.Vorarlberg|BOLD:AAC9956
Scopula incanata|Austria.Karnten|BOLD:AAC9956
Scopula incanata|Italy|BOLD:AAC9956
Scopula incanata|Germany.Bavaria|BOLD:AAC9956
Scopula incanata|Italy.Lombardy|BOLD:AAC9956
Scopula incanata|Italy|BOLD:AAC9956
Scopula incanata|Finland|BOLD:AAC9956
Scopula incanata|Finland.Finland Proper|BOLD:AAC9956
Scopula asellaria|Italy.Sicily|BOLD:ABZ1817
Scopula asellaria|Spain.Balearic Islands|BOLD:AAF0664
Scopula asellaria|Spain.Andalusia|BOLD:AAF0664
Scopula asellaria|Spain.Andalusia|BOLD:AAF0664
Scopula asellaria|Spain.Andalusia|BOLD:AAF0664
Scopula rufomixtaria|Spain.Andalusia|BOLD:AAJ5941
Scopula rufomixtaria|Spain.Aragon|BOLD:AAJ5941
Scopula mentzeri|Greece.Crete|BOLD:AAJ5728
Scopula confinaria|Greece|BOLD:AAD1171
Scopula confinaria|Italy|BOLD:ACE8227
Scopula confinaria|Italy.South Tyrol|BOLD:ACE8227
Scopula confinaria|Italy.Trentino-Alto Adige|BOLD:ACE8227
Scopula confinaria|Italy.Abruzzi|BOLD:ACE8227
Scopula confinaria|Croatia|BOLD:ACE8227
Scopula confinaria|Greece.Peloponnese|BOLD:ACF4768
Scopula confinaria|Greece|BOLD:ACF4768
Scopula confinaria|Italy.Sicily|BOLD:AAD1171
Scopula confinaria|Italy.Sicily|BOLD:AAD1171
Scopula confinaria|Italy.Sicily|BOLD:AAD1171
Scopula alba|France|BOLD:AAD1171
Scopula confinaria|Greece.Epirus|BOLD:AAD1171

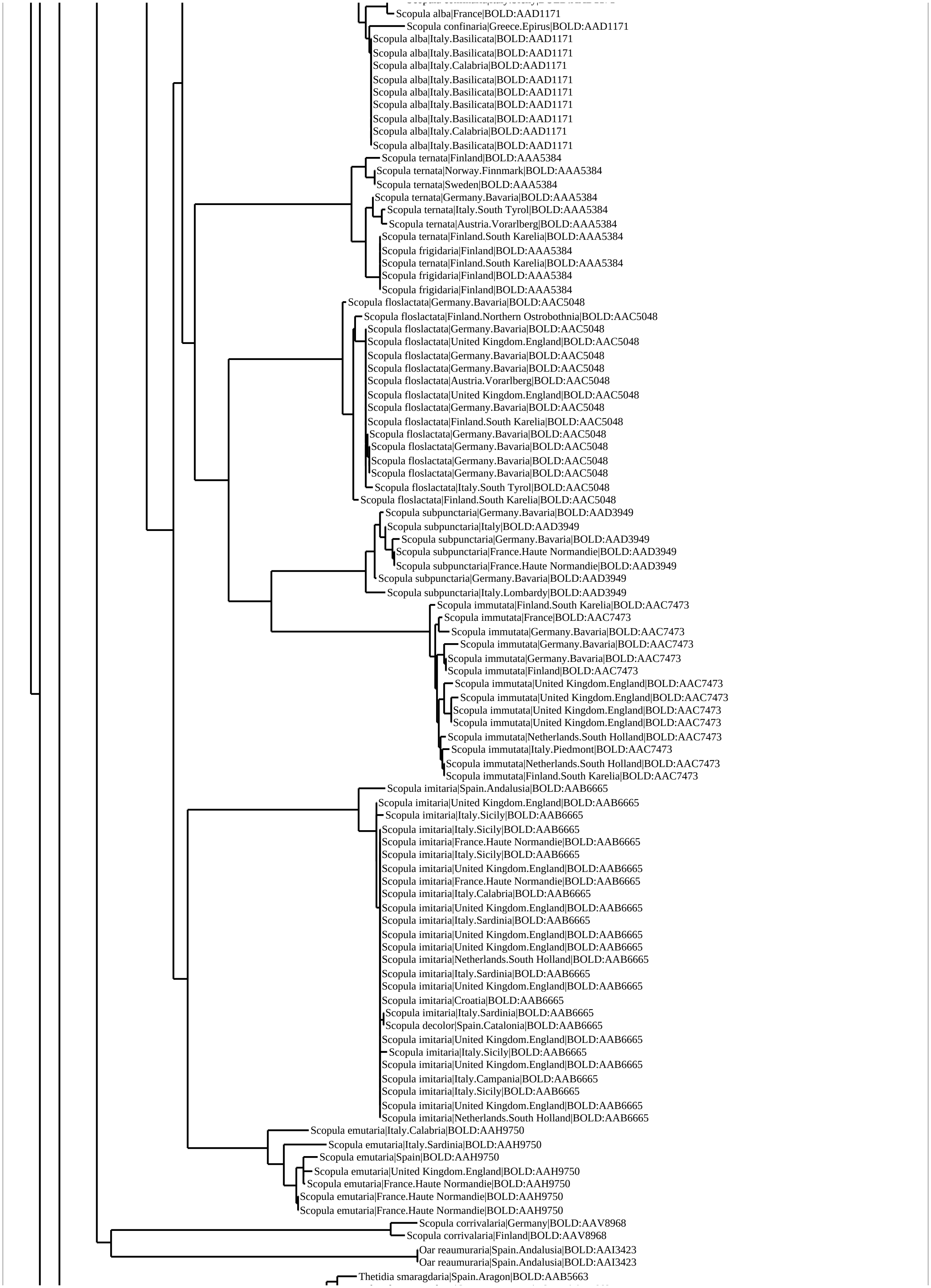
Scopula alba|France|BOLD:AAD1171
Scopula confinaria|Greece.Epirus|BOLD:AAD1171
Scopula alba|Italy.Basilicata|BOLD:AAD1171
Scopula alba|Italy.Basilicata|BOLD:AAD1171
Scopula alba|Italy.Calabria|BOLD:AAD1171
Scopula alba|Italy.Basilicata|BOLD:AAD1171
Scopula alba|Italy.Basilicata|BOLD:AAD1171
Scopula alba|Italy.Basilicata|BOLD:AAD1171
Scopula alba|Italy.Basilicata|BOLD:AAD1171
Scopula alba|Italy.Calabria|BOLD:AAD1171
Scopula alba|Italy.Basilicata|BOLD:AAD1171
Scopula ternata|Finland|BOLD:AAA5384
Scopula ternata|Norway.Finnmark|BOLD:AAA5384
Scopula ternata|Sweden|BOLD:AAA5384
Scopula ternata|Germany.Bavaria|BOLD:AAA5384
Scopula ternata|Italy.South Tyrol|BOLD:AAA5384
Scopula ternata|Austria.Vorarlberg|BOLD:AAA5384
Scopula ternata|Finland.South Karelia|BOLD:AAA5384
Scopula frigidaria|Finland|BOLD:AAA5384
Scopula ternata|Finland.South Karelia|BOLD:AAA5384
Scopula frigidaria|Finland|BOLD:AAA5384
Scopula frigidaria|Finland|BOLD:AAA5384
Scopula floslactata|Germany.Bavaria|BOLD:AAC5048
Scopula floslactata|Finland.Northern Ostrobothnia|BOLD:AAC5048
Scopula floslactata|Germany.Bavaria|BOLD:AAC5048
Scopula floslactata|United Kingdom.England|BOLD:AAC5048
Scopula floslactata|Germany.Bavaria|BOLD:AAC5048
Scopula floslactata|Germany.Bavaria|BOLD:AAC5048
Scopula floslactata|Austria.Vorarlberg|BOLD:AAC5048
Scopula floslactata|United Kingdom.England|BOLD:AAC5048
Scopula floslactata|Germany.Bavaria|BOLD:AAC5048
Scopula floslactata|Finland.South Karelia|BOLD:AAC5048
Scopula floslactata|Germany.Bavaria|BOLD:AAC5048
Scopula floslactata|Germany.Bavaria|BOLD:AAC5048
Scopula floslactata|Germany.Bavaria|BOLD:AAC5048
Scopula floslactata|Germany.Bavaria|BOLD:AAC5048
Scopula floslactata|Italy.South Tyrol|BOLD:AAC5048
Scopula floslactata|Finland.South Karelia|BOLD:AAC5048
Scopula subpunctaria|Germany.Bavaria|BOLD:AAD3949
Scopula subpunctaria|Italy|BOLD:AAD3949
Scopula subpunctaria|Germany.Bavaria|BOLD:AAD3949
Scopula subpunctaria|France.Haute Normandie|BOLD:AAD3949
Scopula subpunctaria|France.Haute Normandie|BOLD:AAD3949
Scopula subpunctaria|Germany.Bavaria|BOLD:AAD3949
Scopula subpunctaria|Italy.Lombardy|BOLD:AAD3949
Scopula immutata|Finland.South Karelia|BOLD:AAC7473
Scopula immutata|France|BOLD:AAC7473
Scopula immutata|Germany.Bavaria|BOLD:AAC7473
Scopula immutata|Germany.Bavaria|BOLD:AAC7473
Scopula immutata|Germany.Bavaria|BOLD:AAC7473
Scopula immutata|Finland|BOLD:AAC7473
Scopula immutata|United Kingdom.England|BOLD:AAC7473
Scopula immutata|United Kingdom.England|BOLD:AAC7473
Scopula immutata|United Kingdom.England|BOLD:AAC7473
Scopula immutata|United Kingdom.England|BOLD:AAC7473
Scopula immutata|Netherlands.South Holland|BOLD:AAC7473
Scopula immutata|Italy.Piedmont|BOLD:AAC7473
Scopula immutata|Netherlands.South Holland|BOLD:AAC7473
Scopula immutata|Finland.South Karelia|BOLD:AAC7473
Scopula imitaria|Spain.Andalusia|BOLD:AAB6665
Scopula imitaria|United Kingdom.England|BOLD:AAB6665
Scopula imitaria|Italy.Sicily|BOLD:AAB6665
Scopula imitaria|Italy.Sicily|BOLD:AAB6665
Scopula imitaria|France.Haute Normandie|BOLD:AAB6665
Scopula imitaria|Italy.Sicily|BOLD:AAB6665
Scopula imitaria|United Kingdom.England|BOLD:AAB6665
Scopula imitaria|France.Haute Normandie|BOLD:AAB6665
Scopula imitaria|Italy.Calabria|BOLD:AAB6665
Scopula imitaria|United Kingdom.England|BOLD:AAB6665
Scopula imitaria|Italy.Sardinia|BOLD:AAB6665
Scopula imitaria|United Kingdom.England|BOLD:AAB6665
Scopula imitaria|United Kingdom.England|BOLD:AAB6665
Scopula imitaria|Netherlands.South Holland|BOLD:AAB6665
Scopula imitaria|Italy.Sardinia|BOLD:AAB6665
Scopula imitaria|United Kingdom.England|BOLD:AAB6665
Scopula imitaria|Croatia|BOLD:AAB6665
Scopula imitaria|Italy.Sardinia|BOLD:AAB6665
Scopula decolor|Spain.Catalonia|BOLD:AAB6665
Scopula imitaria|United Kingdom.England|BOLD:AAB6665
Scopula imitaria|Italy.Sicily|BOLD:AAB6665
Scopula imitaria|United Kingdom.England|BOLD:AAB6665
Scopula imitaria|Italy.Campania|BOLD:AAB6665
Scopula imitaria|Italy.Sicily|BOLD:AAB6665
Scopula imitaria|United Kingdom.England|BOLD:AAB6665
Scopula imitaria|Netherlands.South Holland|BOLD:AAB6665
Scopula emutaria|Italy.Calabria|BOLD:AAH9750
Scopula emutaria|Italy.Sardinia|BOLD:AAH9750
Scopula emutaria|Spain|BOLD:AAH9750
Scopula emutaria|United Kingdom.England|BOLD:AAH9750
Scopula emutaria|France.Haute Normandie|BOLD:AAH9750
Scopula emutaria|France.Haute Normandie|BOLD:AAH9750
Scopula emutaria|France.Haute Normandie|BOLD:AAH9750
Scopula corrivalaria|Germany|BOLD:AAV8968
Scopula corrivalaria|Finland|BOLD:AAV8968
Oar reaumuraria|Spain.Andalusia|BOLD:AAI3423
Oar reaumuraria|Spain.Andalusia|BOLD:AAI3423
Thetidia smaragdaria|Spain.Aragon|BOLD:AAB5663

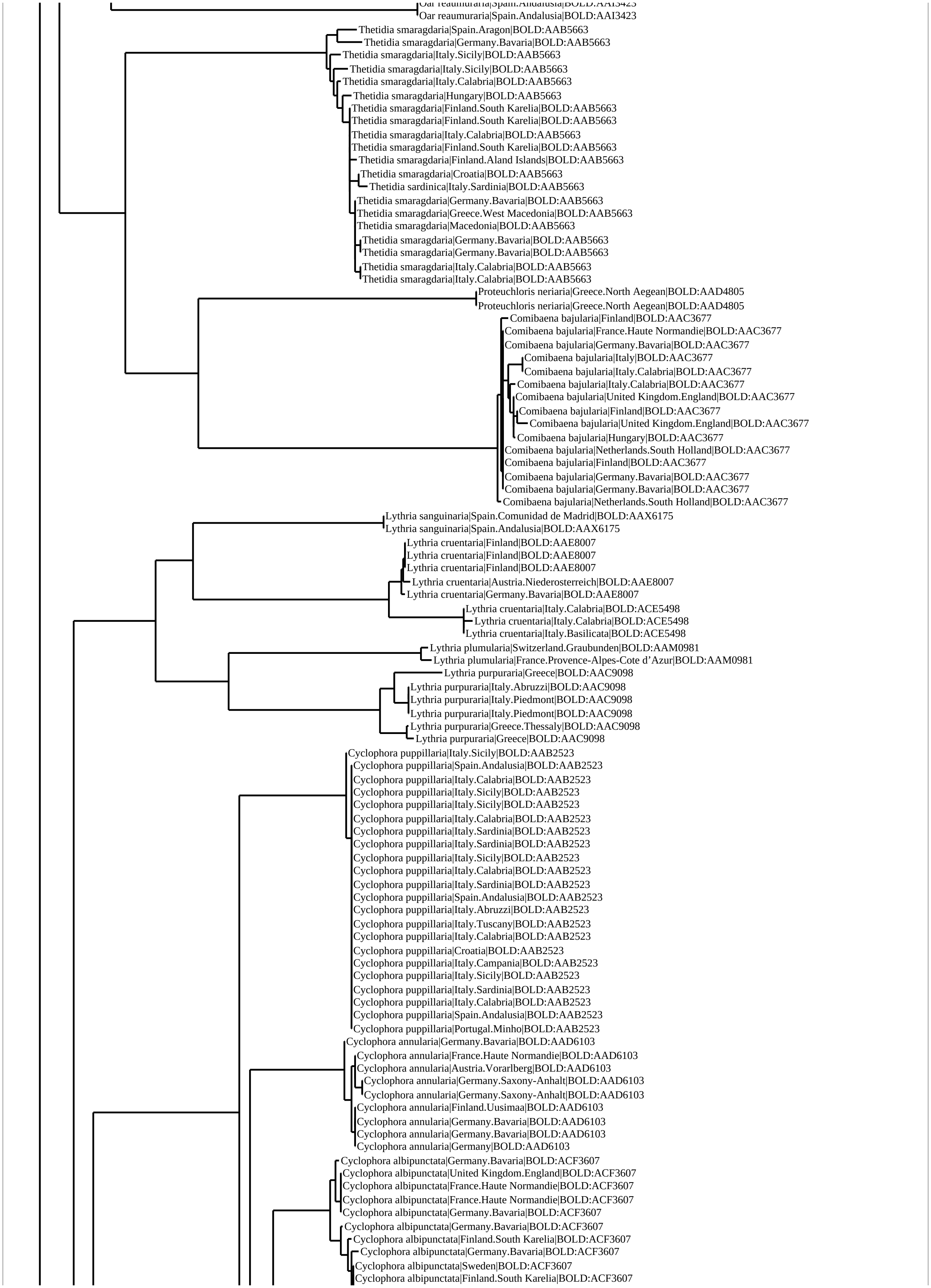
Oar reaumuraria|Spain.Andalusia|BOLD:AAI3423
Oar reaumuraria|Spain.Andalusia|BOLD:AAI3423
Thetidia smaragdaria|Spain.Aragon|BOLD:AAB5663
Thetidia smaragdaria|Germany.Bavaria|BOLD:AAB5663
Thetidia smaragdaria|Italy.Sicily|BOLD:AAB5663
Thetidia smaragdaria|Italy.Sicily|BOLD:AAB5663
Thetidia smaragdaria|Italy.Calabria|BOLD:AAB5663
Thetidia smaragdaria|Hungary|BOLD:AAB5663
Thetidia smaragdaria|Finland.South Karelia|BOLD:AAB5663
Thetidia smaragdaria|Finland.South Karelia|BOLD:AAB5663
Thetidia smaragdaria|Italy.Calabria|BOLD:AAB5663
Thetidia smaragdaria|Finland.South Karelia|BOLD:AAB5663
Thetidia smaragdaria|Finland.Aland Islands|BOLD:AAB5663
Thetidia smaragdaria|Croatia|BOLD:AAB5663
Thetidia sardinica|Italy.Sardinia|BOLD:AAB5663
Thetidia smaragdaria|Germany.Bavaria|BOLD:AAB5663
Thetidia smaragdaria|Greece.West Macedonia|BOLD:AAB5663
Thetidia smaragdaria|Macedonia|BOLD:AAB5663
Thetidia smaragdaria|Germany.Bavaria|BOLD:AAB5663
Thetidia smaragdaria|Germany.Bavaria|BOLD:AAB5663
Thetidia smaragdaria|Italy.Calabria|BOLD:AAB5663
Thetidia smaragdaria|Italy.Calabria|BOLD:AAB5663
Proteuchloris neriaria|Greece.North Aegean|BOLD:AAD4805
Proteuchloris neriaria|Greece.North Aegean|BOLD:AAD4805
Comibaena bajularia|Finland|BOLD:AAC3677
Comibaena bajularia|France.Haute Normandie|BOLD:AAC3677
Comibaena bajularia|Germany.Bavaria|BOLD:AAC3677
Comibaena bajularia|Italy|BOLD:AAC3677
Comibaena bajularia|Italy.Calabria|BOLD:AAC3677
Comibaena bajularia|Italy.Calabria|BOLD:AAC3677
Comibaena bajularia|United Kingdom.England|BOLD:AAC3677
Comibaena bajularia|Finland|BOLD:AAC3677
Comibaena bajularia|United Kingdom.England|BOLD:AAC3677
Comibaena bajularia|Hungary|BOLD:AAC3677
Comibaena bajularia|Netherlands.South Holland|BOLD:AAC3677
Comibaena bajularia|Finland|BOLD:AAC3677
Comibaena bajularia|Germany.Bavaria|BOLD:AAC3677
Comibaena bajularia|Germany.Bavaria|BOLD:AAC3677
Comibaena bajularia|Netherlands.South Holland|BOLD:AAC3677
Lythria sanguinaria|Spain.Comunidad de Madrid|BOLD:AAX6175
Lythria sanguinaria|Spain.Andalusia|BOLD:AAX6175
Lythria cruentaria|Finland|BOLD:AAE8007
Lythria cruentaria|Finland|BOLD:AAE8007
Lythria cruentaria|Finland|BOLD:AAE8007
Lythria cruentaria|Austria.Niederosterreich|BOLD:AAE8007
Lythria cruentaria|Germany.Bavaria|BOLD:AAE8007
Lythria cruentaria|Italy.Calabria|BOLD:ACE5498
Lythria cruentaria|Italy.Calabria|BOLD:ACE5498
Lythria cruentaria|Italy.Basilicata|BOLD:ACE5498
Lythria plumularia|Switzerland.Graubunden|BOLD:AAM0981
Lythria plumularia|France.Provence-Alpes-Cote d'Azur|BOLD:AAM0981
Lythria purpuraria|Greece|BOLD:AAC9098
Lythria purpuraria|Italy.Abruzzi|BOLD:AAC9098
Lythria purpuraria|Italy.Piedmont|BOLD:AAC9098
Lythria purpuraria|Italy.Piedmont|BOLD:AAC9098
Lythria purpuraria|Greece.Thessaly|BOLD:AAC9098
Lythria purpuraria|Greece|BOLD:AAC9098
Cyclophora puppillaria|Italy.Sicily|BOLD:AAB2523
Cyclophora puppillaria|Spain.Andalusia|BOLD:AAB2523
Cyclophora puppillaria|Italy.Calabria|BOLD:AAB2523
Cyclophora puppillaria|Italy.Sicily|BOLD:AAB2523
Cyclophora puppillaria|Italy.Sicily|BOLD:AAB2523
Cyclophora puppillaria|Italy.Calabria|BOLD:AAB2523
Cyclophora puppillaria|Italy.Sardinia|BOLD:AAB2523
Cyclophora puppillaria|Italy.Sardinia|BOLD:AAB2523
Cyclophora puppillaria|Italy.Sicily|BOLD:AAB2523
Cyclophora puppillaria|Italy.Calabria|BOLD:AAB2523
Cyclophora puppillaria|Italy.Sardinia|BOLD:AAB2523
Cyclophora puppillaria|Spain.Andalusia|BOLD:AAB2523
Cyclophora puppillaria|Italy.Abruzzi|BOLD:AAB2523
Cyclophora puppillaria|Italy.Tuscany|BOLD:AAB2523
Cyclophora puppillaria|Italy.Calabria|BOLD:AAB2523
Cyclophora puppillaria|Croatia|BOLD:AAB2523
Cyclophora puppillaria|Italy.Campania|BOLD:AAB2523
Cyclophora puppillaria|Italy.Sicily|BOLD:AAB2523
Cyclophora puppillaria|Italy.Sardinia|BOLD:AAB2523
Cyclophora puppillaria|Italy.Calabria|BOLD:AAB2523
Cyclophora puppillaria|Spain.Andalusia|BOLD:AAB2523
Cyclophora puppillaria|Portugal.Minho|BOLD:AAB2523
Cyclophora annularia|Germany.Bavaria|BOLD:AAD6103
Cyclophora annularia|France.Haute Normandie|BOLD:AAD6103
Cyclophora annularia|Austria.Vorarlberg|BOLD:AAD6103
Cyclophora annularia|Germany.Saxony-Anhalt|BOLD:AAD6103
Cyclophora annularia|Germany.Saxony-Anhalt|BOLD:AAD6103
Cyclophora annularia|Finland.Uusimaa|BOLD:AAD6103
Cyclophora annularia|Germany.Bavaria|BOLD:AAD6103
Cyclophora annularia|Germany.Bavaria|BOLD:AAD6103
Cyclophora annularia|Germany|BOLD:AAD6103
Cyclophora albipunctata|Germany.Bavaria|BOLD:ACF3607
Cyclophora albipunctata|United Kingdom.England|BOLD:ACF3607
Cyclophora albipunctata|France.Haute Normandie|BOLD:ACF3607
Cyclophora albipunctata|France.Haute Normandie|BOLD:ACF3607
Cyclophora albipunctata|Germany.Bavaria|BOLD:ACF3607
Cyclophora albipunctata|Germany.Bavaria|BOLD:ACF3607
Cyclophora albipunctata|Finland.South Karelia|BOLD:ACF3607
Cyclophora albipunctata|Germany.Bavaria|BOLD:ACF3607
Cyclophora albipunctata|Sweden|BOLD:ACF3607
Cyclophora albipunctata|Finland.South Karelia|BOLD:ACF3607

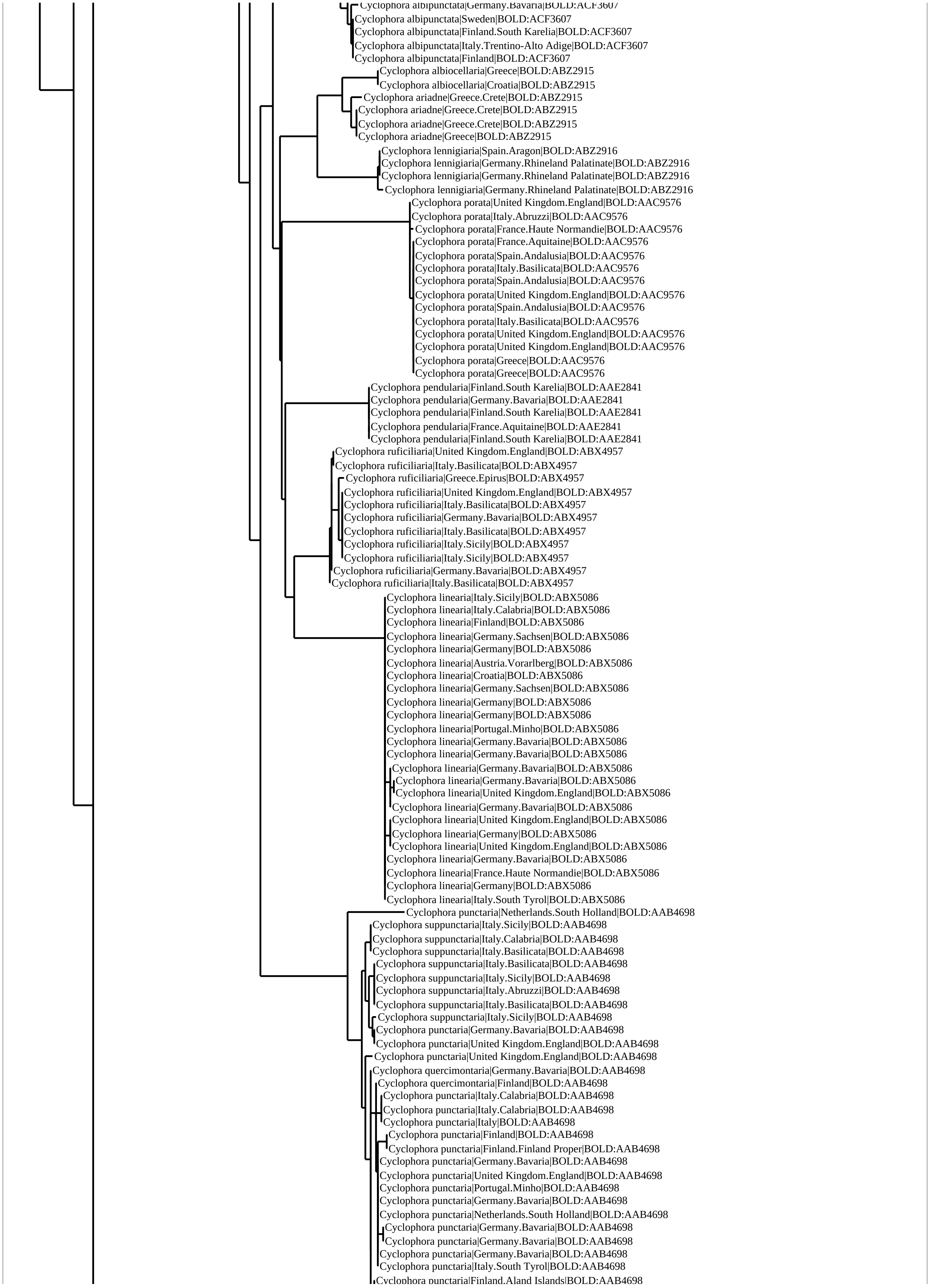
Cyclophora albipunctata|Germany.Bavaria|BOLD:ACF3607
Cyclophora albipunctata|Sweden|BOLD:ACF3607
Cyclophora albipunctata|Finland.South Karelia|BOLD:ACF3607
Cyclophora albipunctata|Italy.Trentino-Alto Adige|BOLD:ACF3607
Cyclophora albipunctata|Finland|BOLD:ACF3607
Cyclophora albiocellaria|Greece|BOLD:ABZ2915
Cyclophora albiocellaria|Croatia|BOLD:ABZ2915
Cyclophora ariadne|Greece.Crete|BOLD:ABZ2915
Cyclophora ariadne|Greece.Crete|BOLD:ABZ2915
Cyclophora ariadne|Greece.Crete|BOLD:ABZ2915
Cyclophora ariadne|Greece|BOLD:ABZ2915
Cyclophora lennigiaria|Spain.Aragon|BOLD:ABZ2916
Cyclophora lennigiaria|Germany.Rhineland Palatinate|BOLD:ABZ2916
Cyclophora lennigiaria|Germany.Rhineland Palatinate|BOLD:ABZ2916
Cyclophora lennigiaria|Germany.Rhineland Palatinate|BOLD:ABZ2916
Cyclophora porata|United Kingdom.England|BOLD:AAC9576
Cyclophora porata|Italy.Abruzzi|BOLD:AAC9576
Cyclophora porata|France.Haute Normandie|BOLD:AAC9576
Cyclophora porata|France.Aquitaine|BOLD:AAC9576
Cyclophora porata|Spain.Andalusia|BOLD:AAC9576
Cyclophora porata|Italy.Basilicata|BOLD:AAC9576
Cyclophora porata|Spain.Andalusia|BOLD:AAC9576
Cyclophora porata|United Kingdom.England|BOLD:AAC9576
Cyclophora porata|Spain.Andalusia|BOLD:AAC9576
Cyclophora porata|Italy.Basilicata|BOLD:AAC9576
Cyclophora porata|United Kingdom.England|BOLD:AAC9576
Cyclophora porata|United Kingdom.England|BOLD:AAC9576
Cyclophora porata|Greece|BOLD:AAC9576
Cyclophora porata|Greece|BOLD:AAC9576
Cyclophora pendularia|Finland.South Karelia|BOLD:AAE2841
Cyclophora pendularia|Germany.Bavaria|BOLD:AAE2841
Cyclophora pendularia|Finland.South Karelia|BOLD:AAE2841
Cyclophora pendularia|France.Aquitaine|BOLD:AAE2841
Cyclophora pendularia|Finland.South Karelia|BOLD:AAE2841
Cyclophora ruficiliaria|United Kingdom.England|BOLD:ABX4957
Cyclophora ruficiliaria|Italy.Basilicata|BOLD:ABX4957
Cyclophora ruficiliaria|Greece.Epirus|BOLD:ABX4957
Cyclophora ruficiliaria|United Kingdom.England|BOLD:ABX4957
Cyclophora ruficiliaria|Italy.Basilicata|BOLD:ABX4957
Cyclophora ruficiliaria|Germany.Bavaria|BOLD:ABX4957
Cyclophora ruficiliaria|Italy.Basilicata|BOLD:ABX4957
Cyclophora ruficiliaria|Italy.Sicily|BOLD:ABX4957
Cyclophora ruficiliaria|Italy.Sicily|BOLD:ABX4957
Cyclophora ruficiliaria|Germany.Bavaria|BOLD:ABX4957
Cyclophora ruficiliaria|Italy.Basilicata|BOLD:ABX4957
Cyclophora linearia|Italy.Sicily|BOLD:ABX5086
Cyclophora linearia|Italy.Calabria|BOLD:ABX5086
Cyclophora linearia|Finland|BOLD:ABX5086
Cyclophora linearia|Germany.Sachsen|BOLD:ABX5086
Cyclophora linearia|Germany|BOLD:ABX5086
Cyclophora linearia|Austria.Vorarlberg|BOLD:ABX5086
Cyclophora linearia|Croatia|BOLD:ABX5086
Cyclophora linearia|Germany.Sachsen|BOLD:ABX5086
Cyclophora linearia|Germany|BOLD:ABX5086
Cyclophora linearia|Germany|BOLD:ABX5086
Cyclophora linearia|Portugal.Minho|BOLD:ABX5086
Cyclophora linearia|Germany.Bavaria|BOLD:ABX5086
Cyclophora linearia|Germany.Bavaria|BOLD:ABX5086
Cyclophora linearia|Germany.Bavaria|BOLD:ABX5086
Cyclophora linearia|Germany.Bavaria|BOLD:ABX5086
Cyclophora linearia|United Kingdom.England|BOLD:ABX5086
Cyclophora linearia|Germany.Bavaria|BOLD:ABX5086
Cyclophora linearia|United Kingdom.England|BOLD:ABX5086
Cyclophora linearia|Germany|BOLD:ABX5086
Cyclophora linearia|United Kingdom.England|BOLD:ABX5086
Cyclophora linearia|Germany.Bavaria|BOLD:ABX5086
Cyclophora linearia|France.Haute Normandie|BOLD:ABX5086
Cyclophora linearia|Germany|BOLD:ABX5086
Cyclophora linearia|Italy.South Tyrol|BOLD:ABX5086
Cyclophora punctaria|Netherlands.South Holland|BOLD:AAB4698
Cyclophora suppunctaria|Italy.Sicily|BOLD:AAB4698
Cyclophora suppunctaria|Italy.Calabria|BOLD:AAB4698
Cyclophora suppunctaria|Italy.Basilicata|BOLD:AAB4698
Cyclophora suppunctaria|Italy.Basilicata|BOLD:AAB4698
Cyclophora suppunctaria|Italy.Sicily|BOLD:AAB4698
Cyclophora suppunctaria|Italy.Abruzzi|BOLD:AAB4698
Cyclophora suppunctaria|Italy.Basilicata|BOLD:AAB4698
Cyclophora suppunctaria|Italy.Sicily|BOLD:AAB4698
Cyclophora punctaria|Germany.Bavaria|BOLD:AAB4698
Cyclophora punctaria|United Kingdom.England|BOLD:AAB4698
Cyclophora punctaria|United Kingdom.England|BOLD:AAB4698
Cyclophora quercimontaria|Germany.Bavaria|BOLD:AAB4698
Cyclophora quercimontaria|Finland|BOLD:AAB4698
Cyclophora punctaria|Italy.Calabria|BOLD:AAB4698
Cyclophora punctaria|Italy.Calabria|BOLD:AAB4698
Cyclophora punctaria|Italy|BOLD:AAB4698
Cyclophora punctaria|Finland|BOLD:AAB4698
Cyclophora punctaria|Finland.Finland Proper|BOLD:AAB4698
Cyclophora punctaria|Germany.Bavaria|BOLD:AAB4698
Cyclophora punctaria|United Kingdom.England|BOLD:AAB4698
Cyclophora punctaria|Portugal.Minho|BOLD:AAB4698
Cyclophora punctaria|Germany.Bavaria|BOLD:AAB4698
Cyclophora punctaria|Netherlands.South Holland|BOLD:AAB4698
Cyclophora punctaria|Germany.Bavaria|BOLD:AAB4698
Cyclophora punctaria|Germany.Bavaria|BOLD:AAB4698
Cyclophora punctaria|Germany.Bavaria|BOLD:AAB4698
Cyclophora punctaria|Italy.South Tyrol|BOLD:AAB4698
Cyclophora punctaria|Finland.Aland Islands|BOLD:AAB4698

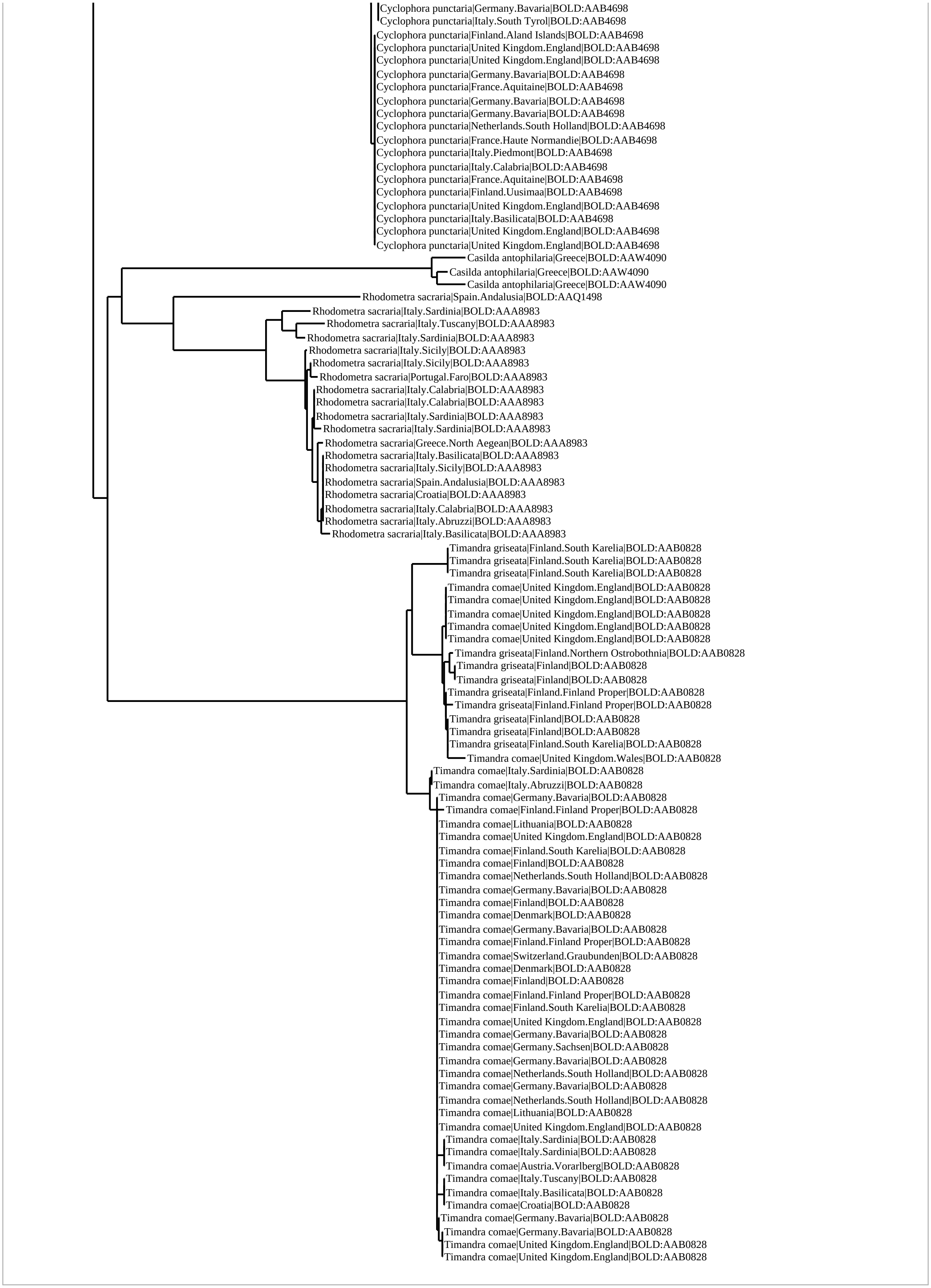
Cyclophora punctaria|Germany.Bavaria|BOLD:AAB4698
Cyclophora punctaria|Italy.South Tyrol|BOLD:AAB4698
Cyclophora punctaria|Finland.Aland Islands|BOLD:AAB4698
Cyclophora punctaria|United Kingdom.England|BOLD:AAB4698
Cyclophora punctaria|United Kingdom.England|BOLD:AAB4698
Cyclophora punctaria|Germany.Bavaria|BOLD:AAB4698
Cyclophora punctaria|France.Aquitaine|BOLD:AAB4698
Cyclophora punctaria|Germany.Bavaria|BOLD:AAB4698
Cyclophora punctaria|Germany.Bavaria|BOLD:AAB4698
Cyclophora punctaria|Netherlands.South Holland|BOLD:AAB4698
Cyclophora punctaria|France.Haute Normandie|BOLD:AAB4698
Cyclophora punctaria|Italy.Piedmont|BOLD:AAB4698
Cyclophora punctaria|Italy.Calabria|BOLD:AAB4698
Cyclophora punctaria|France.Aquitaine|BOLD:AAB4698
Cyclophora punctaria|Finland.Uusimaa|BOLD:AAB4698
Cyclophora punctaria|United Kingdom.England|BOLD:AAB4698
Cyclophora punctaria|Italy.Basilicata|BOLD:AAB4698
Cyclophora punctaria|United Kingdom.England|BOLD:AAB4698
Cyclophora punctaria|United Kingdom.England|BOLD:AAB4698
Casilda antophilaria|Greece|BOLD:AAW4090
Casilda antophilaria|Greece|BOLD:AAW4090
Casilda antophilaria|Greece|BOLD:AAW4090
Rhodometra sacraria|Spain.Andalusia|BOLD:AAQ1498
Rhodometra sacraria|Italy.Sardinia|BOLD:AAA8983
Rhodometra sacraria|Italy.Tuscany|BOLD:AAA8983
Rhodometra sacraria|Italy.Sardinia|BOLD:AAA8983
Rhodometra sacraria|Italy.Sicily|BOLD:AAA8983
Rhodometra sacraria|Italy.Sicily|BOLD:AAA8983
Rhodometra sacraria|Portugal.Faro|BOLD:AAA8983
Rhodometra sacraria|Italy.Calabria|BOLD:AAA8983
Rhodometra sacraria|Italy.Calabria|BOLD:AAA8983
Rhodometra sacraria|Italy.Sardinia|BOLD:AAA8983
Rhodometra sacraria|Italy.Sardinia|BOLD:AAA8983
Rhodometra sacraria|Greece.North Aegean|BOLD:AAA8983
Rhodometra sacraria|Italy.Basilicata|BOLD:AAA8983
Rhodometra sacraria|Italy.Sicily|BOLD:AAA8983
Rhodometra sacraria|Spain.Andalusia|BOLD:AAA8983
Rhodometra sacraria|Croatia|BOLD:AAA8983
Rhodometra sacraria|Italy.Calabria|BOLD:AAA8983
Rhodometra sacraria|Italy.Abruzzi|BOLD:AAA8983
Rhodometra sacraria|Italy.Basilicata|BOLD:AAA8983
Timandra griseata|Finland.South Karelia|BOLD:AAB0828
Timandra griseata|Finland.South Karelia|BOLD:AAB0828
Timandra griseata|Finland.South Karelia|BOLD:AAB0828
Timandra comae|United Kingdom.England|BOLD:AAB0828
Timandra comae|United Kingdom.England|BOLD:AAB0828
Timandra comae|United Kingdom.England|BOLD:AAB0828
Timandra comae|United Kingdom.England|BOLD:AAB0828
Timandra comae|United Kingdom.England|BOLD:AAB0828
Timandra griseata|Finland.Northern Ostrobothnia|BOLD:AAB0828
Timandra griseata|Finland|BOLD:AAB0828
Timandra griseata|Finland|BOLD:AAB0828
Timandra griseata|Finland.Finland Proper|BOLD:AAB0828
Timandra griseata|Finland.Finland Proper|BOLD:AAB0828
Timandra griseata|Finland|BOLD:AAB0828
Timandra griseata|Finland|BOLD:AAB0828
Timandra griseata|Finland.South Karelia|BOLD:AAB0828
Timandra comae|United Kingdom.Wales|BOLD:AAB0828
Timandra comae|Italy.Sardinia|BOLD:AAB0828
Timandra comae|Italy.Abruzzi|BOLD:AAB0828
Timandra comae|Germany.Bavaria|BOLD:AAB0828
Timandra comae|Finland.Finland Proper|BOLD:AAB0828
Timandra comae|Lithuania|BOLD:AAB0828
Timandra comae|United Kingdom.England|BOLD:AAB0828
Timandra comae|Finland.South Karelia|BOLD:AAB0828
Timandra comae|Finland|BOLD:AAB0828
Timandra comae|Netherlands.South Holland|BOLD:AAB0828
Timandra comae|Germany.Bavaria|BOLD:AAB0828
Timandra comae|Finland|BOLD:AAB0828
Timandra comae|Denmark|BOLD:AAB0828
Timandra comae|Germany.Bavaria|BOLD:AAB0828
Timandra comae|Finland.Finland Proper|BOLD:AAB0828
Timandra comae|Switzerland.Graubunden|BOLD:AAB0828
Timandra comae|Denmark|BOLD:AAB0828
Timandra comae|Finland|BOLD:AAB0828
Timandra comae|Finland.Finland Proper|BOLD:AAB0828
Timandra comae|Finland.South Karelia|BOLD:AAB0828
Timandra comae|United Kingdom.England|BOLD:AAB0828
Timandra comae|Germany.Bavaria|BOLD:AAB0828
Timandra comae|Germany.Sachsen|BOLD:AAB0828
Timandra comae|Germany.Bavaria|BOLD:AAB0828
Timandra comae|Netherlands.South Holland|BOLD:AAB0828
Timandra comae|Germany.Bavaria|BOLD:AAB0828
Timandra comae|Netherlands.South Holland|BOLD:AAB0828
Timandra comae|Lithuania|BOLD:AAB0828
Timandra comae|United Kingdom.England|BOLD:AAB0828
Timandra comae|Italy.Sardinia|BOLD:AAB0828
Timandra comae|Italy.Sardinia|BOLD:AAB0828
Timandra comae|Austria.Vorarlberg|BOLD:AAB0828
Timandra comae|Italy.Tuscany|BOLD:AAB0828
Timandra comae|Italy.Basilicata|BOLD:AAB0828
Timandra comae|Croatia|BOLD:AAB0828
Timandra comae|Germany.Bavaria|BOLD:AAB0828
Timandra comae|Germany.Bavaria|BOLD:AAB0828
Timandra comae|United Kingdom.England|BOLD:AAB0828
Timandra comae|United Kingdom.England|BOLD:AAB0828
